# Supplementary figures and images for: Patient perspectives of the Self-management and Educational Technology tool for Atrial Fibrillation (SETAF): A mixed-methods study in Singapore
Source: PLoS One. 2022 Jan 21;17(1):e0262033. doi: 10.1371/journal.pone.0262033 (PMC8782297; doi:10.1371/journal.pone.0262033)

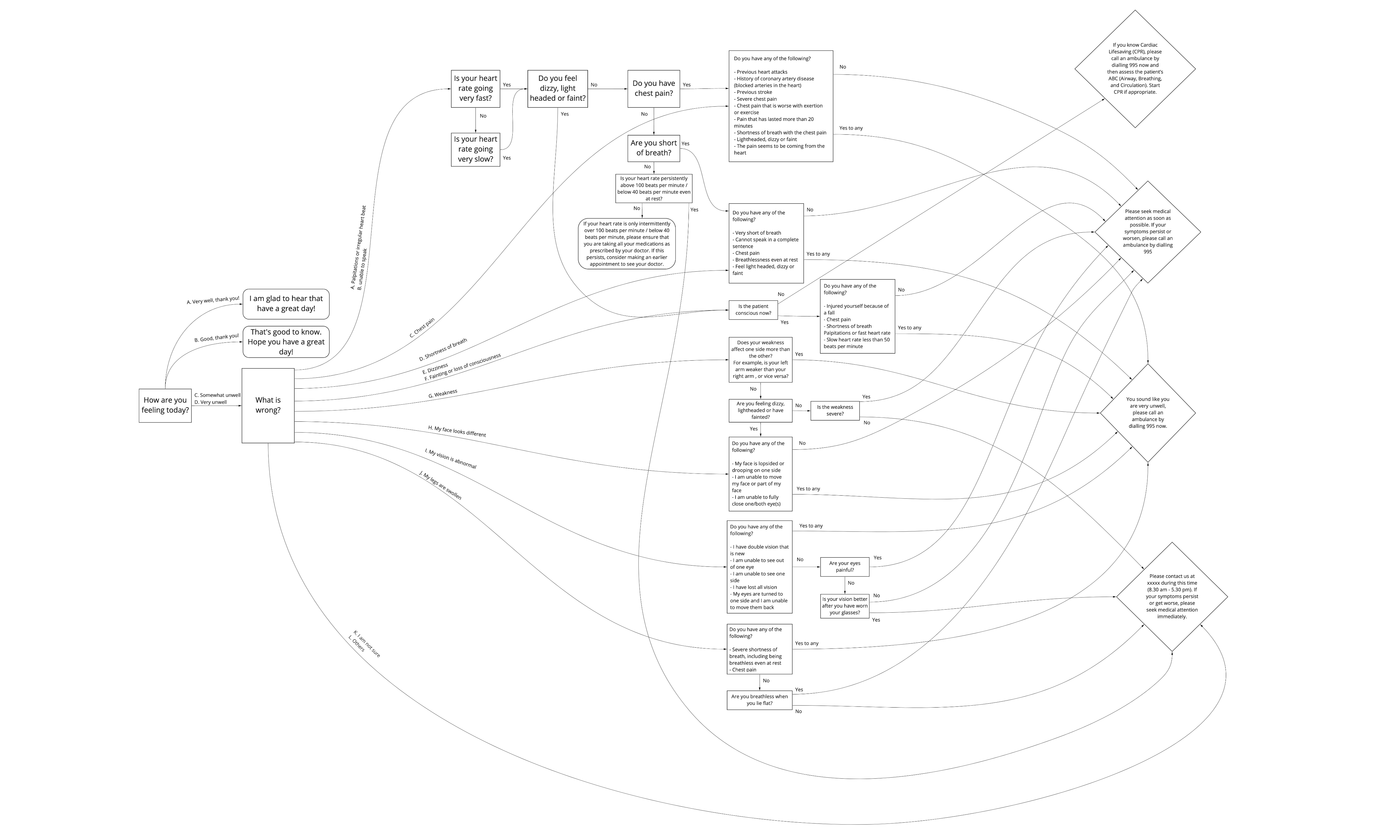

Supplement: S1 Fig — (TIF) [file pone.0262033.s001.tif]

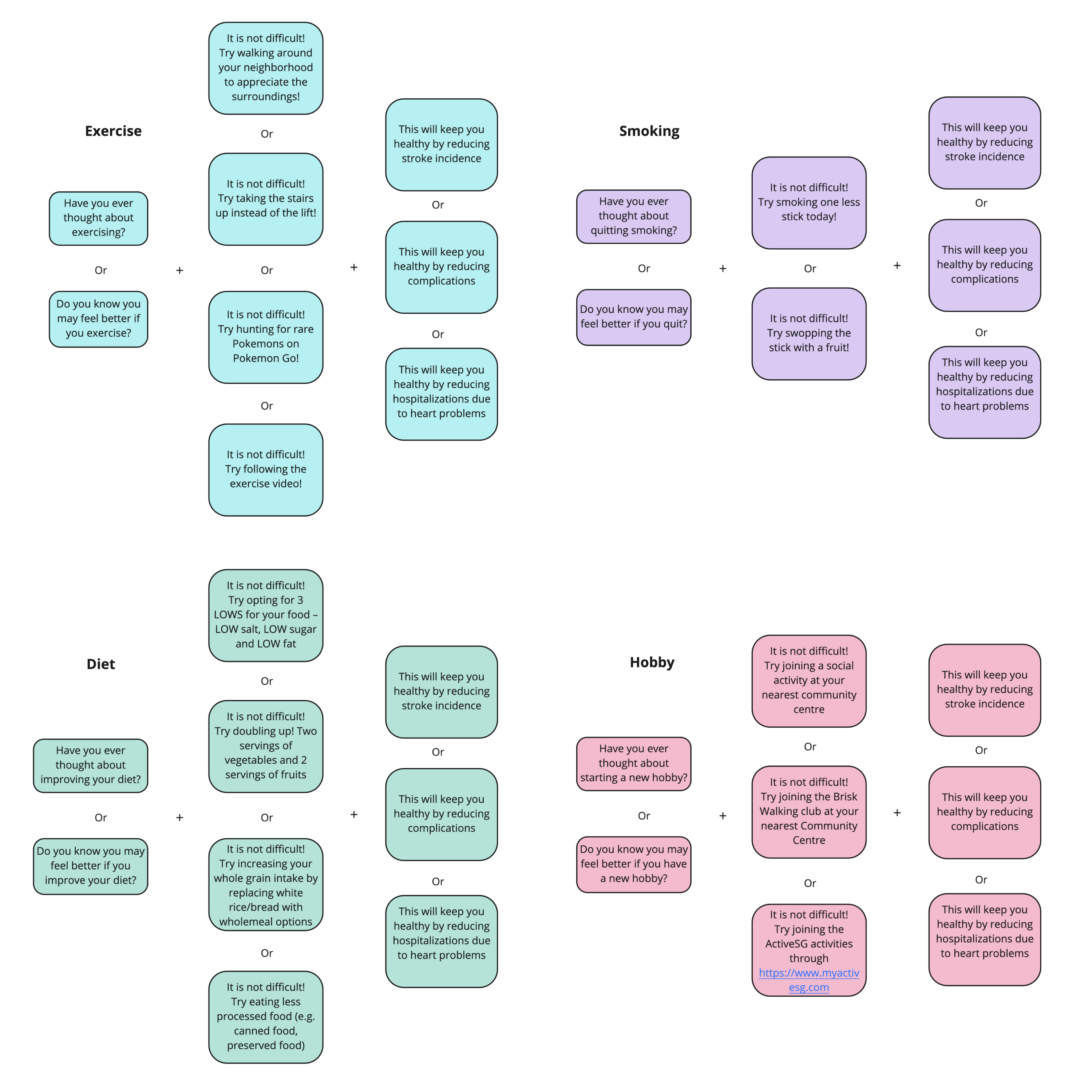

Supplement: S2 Fig — (TIF) [file pone.0262033.s002.tif]
