# Supplementary material for: Patient perspectives of the Self-management and Educational Technology tool for Atrial Fibrillation (SETAF): A mixed-methods study in Singapore
Source: PLoS One. 2022 Jan 21;17(1):e0262033. doi: 10.1371/journal.pone.0262033 (PMC8782297; doi:10.1371/journal.pone.0262033)
Supplement: S1 File — (DOCX) [file pone.0262033.s003.docx]

| Development of a self-management E-Health tool for atrial fibrillation (AF) through a user centred approach | 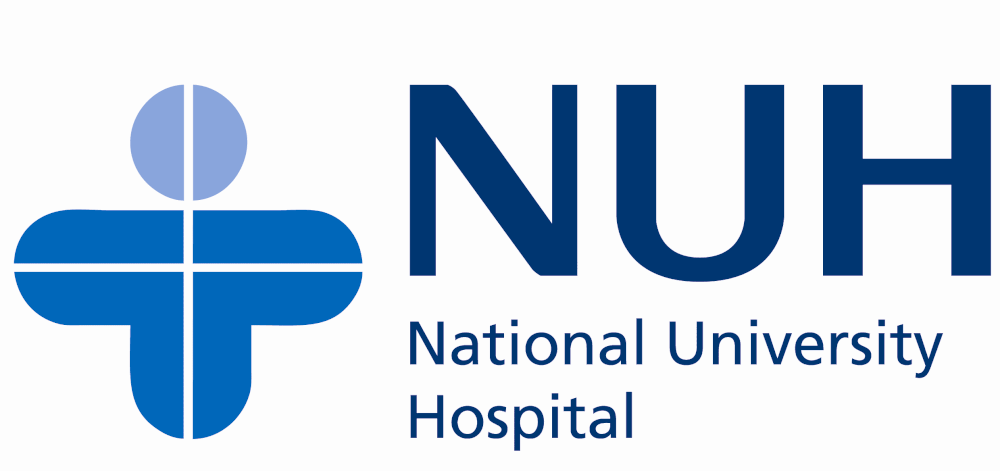 |
| --- | --- |

Dear participant, we would like to gather your opinions after the use of this tablet in managing your atrial fibrillation (AF) condition. This survey will take no longer than 5 minutes and your responses will greatly help us in understanding the needs of AF patients from the patients’ perspective ☺

| Participant ID: |  | Date: |
| --- | --- | --- |

# I use the tablet…

|  | Rarely (0-1 days a week) |  | Occasionally (2-3 days) |  | Frequently (4-5 days a week) |  | Almost every day (6-7 days a week) |
| --- | --- | --- | --- | --- | --- | --- | --- |

# I think the tablet is useful in helping me manage AF

|  | Strong agree |  | Agree |  | Neutral |  | Disagree |  | Strong Disagree |
| --- | --- | --- | --- | --- | --- | --- | --- | --- | --- |

# I would like to continue to use the tablet to manage AF

|  | Strong agree |  | Agree |  | Neutral |  | Disagree |  | Strong Disagree |
| --- | --- | --- | --- | --- | --- | --- | --- | --- | --- |

# I am willing to pay $___________ to continue to use the tablet to manage AF.

# Other comments:

Thank you very much for taking the time to complete this survey. Your feedback is valued and very much appreciated!
