# Supplementary material for: Patient perspectives of the Self-management and Educational Technology tool for Atrial Fibrillation (SETAF): A mixed-methods study in Singapore
Source: PLoS One. 2022 Jan 21;17(1):e0262033. doi: 10.1371/journal.pone.0262033 (PMC8782297; doi:10.1371/journal.pone.0262033)
Supplement: S3 File — (DOCX) [file pone.0262033.s005.docx]

**Revised questions for AF IDI**

| **Getting To Know The Participant** |
| --- |
| - How long have you had atrial fibrillation? - How have you managed your condition before this tablet? [Probe for technological methods] - How comfortable are you in using technology for personal use? [Technology: Devices such as tablet, mobile phone, computer, or other types, with software and app (ef Facebook] in them] Why do you say so? - Do you think that such technology can better help you manage your condition? - Would you say that you prefer in general? Why? |
| **General usage and overall experience**  Let’s move on to talk about the tablet |
| - Describe to me how you use this device on a typical day   - Probe: frequency of use; what does the user do first - sequence of use, reasons for such sequence (?); time of use   - Why do you prefer to use the device - Have you encountered any problems using the tool? - If yes: what do you normally do when facing any problems?   - If yes: who did you ask for help and why? [probe: calls to our team, family members]   - How was the problem(s) resolved? - Did you feel comfortable navigating though the app?   - Which aspects were easy?   - Which aspects were difficult? |
| **Feedback in general on Interactions with Philips (including learning from Philips, availability of contacts)** |
| - Tell us about your interaction with the **Philips team**   - Did they teach you how to use the device?   - Did it take long?   - Was it easy or difficult? - If the team did not teach you how to use the device,would you still feel you’ll be able to use it on your own? - Tell us about your interaction with the **medical team**   - Are you aware that there is a contact number to call if you need any help?   - Did you use the contact number?   - If yes: What was the problem you have experienced? Did calling the number help?   - If no: Was the information about the contact number relayed to you? - Did you mention or talk subsequently about the tablet with your doctors or any other healthcare providers when you see them? Why or why not?   - If yes: was the tool helpful in your communication with them? |
| **Features of different aspects of app**  Now let’s talk about the specific features of the app |
| - How was your experience with the **blood pressure monitor** [Probe for various emotions: easy/difficult, stress/anxiety, happy to know results] - What about your experiences about the **survey** sent to yor device? [Probe for their preference of length, quantity, opinions about having feedback] - Did you had any time to watch the **videos**? Which were the ones you liked the most and why? - How useful were the **pop-up messages**? [Probe: level of motivation, emotions when seeing the messages] - Are there any other features that you would to see in the tablet that would help you in your condition? - In general, how do you feel about the content in the tablet? [Probe for: level of difficulty, quality and quantity] |
| **Usage acceptability and feedback** |
| - Do you think you would be willing to use the device for more than 6 weeks in terms of managing your condition? If so, how long? [Probe: duration was it too long or short] - If given the opportunity to use a device that could help you with your condition, how much time would you feel you would be willing to spend on it? Why? - Would you prefer this to be in a tablet, phone app, computer? - Would you prefer the contents to be delivered to you electronically or non-electronically like in a book or paper form? |
| **Appropriateness and timing of introduction of use of device** |
| - Let’s say the device was introduced to you when you were first diagnosed with AF, do you think it would be helpful for you? If so, why? - Let’s say the device was introduced to you after you have AF, when you are taking medication and seeing the doctor or pharmacist, do you think it would be helpful for you? - What would you say would be the best time for device to be introduced to you since you knew you had AF? |
