# Supplementary material for: Patient perspectives of the Self-management and Educational Technology tool for Atrial Fibrillation (SETAF): A mixed-methods study in Singapore
Source: PLoS One. 2022 Jan 21;17(1):e0262033. doi: 10.1371/journal.pone.0262033 (PMC8782297; doi:10.1371/journal.pone.0262033)
Supplement: S1 Data — (ZIP) [file pone.0262033.s006.zip › AFP2_IDI_0035.doc]

Participant ID number : AFP2_0035

Interviewer Number : I_01

Length of interview : 22.24 mins

I_01 : Ok, thank you for taking part in this study so the first question is how long have you been diagnosed with irregular heartbeat and how did you find out about it?

AFP2_0035 : Diagnosed with what, I feel ok, I I’ve been diagnosed for almost 2 years.

I_01 : Ok, ok what how did you find out about it?

AFP2_0035 : I went to see the school for me to continue on the medication.

I_01 : How did you find out, in the doctor?

AFP2_0035 : Doctor…

I_01 : Ok.

AFP2_0035 : When I have the spasm.

I_01 : Ok, at the hospital ok then, is it at the clinic?

AFP2_0035 : No heart clinic…

I_01 : You…

AFP2_0035 : Heart, heart clinic…

I_01 : Ok, ok you…

AFP2_0035 : There.

I_01 : Found out there, ok then the do you get take any medication or not?

AFP2_0035 : Yah continuously take taking medicine.

I_01 : Ok, what kind of medicine?

AFP2_0035 : [Incomprehensible segment]

I_01 : Warfarin, do you take?

AFP2_0035 : Warfarin I take.

I_01 : Warfarin you take, yah.

AFP2_0035 : Warfarin…

I_01 : Ok.

AFP2_0035 : Then now never ask, many things 5, 5, 5 types.

I_01 : Ok, then you when you where do you get normally get your like information and advice from like you know you want to other than your doctor right and other than the hospital right where do you normally like get information to manage your condition?

AFP2_0035 : Should be pharmacist.

I_01 : Pharmacist ok, then what about like your friends and family… to get information you know they, they help you or not?

AFP2_0035 : I think they, they don’t know nothing they just allow me to go [Incomprehensible segment] at the doctor ask for me to…

I_01 : So usually is the doctor and the pharmacist, then do you like read newspaper, Tv all these to get information or not?

AFP2_0035 : Actually Tv.

I_01 : Tv ah, to get information about your condition?

AFP2_0035 : No, not about all these about other…

I_01 : About other things.

AFP2_0035 : About others.

I_01 : Like, like what?

AFP2_0035 : Like, like those people got chronic disease, I think.

I_01 : Chronic disease ok, ok then do you like go on the computer do you use your computer to get information or, or mobile phone?

AFP2_0035 : No, no, no I just rely on more hospital N doctor that’s all.

I_01 : Hospital N doctor ok, ok, ok so let’s you know during this, this study was about 6 weeks how did you normally everyday you said you take everyday right how did you do it, how did you what did you do first?

AFP2_0035 : When I go and take sometimes I mean sometimes early morning and sometimes late… [Participant laughed]

I_01 : So usually is… morning?

AFP2_0035 : Usually, usually morning.

I_01 : Do you take at other times at any other day, any other…

AFP2_0035 : Maybe in the afternoon, sometimes.

I_01 : Afternoon sometimes ok, then how what did you do first, normally?

AFP2_0035 : Do what?

I_01 : Like you what, what is the first step you, you, you like is it…

AFP2_0035 : When I free I, I just do.

I_01 : You like you just go you just strapped use strap and then you just take ok, then do you have any feelings when you take when you take it, do you find how do you find it?

AFP2_0035 : Just feel normal I don’t feel anything. [Participant laughed]

I_01 : Don’t feel anything, do you feel like you have to do it very I mean like ok is it too many times you have to do it or like too little or just right?

AFP2_0035 : Just do it when I feel like doing it.

I_01 : Just feel like doing when then you do, any particular thing that you like about the this, this, this device the, the blood pressure monitor and the app?

AFP2_0035 : I think this…

I_01 : Anything you like?

AFP2_0035 : Anything I like never, never use also it’s ok because last time also I’ve been using it but I used writing…

I_01 : Writing, last time?

AFP2_0035 : Writing on my on my report on my own.

I_01 : Ok, ok so when you write the.. you write you then you sent to your doctor or what?

AFP2_0035 : No, no for my own this…

I_01 : For you own oh ok, ok you, you prefer the writing one or you prefer the, the electronic one?

AFP2_0035 : Prefer the writing, it’s easier.

I_01 : Oh easier ok, so the, the you use you just record what do you record for the writing one?

AFP2_0035 : The record is what is shown in there, that’s it.

I_01 : Blood pres- blood pressure?

AFP2_0035 : Blood pressure.

I_01 : Any other thing that you record?

AFP2_0035 : No.

I_01 : Ok, ok so you you’re more comfortable with that, why you more comfortable is it you…

AFP2_0035 : To measure my blood pressure, nice I wrote down.

I_01 : But, but you know it’s, it’s you say you write why you prefer writing than electronic, electronic form?

AFP2_0035 : Elect- electronic, sometimes maybe is low the battery low whatever then because then also write.

I_01 : So battery low, so you have when you used that time right this period of time there were a number of times when the battery was low?

AFP2_0035 : No, I never use it, when it getting under 50 I, I charged it first.

I_01 : So you, you will charge it, do you find that the charging is like very long or ok?

AFP2_0035 : It’s ok...

I_01 : It’s ok.

AFP2_0035 : Maybe 2, 2 hours only.

I_01 : Ok, ok then anything you don’t like about this, this product this app and the blood pressure monitor?

AFP2_0035 : I think it’s useful also because when you do your blood pressure it’s, it’s registered inside the app.

I_01 : Ok, ok anything you don’t like about it?

AFP2_0035 : I don’t like?

I_01 : Yah.

AFP2_0035 : This one never use because I seldom use this one.

I_01 : You seldom use but, but, but when you used you got anything that you feel you don’t really find it why, why you seldom use?

AFP2_0035 : Because, because I just rely on doctor when doctor check me that’s all.

I_01 : But on your own you how come you don’t feel you don’t feel like using?

AFP2_0035 : I have my own…

I_01 : Huh?

AFP2_0035 : I have my own, now…

I_01 : You have your own, ah?

AFP2_0035 : Own machine that I...

I_01 : Ok your own machine, so you but do you know that it’s the is it the same one this take blood pressure one?

AFP2_0035 : Something like this but that one is I don’t know what brand is that?

I_01 : Ok, it’s a different brand?

AFP2_0035 : Different brand.

I_01 : Ok, so you, you when you, you, you take your own already right so how come you feel that this one is different because you said that you, you, you take your own you have your blood pressure monitor right so how come you feel that this, this, this program is a bit different?

AFP2_0035 : Different is in writing and in electronic this one the difference.

I_01 : The, the so how about like the, the you know when you take the blood pressure right automatically automatic it will go inside the so how do you find that?

AFP2_0035 : This electronic is quite fast but when you want to this one also showing the graph, graph…

I_01 : The graph correct, so we so you…

AFP2_0035 : This is ok, this one is nice.

I_01 : Ok, but you said you men- you still prefer the writing the writing one?

AFP2_0035 : Yes, I just want to see the, the level.

I_01 : The level ok, ok but the but any reason why writing is better than, than, than, than electronic, anything you can think of?

AFP2_0035 : This one for my own personal reason that’s why, I in fact using this tablet is for people to study but, but I don’t, don’t have such…

I_01 : You don’t ok, ok then ok anyway we wa- because you, you did you did managed to used it a bit right so we want to ask a little bit about the different things that there are in the app and the blood pressure monitor that you like and don’t like. So we’ll go through one by one ok, so the first one is the, the video did you watch the video or not?

AFP2_0035 : The video I think not much useful. [Participant laughed]

I_01 : Oh why, why not useful?

AFP2_0035 : Because I don’t know nothing about it I never study them I, I only see the, the, the notes that’s all.

I_01 : Ok, but the you know the video give you some information right?

AFP2_0035 : Yah.

I_01 : So, how come you find it what, what you not like about it?

AFP2_0035 : The video is I don’t know what is it really and... [Participant laughed]

I_01 : You don’t know what is it really, did you manage to watch any video, did you watch the video?

AFP2_0035 : I don’t watch.

I_01 : Ok, which one you watch?

AFP2_0035 : I watch about the heart, this one…

I_01 : Oh, the heart one!

AFP2_0035 : Because I have problem with it, my heart.

I_01 : How do you find the video?

AFP2_0035 : Video is ok.

I_01 : Did you watch the other videos?

AFP2_0035 : No.

I_01 : So you only watched one video?

AFP2_0035 : Yah.

I_01 : Oh the, the heart one…

AFP2_0035 : Yah the heart.

I_01 : The cooking one, exercise one you didn’t watch?

AFP2_0035 : Yah, I don’t.

I_01 : Ok, ok so the video right the heart one you find that I mean did you find it too long, too short how is it?

AFP2_0035 : It’s too short.

I_01 : It’s too short, so you prefer more the…

AFP2_0035 : I think more or more of it but it’s not, not matter to me [Participant laughed] I mean I don’t really enjoy it.

I_01 : You don’t enjoy the video ok, is it too boring?

AFP2_0035 : Yah, in fact I don’t know anything about this.

I_01 : So let’s say if got somebody to guide you about the video because I know you don’t know much about the like you know these all these condition and I mean like in terms of watching video getting information from video right? Let’s say if got somebody come and maybe guide you a bit is it ok, do you feel more comfortable?

AFP2_0035 : No need, I don’t think of that.

I_01 : I mean not being real- we won’t do it to you but we I we just want to know like is there a better way to make it like more comfortable for people like you, you know?

AFP2_0035 : It’s, it’s I don’t think I need people to guide me what, what is this to is this a lesson to me or what I don’t want like that.

I_01 : You, you don’t want ok why, why don’t want like because you said that you don’t watch the video right?

AFP2_0035 : Yah.

I_01 : Then if somebody can guide you, you also don’t want?

AFP2_0035 : Don’t want.

I_01 : Why, why don’t want?

AFP2_0035 : It’s not much matter to me.

I_01 : Ok, ok, ok then what about like then, then ok anyway you said it doesn’t matter to you is it because you already have this I mean how long have you have this AF this irregular heartbeat you mentioned it was earlier how about how long already?

AFP2_0035 : About 2 years .

I_01 : 2 years, but before that you had any problems other, other condition or not?

AFP2_0035 : Other condition…

I_01 : Ok.

AFP2_0035 : I got some… diabetes only.

I_01 : Diabetes oh ok, ok then what about like the ok there’s some messages you know in the, the popped up message how do you find that?

AFP2_0035 : It’s ok.

I_01 : You do you how do you feel when you see the popped up message?

AFP2_0035 : Which is true also because they tell me what to eat all that, everything.

I_01 : Did you follow or not?

AFP2_0035 : Yeah.

I_01 : You follow, how come how did you follow it in you own in your own what you do?

AFP2_0035 : Which is to less whatever also I forgot already. [Participant and interviewer laughed]

I_01 : Ok, ok never mind then what about like the survey you do the survey or not?

AFP2_0035 : Survey what?

I_01 : Survey, so like they asked you some questions you managed to do the questions or not, the questions you know, they will ask you some questions?

AFP2_0035 : Oh that, oh that I never do that.

I_01 : You never do?

AFP2_0035 : I just watch.

I_01 : Ok, then what about like the blood pressure monitor like when you take you do you feel anything or not how, how do you feel?

AFP2_0035 : I feel ok, improving that’s why I’m happy with this.

I_01 : Ok.

AFP2_0035 : It’s ok.

I_01 : Ok so you, you found that it’s improving, but you got any when you when you every morning you said you or you take sometime sometimes when you used this device right and then you take your blood pressure right how do you do you have any feelings like you feel that it’s do you feel stressed?

AFP2_0035 : No.

I_01 : You feel it’s, it’s ok, you feel comfortable?

AFP2_0035 : It’s feels I feel if it the level is higher that’s worry me know…

I_01 : Worry you!

AFP2_0035 : So I just do what…

I_01 : Ok, ok…

AFP2_0035 : What to take.

I_01 : Ok, ok you feel like happy?

AFP2_0035 : Yah, I’m happy.

I_01 : Happy in terms of when you see…

AFP2_0035 : I see it…

I_01 : The results is, is good.

AFP2_0035 : I see the results is good.

I_01 : Then you feel quite happy.

AFP2_0035 : Yah.

I_01 : Ok, ok then do you find that the we are asking now about the, the, the overall the tablet ok it, it you find how do you find the screen, is it just right?

AFP2_0035 : Ok, it’s ok.

I_01 : Or too big or too small?

AFP2_0035 : It’s ok it’s…

I_01 : It’s ok.

AFP2_0035 : You know it, it looks ok what.

I_01 : It looks ok, then the, the font the size of the, the, the words?

AFP2_0035 : Readable ok.

I_01 : Readable ok, then what about like the brightness?

AFP2_0035 : Ok.

I_01 : It’s ok, then what about going, going like you know when you, you when you go into the app like you have to scroll through a few things right there are a few pages to go into right how do you find that is it how do you find the is it ok for you?

AFP2_0035 : No problem what?

I_01 : No problem ok, then like when Miss M Miss M came right to give you the device you know Miss M the, the girl…

AFP2_0035 : Yah, yah.

I_01 : Who came to give you the, the this device did she explain to you give you instructions on how to use?

AFP2_0035 : Yeah.

I_01 : How do you find the instructions?

AFP2_0035 : Instructions is ok I managed to use.

I_01 : Is it too easy or difficult?

AFP2_0035 : Easy.

I_01 : Ok, was it very too long?

AFP2_0035 : No, it was 15, 20 minutes.

I_01 : Ok, then a few more questions let’s right she, she didn’t she didn’t she come she come here she give you the device only but she didn’t explain to you how to use do you think that you still be able to use or not?

AFP2_0035 : I don’t know I never see this before…

I_01 : You never see this…

AFP2_0035 : So…

I_01 : Before, so…

AFP2_0035 : So she, she taught me what to do.

I_01 : She taught you what to do but let’s say if she did not come here right would you be able to use your on your own?

AFP2_0035 : I can’t, I don’t know. [Participant laughed]

I_01 : You can’t, you won’t be able to…

AFP2_0035 : Yeah.

I_01 : Ok, ok then what about did are you aware there is a number or not there’s a number on the… here that, that you can call?

AFP2_0035 : Yah, got a…

I_01 : So did you manage, try to call it?

AFP2_0035 : Then one day I try to call but nobody answer.

I_01 : Why when, when was that?

AFP2_0035 : That’s first May, I think.

I_01 : Oh you call for what?

AFP2_0035 : Because when I do my blood pressure it won’t register inside you know…

I_01 : Didn’t register, so you tried calling but nobody answer.

AFP2_0035 : Nobody answer.

I_01 : Ok and, and, and did you try to call again?

AFP2_0035 : I, I think around 3, 4 times only.

I_01 : You tried calling 3, 4 times ok, and nobody answered…

AFP2_0035 : No, no.

I_01 : The these 3, 4 times nobody answered?

AFP2_0035 : Nobody.

I_01 : Ok, if you are you wanted to call about the is it the…

AFP2_0035 : It, it about the…

I_01 : The, the, the result!

AFP2_0035 : The result.

I_01 : It was not recorded inside…

AFP2_0035 : No.

I_01 : Ok, so you tried calling a few times is it the same day or different day?

AFP2_0035 : The same day.

I_01 : Same day ok, ok so you did if anyway if you will you find that this number right if let’s say got people answer you will you find it useful for you or not?

AFP2_0035 : Of course, useful.

I_01 : Ok!

AFP2_0035 : That’s why worried know why, why won’t register inside!

I_01 : Ok, ok is that is that the reason why maybe you did it affect the way you take your, your I mean like you when you found out that nobody answered right did it affect the way you take your…

AFP2_0035 : Don’t know.

I_01 : Take the blood pressure?

AFP2_0035 : Maybe just partly it’s ok, I think.

I_01 : Ok, ok, ok then let’s say how do you find the study is it 6 weeks is it too short, too long or just right?

AFP2_0035 : I don’t know your… what you study I don’t know how…

I_01 : But how…

AFP2_0035 : How long you…

I_01 : How do you feel is it is it ok or not too short…

AFP2_0035 : It’s ok.

I_01 : Too long…

AFP2_0035 : This…

I_01 : Ok?

AFP2_0035 : I think it’s…

I_01 : You prefer it to be longer you prefer it to be shorter?

AFP2_0035 : I think it’s o- ok, it’s not that long.

I_01 : Ok, then let’s say if we introduce this device let’s say you first said you first diagnosed 2 years ago right let’s say we introduce this device at the beginning of your this diagnosis you prefer at the beginning we introduce it on or later… later a bit when we introduce… or…

AFP2_0035 : Later also that it’s ok, I don’t mind.

I_01 : You don’t mind right either way, then if let’s say we have a diff- really ok we have a tablet right, this is a tablet and then we also have a phone, we have a phone and we also have a desk top computer would which one will you prefer? You prefer a phone, tablet, a computer or other kind of…

AFP2_0035 : Tablet is much…

I_01 : Electronic devices?

AFP2_0035 : Eas- easier to handle.

AFP2_0035_01 : Drink, have a drink.

I_01 : Thank you very much, thank you. Tablet ok, so you prefer a tablet ok, ok ss- do you have like any other maybe like throughout this, this time right any like opinions about you know how we can improve on this, this experience that you have, and the app? Try to make it more comfortable for you or better I know you I understand that you maybe you say that you may not want to continue but like any anyway we can change your mind, you know?

AFP2_0035 : It’s ok with these 2…

I_01 : It’s ok?

AFP2_0035 : These 2, rather than so many so complicated. [Participant laughed]

I_01 : It’s complicated, oh why, how, what, what, what way is it complicated?

AFP2_0035 : Because so many gadget.

I_01 : You mean like got…

AFP2_0035 : These 2 is… more than enough.

I_01 : More than enough ok, then but you what can make you continue the study like I mean not, not , not study but continue to use these like for a long, long term you know in the future, next time? Let’s say they want to introduce it to the clinic the pat- the patients in the clinic then like what can encourage you to take this up, you know?

AFP2_0035 : I don’t know. [Participant and interviewer laughed]

I_01 : You don’t know any, any i- ideas or not how we can im- improve and make it more comfortable for you?

AFP2_0035 : It’s ok just these is enough.

I_01 : It’s enough ok, ok but when you mentioned that you, you not you don’t want to continue like…

AFP2_0035 : I don’t want. [Participant laughed]

I_01 : Why, why any reason or not… is it tiring to use?

AFP2_0035 : Not tiring but sometimes I, I forgot to think right?

I_01 : So it’s like you just like you’re afraid that you forget like that but let’s say we, we, we, we, we you don’t you don’t know have you don’t have to take everyday you can take like you know whenever you are free whenever you remember is it ok or not?

AFP2_0035 : In that case ok, like that.

I_01 : But you feel that the forgetting part it’s maybe like affects your…

AFP2_0035 : Yah.

I_01 : Your consistency?

AFP2_0035 : Yah, consis- yah it’s consistency because when I it is to take what everyday, once a day, sejuk [ means “cold” in English] so I take once a day but I don’t like to… leave a… space there.

I_01 : You don’t like to leave a space ok, ok, ok so you feel that that’s not very good ok, ok any other reason or not?

AFP2_0035 : Only that.

I_01 : Ok, ok alright, so I think any other comments about you know how we can you know feedback…

AFP2_0035 : Feedback… [Participant laughed]

I_01 : Yah.

AFP2_0035 : What feedback, [Interviewer laughed] what feedback?

I_01 : Like any feelings overall, good or bad feelings about this whole thing?

AFP2_0035 : It’s ok, I thought.

I_01 : Ok, ok, ok alright so thank you very much for, for, for you know answering the questions, I’ll stopped the recording.

End of interview
