# Supplementary material for: Patient perspectives of the Self-management and Educational Technology tool for Atrial Fibrillation (SETAF): A mixed-methods study in Singapore
Source: PLoS One. 2022 Jan 21;17(1):e0262033. doi: 10.1371/journal.pone.0262033 (PMC8782297; doi:10.1371/journal.pone.0262033)
Supplement: S1 Data — (ZIP) [file pone.0262033.s006.zip › AFP2_IDI_0031.doc]

Participant ID number : AFP2_0031

Interviewer Number : I_01

Length of interview : 15.39 mins

I_01 : Ok, thank you for taking part in the study so the first question we want to ask is for how long have you been diagnosed with AF and how did you find out about it? So how long have you been you have your this AF, irregular heartbeat then like how did you find out about it?

AFP2_0031: I think it’s good.

I_01 : Ok, how did you find out about it?

AF_0031 : How did find out?

I_01 : Yah.

AFP2_0031: Use the machine!

I_01 : But like when you first when you go to when you first have this condition so how did you like when, when, when did you like see the doctor then you know that you have this irregular…

AF_0031 : Yah, I see the doc…

I_01 : Heartbeat.

AF_0031 : I see the doctor so some somebody recommend use this one.

I_01 : Ok, but when you when, when you first have the AF the this, this, this heart this irregular heartbeat when, when were you first have it… how long ago?

AFP2_0031: Long time already…

I_01 : How long?

AF_0031 : Use, use my about 5 years.

I_01 : Ok.

AF_0031 : Use my I, I buy the machine.

I_01 : Ok, what machine did you buy?

AF_0031 : Same with this one the a, a simple one.

I_01 : Oh but, but at the beginning when you take medication, do you take any medication?

AFP2_0031: Yah.

I_01 : Ok, and this you find out where, through your doctor is it?

AF_0031 : Yah, yah find out, doctor.

I_01 : So where do you see your doctor?

AF_0031 : I think few years ago…

I_01 : Few years ago, how long ago?

AF_0031 : Few years ago he check my, my heart got some problem it’s so fast slow fast slow not, not constant so another one just high blood.

I_01 : Ok, then what other things?

AFP2_0031: Other thing is like that like this.

I_01 : Urology ok, so the, the that time when you see a doctor did he give you any advice or not, about what to do?

AF_0031 : No advice just must everyday take, take the medicine.

I_01 : Then what about like diet, exercise?

AF_0031 : Yah, yah he call must exercise and, and be careful the, the eating for the food eating the food.

I_01 : Ok, so there are, are there any other like you, you have friends, family who also help you or not?

AF_0031 : No.

I_01 : Don’t, so you mostly you do it on your own?

AFP2_0031: Yah.

I_01 : Ok, but what about like finding out information, finding out how to manage your condition?

AF_0031 : How to manage I, I also don’t know.

I_01 : You don’t know?

AF_0031 : Ah I don’t know how to man-…

I_01 : You don’t know how to manage?

AF_0031 : Ah I don’t know how to manage I just…

I_01 : What, what do you mean by…

AFP2_0031: I just normal, everyday same like that.

I_01 : But what about like you got read anything got go and find out from the Tv or like listen to radio…

AF_0031 : Sometime I, I can find out at the fb and internet.

I_01 : Fb, internet, ok!

AF_0031 : But it’s the Chinese one.

I_01 : Chinese one, so is it helpful or not to you?

AF_0031 : Yah helpful, very helpful.

I_01 : Ok so, how did you how, how did you find out, you, you, you, you read the…

AFP2_0031: So sometime I follow so cannot eat this one, eat more this one.

I_01 : Ok then what about like so during ok so let we go on to the your this study the during this 6 weeks of this study how do you use the device on a av- on every everyday how do you use it?

AF_0031 : Morning time I when I wake up I just check on the on the i.ipad and check.

I_01 : Ok.

AF_0031 : After that then I go google, before sleeping I also check like that.

I_01 : So about 2 times.

AF_0031 : Ah 2 times.

I_01 : Then like when you…

AFP2_0031: Because this one put on, on my bed near, near my bed.

I_01 : Where, near your bed.

AF_0031 : Ah, near your bed!

I_01 : So do you think it’s convenient for you or not?

AF_0031 : Yah, very convenient.

I_01 : Very convenient ok, then what about like do you have any feelings that come out when you when you…

AF_0031 : No.

I_01 : When you take your blood pressure or not?

AFP2_0031: No, no, no feeling.

I_01 : Like you feel like say happy, stress…

AF_0031 : No, just normal.

I_01 : Normal, then were there any parts that were easy for you, which one do you find the easiest to use, to do?

AF_0031 : Not easy?

I_01 : Easiest to do of all these things like say you take the blood pressure then you have to you check that the app right?

AF_0031 : Because sometime, sometime one time is very high one time very low I think it’s not ac- not so accurate put this one.

I_01 : So do you how do you feel when, when you when you see it like this?

AFP2_0031: I, I am not, not con- confident with this machine because sometime it 140 another second time it 121…

I_01 : Ok, so you find that up and down…

AF_0031 : Ah, so I don’t know take 3 time or one time.

I_01 : Ok, so you when you see it’s too high or too low, will you take again?

AF_0031 : Yah I, I almost I take again.

I_01 : Ok, then what about like ok so like you take the blood pressure right you do you think that it’s difficult or not or easy?

AF_0031 : No, no not difficult, very easy.

I_01 : Very easy, ok because already have your device is it?

AFP2_0031: Yah.

I_01 : Ok, then what about like it do you know that it will be some videos to for you to watch or not?

AF_0031 : I have no time to watch, I see inside got 4 video…

I_01 : Ok, how come you, you, you feel that you…

AF_0031 : Because I, I did that one I all those internets, I know already.

I_01 : So you know from the internet?

AF_0031 : Yah internet and fb a lot.

I_01 : Yah, but what about video do you think that let’s say because it’s give given by the doc- I mean like this is created by the hospital one right so do you think that it’s more useful or not?

AFP2_0031: I think useful.

I_01 : Then why didn’t then why you how come you didn’t watch the, the video?

AF_0031 : I got because I, I know a little because exactly which one which one about exercise we, we know I know a little bit.

I_01 : Ok, so you, you, you in the in this time period right you, you didn’t even watch you didn’t watch the video?

AF_0031 : Yah when I free have to have to watch.

I_01 : So you did you watch, [Participant laughed] you got watch or not?

AF_0031 : I watch one, one only.

I_01 : You watch one, so how do you find the, the that one?

AFP2_0031: The, the, the good!

I_01 : Which one did you watch?

AF_0031 : I think, I think the heart one.

I_01 : The heart the…

AFP2_0031: The inform-…

I_01 : The information one is it?

AF_0031 : Ah information.

I_01 : Did you finish or not?

AF_0031 : Yah, the rest I not…

I_01 : You didn’t…

AFP2_0031: I didn’t.

I_01 : Ok, ok so how do you how do you find the information, is it is it helpful, useful?

AF_0031 : Yah helpful, helpful.

I_01 : Helpful in, in what way did you change your behavior or not when you…

AF_0031 : Yah, a bit change.

I_01 : You, you changed?

AF_0031 : Yah.

I_01 : Ok, how, how did you change your behavior?

AFP2_0031: Oh just take care the food and exercise.

I_01 : Ok, ok, ok then what…

AF_0031 : And must everyday eat the nutrition.

I_01 : Ok, so you think, think it’s helpful in that way…

AF_0031 : Helpful…

I_01 : Ok, then what about like the, the popped up message you know there are those popped up message is that…

AF_0031 : Popped up message very simple men, every everyday is the same.

I_01 : How do you find it, do you have any feelings when, when it came up?

AFP2_0031: No, no feeling it’s all normal, normal, normal everyday is normal all, all continue normal, continue.

I_01 : Ok, do you think that it, it, it gives you encouragement or not?

AF_0031 : Yah.

I_01 : It does!

AF_0031 : Yah.

I_01 : Ok, ok in what way it encourage you?

AFP2_0031: Yah!

I_01 : How, how, how does it encourage you?

AF_0031 : Because it’s so see the result so good, confident.

I_01 : Ok, confident you feel did you feel happier?

AFP2_0031: Yah, happy.

I_01 : Happier, but then what if it’s too high what if let’s say your they say that your I mean you said that some of your reading was too high?

AF_0031 : Oh, once a time now everyday certainly one time I very high, 141.

I_01 : Then how, how do you how do you react [Participant laughed] how do you feel?

AF_0031 : To… I normal I act scared a little bit but night time it’s ok already go 121.

I_01 : So, go back down, ok.

AF_0031 : Go back, night time 121 or 111.

I_01 : But let’s say if it’s like when you take if let’s say the reading is high right how, how often was it very high?

AFP2_0031: About 3 or 4 time.

I_01 : 3 or 4 times ok then, then this 3 or 4 times is different day or…

AF_0031 : Different day.

I_01 : Or, or continuous?

AF_0031 : Different day.

I_01 : Different day ok, ok so, so you, you get a bit scared when you see?

AF_0031 : Maybe the machine maybe I’m not, not careful.

I_01 : Not careful, so you feel it’s the machine?

AFP2_0031: Yah.

I_01 : Ok, ok then what about like the, the survey did you do the survey or not there’re those survey questions sent to you?

AF_0031 : 5, 5, 5 survey I, I, I, I don’t know how to read.

I_01 : Don’t know don’t know how to read is it was it too because is it you prefer in Chinese?

AFP2_0031: Yah.

I_01 : Oh so was it too maybe difficult I mean not difficult, you don’t understand you don’t understand the language…

AF_0031 : Yah I don’t understand, so…

I_01 : Ok, so you prefer in Chinese?

AF_0031 : Yah.

I_01 : You hope you can have more all in Chinese ok, then what about like the how do you find the screen is it too big, small, just right?

AFP2_0031: Which are normal can, can see...

I_01 : Can see.

AF_0031 : The screen.

I_01 : Ok, let’s say we have a few different kind of device like we have phone, we have a desk top computer, we have a, a tablet this, this ipad right, you prefer which type?

AF_0031 : I prefer the phone better because…

I_01 : Phone!

AF_0031 : The phone everyday I carry.

I_01 : So you, you, you don’t mind you carry around?

AFP2_0031: Yah, yah.

I_01 : You would you find that it’s a bit like you know when you carry around your results everywhere is it like you feel more…

AF_0031 : Yah.

I_01 : Anxious?

AF_0031 : Not, not no problem I think.

I_01 : No problem ok, ok then let’s say we have a paper form would you want to do it in paper form like so like we have pap- paper that means you, you record down on, on your own using pen or pencil then you sent back to us…

AF_0031 : For what?

I_01 : Then we sent the we sent information, the brochure to you will you prefer this one or you prefer the electronic one?

AFP2_0031: I think electronic is better.

I_01 : Ok why, why you prefer electronic?

AF_0031 : Convenient.

I_01 : More confident ok, you mean convenient?

AF_0031 : Convenient ah, convenient!

I_01 : Any other reasons or not?

AF_0031 : Yah, other reason is… I think it’s convenient it’s...

I_01 : Convenient ok, ok.

AFP2_0031: Those paper always not, not convenient

I_01 : Ok, ok then how do you find the font the, the size of the word?

AF_0031 : This word?

I_01 : Yah.

AF_0031 : Ok.

I_01 : Ok?

AF_0031 : Yes.

I_01 : Can see?

AFP2_0031: Ah can see.

I_01 : Then the, the brightness?

AF_0031 : Brightness also can.

I_01 : Ok, then the do you find that when you use the app you go to the like you s- you scroll through the different screen is it easy or difficult?

AF_0031 : Easy, easy.

I_01 : Ok, ok then like ok so Miss M she went down to give you the, the device right Miss M…

AF_0031 : Yah.

I_01 : She passed you the device, she gave you any instructions or not?

AFP2_0031: No, I just say you, you, you explain to me…

I_01 : Did s-…

AF_0031 : I forgot already.

I_01 : You forgot ok, will you find that it’s helpful or not her explain…

AF_0031 : Yah.

I_01 : Explain, she explained in English or Chinese?

AF_0031 : English.

I_01 : Ok, you understand or not?

AFP2_0031: Understand [Incomprehensible segment] I, I not, not so good, English.

I_01 : Ok, ok so not you prefer in Chinese?

AF_0031 : Yah.

I_01 : Ok, ok then what about like you know there is a number on the on the this you know there’s a number here right did you did you were you aware of the number here?

AF_0031 : No not, not what to…

I_01 : This number here?

AF_0031 : For what?

I_01 : Yah so you did they did she ex- Miss M explain to you this number?

AFP2_0031: No.

I_01 : What is it for or not?

AF_0031 : No…

I_01 : Ok, you so…

AF_0031 : I just I just show me how to use the machine, put the, the, the key how to key.

I_01 : How to press?

AF_0031 : Ah how to press.

I_01 : Then the this she didn’t explain to you the number?

AFP2_0031: No, 6, 7 something.

I_01 : No ok, so you, you not you were not aware that there was a number that you can call to the medical team?

AF_0031 : No.

I_01 : And there this there’s this, this number right you can allow you to call the medical team if you have any problems, you were you aware?

AFP2_0031: No, I didn’t...

I_01 : Not aware, oh!

AF_0031 : Not aware and never use.

I_01 : Never use, do you feel that if there is a number is it helpful or not?

AF_0031 : Yah I think it helpful, really.

I_01 : Ok, ok, ok but you were not aware?

AF_0031 : No, I’m not aware.

I_01 : Ok, ok let’s say if you use you know you take your blood pressure everyday right so you have all these result…

AFP2_0031: Yah.

I_01 : So you when you go and did you see a doctor after that then you bring the result to go and…

AFP2_0031: No.

I_01 : Tell your doctor or not?

AF_0031 : No.

I_01 : Never, didn’t use…

AF_0031 : Never.

I_01 : Do you think it will be helpful to have this kind of track you can track your, your result then you let’s say you go and see a doctor right you can you can pass it to your doctor is it…

AF_0031 : Because I see the doctor out outside he will check this one got the record already.

I_01 : So, so…

AF_0031 : The nurse will check this one.

I_01 : Ok, so but, but they because when they when they come and when you go out to the clinic they take your blood pressure is one time only right but when you have all these you, you can show you have you take over many long period of time…

AF_0031 : Yah ev- everything.

I_01 : So you feel that is it better or not?

AFP2_0031: Better.

I_01 : Ok why, why is it better?

AF_0031 : Because sometime is not something wrong with I don’t know.

I_01 : Ok, ok then what about like how did ok this, this period of time this 6 weeks do you think it’s too long or not or too short how, how do you find it the length, the study ?

AF_0031 : I, I can said I can say it’s take a long more, yah.

I_01 : It is what?

AF_0031 : Too fast or too… I think maybe a 3 month betters.

I_01 : 3 month better ok, ok then what about like if let’s say we introduce this device ok so let’s say we introduce, introduce this device when you first find out that you have the irregular heartbeat you prefer at that point of time or you prefer later, in the middle or may maybe much later?

AFP2_0031: I actually I, I just try only I don’t know I prefer first time.

I_01 : First time that means that when you find out ok why, why you prefer like first time when you find out?

AF_0031 : Because I al- I, I, I also the heart got, got some problem now, got the connect to the my heart problem…

I_01 : Ok.

AF_0031 : Because high blood and not regular…

I_01 : So you mean like it will also help your other condition?

AF_0031 : Yah, yah can help.

I_01 : Ok, ok so like ok you got any other like comments like you can that we can…

AFP2_0031: No.

I_01 : Improve…

AF_0031 : No comment.

I_01 : Anything that you feel that you want to see in this kind of electronic device?

AF_0031 : Very convenient to use.

I_01 : Very convenient ok, but you, you mentioned that you said that you not very confident because the results you know sometimes high sometimes low…

AF_0031 : Yah I..

I_01 : What so if let’s say if it’s like that will you feel that you will still use it or not, this kind of device?

AFP2_0031: I, I think also can use because sometimes maybe the, the battery not enough or tighten not, not so not so well.

I_01 : Ok but will you think that like if it like if it’s high it’s your own your own health and your own condition or not?

AF_0031 : Yah I…

I_01 : Maybe it’s you…

AF_0031 : Maybe....

I_01 : Maybe you just…

AF_0031 : Maybe sometime I eats too fat thing the meat what also it jump to 141 so I take it few time I just 3 or 4 time also 141 I, I believe that.

I_01 : Ok, ok, ok so you would you would you would f- find that it’s still overall it’s still it’s still useful for you?

AFP2_0031: Yah, yah.

I_01 : Ok, would you recommend it to say any, an- your friends let’s say if you got any friends who got this kind of condition will you think that you’ll recommend it?

AF_0031 : Yah, recommend.

I_01 : Ok, ok, ok so I think that’s about it, thank you for, for…

AF_0031 : Ok, ok.

I_01 : For the interview.

End of interview
