# Supplementary material for: Patient perspectives of the Self-management and Educational Technology tool for Atrial Fibrillation (SETAF): A mixed-methods study in Singapore
Source: PLoS One. 2022 Jan 21;17(1):e0262033. doi: 10.1371/journal.pone.0262033 (PMC8782297; doi:10.1371/journal.pone.0262033)
Supplement: S1 Data — (ZIP) [file pone.0262033.s006.zip › AFP2_IDI_0030.doc]

Participant ID number : AFP2_0030

Interviewer Number : I_01

Length of interview : 27.33 mins

I_01 : Ok so thank you very much for taking part in the study so the first question is we’re going to ask is for how long have you been diagnosed with the irregular heartbeat how and how did you find out about it?

AFP2_0030: How long?

I_01 : How long, when was it first time, diagnosed?

AF_0030 : I don’t know, with this, this thing?

I_01 : Yah this, this...

AF_0030 : When 18…

I_01 : Irregular heartbeat.

AF_0030 : 18 May or what?

I_01 : No not, not this tablet as in like your own irregular heartbeat…

AF_0030 : Heartbeat…

I_01 : When, when did you have it, found first time found out?

AFP2_0030 : At least 2 years ago.

I_01 : 2 years ago, you found out.

AF_0030 : Yah, because I stay in and out of hospital N.

I_01 : Ok so where, where did you find out… about…

AF_0030 : 2 years I mean like…

I_01 : Where?

AF_0030 : 15, 2015.

I_01 : 2015 ok.

AF_0030 : Yah end of the…

I_01 : You, you found out when, where in hospital N?

AFP2_0030: Hospital N because no, at home I feel cannot breathe or what everything so I go there they tell me this…

I_01 : They tell you this ok, ok then did they…

AF_0030 : They tell me this, that was in 2015.

I_01 : 2015 ok, did you take any medication did they prescribed you any medication?

AF_0030 : There… a lot!

I_01 : A lot?

AF_0030 : A lot, about…

I_01 : Like what kind of medication?

AF_0030 : I, I don’t know the name but about 5 or 6 type…

I_01 : For what?

AFP2_0030: There’s for heart one and for the…

I_01 : Hello.

AF_0030 : Urine everything.

I_01 : Urine everything!

AF_0030 : Yah…

I_01 : But…

AF_0030 : And the warfarin…

I_01 : Warfarin ok.

AF_0030 : Warfarin, direct take.

I_01 : Ok then other than the hospital right did they where did you get your, your sources of information to manage your, your, your condition outside of the hospital?

AFP2_0030 : Outside the hos- hospital…

I_01 : Don’t?

AF_0030 : I, I go from this poly from poly sent me there.

I_01 : Ok, ok so…

AF_0030 : I go first I go poly then I refer…

AF_0030_01 : Refers first.

AF_0030 : Refer clinic…

AF_0030_01 : Clinic, you know x-ray you…

AF_0030 : X-ray, then I go to poly then they said they don’t have the medicine in this clinic all so you have to refer to hospital N.

I_01 : Hospital N, ok so then they got prescribed any diet for you or not?

AF_0030 : Diet, a lot!

I_01 : Exercise?

AF_0030 : A lot of thing I cannot eat, all good, good thing I cannot eat.

AF_0030_01 : Now when you when you take warfarin so many things you cannot eat…

I_01 : Cannot eat right, yah.

AFP2_0030: Ah cannot eat!

AF_0030_01 : No, the green things you cannot eat too many then when you now…

AF_0030 : Yah, the organ of the…

AF_0030_01 : Eat less and then the blood, it become thin and then thin I don’t know… [AF_0030_01 laughed]

I_01 : [Interviewer laughed] So…

AF_0030 : Then have to sleep, but still you know when I sleep at night I cannot sleep like that what, I must sleep sideways otherwise I cannot breathe you know…

I_01 : Really?

AF_0030 : Still!

I_01 : Still have it?

AF_0030 : Still have it, I cannot sleep like that I cannot because…

AF_0030_01 : Cannot sleep straight away, have to bend like that or either this side or the other side.

I_01 : So normally you all cook or you all go out to eat?

AF_0030_01 : Cooked myself.

AF_0030 : Most of the time cook at home.

I_01 : Cooked at home ok, so you all have to control also.

AFP2_0030 : Control yah if outside we don’t know what oil what oil they use.

AF_0030_01 : Especially they’re oily and then the organ all he cannot eat and then ve- vege is the green thing is not everyday have to take [AF_0030_01 and interviewer laughed] then…

AF_0030_01 : Some fruit also I cannot eat.

I_01 : Fruits also cannot?

AF_0030 : Fruits also got, got…

AF_0030_01 : No, actually is the, the got the…

AF_0030 : Some of the…

I_01 : Some of it, ok.

AF_0030_01 : Not, means got apa [Means “what” in English] like green tea…

I_01 : Green tea…

AF_0030 : That one all…

AF_0030_01 : Cannot take cannot drink no, no…

AF_0030 : Malay, Malay herb…

I_01 : Cannot?

AF_0030 : Herb all cannot.

AF_0030_01 : Herbs all everything herbs he cannot take.

AF_0030 : I cannot take.

AF_0030_01 : No, no, no, no.

I_01 : Ok, ok so like you know how do you like manage all these like you know you cannot eat this you cannot eat that right…

AF_0030_01 : Have to what.

AF_0030 : Yah, I control…

I_01 : [Interviewer laughed] Control?

AFP2_0030: What if what the doctor…

AF_0030_01 : He cannot he…

AF_0030 : Tell me…

AF_0030_01 : He cannot eat, he eat but I have to control him what?

I_01 : So you help, you help out also.

AF_0030_01 : Of course, of course. [Interviewer laughed]

AF_0030 : If he want me to early go… [Interviewer laughed]

AF_0030_01 : Early…

AF_0030 : Then he cook anything!

AF_0030_01 : No, no! It’s not, not, not for me for him you know if he’s he will still want to eat that one I not suffer, he what…

I_01 : Ok, so…

AF_0030_01 : When I want, want every time go hospital I don’t I don’t like…

I_01 : You don’t want also right yah, yah.

AF_0030_01 : That’s why I have to control the, the makan. [Means “ food “ in English]

I_01 : Yah, all these makan [Means “food” in English] thing you all find out from the, the hospital is it?

AF_0030 : Hospital, he said…

AF_0030_01 : Follow!

AF_0030 : These thing cannot eat, this one cannot eat, so I follow.

AF_0030_01 : Have to follow.

I_01 : So they are the one who tell you all one, ok.

AF_0030 : The hospital.

AF_0030_01 : Yah, always the doctor must tell…

I_01 : Doctor!

AF_0030_01 : Then sometimes the warfarin also said what when we read check the blood…

I_01 : Yah, the blood.

AFP2_0030_01: If no good and then sometime, sometime…

AF_0030 : Sometime high…

AF_0030_01 : The warfarin is must to be…

AF_0030 : Ok normal but sometime very low…

AF_0030_01 : 2 point 2 point over.

I_01 : Very low ok, so like oth- other than the hospital outside do you all like find out information yourself like Tv, newspaper, reading, internet…

AF_0030_01 : Sometime we read… paper…

I_01 : Paper!

AF_0030_01 : Sometime I heard from the radio…

AF_0030 : I see…

I_01 : Radio…

AF_0030_01 : Radio!

I_01 : Radio!

AF_0030 : Paper or…

AF_0030_01 : Mostly radio.

I_01 : Mostly radio…

AF_0030_01 : We always support radio.

I_01 : Ok, ok mostly radio ok, ok then what about your other relative your relatives do they help your, your…

AF_0030 : My relative…

I_01 : Yah your relative like you know…

AF_0030_01 : Help what, what you mean?

I_01 : Yours…

AF_0030 : Help…

I_01 : Meaning like your if you have any like relative or friend, relative meaning that your family…

AFP2_0030: Yah…

I_01 : Your other family do they help you?

AF_0030_01 : Of course yah, yah.

AF_0030 : Have!

I_01 : How, how do they help how would they help?

AF_0030 : Yah, have… see what type of..

AF_0030_01 : What, what, what kind of help you, you, you mean?

I_01 : Help meaning like you know give you advice talk to you…

AF_0030 : Yah…

AF_0030_01 : Of course, my son…

I_01 : You [Interviewer laughed] give you information?

AF_0030_01 : My son always advice him, my son my second one…

AF_0030 : Smoke don’t too much but I still smoke what.

I_01 : You still smoke ok, you still smoke?

AF_0030_01 : Smoke but less…

I_01 : Less smoke.

AFP2_0030_01: And then you have the son must go and exercise he said have to go walk…

I_01 : You all…

AF_0030_01 : Exercise, my son always advice him.

I_01 : Ok, very good very good, did you all go together you all go…

AF_0030_01 : No.

I_01 : And exercise…

AF_0030 : Sometime I go…

AF_0030_01 : Last time they always walk together.

AF_0030 : And walk with him.

I_01 : Sometimes you go and walk, you walk with him?

AF_0030 : With him…

AF_0030_01 : No, my son won’t go…

AF_0030 : With the family…

AF_0030_01 : He’s in the army what he know what, what to do.

I_01 : What to do ok, ok.

AF_0030_01 : So he’ll advice the father…

I_01 : Ok, ok so…

AF_0030_01 : Have to walk and, and sometimes he said you go and cycling the…

I_01 : Cycling!

AF_0030 : Exercise, cycling you do your leg…

AF_0030 : Buy for me a lot of bicycle I cannot...

I_01 : Cannot why cannot?

AF_0030_01 : The leg the leg…

AF_0030 : The leg…

AF_0030_01 : One of the leg, cannot.

I_01 : Ok, what happened to your leg?

AF_0030 : I cannot go, that one.

I_01 : Ok, ok.

AF_0030_01 : Because he got an accident last time.

AFP2_0030 : Last time.

I_01 : Ok, ok so limited movement.

AF_0030 : Ah that’s why.

AF_0030_01 : When he got an operation blood clogged, you see now.

I_01 : Ok, ok.

AF_0030 : Here you see lobang [Means “hole” in English] here.

I_01 : Oh ok, ok there was an accident that time.

AF_0030_01 : There, before 2, 2… 0…

AF_0030 : One!

AF_0030_01 : Two zero two.

I_01 : Two zero 2 ok, ok then during this the 6 weeks of the study what do you normally do on a typic- average day what did you do to, to the blood pressure and the app right what you do…

AF_0030 : What I do?

I_01 : What did you do first what’s the sequence?

AFP2_0030 : No, just check and then open the check and see…

AF_0030_01 : Sometime the blood sometime the up…

AF_0030 : Sometime low sometime ok he said normal, like that.

I_01 : Ok, when did you take it more, what time of the day?

AF_0030 : I take sometimes…

AF_0030_01 : Sometimes he…

AF_0030 : 11 to one o’clock like that.

I_01 : Ok, around…

AF_0030 : That time, most of the time is that is the time.

I_01 : Around that time.

AF_0030_01 : 11 something, most of the time.

I_01 : Ok, ok.

AF_0030 : To one o’clock.

I_01 : Ok, ok most of the time ok, ok then like do you have any feelings when you take, take the your blood pressure do you feel anything like…

AF_0030 : No…

I_01 : How do you feel how do you feel?

AFP2_0030 : I feel ok, just take sometime I see very low you know not very good, I changed hand [All laughed] I changed to the… then I…

I_01 : Ok, oh you changed your hand, changed…

AF_0030 : I changed, once inside 80 something, ok.

AF_0030_01 : Once itu cakap suruh makan apa [Means “He said what to eat” in English] protein bukan [Means “not” in English] protein, bread apa roti… [Means “what bread” in English]

AF_0030 : Wholemeal…

AF_0030_01 : Meal apa [Means “what” in English] meal don’t scared they put name must eat the bread…

AF_0030 : Bread, wholemeal bread!

I_01 : Bread, wholemeal bread.

AF_0030_01 : Good for him you see to keep the blood…

AF_0030 : Mostly I eat all, all wholemeal one.

I_01 : Did you watch the video in the, the tablet app or not the video?

AF_0030_01 : No.

AF_0030 : Sometime I see…

AF_0030_01 : Sometime we see.

AF_0030 : Sometime I open…

I_01 : Sometimes you opened.

AF_0030 : Open and see…

AF_0030_01 : No, not everytime.

AF_0030 : I see all the things…

I_01 : You see for how long?

AFP2_0030 : Just see about 10 minute I see…

I_01 : 10 minute?

AF_0030 : 5 or 10 minute I see, like that.

I_01 : Ok, so you watch which one which video did you watch it got a few video right?

AF_0030 : I see, yah.

AF_0030_01 : Tengok aku punya? [Means “Watch mine?” in English]

AF_0030 : Yang tengok orang punya [Means “Watch theirs” in English] got people down there… [AF_0030_01 and interviewer laughed]

AF_0030_01 : I never touch…

AF_0030 : So many…

AF_0030_01 : I don’t want to touch…

AF_0030 : Show everything…

AF_0030_01 : So that anything you cannot blame…

AF_0030 : Here, these thing what, what…

I_01 : Is for him only, yah.

AF_0030_01 : Only for him.

I_01 : Only for him, yah so…

AF_0030_01 : That’s why I don’t want to touch.

I_01 : Any favorite video favorite video?

AF_0030 : Video, nothing because I don’t like to see because I got…

I_01 : Why?

AF_0030 : This type of sickness I don’t want to see this type…

I_01 : You don’t want to see you, you so you feel that…

AFP2_0030: See little bit only I see ok, little bit study what is this, what is that ok.

I_01 : Is it because you, you feel a bit scared is it?

AF_0030 : Yah.

I_01 : Is it?

AF_0030 : That’s why I don’t want to see.

AF_0030_01 : Not scared.

I_01 : Not scared…

AF_0030 : But…

I_01 : Then, then why don’t want to see?

AF_0030 : Huh?

I_01 : Then why don’t want to see?

AF_0030_01 : Act- actually have to see?

I_01 : I mean we, we want to know see but…

AFP2_0030 : Yah, you want to see what the figure down there you put I only see what is this what in the stomach what here, what…

I_01 : The information one…

AF_0030 : Information…

I_01 : Information video…

AF_0030 : That one I see but what happen what sometime I don’t see.

I_01 : You don’t want to see all those things, ok then what about the, the, the exercise one and the cooking one did you watch?

AF_0030_01 : Cooking…

AF_0030 : Cooking one…

I_01 : The cooking one?

AF_0030_01 : [Incomprehensible segment] No, no we never see.

AF_0030 : Cooking one, never see.

I_01 : Never [Interviewer laughed] ok, then what about exercise, the exercise one?

AF_0030 : Exercise I have seen, one or 2 time I see only.

I_01 : Do you think that it’ll it’s helpful or not?

AFP2_0030 : Helpful, but exercise I myself also very lazy to do, I go down also got exercise I also don’t want to do.

I_01 : Oh…

AF_0030 : Because the leg here…

I_01 : Difficult.

AF_0030 : Difficult!

AF_0030_01 : Actually I tell him if, if he do everyday you know I think it can become loose…

I_01 : Can…

AF_0030_01 : Loose already it’s ok but once he start cannot he choose to stop also.

AF_0030 : Very painful, want to start you know some more…

AF_0030_01 : That’s why so long you never do I said must do slowly the son also said [Interviewer laughed] must do slowly, slowly after that you can become better, he don’t want what I don’t know.

AF_0030 : But I feel ok, see I can walk…

AF_0030_01 : He can walk fast…

I_01 : Can walk!

AF_0030_01 : Then 3 bus stop can walk.

AF_0030 : 3, sometime morning, [Interviewer laughed] but morning I do regular one…

AF_0030_01 : Because he can go…

AF_0030 : One whole round.

AF_0030_01 : Shopping…

I_01 : Oh really?

AF_0030_01 : Window shopping round, round, round can what?

I_01 : Oh ok, ok. [Interviewer laughed]

AF_0030 : Morning, 7 thirty I go until 8 o’clock I come back one whole round here.

AF_0030_01 : They go N then they walk and then 8, 8 something they have go out.

I_01 : Oh ok, so like shopping all these you can walk.

AF_0030 : Yah can!

AF_0030_01 : Shopping can what [Interviewer laughed] window shopping inside the supermarket all go round un- one, 2 hours can what.

I_01 : So let’s say we, we have like more interesting video for you to watch you think it will change, encourage you or not the video if we add more video do you think that…

AFP2_0030 : Encourage…

I_01 : It will have a make a difference?

AF_0030_01 : He is very lazy one, you don’t ask him.

AF_0030 : Because…

I_01 : No, but we want to find out you know…

AF_0030 : I, I know…

I_01 : Whether it helps?

AF_0030 : Because of my age now, I feel nothing to do these hard work.

I_01 : Ok, ok. [AF_0030_01 laughed]

AF_0030 : Because I’m 70 over already…

I_01 : Yah, I know.

AFP2_0030 : That’s why I don’t think good to do all the hard…

AF_0030_01 : Want to relax only. [Interviewer and AF_0030_01 laughed]

AF_0030 : Ah just relax…

I_01 : Just relax…

AF_0030 : Mostly of the time, yah.

I_01 : But video, if we think a video can help you or not like if we have people in the video right let’s say like there are people who also exercise in the video you know you think will encourage you?

AF_0030_01 : Exercise!

AF_0030 : Yah exercise, but I don’t know…

I_01 : Maybe, maybe simple one…

AF_0030 : Whether I can make it, yah?

I_01 : Simple one simple exercise.

AF_0030_01 : If you the, the…

AF_0030 : But…

AF_0030_01 : Video you give the story like good, bad, ugly like, like old, old stories he like to see.

AF_0030 : Movie! [AF_0030_01 laughed]

I_01 : Like movie type video like story line ok, ok interesting.

AF_0030_01 : Apa [Means “What” in English] this type one you ask him see… [Interviewer and AF_0030_01 laughed]

I_01 : Ok, then did you do the survey or not there’s some survey that, that come out right?

AF_0030 : Yes, survey come out.

I_01 : Did you manage to do it?

AFP2_0030 : Yah, it asked me question isn’t it, then I… [AF_0030_01 and interviewer laughed]

I_01 : How do you find it?

AF_0030 : I find ok, it asked me ok how are you feel today [AF_0030_01 laughed] I said ok good if I feel good…

AF_0030_01 : If suppose he said no good?

AF_0030 : Ah no good ah, some question he asked this I said I don’t know I, I put don’t know.

I_01 : Don’t know, ok.

AF_0030 : Ah just pressed there.

I_01 : The survey do you find that how did you find the, the length of it, is it too long, too short…

AF_0030 : No, ok what…

I_01 : Ok!

AF_0030 : Sometime he got short 2, 3…

I_01 : Yah correct.

AFP2_0030 : Sometime 10… [AF_0030_01 laughed]

I_01 : Yah, yah so you…

AF_0030 : Ok.

I_01 : Ok with it?

AF_0030 : That’s ok, I make until finished.

I_01 : Ok did you feel that it’s too difficult, the survey?

AF_0030 : No, no not really…

I_01 : So not…

AF_0030 : Nothing, that one is ok what.

I_01 : Ok, ok it’s alright ok then what about like the popped up message you know these popped up message encouraging messages diet advice all that you know did, did you manage to see it or not?

AF_0030_01 : Kau tak buka boleh? [Means “You did not open it right?” in English]

I_01 : Like those, those encouraging messages…

AF_0030 : Message?

I_01 : Like whenever you take your blood pressure…

AFP2_0030 : Like message, yah.

I_01 : It will popped up “well done” that kind of thing.

AF_0030 : Yah, that one that one I o- open yes that message only, I did.

I_01 : Does it help you do you how do you feel?

AF_0030 : That’s why when I see…

AF_0030_01 : Stop apa [Means “what” in English] stop smoking, [AF_0030_01 laughed] they will say know?

AF_0030 : Sometime put “stop smoking”.

AF_0030_01 : Stop smoking uncle. [AF_0030_01 laughed]

AF_0030 : He can… why he saw me or what? [Interviewer and AF_0030_01 laughed]

AF_0030_01 : How…

AF_0030 : That’s why…

AF_0030_01 : How they know?

I_01 : That one is part of the because we they have your profile also.

AF_0030_01 : Yah, yah profile stop smoking like that.

AFP2_0030 : That’s why I stopped smoking.

I_01 : So how do you feel do you that it helps or not?

AF_0030 : Help lah, like that I feel good…

AF_0030_01 : It’s good…

I_01 : It’s good

AF_0030_01 : It’s good, yah.

I_01 : Helped!

AF_0030 : Because she know how did he know today I smoke a lot, more you know I can feel it what I thought “wah” this fellow also know. [Interviewer and participant laughed]

AF_0030_01 : He said that he go out like that like, like that… [Interviewer laughed]

I_01 : So you think that like this popped up message you, you do feel you…

AF_0030 : Yah…

AF_0030_01 : Can help some, some…

AF_0030 : Helped.

I_01 : Helped a bit.

AF_0030_01 : Helped a bit.

I_01 : Ok, good.

AF_0030 : The reading all.

I_01 : Ok, do you would you would you think that you’ll be more motivated to do things, like if let’s say if we have more popped up message right encourage you do you think you’ll be more motivated?

AFP2_0030 : Yah, but exercise I cannot already I like to lie [AF_0030_01 laughed] I take medicine sleep, sleep here one half hour I have to sleep…

I_01 : Sleep…

AF_0030_01 : Like become compulsory already…

I_01 : Oh really?

AF_0030_01 : Take medicine only, must rest.

AF_0030 : Morning I take my medicine 9 thirty, I have rest here.

I_01 : Ok, but…

AF_0030_01 : He did not sleep just rest like that.

AF_0030 : Rest only, cannot…

I_01 : But…

AF_0030 : That’s why I cannot work.

I_01 : Ok, ok you feel sleepy?

AF_0030 : No, I take medicine I feel tired you know?

AF_0030_01 : No actually, actually not feel sleepy you he…

I_01 : Habit?

AF_0030_01 : He…

AF_0030 : Just must, must…

AF_0030_01 : He made all the compulsory you want? [Interviewer laughed]

AFP2_0030: Must rest!

AF_0030_01 : Compulsory, don’t say habit.

I_01 : So is there anything that you find that is your favorite you know like, like you or you like in the in the this program you know the blood pressure monitor and the tablet, anything you like?

AFP2_0030 : Yah…

I_01 : How you feel?

AF_0030 : To check the that the blood pressure is good…

AF_0030_01 : It’s good…

I_01 : It’s good.

AF_0030_01 : Everyday teaching…

AFP2_0030: Everyday check good I know…

AF_0030_01 : Whether you good or no good, it’s better.

AFP2_0030: Normal or bad, if low also you put low normal also you put normal…

AF_0030_01 : So you know your blood how…

I_01 : You think it’s useful to know?

AF_0030 : Yah you to know but , but I have my own one you know this…

AF_0030_01 : But the, the lady said cannot use must use this one…

I_01 : Yah because it’s…

AF_0030_01 : Because this one to go hospital N…

I_01 : Correct, correct...

AF_0030_01 : That way…

I_01 : So do you think…

AF_0030_01 : And then told me you auntie cannot use I know I know got I don’t know I never use one what.

I_01 : [Interviewer laughed] So do you think that it this type of connection back to hospital N will help?

AFP2_0030: That’s good what, I think you know…

I_01 : It’s, is it better than the one that you already using?

AFP2_0030: Yah this one…

AF_0030_01 : That one is for your own.

AF_0030 : Only for myself only at home…

I_01 : For your own, for your own.

AF_0030 : This one direct isn’t it?

I_01 : Yah, yah.

AFP2_0030 : Down there also they know.

AF_0030_01 : For a while that, that this one go to direct and then they know.

I_01 : Direct, they know yah, they know so you do you, you know there is a number over there on the tablet…

AF_0030 : Yes, yah.

I_01 : Did you call or not, the number?

AF_0030 : I never call…

I_01 : Never call...

AFP2_0030 : Nothing happen what, so no need to call. [Interviewer laughed]

AF_0030_01 : They, they, they put there in, in case if, if anything happen you can call…

AFP2_0030: Ah if anything…

AF_0030_01 : Can call.

I_01 : Do you think the number if let’s say if it we have a number there it’s helpful or not?

AF_0030 : Yes…

AF_0030_01 : The better you, you can call.

AFP2_0030 : Ok better, easy for us if anything problem I also can call them direct.

I_01 : Ok, ok so Miss M you know Miss M she came the, the woman right she came and…

AF_0030_01 : The woman ah the, the ladies…

I_01 : Gave you the… yah, she gave any instruction that, that time?

AF_0030_01 : No just…

I_01 : When she came?

AF_0030_01 : She just tell the, the num- the key number…

AF_0030 : Key in the number…

AF_0030_01 : After she said after…

AFP2_0030 : Pin number…

I_01 : Yah pin number…

AF_0030_01 : Anything happen just like that like this, ok.

I_01 : It’s ok.

AFP2_0030 : Yah anything happen you go and call this number.

I_01 : Ok, ok let’s say if she came that she passed you the device but did not give you the instruction you think you can use on your own?

AF_0030_01 : No.

AF_0030 : We don’t know what how want to use? [Interviewer laughed]

AF_0030_01 : The key number all don’t know what?

AFP2_0030 : We also don’t know, ah the pin number press the pin number then only you can use.

I_01 : Yah but she gave you the pin number…

AF_0030 : Yes.

I_01 : Let’s say you go into the, the tablet you think you can use on your own or not the, the app?

AF_0030 : The app.

I_01 : The app?

AF_0030_01 : Complicate all complicate?

I_01 : Yah, can or not?

AF_0030 : Can!

I_01 : Can?

AFP2_0030 : I use like…

I_01 : On your own?

AF_0030 : On my own?

I_01 : Without instruction?

AF_0030 : Yes, that one I know…

I_01 : You know!

AF_0030 : Because earliest she tell…

AF_0030_01 : Can read what can we open the book like that what [Interviewer and AF_0030_01 laughed] but never because the lady teach him already.

AFP2_0030: Teach me and said don’t but like…

AF_0030_01 : After that just off, like this…

AFP2_0030: Don’t off the thing he said…

AF_0030_01 : Ah, after that off.

AFP2_0030: The tablet he said but keep on running [Interviewer laughed] then the battery always must flat what then above 50 you must do…

I_01 : Charge!

AF_0030_01 : Charging, charge.

AF_0030 : Low than 50 you cannot, that’s why.

I_01 : So you all you all go and charge it…

AFP2_0030 : Charge lah.

AF_0030_01 : Yah, charge what! [Interviewer and AF_0030_01 laughed]

I_01 : Is it very troublesome for you or is it ok?

AF_0030 : Ok, like…

AF_0030_01 : More troublesome lah…

AFP2_0030 : 2 days one time I charged full ah 2 days I no need to charge…

I_01 : Oh ok, ok.

AFP2_0030: Because this thing never off one what keep on going sure flat lah, ah like, like that.

I_01 : Ok, ok, ok how do you find the, the screen size is it ok the screen size is it…

AF_0030 : The triangle that one?

I_01 : Like the screen size like you know the… this… is it too small or too big or ok?

AF_0030 : Ok, this one is ok.

AF_0030_01 : Ok just, just like before what.

I_01 : Ok, then the brightness the brightness ok?

AF_0030 : Ok, everything ok.

I_01 : Ok, then the, the font the, the size of the letter…

AFP2_0030 : Letter all, all ok.

I_01 : Can see or not?

AF_0030 : All can see.

I_01 : Can ok, then the what about like the, the move the app you know like you want to go in and out of the app, scroll through the app…

AF_0030 : That one…

AF_0030_01 : Boleh buka boleh buka apa kau tahu apa macam gimana? [Means “Can you open it do you know how?” in English]

I_01 : Meaning go you know the app right the you have to go in to see the video, see your result…

AF_0030_01 : Yah, he know already.

I_01 : Go out you know, is that ok or not?

AF_0030 : Sometime ok.

I_01 : Ok not, can do right?

AF_0030 : I know all, where to press that one, the lady teach me.

I_01 : She taught you already.

AF_0030_01 : Yah, yah everything he teach.

I_01 : So everything ok, ok, ok then let’s say right we have a tablet and then we have a compu- desk top computer, we have a phone we have other kind of electronic device which kind of device will you prefer what type will you prefer so we want to ask right, you…

AF_0030_01 : Well the whole idea prefer like this, easier…

AFP2_0030 : Like this easier one take number…

AF_0030_01 : If the computer one we, we cannot.

AFP2_0030 : Can see!

I_01 : So you prefer like that…

AF_0030 : Like that easier.

I_01 : Tablet it’s ok, what about computer?

AF_0030 : Computer is too big…

AF_0030_01 : Don’t know how to use.

AFP2_0030: I don’t know how to use. [Interviewer laughed]

AF_0030_01 : Don’t know to use.

I_01 : What about phone, phone?

AF_0030_01 : And phone also…

I_01 : Hand phone, can?

AF_0030 : Phone also…

I_01 : So…

AFP2_0030 : I don’t know how to use…

I_01 : The cause…

AFP2_0030 : Because I use hand phone I never send message or what…

AF_0030_01 : We never use no, no.

I_01 : Ok, ok.

AF_0030 : All, I just people call me, I take.

I_01 : Ok, ok so you don’t use the other features?

AFP2_0030 : Those…

AF_0030_01 : No, no, no.

AFP2_0030 : If see the what, you tube ok I see for want to sleep I put the you tube I see or… that one it’s ok.

I_01 : Ok, ok so we if we have the electronic form right and paper form, paper meaning ok you, you record the blood pressure you sent to us and then we sent you some information…

AF_0030 : Yes…

I_01 : For you like brochure form, would you prefer the paper form or the electronic form?

AF_0030 : Electronic form what, what type of electronic?

I_01 : Electronic means this the tab- either a tablet…

AF_0030_01 : Tab-

AF_0030 : Tablet?

I_01 : Can be phone can be can be which one will you prefer?

AF_0030 : I prefer this one.

AF_0030_01 : It’s easier.

I_01 : Ok, ok why you prefer this one?

AFP2_0030 : This one easy just put and let it go see you can because you can check what.

I_01 : Ok.

AF_0030_01 : Yah, tapi [Means “but” in English] your current is ok [Interviewer laughed] kalau [Means “if” in English] paper you have the current kalau ini [Means “if this you” in English] about the current...

I_01 : Yah.

AF_0030 : Yah…

AF_0030_01 : Your electricity, you have to charge.

I_01 : Yah, yah but you’re ok with it you prefer the, the electronic one?

AF_0030 : Yah like this, ok.

I_01 : Ok, then ok so let’s say we introduce this program like the very first time when you found out you have the AF the irregular heartbeat right? You prefer that we introduce this program at the begin at the beginning when you first find out that you have the irregular heartbeat or later a bit or later I mean?

AF_0030 : No, this one I know I have got we the regular heartbeat?

I_01 : The, the irregular you know you just we this is to measure you’re the blood pressure right but it’s because of the your irregular heartbeat right? So we, we introduce if this, this program if we introduce it when you first find out when you had irregular heartbeat, you prefer we do that or we do it or we introduce it later?

AF_0030 : That one…

I_01 : Which will…

AF_0030 : You mean later then?

I_01 : Later then we, we introduce it or, or when at beginning when you when you find out?

AF_0030 : When we find out in the beginning like this ok, so when you finished then you checked, checked there ok the thing is there already, easy.

I_01 : Yah, you know I mean right beginning, beginning mean like ok 2, 2015 you found out you have the AF right?

AFP2_0030 : Yes.

I_01 : Then like you prefer let’s say we introduce this to more patients right, at the beginning in 2015 when you find out right we introduce it when you find out or introduce it later a bit, later?

AF_0030_01 : Later…

AFP2_0030 : Later…

AF_0030_01 : Better!

I_01 : What, what do you think? [Interviewer laughed]

AF_0030_01 : Awak lah. [Means “You” in English]

AF_0030 : Yah, later is better.

I_01 : Why, why better why better?

AF_0030 : Yah, so you can know what much more about the heart.

I_01 : Ok, but at the beginning also you can find out what?

AF_0030_01 : Still, still the same what!

AF_0030 : Still same, but at the beginning I also not, not very I only kena [Means “got” in English] this type of sick about 2, 3 month you know in and out of hospital, go in 5 days go out, stay one week again go in I think 3 or 4 time I go in.

I_01 : So that was the beginning?

AF_0030 : The beginning and the, the no good one tomorrow is my Hari Raya [Means “Malay New Year” in English] you know yah, tomorrow is my Hari Raya [Means “Malay New year” in English] I eat the what…

AF_0030_01 : Organ.

AFP2_0030: The organ or what the doctor said cannot eat, I eat that one at night I already like half dead already then I go in 5 days…

I_01 : Go back in, again.

AFP2_0030 : 5 days, Hari Raya [Means “Malay New year” in English] in the hospital.

I_01 : Ok, so let’s say that we have we introduce this program it might be a bit difficult, is it?

AF_0030 : Yah.

I_01 : Because you go in and out.

AF_0030 : In and out, last time but after that I discharged the last one is 2015 after that I, I never go in already.

I_01 : Never go in.

AF_0030 : I got regular check up and the medicine they give is the doctor give is very good one…

I_01 : Very good lah!

AF_0030 : Because last time he gave me day only day and I take at night I, I feel the pain so he gave me day and night one ok, then it’s ok the doctor is ok.

I_01 : Ok, ok, ok then any other suggestions on how we can improve the this program you know what, what you want to see you know let’s say we in- we next time we implement it for more permanent one right, what would you like…

AF_0030_01 : Have a drink?

I_01 : Thank you very much thank you, terima kasih. [Means “thank you” in English] [Interviewer laughed]

AF_0030_01 : You’re welcome.

I_01 : What, what you prefer like what you want to see that we can add on to it you know or maybe take away what, what do you hope to see in this kind of program?

AFP2_0030 : What we want to see?

I_01 : Yah, like something that you, you think you that, that you…

AF_0030 : I’m just what you ask me to do then I check and check only, like that only?

I_01 : Let’s say we do very long so it now, now it’s only 6 weeks right…

AFP2_0030: 6 week…

I_01 : Let’s say we do like a few years or one year do you think is it will you do it or not it’s like…

AF_0030 : One year?

I_01 : Ah one year. [Interviewer laughed]

AF_0030 : I don’t know.

I_01 : A longer term, we do very long term one you think you will you will be able to do it?

AF_0030 : That one I don’t know.

I_01 : Don’t know, don’t know you…

AFP2_0030 : Because very hard because I also…

I_01 : Ok, so you just a few more questions, did you sub- manage to go and see a doctor in, in this time period of the this time period the 6 weeks of the study…

AF_0030 : Yes.

I_01 : Did you go and see a doctor in the mean in the meantime and then bring the result to, to show your doctor or not?

AF_0030 : These result?

I_01 : Ah these result the you know they have…

AFP2_0030: I never bring what?

I_01 : The graph right, the graph yah.

AF_0030 : Last, last week last week I just go what…

I_01 : Oh, you got bring or not?

AF_0030 : I got the I never bring because the woman said…

AF_0030_01 : Hari Raya [Means “Malay New year” in English] you cannot come…

AFP2_0030 : Don’t bring…

I_01 : Oh terima kasih, [Means “Thank you” in English] very much, [AF_0030_01 laughed] very nice, thank you very much.

AF_0030 : Because the, the…

AF_0030_01 : Have a drink first have a drink first.

AFP2_0030 : Lady said you don’t have to bring this anywhere, just leave it here.

I_01 : Ok.

AF_0030 : That’s why I go my son house Saturday or Sunday I sleep then I never bring what that’s why?

I_01 : Ok, ok, ok…

AFP2_0030: I can’t…

I_01 : So when you go out you don’t you don’t…

AF_0030 : Don’t bring, that’s why I miss one or 2 time before meal…

I_01 : Ok, ok.

AF_0030 : So I go the doctor last week I go all ECG everything.

I_01 : But you didn’t bring?

AF_0030 : I never bring because…

I_01 : You…

AFP2_0030 : She told me not to bring out, I test her I want to go to my son house this Friday, can I bring, no need no need…

I_01 : Who, who tell you not to bring out, the Miss M?

AF_0030 : The woman…

I_01 : The woman who came, oh is it?

AF_0030 : Ah said don’t bring anywhere, just leave it here.

I_01 : Ok, ok but oh ok, ok then a few just maybe one more question… what did you is, is there anything like any other comments about opin- thought opinion about like you know how we can like you know… about your own experience about your experience? What would you like what would you want to… is there something electronic that can that we can introduce in your in, in your with your condition like to improve your life?

AF_0030 : No, I don’t know this one just to check only isn’t it?

I_01 : To check only, yah.

AF_0030 : Not said medicine or what nothing what just to check and then I don’t know who receive the this but…

I_01 : Yah, the…

AFP2_0030 : What they do also don’t know?

I_01 : You don’t know?

AF_0030 : I don’t know what I just do like that, they said don’t…

I_01 : The woman didn’t tell you?

AF_0030 : Ah he said I don’t what just he said ok you just make this one then don’t off this thing, ok.

I_01 : Ok, ok…

AF_0030 : I also don’t know what happen.

I_01 : Ok, but let’s say if you we tell you that it’s the doctor who know who, who, who see it or the nurse would you feel that better or not?

AF_0030 : Yah, if he can tell us what, what ok but we also don’t know what…

I_01 : You don’t know...

AFP2_0030 : We just check put what like the high blood or what 112 or 110…

I_01 : Yah ok…

AF_0030 : Sometime low, sometime normal very good like that just put there only, at the tab.

I_01 : Ok, ok so I think yah that’s about it, thank you very much for the…

AF_0030 : Ok.

I_01 : For letting us know, I’ll stopped the recording.

End of interview
