# Supplementary material for: Patient perspectives of the Self-management and Educational Technology tool for Atrial Fibrillation (SETAF): A mixed-methods study in Singapore
Source: PLoS One. 2022 Jan 21;17(1):e0262033. doi: 10.1371/journal.pone.0262033 (PMC8782297; doi:10.1371/journal.pone.0262033)
Supplement: S1 Data — (ZIP) [file pone.0262033.s006.zip › AFP2_IDI_0029.doc]

Participant ID number : AF_IDI_0029

Interviewer Number : I_01

Length of interview : 32.51mins

I_01 : Ok thank you for taking part in this study so first question is how long have you been diagnosed with atrial fibrillation and how long how did you find out about it?

AF_0029 : Actually I, I can’t remember you know unless I go and check my medical record I think…

I_01 : Around what time when?

AF_0029 : 4 years...

I_01 : 4 years…

AF_0029 : Or 6 years I can’t remember there was once I admitted A and E because what did I feel maybe I feel giddy or what or, or I vomited after taking, taking some steamboat maybe the clam or what raw one I, I think I can recalled 2007…

I_01 : 2007.

AF_0029 : So the next day I wanted to go to school “N” and teach then halfway I feel very giddy and couldn’t stand it I, I revert back then went to the my family doctor then I was chou jing [Means “cramps” in English] what you called that?

I_01 : That means…

AF_0029 : Like got fits like that…

I_01 : So excited like you mean overly active?

AF_0029 : Yah then like cramp like that the whole body then I, I cannot breathe then the family doctor quickly called ambulance it’s near here my MD they called “J” clinic but she’s an MD so they fetched me to the A and E then from there they found out I had this atrial fibrillation…

I_01 : Ok subsequently they yah…

AF_0029 : 2007…

I_01 : Did you go for like any like follow up you could…

AF_0029 : Yah, yah ever since then, then they, they need to do a angiogram so they found out that one of my ves- vessel clogged 40 percent cholesterol clog clogged 40 percent and then they wanted me to take this one aspirin…

I_01 : Yah aspirin.

AF_0029 : And don’t know what then the rest of my life but then after that they find that aspirin is not powerful enough so they switched to warfarin and in fact that was a second A and E when I was discharged then they said I have to take warfarin that was on a Friday then Saturday they don’t opened hospital “N” the pharmacist. So they called me talked over the phone for about one hour telling me that you know take vitamin “K” cannot take Chinese herbs cannot take green tea blah, blah, blah a lot of thing. This one until I get groggy you know then on the same night I entered A and E again…

I_01 : Oh that was being…

AF_0029 : Yah because I, I had palpitation then I get worried because first time I encountered these kind of thing how come so many thing cannot take like chrysanthemum, bean curd all these you know green vegetables all these cannot take. And then all the health supplementary also cannot take all the Chinese herbs cannot touch, touch hence at every now and then I do take a bit of Chinese herbs boiled soup so now I have to totally cut off. And then the other thing is when you take warfarin make sure you don’t get cut you cut your blood cannot stopped you see.

I_01 : Yup that’s right.

AF_0029 : So this is what I worried you know what happened you caught accident and all these and people is not aware that you are on warfarin and then they also reminded me whenever I go to doctor dentist have to tell them I’m on warfarin just before anything so they need to record down.

I_01 : So did that affect your like say your how you managed your condition?

AF_0029 : Yah that’s why I become a bit cautious then for the past few year like last year I tend to had a fall a few time 3, 4 times within a month then you looked at my scars cannot heal you know…

I_01 : At home or…

AF_0029 : Outside…

I_01 : Outside!

AF_0029 : Like the walker center actually there’s no obstacles then walked suddenly you know I fell down then here cut big, big hole like that then the next day I went to my sister’s church it’s also outside a walk walker center also walked half way the [Participant laughed] then it’s the same place [Participant laughed] that’s why you see the scar so big.

I_01 : The same kind of fall basically.

AF_0029 : Yah, yah, yah then I thought maybe my kneecap weak or what and then vision everything ok because I was told if you have frequent fall might be a sense of like stroke or what you know.

I_01 : I mean not necessarily but so you have to also check with the, the…

AF_0029 : Yah so, so and I find that you see I have a lot of scar I walked here only cut a bit then bleeding knocked there a bit bleeding then…

I_01 : Bleeding partly because of the warfarin you are taking now is it?

AF_0029 : Yah because of the warfarin so it very hard to heal so you see a lot of…

I_01 : Correct, correct.

AF_0029 : Small, small scar here and there you see my 2 leg then sometimes also here see so many months…

I_01 : A lot it happens then how long it heals take sometime…

AF_0029 : No not even heals you see if I can see the scar you see I can see the scar here already 5, 6 months still there is it because of the warfarin…

I_01 : Ok I mean the scar will definitely be there because like every injury will have scars but…

AF_0029 : I thought some will disappeared.

I_01 : It won’t disappeared completely one…

AF_0029 : It won’t…

I_01 : I mean for a lot of scars I think it’s not bleeding already yah.

AF_0029 : It’s just a small cut you know it, it just a small cut only just knocked only you bleed.

I_01 : Ok so anyway you know all these scars that you get and also like you know the because of the warfarin that you take right how do you like the information where do you get it from to try to manage all these things other, other than the doctor?

AF_0029 : Hospital “N” no it’s all they gave me a booklet will tell me what you suppose to eat and what you not suppose to eat and then they will put like cauliflower is the green one is got high, high vitamin K actually whatever high vita- high vitamin K will clash with the warfarin. So have to avoid but then they also say that you don’t have to cut off completely but you have if you want to eat that you have to eat it consistently like every week if you eat 6 leaves of greenery vegetable make sure every week 6 leaves. You cannot said one week whole you eat a lot of greenery…

I_01 : Green means vegetables?

AF_0029 : Like green vegetable then the next week all white vegetable then very hard for them to monitor your blood ratio so my blood ratio at the beginning was every 3 day got to have you know one tube of test then at first was quite unstable after that is weekly then fortnightly, monthly. But now mine is quite stable every 2 months then I go back and have the testing the tube one but then if this one more than 3 my ratio is within 2 to 3 then it’s ok if more than 3 then I have to take the one tube one then have to wait there for 2 hour to see the results so this is a bit troublesome. Then I was told that lately they have a new medicine which is not subsidy so it’s quite expensive 3 dollars a tablet but for the rest of my life is quite a burden because right now I take the warfarin is only few cents. But then that one the advantage is you take that you don’t have to go and test your blood and then you can eat freely don’t have to avoid what Chinese herbs and all that the difference is there.

I_01 : Ok do you find that the I mean did I the cost matters ok, do your I mean are you do you find that you are open to this to this?

AF_0029 : I, I wanted to change to that but then because of the cost then I have to consider because this is the rest of your life then now I already getting lesser and lesser income so I worried I may not be able to afford to buy that kind medicine. Then the, the specialist doctor said you are well on these safalo and warfarin why you want to change and also in between there was once I just want to proof that certain Chinese herbs it really helps to clean the blood. So there was once that I ate black fungus which is quite a big amount continuous for 2 days then the 3^rd^ day I went for blood checked my blood become very thin then the doctor got a shock how come your blood so thin what did you eat? So I said I presumed I ate that one so I asked him so can I consistently eat the black fungus then I don’t take excuse me…

I_01 : Then what did the doctor say?

AF_0029 : I don’t take warfarin then he said cannot because we don’t know the content of the, the black fungus so…

I_01 : So…

AF_0029 : I find that taking a lot of medicine is no good right now I’m taking about 7, 8 medicine a day beside the heart one I also have depression and then I have osteoporosis so a lot of medicine.

I_01 : Ok, ok so did do your like you have any like friends around you or like family that can help you also in terms of those…

AF_0029 : Financially?

I_01 : Not financially but in terms of knowledge you know information knowledge?

AF_0029 : Yah in fact they are quite againsing me of taking warfarin…

I_01 : Oh really because of…

AF_0029 : Because a lot of side effect you see once, once you have fall once you have cut the blood cannot stopped you know and some even said that warfarin is a poison.

I_01 : I mean it is actually a rat poison it’s, it’s they used it for like to for rats I think I mean but not it’s different I think different weight different formula…

AF_0029 : This is what they say.

I_01 : Yah that’s what they say.

AF_0029 : But then I asked them what you want me to do if I stopped can then you taking a risk of getting stroke right?

I_01 : Yah correct.

AF_0029 : Because AF like now my cholesterol is already normal know I also take cholesterol medicine but then I asked doctor since my cholesterol is normal why can’t I stopped I scared to take 20mg which is high already then he said your case is different because you got AF. Once if you stopped the, the se- sevastarin then your, your cholesterol may come back again then you may have a risk of getting stroke so I follow.

I_01 : Ok, ok then also like any other sources of information like maybe like news, media you know do you go and find yourself or not?

AF_0029 : Sometime I go to internet but not very frequent don’t have then sometime I watched those medical program so more or less can get some knowledge like channel 8 or what then…

I_01 : How, how often?

AF_0029 : Once a week but sometime I missed they that program is once a week one or that medical so they get some specialist to talk about not, not only on AF they also talk about cancer all that about high blood, diabetes. And yesterday I feel very strange why they sent me a, a letter about I may have a risk of getting the diabetes…

I_01 : Is it the hospital “N” sent you?

AF_0029 : Yah, yah why, do I have the symptom?

I_01 : Do you base, base on your results maybe?

AF_0029 : I have no I, I checked my, my result they said very good I don’t have diabetes so they sent me a brochure…

I_01 : Just a brochure, I think for info only.

AF_0029 : For info only.

I_01 : For info only yah, sometimes they do that to kind of you know screen you…

AF_0029 : I thought only sent to those…

I_01 : No you know they will send like…

AF_0029 : May have a risk of getting…

I_01 : Reminders you know kind of reminders brochures because yah if they no this is not this is not it’s mine yah.

AF_0029 : Sorry, sorry…

I_01 : Yah don’t worry.

AF_0029 : Maybe in the room… the brochures is are you at the risk of getting diabetes?

I_01 : Yah it’s just a I think a information brochures is I just put this out inside ok so anyway during this time period of this 6 weeks of this study what was the daily routine that you did like I, I mean how how’s the sequence of, of you know taking the blood pressure then monitoring yourself?

AF_0029 : Every morning I do it before breakfast I was told cannot do it after breakfast so maybe 7, 8 o’clock and then if that day I need to go out like every Monday and Thursday I’ve got I have to go to “C” hub do exercise so it’s a bit rush so I didn’t do so usually I come back home. Sometime I come back home quite late, late afternoon or at night then I almost forgotten then when I remember I might do it quite late at night I want to make sure I do it once a day. Then sometime also not sure today did I do it so [Participant laughed] I may repeated also I thought I didn’t do then I do one more time.

I_01 : Oh ok so you but you know you can do more than one time right it’s, it’s, it’s ok one doesn’t mat- doesn’t really matter one…

AF_0029 : Yah, it’s ok.

I_01 : Yah but anyway right do you find that you was it a bit troublesome for you or like or it was ok for you, you know when you when you have to do this everyday?

AF_0029 : No it’s ok with me only worried that let’s said if I go travel go tour then I may not be able to do for that one 2 days.

I_01 : Yah ok so when only when, when traveling ok so like was there any of these which one did you found was the easiest to do?

AF_0029 : Easiest?

I_01 : Yah the easiest like of all the features like which one was do you think oh, the strapping was the easiest ok then the is there anything…

AF_0029 : Because some of some of that when they asked the questions some I’m not sure how to answer…

I_01 : Oh ok.

AF_0029 : Yah, washing the foot path take your shoe in.

I_01 : Thanks, thank you. Ok, ok then like yah you mentioned about some was you didn’t know how to do is it?

AF_0029 : No, no…

I_01 : Or you didn’t understand?

AF_0029 : I don’t quite understand the…

I_01 : Yah what about that?

AF_0029 : Question, there was once a lot of question one…

I_01 : The survey is it?

AF_0029 : The survey then…

I_01 : Ok so what about it too difficult too the language was too like…

AF_0029 : The, the language was a bit…

I_01 : Not clear?

AF_0029 : Misqual I, I don’t really quite understand…

I_01 : You don’t understand ok.

AF_0029 : Maybe because I’m Chinese ed…

I_01 : But, but I mean it you ok so it’s ok was it too don’t understand in what sense?

AF_0029 : The sentence because ok do you have one example to…

I_01 : But actually…

AF_0029 : You don’t have?

I_01 : Yah it’s here never mind it’s just that...

AF_0029 : They have a, a few questions I remembered there was one survey they have a few question then maybe…

I_01 : No this…

AF_0029 : I narrowed down to like they question one like whether you, you everyday had that kind of mood or, or some how you have that mood or maybe unlikely or what there was one part I a bit difficult to answer…

I_01 : Ok, ok, ok then what about like the popped up messages do you know that they are like…

AF_0029 : Video clip?

I_01 : Yah encouraging, video clip is one also how do you find the video?

AF_0029 : Need time to digest about the pumping all these the blood how you work…

I_01 : Yah so do you watch any of the any of them is your favorite, the video?

AF_0029 : So far only watched 2 or 3 then I didn’t really pay attention on that.

I_01 : Oh which one you watch which one did you watch?

AF_0029 : The, the blood don’t know…

I_01 : The, the info one right?

AF_0029 : So that maybe you got not enough strength you have to pumped more that’s why caused your blood heart, heart beat fast and slow is it?

I_01 : How do you find it was it helpful for you?

AF_0029 : In a way it’s helpful for me to understand why we have this atrial fibrillation.

I_01 : Ok, ok then what about the you said that you watched another video 2 videos right what was the other one?

AF_0029 : I, I can’t remember because I, I didn’t really pay attention to that video because sometime a bit busy and I find it I want to quickly finished it but then once I clicked that one they need to showed the video clip then you have no choice to watch definitely.

I_01 : Do you find that the video was the, the duration how do you find it?

AF_0029 : Duration I cannot complained because you need that length then you can explained the whole thing I cannot say it’s too long right?

I_01 : Can you can say if you want to if you if you think you…

AF_0029 : Then if not long enough how to explain to the patient?

I_01 : If we can cut it in smaller pieces the…

AF_0029 : Still can understand ?

I_01 : Can, can like cut, cut the video into shorter…

AF_0029 : Shorter.

I_01 : But I mean how was your experience?

AF_0029 : I mean if shorter would be better for us.

I_01 : Ok, ok so you prefer that ok then was it in like was it interactive I mean how do you find the, the like did…

AF_0029 : There’s no interaction what.

I_01 : No interaction but in the sense that do you feel like it’s you connect with, with the video or not?

AF_0029 : Yah.

I_01 : Ok, those ok, ok…

AF_0029 : Have a look, yah.

I_01 : Then what about like the popped up messages?

AF_0029 : Popped up messages yah they always tell the result then I know I’m in the safe zone or right…

I_01 : Yah correct so was that helpful?

AF_0029 : I’m doing well, yah because we need to know…

I_01 : Were there…

AF_0029 : But sometime they warned me one you know they said you have to take more don’t know what, what food maybe that day the blood pressure not too good.

I_01 : I mean it really yah depends on the level…

AF_0029 : I see also not bad one the blood pressure but they once a blue moon they do alert you.

I_01 : The idea is to, to, to also to manage so, so these messages are meant to manage helped you to remind yourself and all that.

AF_0029 : Maybe I have to cut down deep fried food all that oily food but not very frequent once in a while then they came out with these…

I_01 : Then the…

AF_0029 : Mostly are all ok one.

I_01 : Ok were there any emotions you felt when, when it came out all these popped up mes- messages?

AF_0029 : Of course when they popped up a bit worried then when I read then I think it’s ok just to tell me that maybe you cut down your sugar or what I mean it’s quite ok it’s not that difficult.

I_01 : You get like…

AF_0029 : The advice…

I_01 : Do you feel like I mean like stressed or, or you feel better you know?

AF_0029 : No once I see of course I feel a bit stressed but I read carefully then I relieves.

I_01 : Ok, ok then what about like the blood pressure monitor right when you, you take everyday almost everyday right how did you find did were there any feelings that came about when you when you take your when you see the results of what you take so do you have any feelings you know?

AF_0029 : I feel comfortable because…

I_01 : Comfortable.

AF_0029 : I always maintained 80 or 80 and below only there was once or twice goes up then I a bit worried but then I read the answer sheet you’re still ok then…

I_01 : Oh ok so it’s, it’s alright for you ok then…

AF_0029 : So far I haven’t encounter some very serious…

I_01 : Where you…

AF_0029 : Very high pressure but then I should get worried so far never.

I_01 : Ok and let’s say if you see like maybe one day if you see like one high reading how will you feel?

AF_0029 : Of course I feel worried then I have to call, call that number you see.

I_01 : So you are aware there’s a number right?

AF_0029 : Yah on the tablet.

I_01 : Did you try calling it?

AF_0029 : Never because I got no problem so never called.

I_01 : Ok do you think the number will be useful in these kind of study and these kind of app is it?

AF_0029 : Is there a 24 hours service?

I_01 : It’s not necessary…

AF_0029 : No.

I_01 : It’s not really it’s…

AF_0029 : What happened if the patient at night then feel uncomfortable then how?

I_01 : If you feel uncomfortable you can try I mean the you can contact us the following day you see.

AF_0029 : A and E, no it’s too late already let’s say if you have…

I_01 : So you, you wished to see something more like 24 hours it’s really much more helpful.

AF_0029 : Because AF is sudden one quite serious one how can you wait until next day my only worry is like initial year that few year I always have palpitation at night one know about 10, 11 then very fast so I was a bit worry whether I should go A and E or whether I should keep calm and, and you know? And I also don’t want to waste the manpower and all these a bit only you go A and E then I did asked the doctor then he said unless you really feel very uncomfortable then you call him you see so I asked him do I take this medicine or take that medicine to, to stop and slow down he, he said no. So the answer is also like if you really cannot handle it then you go A and E but I, I told him the symptom is only the palpitation maybe don’t have giddiness then sometime the doctor said oh maybe it’s at night very quiet that’s why hear your heart beat very fast. But actually it’s not know not psychologically I hear heartbeat very fast I can feel it’s very fast but the past one year is quite stable I they give me the sopalo so now quite calm but doesn’t…

I_01 : So it when Miss “M” came to give you the device you know she explained to you some more instructions right?

AF_0029 : How to use yah…

I_01 : Do you think that was it useful for you the ex- the instructions the explanation let’s if you didn’t have all these instructions do you think that you will be able to use it on your own not, not really, why?

AF_0029 : Because first time I, I encountered this machine…

I_01 : Ok so…

AF_0029 : How do I know how to use?

I_01 : So not very familiar.

AF_0029 : But, but certain things she also doesn’t able to answer me just like just now I asked you that one…

I_01 : Ok, ok because we are…

AF_0029 : 80 upon…

I_01 : We are not medically trained I mean as you…

AF_0029 : So she also cannot answer me.

I_01 : Ok and it’s better that actually you consult your doctor for that because they…

AF_0029 : No, no, no what I mean is you all can always asked the doctor and give us a, a…

I_01 : A gauge…

AF_0029 : Stable…

I_01 : A range?

AF_0029 : Stable range of the ratio so at least we know what is high what is mediocre what is low and…

I_01 : Ok, ok yah ok anyway the you were you find that was too was it easy or difficult when she gave you the instructions was ok? Ok, ok! Ok then what about like how do feel about the overall duration of this study was it too short too long, average, 6 weeks?

AF_0029 : I, I cannot said too short because you need 6 weeks to get the result like what I was told even if you got heart attack or heart problem you go to A and E they immediately they do the ECG at that moment you don’t have the symptom also they won’t be able to detect isn’t it? So this one if I do it everyday at home I think is better you, you will know your blood pressure everyday is on what range otherwise I wait for like monthly or bi-monthly then go there and checked then after that then I know.

I_01 : Ok, ok so you, you don’t mind that…

AF_0029 : It’s useful.

I_01 : It’s useful the daily taking of your truck…

AF_0029 : You see at the end every patient had one.

I_01 : You, you mean the track the results?

AF_0029 : This one, no I mean it in future the whole Singapore whoever has the AF…

I_01 : They hope to…

AF_0029 : Then you introduced this machine.

I_01 : Yah actually they hope to implement this on a wider scale if it works for people if it works for like patients like you and also like you know you are kind of like piloting this study and you’re doing it, it as a trial for us. So if it works for you and for everyone a lot of people and that’s the kind of response that we get then we will implement it on a wider, wider scale because you know as you know that the hospital setting is the they are having a bit of over capacity overload actually not, not over capacity…

AF_0029 : Overload.

I_01 : Yah overload not over capacity yah so anyway right let’s say right if this we have a option of a tablet at a, a phone, computer and other kind of electronic devices which one will you prefer to use?

AF_0029 : Tablet, computer and…

I_01 : Phone?

AF_0029 : Phone…

I_01 : That means like on your phone the app is on your phone, mobile phone right or any other kind of devices electronic device which one will you prefer which form?

AF_0029 : Tablet, tablet…

I_01 : You prefer tablet yah ok.

AF_0029 : Because it’s bigger.

I_01 : It’s bigger ok but what about like desk top computer desk top computer is very big also?

AF_0029 : Yah but then every time you got to go there because my laptop is at the other room then I have to take the trouble to go there or if this small one let’s say if one day I need to bring out also quite easy.

I_01 : Ok so you are ok with this size ok.

AF_0029 : This sizes yes.

I_01 : Ok, ok then what about like ok we talk a little bit about the interface of the tablet that means like the how do you find the font was it too…

AF_0029 : Yah big enough.

I_01 : It’s ok, ok then the screen size? It’s ok, then the brightness? Also ok, then…

AF_0029 : Yah that one can adjust one.

I_01 : Ok then what about navigating do you find it a bit difficult…

AF_0029 : Navigating…

I_01 : Yah navigating the app that means going from…

AF_0029 : Navigating means what?

I_01 : Navigating means like you go to watch the video then go out then go back to tracking your, your blood pressure…

AF_0029 : Just tapped only…

I_01 : Your but…

AF_0029 : See which column you...

I_01 : Was it ok for you?

AF_0029 : So far ok.

I_01 : Ok, ok then…

AF_0029 : Unless you want me to go and search myself then I find a bit difficult.

I_01 : Search yourself what, what do you mean by that?

AF_0029 : Like maybe you want me to, to go to the internet and search for…

I_01 : Is it?

AF_0029 : A certain thing and get the answer or what then it’s…

I_01 : So you prefer more, more information into the tablet more, more, more like…

AF_0029 : Just like one click only I can know what question I need to ask and then they would be able to answer me…

I_01 : Like but let…

AF_0029 : I’m sure you all have a lot of question rea-…

I_01 : Correct yah.

AF_0029 : Ready for patient.

I_01 : But let’s say if there is too much info will you find that it’s too like can be too overwhelming let’s say we have…

AF_0029 : Overwhelming…

I_01 : Information right we put a lot of information inside you know you…

AF_0029 : Put the important one will do…

I_01 : And…

AF_0029 : Too much also sometime we don’t have the time to do.

I_01 : Ok, ok so only the important ones ok then let’s say if we have the paper form would you be interested in it or not like, like paper…

AF_0029 : Filled in?

I_01 : Yah filled in then you, you mailed it to us you know then we will send you some brochures to for the information rather than the electronic form would you prefer the electronic form or the paper form?

AF_0029 : Maybe electronic form would be faster then you save paper save the world.

I_01 : [Interviewer laughed] Ok then so let’s say if we introduce this app and the tablet into let’ say when you, you found out you had AF a few years ago I mean 2007 that you mentioned. So like you prefer it to be introduced during that time or in the middle you know much later when you have AF already. Or maybe much later so when would be the right time for, for like if we let’s say we want to implement this on a wider scale and introduced it to people you think that when is the best time to for us to introduced it to people who have AF? When they first got diagnosed or in the middle or…

AF_0029 : First they got diagnosed as early as possible…

I_01 : Ok why, why do you say that?

AF_0029 : Mine is because it’s too late then you invented this.

I_01 : Yah it’s only recently that we have these but like, like in the let’s say for if you could let’s say get new…

AF_0029 : Get new patient.

I_01 : New patient but also like let’s say in the if you we could introduced it when you first had it…

AF_0029 : First had would be better.

I_01 : Would be better why, why do you say that?

AF_0029 : So that maybe can help him to recover faster or cannot be recovered, AF.

I_01 : So [Interviewer laughed] I mean ok so you think that it can actually help to like to actually…

AF_0029 : I mean any illnesses also it’s better that you discovered earlier and then you start treating earlier just like cancer the first stage to handle is better wait until fourth…

I_01 : Ok, ok then what about like so a few more questions you have any like comments or any kind of recommendation or you know opinion about how we can improved on this app or not you know how we can add, add on to it you know any other features that you will like to see?

AF_0029 : This one do you have the Chinese version?

I_01 : At the moment we only have English, yah.

AF_0029 : Ok I mean to me I still can understand but my friends they don’t understand English so what happen if you want them to try all these then there is a problem.

I_01 : So more language versions that catered to different language speaking people.

AF_0029 : And different age because some like, like usually for my age my classmates all they only know Mandarin…

I_01 : Mandarin ok.

AF_0029 : They are not…

I_01 : More comfortable also.

AF_0029 : Comfortable in English because my exposure maybe a bit different with them.

I_01 : So ok more language any other kind of features you like to see or not ok, ok, ok then I think that should be it ok so I’ll thank you very much first stopped the recording.

End of interview
