# Supplementary material for: Patient perspectives of the Self-management and Educational Technology tool for Atrial Fibrillation (SETAF): A mixed-methods study in Singapore
Source: PLoS One. 2022 Jan 21;17(1):e0262033. doi: 10.1371/journal.pone.0262033 (PMC8782297; doi:10.1371/journal.pone.0262033)
Supplement: S1 Data — (ZIP) [file pone.0262033.s006.zip › AFP2_IDI_0024.doc]

Participant ID number : AFP2_0024

Interviewer Number : I_01

Length of interview : 31.18 mins

I_01 : Ok so thank you very much for participating in this sur- study, the first question is what how long have you been diagnosed with AF and how did you find out about it?

AFP2_0024: How long ok, found out about it when during my first pregnancy and I think that was around 1999 no, no wait I gave birth to my, my, my son is 1990 so that my first pregnancy that time so was found out but I went to P so P notice there’s something is wrong so sent me to hospital N that’s it, from there onwards.

I_01 : Ok, so you found out through hospital N.

AF_0024 : P first, then go to hospital N.

I_01 : P first, then referred to hospital N…

AF_0024 : Hospital N, yes right.

I_01 : Ok then were there any medication that were prescribed to you at that point of time?

AF_0024 : During that time?

I_01 : Yah.

AF_0024 : Let me see… not that I can remember only go for regular check up and every time see the O.n.g. and I will see the cardiologist also that time every time but I…

I_01 : Together, concurrently.

AFP2_0024: Yes together, every time yes.

I_01 : Ok then what other advice did the doctor give you in terms of managing your AF together with your pregnancy at that point of I mean you, you were just you just have a kid right, so like what were the challenges and also what were the advice that was given to you?

AF_0024 : To make sure that only my only one and only child [Participant and interviewer laughed] don’t get pregnant again and then after that also I think he asked about my I think that time I was a housewife so shouldn’t be a problem only lesser housework don’t carry heavy things. That is about it then that time I think I was still ok I was only like what 20 years old I think.

I_01 : Ok, ok but in terms of like managing what advice was did the doctor give you…

AF_0024 : Long, long, long, long ago.

I_01 : Or the hospital give you…

AF_0024 : Long time ago.

AF_0024 : That you can think of you know at that point of time?

AF_0024 : Oh not much I can say because I think I still remember he said that well that time I was only about 20 years old so I’m still ok nothing much asking me every time will be like any breathlessness any anything. Heart, chest pain whatever so that time no I don’t have anything of that sort but well yah, yah I was ok my first pregnancy was ok, my second one also was ok only my third one that was a problem…

I_01 : Ok, ok you mean in terms of the AF, you mean you mean AF being involved like say affecting the, the…

AF_0024 : My daily thing is it?

I_01 : Yah I mean like because you said that your third pregnancy is it because of the preg- is it because of A, AF that, that complicated some issues or…

AF_0024 : I yah, my heartbeat was very, very fast always, yah that was the time also the first pregnancy my doctor said my heartbeat was like horse riding, that what he said.

I_01 : Ok, then the third one you said that what, what was the issue actually at that point of time?

AFP2_0024: The third one I think my age was like I mean I think I was 33 years old then I can feel already I mean the tiredness some sort of breathlessness also but not but I still can go to work everyday not a problem. Not that I can remember I wasn’t hospitalized or whatsoever during the third pregnancy but I was tired, tired yes correct.

I_01 : Ok, ok so in terms of your own the outside of the hospital how do you normally manage your like, like AF on your own or like you know what kind of information did you go to try to find…

AF_0024 : You talking about that one after the, the, the surgery I went for the surgery 2000, 2005…

I_01 : What surgery was that?

AF_0024 : Open heart surgery!

I_01 : So you actually went twice?

AF_0024 : No, the 2005 was the first one I went for the open heart surgery to change the valve…

I_01 : Then the other one?

AF_0024 : If it’s before that, you are talking about before that?

I_01 : Both.

AFP2_0024: Both oh, before that after I gave birth I’m talking about the third one, ok during my third pregnancy the doctor gave me this medication I cannot remember the name I think to moni- to, to lower to slower down my heartbeat I think. Like high blood pressure kind of medication that one to, to make it every time like 80, 90 you know or else it…

I_01 : Blood thinner?

AF_0024 : No, no that one haven’t yet…

I_01 : That was later.

AFP2_0024: That one is like for blood pressure to, to maintain my heartbeat to not being so fast because every time it was like maybe 120 above every time, so to maintain I think about 80 I think if I’m not mistaken that the only medication I took during the third pregnancy, so after that one year later then I go for the open heart surgery.

I_01 : Ok and, and then for you…

AF_0024 : Then I’m on medication for the blood thinner.

I_01 : Ok then on your own like let’s say like out outside of the hospital because you spent most of your time you have to work you have your family like what kind of information did you go to…

AF_0024 : Oh…

I_01 : Usually…

AF_0024 : Sometimes I…

I_01 : Or find out from…

AFP2_0024: I go right and googled [Participant laughed] just googled to find out I also see exactly how they do the surgery, all via google.

I_01 : You tube is it?

AF_0024 : You tube yah, google then go you tube yah.

I_01 : Ok then what about like other sources like in terms of your friends, family, people around you?

AF_0024 : None of my family have this so I was the only one I mean and my siblings, don’t have! But my mother have my mother have diabetes, high blood pressure yes, but I think until she passed away hers was still manageable not a problem, she passed away also not because of that.

I_01 : Ok was it a challenge I mean because you said you’re on your own and you having this condition in your family was it a challenge getting information finding things out you know learning from people…

AF_0024 : Yah, I think I never ask anybody also I think I do not know anybody who has this, like I say I only do I google I type out what I want to know…

I_01 : Yes…

AF_0024 : And then the you tube thing, that’s about it… what…

I_01 : Ok what about TV, radio, newspaper, books?

AFP2_0024: I seldom read newspaper seldom TV, so long I also never watch TV, [Participant and interviewer laughed] but books but not but not that kind of books not medical…

I_01 : Ok not health not…

AF_0024 : Not health!

I_01 : Not, not health related.

AF_0024 : Mine were if, if like if not health related but sometime usually is how to manage myself that’s about it and novels that’s what I read.

I_01 : Ok, ok so the, the information you get means mainly from the hospitals the doctors’ clinic.

AF_0024 : If anything I will ask them whenever I go for the check up but so far so far after the surgery mine my, my, my blood thinner right also touch wood, sometime ok sometime not ok sometime even I hospitalized…

I_01 : You mean after 2005 that, that surgery?

AF_0024 : Yes, so many times.

I_01 : Ok, a few times you, you went back in already?

AFP2_0024: Yes, yes even after the surgery right my blood was ok and then they gave me 3 months later for the check up, and that first check up my blood start go down zero point 9, that one immediate hospitalization. [Participant laughed]

I_01 : Really ok, ok, ok so a few times already?

AF_0024 : A few times already, then got sudden fever that was I think that was the last shot, that was the last time I went I hospitalized few years back I had a sudden fever.

I_01 : Ok, ok so like you know during this 6 weeks of the study what was how do you use the device a typical average day?

AF_0024 : You know in the in the morning before I go to work or night time when I come back from work.

I_01 : Oh ok, so you do it twice or one time?

AF_0024 : Sometimes, sometimes twice sometimes once, most of the time one time…

I_01 : Most of the time one time.

AF_0024 : Either night or morning.

I_01 : Ok then like the what do you do first were there any feelings that come about when you used tried to use the monitor or not?

AFP2_0024: Oh feeling?

I_01 : Yah, any emotions?

AF_0024 : Cool down, [Participant and interviewer laughed] not, not nervous ok, most of the time like that but I think during the course of this doing myself the, the blood pressure thing right? That was once I think suddenly is low blood pressure I was alamak [Means “oh no!” in English] this one that was in the morning I think and I was like just wake up so I saw the this the device I said better do first, wait I do something else I forgot. So I go and do and then suddenly alamak [Means “oh no!” in English] low blood pressure! [Participant laughed]

I_01 : Ok, low blood pressure so...

AF_0024 : They asking me to then I go and do something 15 minutes, do it again then after that ok. [Participant laughed]

I_01 : Oh ok so, ok so there was some message to guide you?

AF_0024 : Yah, yah correct.

I_01 : How do you find the, the messages like to guide you?

AF_0024 : Very good, I see the first video the one the picture of the valve I never thought that it, it looks like that. [Participant laughed]

I_01 : Ok, ok so you there was something new that you found out?

AFP2_0024: Yes, yes correct, correct and then asking me to do exercises but actually the exercise but I think the recommended right the, the, the, that type right, I, I’ve been doing that, some sort of it.

I_01 : Oh, you follow in some way?

AF_0024 : But before, before doing this I already like do walking…

I_01 : Ok, ok, ok, ok so you already did your own, but this video kind of also maybe en- encourage it?

AF_0024 : Yes, yes, yes correct.

I_01 : Ok, do you feel that it change you change the way you do things you know in anyway like say you were doing it in one way, then after watching the videos you do it in another way?

AF_0024 : The video as in?

I_01 : Ch- or, or the messages you know…

AF_0024 : Yes, yes.

I_01 : Affected the way you do things do you think that it does?

AFP2_0024: Yes!

I_01 : Ok, in what way does it affect, change the way you do things?

AF_0024 : Well… to me how to say the messages ok is if, if the my blood pressure was ok said like done well blah, blah, blah all that right that is like some, some sort of a motivation also but I think I, I also like how if do via this type right to because this also by the blood thinning thing right, I’m taking right?

I_01 : Yah, correct.

AF_0024 : That one is also quite stressful.

I_01 : Oh what, what aspect is stressful?

AF_0024 : No, I mean you know to, to manage my food intake that is the major problem for me.

I_01 : Ok so the food intake part you found that a bit more challenging but…

AF_0024 : Otherwise other than that my exercises or my have to, to relax myself doing whatever somewhat I, I do follow some of the like the thing all that.

I_01 : So let’s say the exercise one right I know is only one video you must still watch it and of… no sorry the, the…

AFP2_0024: I watched it.

I_01 : About the diet one right you watch did you watch it, it how do you find it?

AFP2_0024: Well ok, to me it it’s ok because I also my, my eating habits I to me I always I only eat when I’m hungry so when I’m hungry then I eat but then after because of this warfarin right so I will make sure I take some vege just to, to maintain. And then otherwise sometimes I can like one day I never eat rice the never eat anything I eat bread or, or biscuits that’s about it.

I_01 : Oh I see ok, so you, you feel would like let’s say more videos about the cooking part right you know more, more…

AF_0024 : That’s good.

I_01 : Details about it yah…

AF_0024 : That better.

I_01 : Will you think it will help you more or not?

AF_0024 : Teach me better…

I_01 : To eat…

AF_0024 : I mean I know better then you know so can eat, eat better if not I will be like that eat roti [Means “bread” in English] eat bread or biscuit… [Participant laughed]

I_01 : Or just…

AFP2_0024: And then and then sometime I said few days already I never eat rice so meaning there’s no vege then I will take rice and vege extra that’s it and then back to bread and then back to biscuit and then another meal of rice with vege that’s how I… [Participant laughed]

I_01 : Ok, ok so that’s you, you will like oca- a few I mean after a few while a, a short while of eating bread and biscuit then you take the rice and vege then it, it you think that like more videos will help you?

AF_0024 : Yah.

I_01 : Learn about cooking?

AF_0024 : Better!

I_01 : Ok, ok then let’s how do you find what is your favorite feature in the this program this app this blood pressure, is there anything that you that’s that will stood out for you…

AFP2_0024: This program…

I_01 : That you felt was useful, helpful, interesting?

AF_0024 : The videos, like I said in the, the videos of how, how the heart pumping with the valve open, closing these thing that is very, very so call open my, my eyes thinking as in like I never thought that thing is look like that. And then it was like then I try to imagine that the that valve changed the, the valve that I, I have now it’s a metal valve I think that is very, very interesting that kind of video is, is good. [Participant laughed]

I_01 : You… so if we have more of that you will you will like it?

AF_0024 : Show it… yes, yes, yes.

I_01 : Ok, ok.

AFP2_0024: Very interesting.

I_01 : Ok, how do you find the survey there, there was some survey that was being done like for you know survey questions you, you know…

AF_0024 : Oh some of the questions like really like there is no how to say it’s a question like A,B,C that one is good, bad, no good that’s it there is no in between so I said alamak [Means “oh no!” in English] mine is not so good but not so bad then how am I going to, to, to, to input right? So I just I think just bad just I, I just like that so if got like you know that there are other… other point…

I_01 : Options?

AF_0024 : Other options so I can you know really, really input how I really feel or how I really you know?

I_01 : Ok, ok so…

AF_0024 : Because it’s very standard one.

I_01 : Ok, ok how’s the length of the survey for you, you find?

AF_0024 : Ok, so, so.

I_01 : Ok, ok means the…

AFP2_0024: Ok then you are talking the…

I_01 : The, the, the length…

AF_0024 : The how many…

I_01 : The how many questions yah.

AF_0024 : How many questions, the question was ok, is not is not long.

I_01 : Ok, ok, ok it was, was it too easy, difficult, alright?

AF_0024 : There are…

I_01 : The difficulty of the questions?

AF_0024 : Oh you mean some, some of it I well… so called like agak I agak, agak [Means “I estimate or guess” in English] only because some of the questions I don’t really understand some…

I_01 : Oh, what, what do you don’t understand, what questions?

AFP2_0024: No not, not about the, the sentence it’s just some of the words I just to me right like I said I just agak, agak… [Means “estimate or guess” in English]

I_01 : What words…

AF_0024 : I think I…

I_01 : What do you mean?

AF_0024 : I can’t remember but not all of the survey is like that so I just looked at the question I mean I looked at the answer I the need the, the, the answer that I need to, to tick right I need to choose then I said ok then I can roughly understand what exactly the question is. So some of the words are really a bit a bit chim [Means “intricate” in English] so yah I, I don’t understand…

I_01 : Some of the terms?

AF_0024 : Yah correct, the terms.

I_01 : Ok, ok then what about like ok the blood pressure monitor right what when you take took your blood pressure right what was some of the feelings that came about some what are the emotions that, that come out when you take it?

AF_0024 : Feelings, you mean as in?

I_01 : The like the do you feel anything when you the blood pressure like what…

AFP2_0024: I feel happy…

I_01 : Was some of the…

AF_0024 : This one also good for me to check…

I_01 : Really?

AFP2_0024: On my own blood pressure [Participant laughed] if not I won’t be doing that thing myself unless I go for my, my check up, that’s about it. [Participant laughed]

I_01 : Ok, ok do you find that the results are helpful in anyway, like you said you’re happy right, happy with what?

AF_0024 : Yah correct, correct happy!

I_01 : Happy with?

AF_0024 : When I see it said that my blood pressure is normal keep it up whatever, of course I happy!

I_01 : Ok, ok so…

AF_0024 : Very happy!

I_01 : You, you are glad to have a good results?

AFP2_0024: Yes correct, correct.

I_01 : What if the results was not so good?

AF_0024 : Not so good… yah it stated there if not so good there is a number to call I will definitely call. [Participant and interviewer laughed]

I_01 : Did you try calling the number did you manage to try to call the number?

AF_0024 : No, because most of the time…

I_01 : Ok, how come?

AFP2_0024: All the time was good except the one that is said at a lower, low blood pressure and asking me to redo it again then after that it was ok then ok happy already, so no problem.

I_01 : Ok, do you find that the number being there is it useful, do you think that is a useful thing to have a feature to have?

AF_0024 : Number, which number?

I_01 : The, the number on the, the tablet the number that you can call right?

AF_0024 : Yah.

I_01 : Do you think that is a useful thing that to have it in the tablet…

AFP2_0024: Yah, yah of course so if…

I_01 : Or something that you prefer not to have?

AF_0024 : So if I need to call then I will talk to a person right, so that is much more better!

I_01 : Ok, ok, ok then about the beginning when Miss M came to give you the device right did she give you any instructions, Miss M she pass you the device right?

AF_0024 : Ok but that time she said do not off it, so if it’s low reached until certain percentage you quickly go and charge it, then asking ok say if you want to do the, the, the, the what’s that thing the blood pressure thing right you can you can do twice, trice up to you, as long as you, you do it ok, that’s about it.

I_01 : Ok, ok do you think that the what she told you was a bit too difficult, too long?

AF_0024 : No, ok what she came also not, not so long for a just a while explaining to explaining about that thing then understand something, everything I also like, like now I also want to go to work one, I’m a…

I_01 : Ok, let’s say if she did not come right to you and would you think that or I mean she came but she did not explain to you the give you instructions would you think that you will be able to, to use it on your own, the device?

AF_0024 : Yah, I think there’s a there’s a, a paper right also I think…

I_01 : Ok the instructions…

AFP2_0024: Some sort of instructions…

I_01 : Booklet…

AF_0024 : I think not so complicated.

I_01 : So you, you find that…

AF_0024 : Wouldn’t be a problem.

I_01 : Ok, ok, ok then would about the results you know tracking the results right did you bring it to see any doctor in the in the mean time during these time period?

AF_0024 : Doctor, as in like?

I_01 : Like you know you’ll be able to see your blood pressure like results over a period time right do you do you like you know show your doctor you know the graph or like the, the, the series of results?

AF_0024 : No, because…

I_01 : When you saw during these time period?

AFP2_0024: The last my check up was in Hospital N that time I think last month, no I didn’t bring, that one is just at home and I, I never been to GP…

I_01 : Ok, do you think that it’ll be useful to, to, to bring it to GP to let them know and you know…

AF_0024 : Good what!

I_01 : This, this time period of, of, of results rather than you take one time right…

AF_0024 : Oh yah there’s a graph then yah…

I_01 : Yah, so how you find…

AF_0024 : So let the doctor see it the, the graph that’s what you are asking?

I_01 : Yah, yah that’s right.

AF_0024 : Oh!

I_01 : Do you think that it will be something that you will do like on your own like if let’s say you have this like for longer term, would you think that you would do that or you just go to the doctor and, and just take the blood pressure like one time?

AFP2_0024: Oh ok…

I_01 : How…

AF_0024 : To me if it’s just the blood pressure then even I don’t need to bring over there also they can do the check up I mean the, the blood pressure thing but if to me whenever I do the, the, the blood pressure right and then when I received the, the notification and all that right? And stated was ok everything so I don’t think there is a need to see to bring to see the doctor unless when something wrong like I said may I can get still a call or it’s like continuous that will be a problem of course I will bring.

I_01 : So only when there’s some issue then you bring it, is it?

AF_0024 : Hmm, hmm.

I_01 : Oh I see ok, ok so how do you find feel about the overall duration of the studies these 6 weeks you think that is just ok, let’s say if we pilot this to more people do you think that you know what would be the, the ideal length of the study, that we should have?

AF_0024 : 6 weeks is, is, is not is it in 6 weeks 6 weeks also not 2 months, right?

I_01 : It’s about one and a half months.

AF_0024 : One and a half month one and a half month also good, long term also very good!

I_01 : So what, what you prefer on your own let’s, let’s say for yourself like what will you prefer?

AFP2_0024: This blood pressure thing just the, the blood pressure the thing right if I if I can afford it I will buy one I can do it myself check every time, everyday.

I_01 : Everyday!

AF_0024 : This kind of thing I think 6 month 6 weeks not it’s too, too short make it maybe…

I_01 : It’s too short? Ok.

AF_0024 : 2 months 2 months, should be ok.

I_01 : Ok, ok so like for a study 2 months will be ok, then for yourself you don’t mind using it for a much longer more longer term.

AF_0024 : Yah, yes.

I_01 : Ok, then ok so let’s say we have a few different kind of devices we have a phone, we have a tablet, we have a computer, desktop or any other kind of electronic device which one would you prefer what form would you prefer?

AF_0024 : This one is ok, cannot be desktop I my…

I_01 : Ok why, why not desktop?

AFP2_0024: My house no desktop, all is laptop and then also my work is like very hectic one my, my of- my shift work morning, night, morning, afternoon then morning again then afternoon again so my working hours irregular you see so…

I_01 : Ok some something more mobile?

AF_0024 : Yah correct, so this one is ok.

I_01 : But you don’t bring this to your work right, do you?

AF_0024 : No!

I_01 : Ok.

AF_0024 : If, if it’s stated there I need to bring of course I will bring, not a problem.

I_01 : Ok, ok so let’s say we have a, a paper form right a paper form where you write down everything then you send you mail to us or we send you some brochures for information you think that you would you prefer something like that…

AF_0024 : Ok!

I_01 : Or you prefer the electronic one?

AFP2_0024: That is not a problem also.

I_01 : Not a problem!

AF_0024 : Email thing asking me to fill up and then via email system, no problem.

I_01 : Ok, ok or you prefer like ok let’s say you have this paper form compared to the electronic form which one will you prefer?

AF_0024 : Paper?

I_01 : Yah.

AF_0024 : If paper form I can bring to work…

I_01 : Oh ok.

AF_0024 : Easy, easier lighter you know this one then I need to bring the, the pad and all and then the other ok, I think paper is better but ipad also still ok for me only just do it at home, that’s about it.

I_01 : Ok, ok so paper you actually bring it around?

AFP2_0024: I can because last time I… wait, try to remember yah my third pregnancy I also have this, need to do my sugar, kidney so that is I bring to work, so do the paper thing also so was ok, not a problem.

I_01 : Ok, ok so let’s say we introduce this device and the, the blood pressure monitor and the app to you so when do you think will be best time to introduce it to you, when you were first diagnosed or maybe later a bit or maybe at much later. So like when would be the ideal time, in the middle when you have had this AF or like later a bit or at the beginning?

AF_0024 : Oh, but even in it’s at the beginning this thing is already like this I mean my the valve thing here right so doing this so call checking of this blood pressure, will it change anything I don’t think so right? So to me I mean if just look at I mean I I’m looking from the outside thing and then anytime before or after or now I think but I think now is also much more better because I’m much more older and then I already gone through all these thing so this is to, to make sure I main- I maintain myself my health. Then you know so can be better if before I don’t think it will change I still need to go for these thing the surgery and all so I think after then is much more better…

I_01 : You mean after your surgery?

AF_0024 : Yah, after surgery like right now I mean even though it’s so long already my surgery 2005 but still now I my I’m like now I 47 years old so I this thing also I’m getting older so I need to know my health maintain my health, manage it to make sure I’m ok, if this thing right so…

I_01 : Ok, so but with what about before the surgery?

AF_0024 : For this thing?

I_01 : Yah this thing, this, this app and tablet?

AF_0024 : If before surgery of course will make me nervous!

I_01 : Will make you nervous…

AFP2_0024: Because why…

I_01 : Why?

AF_0024 : Of course my blood pressure all very fast… [Participant and interviewer laughed]

I_01 : Oh ok.

AF_0024 : My heartbeat will be like very fast I think if I looked at the, the result and then it’s like, wah lau like that then I will be like stressed, then how?

I_01 : Ok, so you think it would actually caused a bit of stress, let’s say if it’s before your surgery or just before your surgery ok, ok then let’s say ok do you have any like comments or any you know opinion about like how we can improve this app you know? The time period when you’ll be you have been using this, this app and the tablet for 6 weeks, what do you think you want to see you know that we can improve on, that we can change you know…

AFP2_0024: I don’t know…

I_01 : So that more people can benefit from this?

AF_0024 : Or maybe some things that we can I can write down I mean you know where, where whereby you are asking me about how I feel today, so like the question that is like very good, good you know, not so good you know maybe is something I can write down what I feel at that point of time. Ok I’m I feeling sad you know or something maybe can do like that.

I_01 : You mean like a kind of note pad is it?

AF_0024 : Yah, so I can write you know?

I_01 : Note pad feature within the tablet, the app?

AFP2_0024: Yes, yes.

I_01 : Ok, ok why would you like to see something like that?

AF_0024 : So I think like that it will be better for, for we all to understand our daily thinking you know and then about our health issues maybe we can write down sometime like a diary know?

I_01 : Like a diary like that ok, ok...

AF_0024 : Sometime right?

I_01 : Did you feel that if you do something like that will be like a bit of a lot of work?

AF_0024 : No, this one I can do night time come back come back before sleep then write down because that night time will be the whole day’s kind of thinking, feeling right so before sleep everything will come into your I mean the think about it I mean that is for me…

I_01 : Yah, yah, yah it’s, it’s very interesting.

AF_0024 : I think about it so can right penned down whatever I feel at that day during on that day and then ok, so generally I can say was ok but I have I have I got stress at my work blah, blah make me headache or whatever then I got migraine you know, good what!

I_01 : Yah, so something more like qualitative something more detailed you know involving…

AFP2_0024: Yah, yah yes correct more detailed!

I_01 : More, more, more of your experiences for the whole day not just like you know one…

AF_0024 : The standard kind…

I_01 : One of time…

AFP2_0024: Yes correct, correct.

I_01 : One question ok, ok that’s very, very interesting, then what about like any other features that you like to see you know that we can add im- add on to the tablet and app?

AF_0024 : Just now you say?

I_01 : Other than this one?

AF_0024 : The videos for food like healthy food and also more about you know this is a heart problem right so I, I, I want more on, on this about my condition how it how exactly it happen or why it happen and after done the surgery what you know how it looks like you know something like that. So I can know better you know the so call the any an- anatomy of my heart itself I know my, my own heart right so I want to know so like, like now it’s already with the what’s that the metal valve right so I, if can I want to see how it looks like with that metal valve… [Participant laughed]

I_01 : Oh ok, so in- including like let for those like for you especially you want to see like what had changed that kind of thing ok, ok so more detailed about the that’s more tailored to you.

AF_0024 : So you know sometimes my friends will ask me you know sometimes they asked me why, why I cannot eat this certain food, I said not that I cannot eat certain that food it’s not, I have to minimize it lower it, sometimes you know the green vege I like to eat vege. But now because of the warfarin thinking I cannot eat much…

I_01 : Yup, yup.

AFP2_0024 : So they like they will look at me like why so, so weird I mean I, I, I I’m weird then I said because I something can be shared I take this medication so they still cannot understand if like this, this kind of video right I can show them how, why like that!

I_01 : Oh so actually the video also helped you to explain to other people…

AF_0024 : Yes, yes correct…

I_01 : Your friend!

AF_0024 : So I can tell them it’s not that I’m weird it’s just that I have to take care of myself if anything happen to me you can help me or not, cannot right, [Interviewer laughed] so that’s why I want to let them know.

I_01 : Ok, ok, ok, ok so I think one last question will be to ask you I mean any other comments that you have you know…

AF_0024 : About what?

I_01 : In general, do you or do you find the actually do you find the app right interactive or not do you feel that you’re interacting with the app?

AF_0024 : Yah.

I_01 : I mean other than because you know you answer the questions and…

AFP2_0024: Because when I answer the question it was like talking to somebody when I read I ok you know, you know something like that so if, if make it more interactive is better got picture also better…

I_01 : Got…

AFP2_0024: Got face also… [Participant laughed]

I_01 : Got, got picture and got face will be better…

AFP2_0024: Better!

I_01 : Ok, ok in terms of the messages…

AF_0024 : Yes correct, correct.

I_01 : Ok, ok that’s very interesting actually very helpful to us [Participant laughed] any, any other things you think can make it more interactive picture, face other any other things?

AF_0024 : Or maybe if let’s say right if you…

I_01 : Yup…

AF_0024 : You doing it in a long term kind of thing or maybe because 6 weeks or 8 weeks, 2 months right like every end of the week maybe we I mean you all can call us I mean I mean generally in person asking. So ok we, we, we noted on all your, your, your so call submission of your, your, your blood pressure you know your, your quest the question the questionnaire…

I_01 : Yup, yup…

AF_0024 : So now you as a person calling, asking so how generally now how you feel are you ok blah, blah, blah you know maybe can also.

I_01 : Ok, ok so like follow up…

AFP2_0024 : Yah correct, correct, correct.

I_01 : In the in the meantime in the in between.

AF_0024 : In between!

I_01 : Like having a few times follow up…

AFP2_0024: Follow up yes, correct.

I_01 : With you ok, ok, ok that that’s very helpful alright so I think…

AF_0024 : Ok that’s all.

I_01 : That’s the end of our interview thank you very much…

AF_0024 : Ok can, no problem.

I_01 : I’ll stopped the recording.

AF_0024 : Can.

End of interview
