# Supplementary material for: Patient perspectives of the Self-management and Educational Technology tool for Atrial Fibrillation (SETAF): A mixed-methods study in Singapore
Source: PLoS One. 2022 Jan 21;17(1):e0262033. doi: 10.1371/journal.pone.0262033 (PMC8782297; doi:10.1371/journal.pone.0262033)
Supplement: S1 Data — (ZIP) [file pone.0262033.s006.zip › AFP2_IDI_0023.doc]

Participant ID number : AFP2_0023

Interviewer Number : I_01

Length of interview : 29.23 mins

I_01 : So thank you very much for taking part in this study, ok the so the first question we want to know is to how long have you been diagnosed with atrial fibrillation?

AFP2_0023 : Many years ago I think maybe twenty 5…

I_01 : 25 years ago is it?

AF_0023 : Yah.

I_01 : Ok, how did you find out about it?

AF_0023 : My, my heart got pal palpitation, yah.

I_01 : Ok, ok where did you find out the about…

AF_0023 : When?

I_01 : Where?

AFP2_0023 : Where, in Hospital N.

I_01 : N so Hospital N ok the so did you take any medication or not?

AF_0023 : May… when and when I take it I mean when it in another endoban I, I take med- endoban.

I_01 : The medicine and also together with warfarin?

AF_0023 : No, no warfarin, later.

I_01 : War- warfarin much later?

AF_0023 : Yah, yah and it’s about 5 years ago…

I_01 : 5 years ago!

AFP2_0023 : When I got stroke.

I_01 : So you actually got the you were hospitalized for the stroke about 5 years ago, ok then in terms of managing your, your health right how do you normally find information to help you to manage?

AF_0023 : I, I, I go and see doctor yah, go and check up and all these.

I_01 : So doctor will give you some advice on what to do…

AF_0023 : When I what I have a renal failure…

I_01 : Ok also renal failure did they prescribe any kind of diet for you?

AF_0023 : Yeah!

I_01 : Ok so you seen the dietician also ok, but on your own outside of the hospital setting how do you do it how do you find information, where do you go to?

AFP2_0023 : Go to?

I_01 : No which, which kind of sources do you go to, to find information?

AF_0023 : I had at, at every doctor I think…

I_01 : Every doctor…

AF_0023 : I mean see doctor and dietician and all these.

I_01 : How about newspapers, Tv , radio…

AF_0023 : Yes, yes, yes…

I_01 : Which one?

AFP2_0023 : I, I listen to radio.

I_01 : Radio ok, ok so does it you… have you think that there’re any programs that can help you, give you information on what to do?

AF_0023 : Hardly… [Participant laughed]

I_01 : Hardly yah, so not much?

AF_0023 : Not, not much.

I_01 : Then what about friends and family?

AFP2_0023 : I, I had I, I do, do it on my own my own.

I_01 : On your own, your relatives or like your friends in terms of like talking to them about it you know?

AF_0023 : I, I, I seldom talk to…

I_01 : Seldom.. ok, ok so not much not that much ok, seldom means like how often?

AFP2_0023 : I, I often talk to them but then I talk not much about myself…

I_01 : Your, your, your condition.

AF_0023 : Yah, yah, yah.

I_01 : Is there any reason why you don’t talk about it?

AF_0023 : I hardly talk...

I_01 : Just, just, just don’t raise it up, do you feel uncomfortable talking about it?

AF_0023 : No, no, no.

I_01 : Ok so they know?

AFP2_0023 : Yah.

I_01 : Ok they know but…

AF_0023 : The dialysis and all these, they know.

I_01 : Ok, ok, ok then the other one ok so during this 6 weeks of the study how do you normally use the, the app and the blood pressure monitor on a average day, what did you do first?

AF_0023 : App?

I_01 : The, the tablet you know the this, this tab this tablet and the…

AF_0023 : I yeah…

I_01 : Blood pressure monitor so what did you do first when did you do it?

AFP2_0023 : Maybe last time I do it very freq- very freq- frequent…

I_01 : Last time…

AF_0023 : Maybe 10 years ago and all that.

I_01 : I mean the this, this, this the one that we gave you right?

AF_0023 : Yah, yah no, no you no…

I_01 : The so the one we passed to you to, to do for the your, your stud- during the study period how what do you normally do first?

AF_0023 : Normally wake, wakeup at the when, when I read the, the, the what… survey and then and about evening I mean I mean one o’clock at noon I take my pressure.

I_01 : About one o’clock ok afternoon ok, in the morning?

AFP2_0023 : Because…

I_01 : In the morning?

AF_0023 : Because they, they asked me to make a suitable time, then I find that my time is of about one o’clock.

I_01 : Ok, ok they means Miss M right?

AF_0023 : Yah, yah.

I_01 : She came down right and then she gave you… so did she give you any instruction of how to use the, the tablet and the app?

AF_0023 : Yah, yah.

I_01 : Do you find that helpful?

AFP2_0023 : Yah, ok.

I_01 : Ok so like did you did you find that it ok to understand?

AF_0023 : Yah I, I, I make use of a tablet at, at home. [Participant laughed]

I_01 : You have your own tablet already ok, then let’s say Miss M came down to pass you the tablet and the blood pressure monitor but did not explain to you do you think that you will be able to use it or not?

AF_0023 : A little…

I_01 : So she… a little, still will be able to…

AF_0023 : My… I on and off and then I, I play, play around.

I_01 : Play around ok, ok so which part of the tablet and the app are overall right was your favorite I mean it was, was the easiest or favorite for you all, for you?

AFP2_0023 : Yah, [Participant laughed] the easiest is the survey…

I_01 : The survey ok, then the most difficult?

AF_0023 : Not much I mean eas- easy I mean very easy.

I_01 : Ok, very easy ok. Then did you watch the videos or not?

AF_0023 : Yah.

I_01 : Ok how do you find the videos?

AF_0023 : Not, not so in interesting then the, the first part that about the atrial…

I_01 : AF.

AFP2_0023 : AF and stroke and all these… interesting.

I_01 : Interesting, in what way?

AF_0023 : I, I get know- know- knowledge.

I_01 : Knowledge ok, this knowledge right last time before you watch the video did you know about it or not?

AF_0023 : A, a, a slight…

I_01 : Slightly…

AF_0023 : Yah, yah, yah.

I_01 : Some you, you knew a little bit.

AFP2_0023 : Yah, yah.

I_01 : But after watching was it bet- you think you know more?

AF_0023 : Yah, yah, yah.

I_01 : So yes, ok then what about the cooking and exercise videos did you watch?

AF_0023 : Exercise I…

I_01 : And the cooking one?

AF_0023 : Cooking?

I_01 : Yah also, also cook…

AFP2_0023 : Yah, yah I, I ignored that. [Participant laughed]

I_01 : You ignored so you didn’t watch at all?

AF_0023 : Yah because I just glanced through, yah.

I_01 : Ok, any reason why you…

AF_0023 : I, I…

I_01 : Don’t really want to watch?

AF_0023 : I, I know already.

I_01 : You know already ok, you know what to do is it?

AFP2_0023 : Yah, yah.

I_01 : You got these knowledge from where before?

AF_0023 : The dietri- cian dietrician and picked up some tips at hand, can.

I_01 : Ok, but ok so like the dietician gave you advice did you follow up and you know do your on your own or not?

AF_0023 : No.

I_01 : Oh never really?

AF_0023 : Yah. [Participant laughed]

I_01 : Do you think if we have such a app you know such videos would help you to like do it more like to kind of encourage you to do it like exercise, cook better…

AFP2_0023 : Yah, yah.

I_01 : You think will help or not?

AF_0023 : Can ok, can, can.

I_01 : Yah, can meaning what?

AF_0023 : Can help. [Participant laughed]

I_01 : Can help ok, but because you didn’t you said you didn’t watch right so how can we…

AF_0023 : No, no, no I, I flipped through only.

I_01 : Flipped through only never really watch through everything?

AFP2_0023 : Yah, yah.

I_01 : Ok, so only like glanced through it ok, so what can we encourage you to watch it more like to you know really watch, watch it what can we do you know to help you?

AF_0023 : A bit slow it, it the, the that exercise you know a bit slow.

I_01 : A bit slow meaning the, the length is too long, the length of the video too long?

AF_0023 : No it, it break repeat…

I_01 : Repeat, repetitive so you find it like repeating… a bit…

AF_0023 : Boring.

I_01 : A bit boring ok, ok. [Interviewer laughed] Ok then so you pre let’s say we do it on a like a shorter video would you think that it’s better or not, shorter videos, more videos?

AFP2_0023 : Maybe.

I_01 : Maybe ok, ok so maybe means?

AF_0023 : Shorter means you, you, you, you do it once and then [Participant and interviewer laughed] I, I, I don’t…

I_01 : Never mind take your time, it’s ok.

AF_0023 : You…

I_01 : Slowly, slowly.

AF_0023 : You, you like, like say sit up and, and sit down and all these and then and, and the, the following one… maybe another, another…

I_01 : Another video?

AFP2_0023 : Yah.

I_01 : Oh so that means like one video like one topic one small topic…

AF_0023 : Yes!

I_01 : Or small a- subject area or one, one, one instruction is it?

AF_0023 : Yah, [Participant laughed] yah.

I_01 : Ok, [Participant and interviewer laughed] yah you know if you know what I mean right?

AF_0023 : Yah.

I_01 : Ok, ok then anyway right you said you did the survey how you find the survey, were there any emotions that come up when you do the survey?

AFP2_0023 : This, this that, that survey your you…

I_01 :The survey in the, the survey in the app the app the, the…

AF_0023 : Ok, no, no I…

I_01 : No means you did the survey?

AF_0023 : Yah.

I_01 : Ok yah, so how’s the survey?

AF_0023 : Ok, I mean no, no in interruption or what…

I_01 : No interruption, but how do you feel when you do the survey?

AFP2_0023 : How do I feel?

I_01 : Yah how do you feel?

AF_0023 : Normal. [Participant laughed]

I_01 : Normal, normal means what?

AF_0023 : I…

I_01 : Like do you…

AF_0023 : No, no it interruption or I, I find it like nor- normal I, I, I take my pressure and all these yah like…

I_01 : Yah ok, ok then like did you find that the survey was a bit too long or normal or too short?

AFP2_0023 : This one…

I_01 : Yah, the survey as in the one the tablet survey, the tablet survey the one that you do one…

AF_0023 : Yah, yah…

I_01 : Yah the quest- there are many, many questions?

AF_0023 : Ok, too short.

I_01 : Too short ok, ok so just now I, I was asking the survey was about, about the, the tablet one then the emo- how do feel like during that time?

AF_0023 : I, I, I don’t feel anything…

I_01 : Don’t feel anything it’s ok, then what about like popped up messages you know there are some popped up message to encourage you, give you diet advice how did you feel? Those popped up messages…

AFP2_0023 : What, what…

I_01 : Popped up message means like those, those small little short message…

AF_0023 : Yah, yah, yah…

I_01 : They prompt, then you like ok, ok, ok so like how, how is those messages how do you find it?

AF_0023 : Ok, I mean… [Participant laughed]

I_01 : Ok means what ok means did you do any did you do then did you do it?

AF_0023 : Yah, yah.

I_01 : Ok then, was there any like feelings that, that, that, that, that you have when you saw the messages?

AFP2_0023 : No.

I_01 : Like the encouraging messages do you feel better or not do you feel happier or not?

AF_0023 : Happier…

I_01 : Or like more satisfied?

AF_0023 : [Participant laughed] Normal I, I feel like…

I_01 : Normal?

AF_0023 : You, you just take and, and that’s it. [Participant laughed]

I_01 : That’s it ok, you did you feel anxiety or not?

AFP2_0023 : No.

I_01 : Anxiety means like scared?

AF_0023 : No.

I_01 : Don’t have?

AF_0023 : No.

I_01 : Ok, ok then what about like the blood pressure monitor also it, it was it easy or difficult?

AF_0023 : No, I’m not… easy!

I_01 : Ok easy then how did you feel did you feel stress, anxiety when you when you take your blood pressure?

AFP2_0023 : No, I don’t know.

I_01 : You don’t know, but you said that there were sometimes when your blood pressure was a bit high is it?

AF_0023 : No yah, pulse… pulse, pulse…

I_01 : Pulse, but we never measure pulse what we only measure blood pressure.

AF_0023 : Yah I, I, I, I, I was talking about pulse at high, high, high or low but normally is pres-, pres-, press- pressure is, is normal and then it, it said very good and all these and then that’s all and then everything is good but pulse in my side is high.

I_01 : So you feel that this device never measure the pulse…

AF_0023 : Yah, yah, yah.

I_01 : So don’t give you the feedback on the on the pulse the reading ok, ok so that you, you want to see something more like the pulse reading that kind ok, ok, ok. Because actually just to explain to you we are starting very small first then maybe in the future we might expand to more features so we really want to hear from you also what you want see yah, yah! So you want to see the pulse and, and any, any other things that you perhaps want to see in this base on your experience?

AFP2_0023 : Not much but the, the, the, the pulse is very important.

I_01 : Pulse is very important ok, you feel it’s impor- you want it you want to see it, ok.

AF_0023 : And, and I meant AF…

I_01 : AF…

AF_0023 : It is measuring the pulse…

I_01 : And also blood pressure because blood pressure is related to your medication so your medication right the medicine you take you cannot you cannot have too low blood pressure .

AF_0023 : Yah I know, I know.

I_01 : Yah so it has to be at say right level otherwise it will cause complications ok, anyway the, the tablet right how do you feel about the screen the size, is it ok?

AFP2_0023 : Ok.

I_01 : Ok, let’s say we have a few options for you we use ok we have a phone we have a tablet we have a desktop computer and also any other elec- electronic device what will you prefer to use which form, which…

AF_0023 : Tablet.

I_01 : Tablet ok, ok.

AF_0023 : Tablet!

I_01 : Why, why tablet?

AF_0023 : [Participant laughed] My eyesight!

I_01 : Oh eyesight ok, ok so you eyesight meaning you can you, you, you…

AFP2_0023 : I’m…

I_01 : Want to see a bigger screen or a smaller screen?

AF_0023 : Bigger!

I_01 : Bigger screen better…

AF_0023 : Not…

I_01 : But the tablet that we gave you, was it ok?

AF_0023 : Ok, ok but my eyesight is a I, I used the phone on and off but then tablet is better.

I_01 : Tablet better ok, ok so ok so what about like the font the font meaning the size of the words?

AFP2_0023 : Yah, yah ok.

I_01 : Ok, ok the brightness?

AF_0023 : Ok, the brightness...

I_01 : So, ok meaning?

AF_0023 : Yah… you mean…

I_01 : Just right?

AF_0023 : Yah, just right.

I_01 : Just right ok, ok then what about like if the if the navigating the app navigating the app meaning like you know when you use the you on already then you go, go into the different features. Like see your blood pressure or you go and watch the video is it ok to go through all these things you was have any problems?

AFP2_0023 : No problems.

I_01 : Technical problems?

AF_0023 : No, no problems.

I_01 : Ok, it’s ok, ok so the one did you know that there was a number on the tablet or not a number…

AF_0023 : Yah, yah.

I_01 : You can call one…

AF_0023 : Yah.

I_01 : Did you try calling?

AFP2_0023 : No! [Participant laughed]

I_01 : Oh ok why, why didn’t?

AF_0023 : No, no, no problem.

I_01 : No problem ok so, so you didn’t call you didn’t use it, you feel that would it be better for us to have a, a number there or not have a number?

AF_0023 : I think it it’s better because when problem and you, you can call.

I_01 : Ok, so you it it’s use…

AF_0023 : Then, then…

I_01 : Useful!

AFP2_0023 : Maybe a, a short a, a short one let’s, let’s say when there a problem call this, this number and because too long… [Participant laughed]

I_01 : What’s too long?

AF_0023 : The, the, the instruction…

I_01 : You mean the instructions on the, the, the instruction manual that they gave you is it?

AF_0023 : No, no, no the, the, the tablet.

I_01 : The tablet in, is it the one inside?

AF_0023 : Yah, yah.

I_01 : Oh it’s like the instruction too long?

AFP2_0023 : Yah.

I_01 : What, what instruction is that?

AF_0023 : It, it say… [Participant laughed] or [Incomprehensible segment]

I_01 : Maybe I can on it.

AF_0023 : Cannot?

I_01 : Which, which instruction?

AF_0023 : No, I it’s, it’s, it’s not on…

I_01 : Ok it’s not on yet.

AFP2_0023 : When, when you, you, you when, when you short shorter and short time…

I_01 : Shorter, shorter messages…

AF_0023 : Yah, yah.

I_01 : Shorter instruction on what to do oh ok, ok you find it a bit too long?

AF_0023 : Yah, [Participant and interviewer laughed] yah.

I_01 : Ok, ok then do you know that there’s a feature where you can track your blood pressure the graph…

AF_0023 : Yes.

I_01 : So let did you see a doctor in this time period to you know you bring your results to the doctor in this time period?

AFP2_0023 : No.

I_01 : Don’t have ok you didn’t use it ok, ok you think that…

AF_0023 : I…

I_01 : It will be useful for to have this kind of graph to or this kind chart to be able to show your doctor in the future?

AF_0023 : May- maybe I, I, I just, just the pressure en- I, I enough already.

I_01 : Can be other reading next time also but at the moment it’s like the blood pressure only do you think that it’s useful for like for you to see the doctor with that or not?

AF_0023 : I, I didn’t use.

I_01 : You didn’t use ok, ok but like but let’s say you go and see a doctor you think that can help?

AFP2_0023 : Maybe.

I_01 : Maybe, maybe means?

AF_0023 : Yah, this you, you maybe your, your pre- pressure high and low it, it, it’s, it’s [Participant laughed] also no normally you… I at N place we, we, we do a, a…

I_01 : Monitoring?

AF_0023 : Chart…

I_01 : Chart ok.

AF_0023 : Then not, not, not chart like the said when… where our when, when blood pre- blood pressure is taken and then when and all these…

I_01 : Your result…

AFP2_0023 : And yah, yah… it, it’s good enough.

I_01 : It’s good enough ok, but in ok can, then what about like would ok let’s say we have a paper form, paper form meaning like you record it on paper then you send it to us and then like we send you some brochure for information, would you prefer like paper form or electronic form?

AF_0023 : Electronic!

I_01 : Electronic form, why you prefer electronic form?

AF_0023 : [Participant laughed] Easier!

I_01 : Easier, ok.

AF_0023 : Because e- electronic you, you can, can send… better, than paper form you got to hand over and all these.

I_01 : Yah, yah and mail.

AFP2_0023 : Yah, yah.

I_01 : Ok you, you so you prefer electronic ok then let’s say a few more questions do you have anything that you would like see I mean that we can add on to improve this app and the this program that we have other than the one you earlier you mentioned about the pulse. Taking your pulse right any other feature you can think of that we can add on to it, to take away… that we can improve on?

AF_0023 : Not, not, not, not, not much.

I_01 : Ok, ok not much ok, [Participant laughed] any comments on your o- your experience or not do you face any like difficulties, challenges during this 6 weeks or not?

AF_0023 : Yah, yah.

I_01 : What, what challenge?

AF_0023 : [Participant laughed] I was hospitalized, hospitalized.

I_01 : You hospitalized, how long were you?

AFP2_0023 : 5 days!

I_01 : 5 days, so you couldn’t use it during that time ok, ok.

AF_0023 : And because of my heartbeat.

I_01 : Did you try to let us know that the call the number or not?

AF_0023 : No.

I_01 : Don’t have ok, then like why do you say that is a pro- is a challenge the when you hospitalized?

AF_0023 : Because you’re my, my I can’t like dialysis because the, the pulse rate is high and then I [Participant laughed] got to hos- hospitalized then…

I_01 : Ok, but you, you mentioned there is a challenge during this 6 weeks?

AFP2_0023 : Yeah and then you the, the a, a, a dust adjust a adjustment in, in tablets and all these and then I got to stay for 5 days.

I_01 : Adjustment in tablet what…

AF_0023 : Yah then…

I_01 : Meaning, what do you mean?

AF_0023 : Warifin, warfarin…

I_01 : Warfarin yah!

AF_0023 : Be because I take 2 and a half, then they, they changed to the doctor they changed to 2 and then the, the calcium the they, they I, I mean they take me take 3, 3 everyday, then they changed to one.

I_01 : Ok, ok that was when you were hospitalized, ok.

AFP2_0023 : Yah but then there’s no change one then I…

I_01 : No change what?

AF_0023 : My, my pulse, no change!

I_01 : You pulse or your or your blood pressure?

AF_0023 : Pulse!

I_01 : Pulse ok, ok no change ok, so you mean like ok you after you hospitalized did not affect your pulse?

AF_0023 : Yah, yah no.

I_01 : Ok, ok any other challenges that you faced like during the time this time period when you when you do the this study other then the hospitalized…

AFP2_0023 : No yah, yah. [Participant laughed]

I_01 : Ok, ok so like let’s say we introduce this device to you when you were first diag- when you were first diagnosed right do you prefer us when do you prefer us to introduce this device when you first diagnosed or later a bit or in the middle. So when will be like a good time for us to introduce this like looking, looking back you know you know on the on your experience in this study right? Let’s say we try to introduce this device to patients right you think that we can we should introduce it where at the beginning when they have when they were diagnosed with AF or in the middle or later a bit?

AF_0023 : Maybe… beginning.

I_01 : Beginning why, why beginning?

AF_0023 : So that you, you… you monitor the… the, the…

I_01 : The…

AF_0023 : E- effect, I mean…

I_01 : The result the, the condition the reading?

AFP2_0023 : Yah, may maybe earlier.

I_01 : Earlier ok, ok so like you think is better?

AF_0023 : Yah.

I_01 : Why is it better?

AF_0023 : Because of [Participant laughed] my, my sid- my side already too high already I, I AF too high already then, then there’s not- nothing you can do.

I_01 : You can do is it, but what about man- how about managing you think that can help you to manage because I know it’s you’re you are a bit like you have it for many, many years already and you have also other you have…

AF_0023 : Condition.

I_01 : Conditions right do you but at the moment you think that can help you to like maintain or not maintain manage…

AFP2_0023 : Maybe, maybe exercise or…

I_01 : Yah, yah so, so this kind of tablet can, can help!

AF_0023 : Tablet?

I_01 : I mean this, this tablet this device can help you to maintain or manage you think so or, or not or it doesn’t really make a difference?

AF_0023 : Not make a big difference.

I_01 : It doesn’t really make a big difference ok, ok, ok can ok, ok can so act- actually that’s all about it we want to find out your how you feel you know ok about how about using this device yah, ok that means ok anything else to you comment or anything?

AF_0023 : No… no.

I_01 : Ok, stopped the recording.

End of interview
