# Supplementary material for: Patient perspectives of the Self-management and Educational Technology tool for Atrial Fibrillation (SETAF): A mixed-methods study in Singapore
Source: PLoS One. 2022 Jan 21;17(1):e0262033. doi: 10.1371/journal.pone.0262033 (PMC8782297; doi:10.1371/journal.pone.0262033)
Supplement: S1 Data — (ZIP) [file pone.0262033.s006.zip › AFP2_IDI_0021.doc]

Participant ID number : AF_IDI_0021

Interviewer Number : I_01

Length of interview : 44.16mins

AF_0021 : You test it and see whether it’s working?

I_01 : It is working yes ok…

AF_0021 : Alright very good.

I_01 : So thank you very much for, for taking part in this interview…

AF_0021 : Sure.

I_01 : And, and helping us with this study.

AF_0021 : Sure.

I_01 : So actually we want to ask you right the first question is that you know how long have you been diagnosed with the AF and irregular heartbeat?

AF_0021 : Oh that is a long time long time…

I_01 : How yah…

AF_0021 : Maybe, maybe for the last 30 over years from various doctors you know from my first cardiologist who is my brother’s classmate you know I can’t remember his name now I think he’s already retired but he’s a very well known cardiologist. But we, we he knows about this problem this you know Fib- fibrillation and later you know when I was working and at a certain level you know when I go for my grade “A” different grades. But when I’m at the executive level they gave me the whole works you know and they found out about this already and some of the doctors you know like in Hospital “B” one of the doctor said that you know I can die of a stroke you see…

I_01 : Oh ok.

AF_0021 : But the uttered rubbish I went through my national service in, in combat went to officer cadet school I’m a prize winner you know I’m the best cadet before they have the name iron man they already called me iron man in “C” [Interviewer laughed] and all the time I have this problem you know?

I_01 : You even before you went to NS?

AF_0021 : Yah no this is something that is born I’m born with you know alright?

I_01 : Congenital right?

AF_0021 : Alright and the important thing is that I gone through all the exercises and everything I didn’t die number one number 2 before I got into this trouble you know when I only got into this trouble when I went through a very bad period of mourning because my wife passed away alright? Before that and after that you know both my parents passed away also because you know they were so dependent on my wife to look after them you know but to cut all these short right every morning right I run 7 kilometres ok before I get into this trouble you know 3 years or 4 years ago. Without fail 7 kilometres.

I_01 : 3 or 4 years ago?

AF_0021 : Yes.

I_01 : So that was long after you were diag- you, you were diagnosed with AF?

AF_0021 : Oh yes.

I_01 : Long after.

AF_0021 : 7 klicks I get out from here I get out “D” go up “E” all the way towards the back of…

I_01 : Wow it’s quite long. [Interviewer laughed]

AF_0021 : “F” go up right and then “G” and then down again.

I_01 : Ok so why do you change why, why, why do you like you know…

AF_0021 : No because I know I had this problem you know then I stopped you see but now you know that I’m feeling much better as soon as now you know my, my the specialist you know looking after me a new a new cardiologist you know he is doctor “H” he’s from Indonesia. Before that it was a professor assistant, assistant professor or full professor “I” you know he, he, he has gone into the private sector so this doctor “H” took over he’s so happy with my results that he says you come back and see me only 6 months later. Right so what happened is that the moment I, I, I’m finished here with this, this interview and then you know a little bit of tightening up you know or whatever that I’m under medication I will go for my run.

I_01 : Ok what kind of medication you are taking?

AF_0021 : Oh the usual you know for to prevent stroke and all these you know they said that when I when I first was put under this MRI they said that I had…

I_01 : I see.

AF_0021 : 4 strokes you know history of 4 strokes before but I did not die from it you know and because of that you know I’m under medication.

I_01 : Medication like warfarin all these…

AF_0021 : Yah, yah that’s what the, the usual things.

I_01 : The usual things ok, ok.

AF_0021 : And they said that you know I’m a bit a bit diabetic you know maybe is because it’s not type one you know but type 2 for age right but it’s under control.

I_01 : Do you take medication for diabetes also?

AF_0021 : Yah, yah, yah dia- diatram or whatever you know so that’s the usual thing.

I_01 : Ok, ok so how do you find out about your like to manage this AF other than the from the doctor right that’s why are there…

AF_0021 : No, no the, the AF thing is over…

I_01 : Many years…

AF_0021 : No, no it’s, it’s, it’s been with me all the time and what happen is that you know unless you know they tell me hey this is very bad you know I have to put a, a pacemaker but I don’t need a pacemaker you looked at my blood pressure results you know right?

I_01 : Yah it’s very stable.

AF_0021 : It’s consistent stabled so what more do the doctors want I can easily tell the doctor looked I don’t want any treatment.

I_01 : But on your own like you know you want to manage the…

AF_0021 : No but I don’t have to manage it.

I_01 : Yah, yah ok so you don’t you don’t…

AF_0021 : I don’t feel I don’t I don’t have a…

I_01 : Ok you don’t feel you need to…

AF_0021 : My heartbeat is whatever it is normal or not normal I still I just told you right every morning without fail I go for a…

I_01 : Can.

AF_0021 : 6 to 7 klick run you know and you looked at me I am 71 years old do I have any [Interviewer laughed] bandage on it, why because I warmth up before I run…

I_01 : Ok so you…

AF_0021 : My all my other classmates, my friends of my same age they will have problems with their knees with their ankles and all that for me nothing.

I_01 : Ok so it’s very good anyway the you, you got the do you get sources of information for like say you know general generally health…

AF_0021 : Very important…

I_01 : Health issues?

AF_0021 : Very important let me tell you let your body tell you how you feel if, if you are conscious of your own body you will never go wrong.

I_01 : Ok, ok, ok so you, you pay attention a lot to your…

AF_0021 : Of course.

I_01 : To your health your body…

AF_0021 : No I drink I smoked you know but reg- you know on a reg- on a, a modest you know I can drink very heavily I can also not drink at all part of my work you know part of me talking to people you know when I’m overseas I’m based in so many…

I_01 : For business you mean?

AF_0021 : Countries you know when I was with the “Z” so that is not a problem.

I_01 : Ok, ok so like you know when you during this time period of the study the 6 weeks like what are the ss- the routine that you go through everyday like what’s the sequence of your you…

AF_0021 : No normal I wake up you know do my 3 s-es and then after that I’ll be on my on my computer but before that you know because I was still working you know I worked for Company “Y” for a very long time. So I was actually based in country “X” and well I, I do my normal things when I’m in you know overseas.

I_01 : Yah, yah in the morning or in the afternoon…

AF_0021 : No it depends you know well about the I…

I_01 : It’s about the it’s ok.

AF_0021 : I normally my, my habit you know from, from, from the beginning when I started working is that you know I find that I will only want to have lunch meetings you know with my clients or visitors why because if it’s dinner it will always end up in a karaoke [Interviewer laughed] or a nightclub. And I’m not into these you know so I will never have unless it’s very important and you know I will always tell people you know that after dinner you know 10 thirty I am leaving already so no point spending thousands of dollars you know in karaoke and all that you know uttered rubbish alright.

I_01 : Yah, yah you so you, you when you were doing this tablet device right do you feel that it was interfering in your work?

AF_0021 : No doesn’t take me more than 5 minutes you know to get it done but I don’t see the results you know I don’t see the, the efficacy…

I_01 : Oh ok yah why?

AF_0021 : Because the there’s no feedback you know it’s only one way you know and the feedback is always oh well done you know you are under normal this and that you know and that’s it you see?

I_01 : Yah, yah the messages?

AF_0021 : Yah the messages and then I don’t know you know there were these surveys you know…

I_01 : Correct yah.

AF_0021 : At one stage I thought the surveys were just you know very normal surveys right but then later they become you know I, I supposed you know it’s a question of whether the people who designed the, the survey especially when they are not in front of you, you know? The way they put the questions to you, you know and then and repeat the questions in different way and all that just to find out whether you know you are telling the truth. But let’s faced it you know there are people who are now in, in, in the parliament you know these are people who have been coached you know and pay good money to get into the party “W” and into government. Because they have psychologist friends and all that but people who pay really good money you know to coach you, you know how to answer so what is what is me you know trying to con no point. [Interviewer laughed]

I_01 : But how do you feel about the, the questions do you feel that they are like the phrasing…

AF_0021 : Oh they are you know, know the normal…

I_01 : The phrasing kind is it?

AF_0021 : The normal the normal kind of questioning like you cannot be very intrusive at the same time you know what you know what there’s not enough of medical data you know to make a doctor a proper questioner who is a doctor really asked important questions. Like I tell I can tell you, you know if you are a doctor l said looked you know I may have this problem in my chest you know in my lungs in my heart but I can run. Of course now I because I had not been running for at least 3 years so I need to build up my stamina and all that but if you want to take a bet with me not for any money I will tell you in 6 months you run with me.

I_01 : [Interviewer laughed] Ok so that’s yah that will be challenging right?

AF_0021 : Yah but the thing is that you know I are am, am able right from that moment I knew that I was going into national service I always the first thing to do is what do you do in the army you have to be fit you know when you are fit you enjoy your exercises.

I_01 : You yah…

AF_0021 : I have never from you know my, my service was 3 years you know full time I have never ever gone to see a doctor to say that I have a problem with my knee I have problem with my ankle, never!

I_01 : Ok, ok so it was ok throughout so anyway the, the questions that came out was it very too you find it too regular too much too regular the frequency I mean?

AF_0021 : No I I’m a educated man right so I can understand why they are asking all the questions but I’m not sure you know people who are you know maybe at “O” or “A” level you know would want to you know go through this process you know.

I_01 : Yah so it may be a bit too difficult?

AF_0021 : I no I don’t think so but it personally right if I’m taking for the other…

I_01 : People.

AF_0021 : People who are you know especially those who are not educated how are you going to put across to them you know maybe the questions you know you feel well you don’t feel well all that yah. You, you have when you when you walked you know do you find that especially up staircase you know or up, up, up slopes and all that do you find that you know you, you have difficulty you know in and you need to take a rest right. I was in country “U” my daughter and her friend you know they have been to country “U” before they know Country “U” very well and we go to places that are very…

I_01 : Steep!

AF_0021 : Steep you know I have no problem.

I_01 : Ok, ok, ok then what about like the videos did you manage to watch the videos…

AF_0021 : Yah, yah you know…

I_01 : How do you find it?

AF_0021 : Some of the…

I_01 : Honestly?

AF_0021 : Exercises you know the video on exercises you know that 9, 9 exercises for you to do it’s rep- repetitive you, you know I cannot I am a person who say now you tell me you gonna spent 10 minutes on these or you want to spend half an hour, right? And if you want to spend you want to want if you want me to spend half an hour on these right you better tell me you know this is the general picture the, the, the power point right these are the things you need to do. See the people who designed these questions right should learn from the people in Company “V” you know where they give you a pic- a big picture first macro picture and then after that give you modules of it you know but all re- related you see. You see 3 people you know of 3 different race doing exercise swinging this and that you know looked [Interviewer laughed] I do more vigorous exercise than these you know so forget it.

I_01 : Ok, ok, ok so you found that you actually feel that you preferred like if there’s a overall picture more, more detailed content and then you, you’ll be more…

AF_0021 : No, no you see then you can have a choice you know you want to repeat this you want to see something else so there’s not enough of inter activity you see, right?

I_01 : Ok, ok not interactive ok, ok then, like what about you there’s one AF information video did you manage to have a look at it?

AF_0021 : Yah, yah I know about that.

I_01 : How do you find it?

AF_0021 : That’s all uttered rubbish.

I_01 : Why?

AF_0021 : Why they tell me you know it doesn’t apply to me I don’t feel know my heart don’t give, give a skip you know even if I see the most beautiful girl [Interviewer laughed] my heart will be normal you see I won’t my heart beat will not missed a skip. So it doesn’t you looked at me talking to you now do I looked like somebody who is breathless?

I_01 : No I mean I that’s why we want to know find out and, and…

AF_0021 : Precisely.

I_01 : What how you feel.

AF_0021 : So the thing is that you know I am not one of those under the category you know where doctors will say looked this is a serious case this is not a serious case in the first in the first instance right I think the cardiologist must decide you know over a period of time whether this is a serious case. Mine is a serious case or it’s not you know and after so many years you know being diagnosed with this kind of condition I’m still around I run I used to play golf I just came back from 2 trips you know to Country “T” one to state “S” and one to resort “R” in a car.

I_01 : So if the videos were more tailored to you you’ll be you feel bet- you’ll be better?

AF_0021 : No I don’t think it can be tailored to me…

I_01 : Cannot?

AF_0021 : Because I’m not the normal patient of AF.

I_01 : AF, ok, ok can ok then like the blood pressure monitor right do you how do you find like taking it was it troublesome for you?

AF_0021 : No, no I, I, I have my own I’ll show you I have my own…

I_01 : Ok so…

AF_0021 : I have my blood, blood sugar everything is here.

I_01 : Ok, ok and you take it every regularly.

AF_0021 : Yah this morning I just I tried it on the, the new one and then I tried it on this one.

I_01 : So you take it 2 times, both?

AF_0021 : No, no, no I when I when I knew that this study is already concluded I ss- like this morning I didn’t I didn’t take any reading.

I_01 : Ok you switched to your, your one.

AF_0021 : I went to the old one.

I_01 : Ok, ok so when you take do you feel any what are the some of the emotions that come about do you have any…

AF_0021 : Nothing!

I_01 : Nothing ok.

AF_0021 : Nothing I just take it you know just…

I_01 : Ok, ok just, just wondering only right.

AF_0021 : Squeezed and then that’s it.

I_01 : Yah ok then what about like the there was also like ok the, the popped up messages you, you mentioned that it was not really useful right or, or you…

AF_0021 : No it’s not very useful it’s just that it’s a standard response you know right and I supposed you know the, the reply you know is that if I’m within that parameter then it will be the standard reply you see, right?

I_01 : So it’s very it’s very like you, you know that it’s actually a standard reply very obvious to you?

AF_0021 : Sure.

I_01 : Ok can ok then like what how do you feel about the interface do you find that it was like the screen too small for you or not?

AF_0021 : No, no, no.

I_01 : Ok or too big?

AF_0021 : No, no, no.

I_01 : It’s ok just right?

AF_0021 : It’s ok you know no big deal if I can handle a [Interviewer laughed] a 4G phone you know this is nothing.

I_01 : Ok then what about like the font is it too small too big?

AF_0021 : No I can always controlled it what.

I_01 : Ok, ok yah then the color everything was it was too bright…

AF_0021 : No not a problem not a problem.

I_01 : Ok so it’s all ok, ok we want to find out about that ok then like did you manage to talk to when Miss “Q” came here to pass you the device is it?

AF_0021 : Yah that’s all.

I_01 : Ok did she ex- give you like explained to you how to do it…

AF_0021 : Yah, yah I know exactly yah.

I_01 : How do you feel about it was it useful for you or not?

AF_0021 : That’s a standard [Interviewer laughed] a standard thing…

I_01 : Standard thing ok.

AF_0021 : I’m not a I’m not a child you know [Interviewer laughed] I, I built my own computers you know…

I_01 : Ok sorry like what kind of computers?

AF_0021 : Before my, my desk top…

I_01 : Oh the desk top ok, ok.

AF_0021 : It started when we had…

I_01 : The early time.

AF_0021 : 286 then 386, then 486 I do my own…

I_01 : Oh wow that was quite, quite good.

AF_0021 : Yes I changed the colour you know of my graphic, graphic card from 256 to you know 14 million colours…

I_01 : Ok so you are very comfortable with technology already for a long time already.

AF_0021 : Sure.

I_01 : Oh ok, ok, ok that and it helps.

AF_0021 : I am a [Interviewer laughed] registered Company “V” data tester in the old days right before the internet was so fast they will sent me CD roms you know…

I_01 : To test the…

AF_0021 : From for me to test out some of the programs for them…

I_01 : I see ok you still have it around?

AF_0021 : You look at my, my window stand right it is the latest you know latest built 1803.

I_01 : Oh ok, ok yah they had it for I think they still had it now also the better testing that one.

AF_0021 : Of course now they called it experienced users you see.

I_01 : And they still need people to do it.

AF_0021 : Of course and I give them good, good information because when I was in service you know I have easily 400 people under using computers you know and I will tell them you know all these people you are stupid you know?

I_01 : Oh ok why?

AF_0021 : Why… because there are stenographers and secretaries when they do up a let’s say a spreadsheet you know about ok we are going to have our company AGM you know off site you know that means in, in country “X”. So they will produce you know spread sheet you know where who are involved this and that you know the task and this and all that right and they keep on sending me stuffs right when there are amendments right they don’t put this is you know the latest update you know. And the reference number and all that they just keep on typing it there into the usual template you know and just make the changes without even highlighting you know where the changes are…

I_01 : Oh ok so it’s all…

AF_0021 : Is that the way you used the computer?

I_01 : [Interviewer laughed] No you have to do it you have to list the memory…

AF_0021 : You got to annex 1 annex 2 you know and then now you list out you know where it’s important you know you highlight the changes alright?

I_01 : Yah [Interviewer laughed] so you do you know there is a medical team for the for this tablet and then there is a number to call?

AF_0021 : Yah I, I called them once…

I_01 : Did you try calling…

AF_0021 : And the lady says…

I_01 : How was it?

AF_0021 : She, she is multi tasking you know she is the secretary to the doctor who is in charge of this project you know and she, she I wanted to tell her that you know I am going to City “Q” you know and she said that I just take it down you know that’s it no feedback. So there are no people specially but it’s not important to me you know why because looked I’m going already right nobody is going to stop me you know so I’m just going to be here absent you know from let me throw this away…

I_01 : Yah ok.

AF_0021 : You want a drink?

I_01 : Water or something?

AF_0021 : Just plain water?

I_01 : Yah just yah plain water will do, thank you… thanks… so what kind of feedback I just to ask would you like to have like I mean ideally what kind of feedback would you like to, to see you know in this in using this kind of app where you…

AF_0021 : I think so far my, my, my only reaction is that you know so far whatever that has been recorded right over the period that…

I_01 : Thanks.

AF_0021 : The this survey was done I don’t think any medical doctor is actually seen the results my results you see?

I_01 : Actually the doctor did looked at it…

AF_0021 : Really…

I_01 : Briefly.

AF_0021 : Yah but then you know as long as you know the parameters are, are you know within the so there’s no, no big no, no, no need for alarmed you know and the doctor will probably say this guy must be strange you know how come you know he’s got AF but yet his results still very stable you know.

I_01 : But there’re man- there are many severity right so…

AF_0021 : Precisely so I am not one of those I just told you I can run I still smoke I have no problem sleeping [Interviewer laughed] I wake up regularly at the you know so what is there? [Participant laughed]

I_01 : Ok then let’s say for the duration of this study 6 weeks was it too what how do you feel about the period…

AF_0021 : It’s ok no problem.

I_01 : It’s ok?

AF_0021 : I, I I’m not bordered by it you know.

I_01 : Ok you are not bordered.

AF_0021 : I don’t have to change my…

I_01 : Your lifestyle?

AF_0021 : Rou- my routine yah…

I_01 : Routine yah, yah.

AF_0021 : To take part in this.

I_01 : So it didn’t really affect…

AF_0021 : It takes me only less than 10 minutes you know to hooked up you know and then turned it on and then finished, end.

I_01 : Very fast ok, would you recommend any other thing that we can add or, or you know some general like kind of ideas on how we can improved this you know this device because you know as you as you know that, that we might want to implement it on a larger scale. Use more technology to help you know patients of all kinds of all conditions so like how can we improved on this, this, this app…

AF_0021 : No you see that…

I_01 : This, this device?

AF_0021 : This, this device is just to monitor right it does it is not something where doctors or trained personnel involved in this AF problem will make house calls you see right because the cost will go up so my only conclusion is that you know for me right it’s a waste of time. Because you know I’m not affected by one way or the other right but for those who really are suffering you know it’ll be good if these people are monitored lo- closely and then help comes to them you know in person form.

I_01 : Ok in per- so…

AF_0021 : So that so that you know they can improved their livelihood you know right if they are actually suffering you know they have got ss- their heart is no good you know this and that right they can’t go they can’t walk they can’t you know move about and all that. How these people can be their life can be improved you know and probably you know after some survey you know to make them go through a regime of exercises you know to improve their bodily function you know.

I_01 : So after the survey there will be a like exercise feature it like concurrently…

AF_0021 : Whatever, whatever you know the physiotherapist whatever but you see some of these people who are selected for the survey should be more stringent you know to see whether these people can actually benefit from it or not, right? Many of these are not English educated you know so there is a problem well with language you know?

I_01 : So there is one thing they can improved the language part?

AF_0021 : Yah the language part you know?

I_01 : Yah more accessible culturally to people.

AF_0021 : Not culturally just to make them…

I_01 : Language only.

AF_0021 : Make them understand you know the importance of some of these messages you know that the, the, the therapists is going to impart you see for example you know for me when I fly you know I fly very regularly…

I_01 : Ok so just a few final questions…

AF_0021 : Looked…

I_01 : Oh where did you go, this is a oh…

AF_0021 : What card is this?

I_01 : No this is a “P” flyer right?

AF_0021 : It’s solitaire you know?

I_01 : Yah so it’s a frequent flyer the thing quite a…

AF_0021 : Yah frequent flyer already…

I_01 : Normally you taken here…

AF_0021 : 13 years.

I_01 : Wow so you got a lot of points. [Interviewer laughed]

AF_0021 : Yah and I only fly first class.

I_01 : Oh really, on “O” Airlines?

AF_0021 : Yes.

I_01 : Ok you can get a lot of points then.

AF_0021 : Yah but I find my what I’m trying to tell you is this…

I_01 : Where did you go normally?

AF_0021 : All over the world.

I_01 : Wow that’s nice, very nice. So just now you mentioned that you said you won’t buy the these kind of product you know off the shelf or would is, is there any reason why you, you, you won’t think this is like a something that you will buy?

AF_0021 : No I have I have my own…

I_01 : I mean other than that let’s say you don’t have ok let’s say you don’t have a device for your for yourself and then let’s say like this one is off the shelf like either is part of your care or, or you want or separately in a shop you can by it off the shelf. So you know if let’s say you don’t have a blood pressure monitor would you want to would you to get thing oh wow “N” city… nice…

AF_0021 : Country “M”… this one go to where?

I_01 : “N” city is it?

AF_0021 : No this is “L” airport in “N” city, “L” airport…

I_01 : Country “U”.

AF_0021 : Country “U” so this one the latest…

I_01 : Oh all first class!

AF_0021 : Country “U” yah but what I’m trying to say is that you know I looked after myself very well even when I’m flying first class…

I_01 : Flying yah…

AF_0021 : And all that I exercise.

I_01 : Yah it’s very nice that’s good you take care of your health.

AF_0021 : Yah so no problem but if you about you know whether I, I need to buy one of these or not…

I_01 : Yah we want to ask you why, why you, you mentioned earlier that you said you won’t you actually won’t, won’t buy other than having your own device right let’s say you don’t have it will you go and get it would you buy it yourself ?

AF_0021 : No I’ll get a brand “M”.

I_01 : You’ll get brand “M”.

AF_0021 : I’ve never seen this brand before this is what made in made by Company “L’’.

I_01 : Company “L” yah but with the app because brand “M” the device is without the app so like if you have the app like additional thing you know like the app with video with messages, survey with…

AF_0021 : No I don’t think…

I_01 : That is tailored to…

AF_0021 : I don’t think I don’t think it will work because the important idea is this is just a, a, a mechanical measure…

I_01 : Yah a measure…

AF_0021 : Right?

I_01 : And also monitoring right?

AF_0021 : Yah it’s just monitor on the particular day right at that particular time you know this is the reading I get and that’s it you see so and then you know the usual, brand “M” has it also you know between, between this and this you know optimal you know? And then afterwards this is rated reasonable and then you have to be careful even when the, the pressure is high they will tell you, you know you got to go and see a doctor or you better go and checked with your doctor you know and have another reading you know. In another 15 minutes…

I_01 : Sure.

AF_0021 : Or whatever to make sure you know that this is not something you know that somebody called you, you know and, and, and, and scold you, you know then your blood pressure go up [Interviewer laughed] and then you take a measure no you see so…

I_01 : Yah you feel reassured, reassured that, that when you see all these readings you know you keep track of it does it give you a sense of like you know reassurance you will take…

AF_0021 : I will know I will know when I wake up I will know if ss- if I wake up right before I wake up right I’ll be in my bed I’m awake right with the TV on or not I will go through my cockpit drill you know everything working all ok before I get up. So that will already tell me whether I have my blood my blood pressure is high or not.

I_01 : Ok, ok, ok so you have your own way of doing things?

AF_0021 : Yah, yah, yah but I don’t know whether I think many people do but in their own way you know and…

I_01 : And this one does it help?

AF_0021 : These are the people that will live longer long longer than normal.

I_01 : But this device does it help your and in addition to all your own you know you have your own method right does it add, add on to it?

AF_0021 : A device that take my blood pressure on a regular basis will help to reassure myself you know that I feel I feel as good as what the…

I_01 : The, the…

AF_0021 : Ins- instrument tell me you see right? I have friends in country “X” you know they don’t they, they don’t they can’t pay for this, this device. And what they do was that when they go to the malls there will always be places where you know they will sell you, you can take your blood pressure you can take your blood sugar and all that for a small fee you know maybe 5000 “X” currencies. And these people will just go there and pay 5000 and then…

I_01 : And do it.

AF_0021 : Take a reading, yah.

I_01 : Ok so you also think that the cost is actually quite high like, like…

AF_0021 : The cost depends…

I_01 : Because you said that they cannot afford.

AF_0021 : You know the one I bought the brand “M” I can’t remember how much I you know…

I_01 : But do you think it will…

AF_0021 : I am I am rich enough…

I_01 : I know yah I understand but your country “X” friends you mentioned that you know…

AF_0021 : My country “X” friends that those people I know you know that I meet regularly they have no personal machine you know.

I_01 : That’s right that’s right.

AF_0021 : So they, they will you know when I’m with them we go to the mall you know in “N” city and then we come across this you know then they would just pay for it you know to be…

I_01 : What about like for Singaporeans other Singaporeans do you think that like…

AF_0021 : If, if, if this is a national initiative or aimed at older people you know the best way is not at the malls at community centres…

I_01 : Ok or hospitals is it?

AF_0021 : Or hospitals, hospitals may not be the best way but at community centres you know designated ones where the community centres the people you know instead of just getting you know National Day a watch you know they should also know on their finger tips where their elderly people are you know within the blocks ok?

I_01 : Ok so…

AF_0021 : The RC the RC leaders and all that and they are the ones who is going to give the feedback to the hospitals to say whether you know these things are useful or not right, see the feedback is not just me you know I stayed here I don’t know any many of my neighbours you know?

I_01 : Ok so feedback in the sense that like a kind of a community feedback like I mean the person who is using this device will also get feedback like in term in person you feel that…

AF_0021 : No more important is that whether it’s going to be something you know that is going to be at a not only age group or you know people suffering from certain deficiencies like AF or whatever and or people who are you know highlighted because of age group you know right? And whether the initiative can actually benefit them or not right because let’s for argument right these things if it is arrested you know let’s say high blood pressure it can lead to other problems you see right? So the resident is always for one of the shoe for one of the shoe you know the, the guy becomes limp right or for, for one of a better tire you know the journey is not made so question is whether government wants to spend the money and at what age you know should be the spending be done. So that they can monitor some of these is how do I put it you know probably the profile is from babies you know right they will they can tell you know and then they do another one maybe when they are in primary school and then later you know maybe their mid primary you know right? And see whether the school children you know can get the right kind of fitness for their age group right and whether those who are lethargic you know pale you know and always absent are being highlighted or not right? These are some of the things you know that the teachers you know must be aware right they talked about oh this guy you know one guy call Lee who is 19 years old and he died of…

I_01 : Yah correct yah the recent one right yah.

AF_0021 : Yah come on who do I blame if I’m I if I’m the parents right I blame myself because before he was in NS right he’s staying with you, you know?

I_01 : I know what you mean like the you have to be like the…

AF_0021 : So I said…

I_01 : Found out earlier.

AF_0021 : If I want if I want to be I use is this still on?

I_01 : Yah it’s still on but it’s ok doesn’t matter…

AF_0021 : What I…

I_01 : Go ahead yah.

AF_0021 : What I‘ll tell the parents that fuck you, you know [Interviewer laughed] you are the one responsible for your son’s death end of story…

I_01 : Ok, ok because they are ss- I mean there are all these other factors that come in…

AF_0021 : Looked at the fucking mother you know looked at the bloody fucking face what about their friends you know what do they say about these parents…

I_01 : Yah it’s, it’s tragic yah.

AF_0021 : Tragic so what you know happens everyday! [Interviewer laughed] Does it mean that the SAF the our guards are going to be less stringent?

I_01 : I mean yah because they are military you see cannot be… yah anyway the there’s one more thing ok so you finally you have any other comments any opinion about like how we can maybe improved on this or not add more features or in, in…

AF_0021 : I don’t know I don’t know I, I, I have never given much thought, thought to this problem because you know number one I’m, I’m not a sufferer I don’t think I suffer from it of course you know I’m, I’m very glad you know that Hospital “A” you know is paying so much attention to me. But you know some of the procedures which I go through right I pay for it you know and it can be very expensive but then you know because of my age you know and my pioneer status and all that a lot of the cost right has been absorbed by the government. But the issue is you know if everybody does this right I tell you our, our income tax you know must go up you know?

I_01 : Yup to pay for the cost of…

AF_0021 : It’s a question of you know…

I_01 : That’s right.

AF_0021 : You know whether we can actually afford it or not?

I_01 : Afford it that’s why they want to use this device to make it like more you know it can reduced the load in the in the hospitals.

AF_0021 : Yah maybe when the, the problem is discovered earlier you know so that remedial action can be taken but sometimes you know this cannot you know and then you got to go for surgery and this and that then it becomes a big thing you know?

I_01 : That’s right a big thing…

AF_0021 : Because of…

I_01 : It’s usually very expensive yah, yah, yah so anyway we just need you I just need you to help me to sign your name here and that’s about it we have ended the interview and…

AF_0021 : Sure.

I_01 : That’s the end of it thanks very much.

End of interview
