# Supplementary material for: Patient perspectives of the Self-management and Educational Technology tool for Atrial Fibrillation (SETAF): A mixed-methods study in Singapore
Source: PLoS One. 2022 Jan 21;17(1):e0262033. doi: 10.1371/journal.pone.0262033 (PMC8782297; doi:10.1371/journal.pone.0262033)
Supplement: S1 Data — (ZIP) [file pone.0262033.s006.zip › AFP2_IDI_0020.doc]

Participant ID number : AF_IDI_0020

Interviewer Number : I_01

Length of interview : 24.25mins

I_01 : Ok so thank you for taking part in this study so…

AF_0020 : No problem.

I_01 : The first question is how long have you been diagnosed with AF and how long how did you find out?

AF_0020 : 2014 I feel my heart my heart not so good and, and the heart like pumping very you know and how I say I scared I scared my heart you know then I breathe cannot good and I scared my heart pumping no good then I see doctor. I said I like that like that then…

I_01 : So…

AF_0020 : But I already got AF you know I don’t know I go see my own doctor not, not for not for AF…

I_01 : Ok for…

AF_0020 : For other thing then the doctor was pumped, pumped the doctor also no say anything I think you got your heart a bit no good on top like that one oh yah, yah I, I went to also go to this polyclinic annual check up then they said my I got AF then I said what is AF then they sent me there.

I_01 : Oh so you found out at the polyclinic one, ok.

AF_0020 : Then I said just I and you talk never mind if I got record the doctor one doctor I don’t know the name tell me get appointment all there you know ok then I come home then I feel not good but I dare not say so I come home I thought know you doctor very clever what make appointment I go what. Then the next then the night time the doctor called me you know mister I doctor from “C” then I said ok what happen you got this letter to hospital but if anything happen to you, you not comfortable quick go…

I_01 : So did you go?

AF_0020 : Then that night I scared already the more you scared the more you pumped right I said no, no, no then I quickly go already take a little then I go then I then from there I, I, I got medication already.

I_01 : Ok, ok so you took medication after that ok did they tell you anything else like go do you exercise you know or other things or not like even…

AF_0020 : No, no I all a- I a-…

I_01 : What you should eat all that?

AF_0020 : Yah got, but I along all along got exercise already.

I_01 : Ok, ok so you…

AF_0020 : Now you want me to run I also can run already. [Participant laughed]

I_01 : Ok so you do it regularly on your own a-, a- already .

AF_0020 : Now after taking medication I feel ok you know not said not said 100 percent ok but not, not so scared already die, die so I die, die also go and run, run and then hoping die, die. [Participant and interviewer laughed]

I_01 : Ok but, but it’s good exercise is very good for you is very good for you so did you get any other information or not where you get other information?

AF_0020 : Very simple then my son see me then doc- computer then paper all that you know then like that.

I_01 : So they talked to you your son and about…

AF_0020 : Then, then, then, then like that one.

I_01 : Ok, ok so, so you, you get your, your family members’ help ok, then ok so like during this 6 weeks of this study you know how, how do you do the how do you take your, your blood pressure…

AF_0020 : Yah I try…

I_01 : Everyday?

AF_0020 : Yah everyday…

I_01 : It’s everyday?

AF_0020 : I try punctual…

I_01 : Ok, ok what time a around what time?

AF_0020 : Example, example now 2 o’clock I start alright then at night 9 o’clock I start again another one so 2.

I_01 : 2 time per day ok.

AF_0020 : Because I must see not everyday same time what right sometime aft- night time before you sleep what happened right then not so I not happy know I said other the girl told me the your, your, your…

I_01 : Miss “M” yah.

AF_0020 : Girl told me one time can already I not satisfied so I did 2 time then I try again I said the last week or what I don’t know what I must checked also I, I try one time know wake up 6 o’clock or 5 o’clock the thing shoot very high you know of course…

I_01 : 6 o’clock in the morning?

AF_0020 : 130 like that after sleep like that one the doctor polyclinic also tell me no, no problem one then I then I try again morning 8 o’clock then the thing go down already like that.

I_01 : So how you feel when you when you see the very high how, how do you feel like you feel?

AF_0020 : Very high, good.

I_01 : You feel good because when it’s high you know?

AF_0020 : Yah but very low, no good.

I_01 : Yah, very low no good ok!

AF_0020 : I don’t like 97 one time 96 you know 97 at night then it frightened me you know your, your, your BP very low you know please see your d- see the I say what the jit bai [Means “once” in English] very low kio wo chow ke [Means “asked me to go to” in English] A and E siao [Means “crazy” in English] right?

I_01 : Yah so you, you know you know it don’t is not base on one time only right not base on one reading you take [Participant laughed] you, you know a few reading everyday.

AF_0020 : Jit bai juo jit a chow ke, siao [Means “just frightened once and you go, crazy” in English] right? [Interviewer laughed] then ok but I like 120, 126 high, high you said 130 you scared also but ok you know but it come down ok 120, 126 ok. But not ok very low 110 you know ok 100 ok, ok already not then come to very low very scared.

I_01 : Very low very then you get scared?

AF_0020 : Because too low what bo bo ko leng [Means “not possible” in English] jit a an chua [Means “ this one why” in English] very low maybe you when you tell people to do this you cannot said sit down no, no not said sit down you, you don’t do anything first. Like half an hour no, no normal then you pumped then normal right?

I_01 : So, so you prefer that when they when, when, when they she tell you the instruction…

AF_0020 : She said…

I_01 : Correct.

AF_0020 : Ni ni bu yao [Means “you don’t” in English] like very what must rest sit down half an but why I must sit down half an hour [Interviewer laughed] the then you rest wait, wait, wait then the BP down, down, down, down no, then not normal what right?

I_01 : So you so you find that if it’s too low also you get worried.

AF_0020 : Bo ko leng ma wo mm pat an ne [Means “Not possible I never experience this before” in English] mm pat a wo kia ma [Means “never before, I am afraid” in English] so wo jei diao jei [Means “sit down” in English] like make it very low like qi gong. [Means “a kind of breathing martial arts” in English] [Participant and interviewer laughed] zhen de. [Means “it’s real” in English]

I_01 : Hui, hui xia qu? [Means “it will go down” in English]

AF_0020 : Wo hui zuo qi gong [Means “I know qi gong” in English] that fellow will go down kao chap luck kao chap gor. [Means “96, 95” in English]

I_01 : Then you get worried then get worried so, so not too low you don’t want like you just say…

AF_0020 : Normal…

I_01 : Think normal, normal just take.

AF_0020 : Normal!

I_01 : Correct ok, ok, ok then you, you how do you do you think that this, this you know you can see the when you take…

AF_0020 : Reading?

I_01 : Reading many times right you can see the, the over a period of time how do you feel I mean like do you think it help it helps you or not?

AF_0020 : Yah actually ok…

I_01 : Help you?

AF_0020 : Very good what, happy you know…

I_01 : Happy yah.

AF_0020 : An ne bui [Means “Not so” in English] not very high not very really low ok never mind what like that right 110, 120 then go up a bit then go life like that up a bit…

I_01 : Yah, yah up and down yah.

AF_0020 : Not, not, not shoot very high very high ok.

I_01 : Ok then you got bring to see the doc- like you after that you go and see doctor you got bring this…

AF_0020 : Not yet not yet see poly even…

I_01 : Haven’t yet haven’t yet?

AF_0020 : That’s why I want to, how to get the print out?

I_01 : The oh ok you want the result right I’ll give you it’s ok I’ll, I’ll let me write down I…

AF_0020 : Because I can show the, the poly polyclinic doctor the pre- polyclinic doctor said what you know yah last time I have last time I have you know that time I not yet take this, this…

I_01 : This, this stu- this thing…

AF_0020 : No, not yet take this…

I_01 : Warfarin?

AF_0020 : Ah warfarin, I take these BP tablet then the then I checked wu si ho wu si pai [Means “sometimes good sometimes bad” in English] then we sometime I said no need I see the thing I scared already…

I_01 : Really [Participant and interviewer laughed] still high?

AF_0020 : Now, now after taking warfarin then after all the checking I find ok what the thing not very, very high…

I_01 : Yah not very high so, so it’s ok now?

AF_0020 : Ok then psychologically mein kiah liao lang kong lu a [Means “no need to be afraid of people saying” in English] my gu cah [Means “ex” in English] doctor boh [Means “not” in English] polyclinic ih kong [Means “he said” in English] he know I running. You running your BP very high you know, you running? Wo kong siao a si mi dai ji chao lah [Means “I said crazy what’s wrong just run” in English] then I see my BP now like normal…

I_01 : Normal.

AF_0020 : Wo chao lah [Means “I just run” in English] now chao… [Means “run” in English]

I_01 : So encourage you to exercise…

AF_0020 : Lu a chai bui bo dai ji [Means “you know nothing will happened” in English] you know mein kiah u dai ji lu kong ji a [Means “no need to be afraid” in English] go to hospital “N” si boh [Means “right” in English] then u si mi dai ji ka kin ga lu kong. [Means “anything happened they will tell you” in English]

I_01 : So…

AF_0020 : Jit bai bo yao kin an ni lok [Means “it ok if it go down once” in English] because tat bai lok, lok lok… [Means “if it go down all the time” in English]

I_01 : Tai di le, [Means “too low already” in English] ok then do you know that there are also like those mes- messages those popped up message…

AF_0020 : Ok u si… [Means “sometimes” in English]

I_01 : How, how do you find the popped up message [Participant laughed] those, those encourage you to…

AF_0020 : Bo, bo, bo [Means “no, no, no” in English] know sometime asked too much irritating…

I_01 : Iri- ok irritating.

AF_0020 : Like survey number one…

I_01 : Yah correct, correct.

AF_0020 : How are you, very well ok…

I_01 : [Interviewer laughed] the sur- so the survey is it too long is it too long?

AF_0020 : Initially I don’t understand…

I_01 : You don’t understand ok, ok.

AF_0020 : The…

I_01 : The language too difficult?

AF_0020 : The language jit tao kong lah lu u dai ji bo ji a le pai jit ko que cui kun u dai jib o mai kong [Means “direct to the point do you have any problems lately don’t talk” in English] the last 6 months and then kao jit chun an chua wo [Means “till now” in English] last 6 month si mi dai ji wo mana a chai [Means “I would not know what happened” in English]

I_01 : Ok, ok.

AF_0020 : Lu ai dai ji jit chun a ma [Means “you need to know what is happening now” in English] doctor ai chai jit chun bo ai chai [Means “wanted to know this period not” in English] 6 months tio bo? [Means “right” in English]

I_01 : So the question asked too long already ago.

AF_0020 : Mai kong luc go geh cheng kia jit lah cha jit lah tio bo sah jit lah tio bo u dai ji sah jit lah [Means “don’t talk about six months ago like today or yesterday or within these 3 days correct” in English]

I_01 : That one for the survey then the there are those message those na ge [Means “that” in English] message gu li ni hao xiang… [Means “encouraged you like” in English]

AF_0020 : Ke yi doa yi de. [Means “Can but just extra” in English]

I_01 : How is it can or not?

AF_0020 : Doa yi de. [Means “just extra” in English]

I_01 : You think too much tai doa [Means “too much” in English] ok.

AF_0020 : Dui wo lai jiang si doa yi de zai… [Means “to me it’s just extra” in English]

I_01 : Ni bu da xi huan? [Means “you don’t like it” in English]

AF_0020 : U lah u si kong [Means “yes sometimes they said” in English] very good very good ta tio u lah [Means “they said that” in English] but for me you very good also I know already what. [Participant laughed]

I_01 : [Interviewer laughed] Yah I know.

AF_0020 : I no good then no good right or not.

I_01 :So you already know so you don’t like you don’t like too much.

AF_0020 : If, if no, no if got problem you said your BP today is low for to this time please checked again say after a few days if no problem we will ah got problem we I call you like that mm si hoe lo. [Means “will be better” in English]

I_01 : Then you know that there are videos also right?

AF_0020 : Videos mai kua. [Means “don’t watch” in English]

I_01 : You didn’t you didn’t watched?

AF_0020 : I see ai ya chai chai liao mai pa kia wo [Means “I know already don’t frightened me” in English] you know don’t frightened me right?

I_01 : But the videos is to encourage you…

AF_0020 : Bo [Means “no” in English] I know I know yah, yah…

I_01 : You know like to give you some information give you some info…

AF_0020 : But as I said I can say the more we see these ming zi dao ni you zhe ge si qing [Means “you already know I have this problem ” in English] the more you see this the more you fed up right?

I_01 : So you feel fed up?

AF_0020 : So I, I no see I said I yah you know the thing go like that like that know already what for right or wrong actually even the TV got anything I, I don’t see already right.

I_01 : You don’t you don’t watched one?

AF_0020 : Even my friend said sometime got TV talk about this they don’t watched already they said what for we know already alright only for people don’t know.

I_01 : For info let’s say for info?

AF_0020 : Info is not ok but for me as I said I don’t see people don’t know…

I_01 : Ok, ok can.

AF_0020 : For me I don’t see I already what about right you know what I mean? [Participant and interviewer laughed] Joking!

I_01 : Ok then what yah ok don’t worry then also what about like the blood pressure monitor do you feel that is…

AF_0020 : No, no it’s ok it’s ok…

I_01 : It’s ok can you know taking it…

AF_0020 : Better wo u jit a liao [Means “I have one already” in English] I, I throw away already the old one I don’t know what brand what is the brand?

I_01 : You got buy a new one brand “O” is it brand “O” yah, yah.

AF_0020 : Brand “O” brand “O” bo, bo, bo [Means “no, no , no” in English] this one old one…

I_01 : Yah the old one brand “O”.

AF_0020 : The old brand “O” but I want the cuff, cuff type an ni tup diao [Means “just connect it like that” in English] you see.

.I_01 : Like the one the, the one in that you used for the study one.

AF_0020 : This one this one that one strapped this one good you see you see mine an ni tup diao. [Means “just connect it like that” in English]

I_01 : So, so like…

AF_0020 : So it tup dio huh [Means “got connected” in English] then you like that you see you see that?

I_01 : Ok, ok can I’ll write this down so you prefer some like a…

AF_0020 : Cuffed this is called cuffed…

I_01 : Can cuffed ok but…

AF_0020 : C. u. f. f.

I_01 : Yah, yah [Interviewer laughed] blood pressure monitor with…

AF_0020 : Belt, belt…

I_01 : Strap prefer..

AF_0020 : Strap.

I_01 : Cuffed type cuffed type.

AF_0020 : The strap mm si kong bei sai a sai lah [Means “not cannot, can” in English] but…

I_01 : How you find the strap this one the one for the study one how you find it?

AF_0020 : Ok, no problem…

I_01 : It’s ok no problem.

AF_0020 : But I, I when I put in I strapped, strapped.

I_01 : Because nowadays the newer version all something like that…

AF_0020 : Never mind jing cai. [Means “whatever” in English]

I_01 : But you prefer this one you prefer…

AF_0020 : No I just say only u jiu u [Means “if you have is ok” in English] I suggest only what right because some people like very troublesome they said aiya kum yuang hai yao kum [Means “like that and need to do that” in English] like that like that an ni kuang [Means “like that” in English] strapped, strapped you know what I mean jit a jiu [Means “only one hand” in English] I one, one people do right if got people help you don’t got people help you easy.

I_01 : Ok so like you know when Miss “M” came and she give you instruction is it helpful or not for you?

AF_0020 : Can I already understand already what.

I_01 : You already understand so also no problem that one.

AF_0020 : No problem one.

I_01 : Is it too long she, she or too short?

AF_0020 : No she, she ok one…

I_01 : Or too short?

AF_0020 : She’s scared I don’t understand you know she ok like, like, like…

I_01 : So diff- so easy or difficult?

AF_0020 : You want to no, no, no, no very ok very easy like you scared I don’t know right [Interviewer laughed] so you scared only what it’s you not me you know I know I said I got used you just said pressed, pressed ok I can used right no, no problem she ok she ok.

I_01 : Ok so you know there’s also like a number right the tablet got also got number you know the on the on the tablet got a number you got try calling or not, never?

AF_0020 : She said she said got trouble problem call I no problem what.

I_01 : So don’t have anything?

AF_0020 : No problem no call want to talk to who?

I_01 : You think the number can help?

AF_0020 : I don’t know, know I never tried what?

I_01 : Never tried but let’s say let’s say you might need it to make like if you used…

AF_0020 : Maybe in case…

I_01 : In case yah.

AF_0020 : I just say only I just say in case because this one connect to there right?

I_01 : Correct, correct yah.

AF_0020 : If he called they know right?

I_01 : Yah, yah they know.

AF_0020 : Then they can dispatched sorry dispatched…

I_01 : Yah to you…

AF_0020 : Am- ambulance… [Participant and interviewer laughed]

I_01 : No maybe nurse, nurse how nurse maybe nurse as well.

AF_0020 : No you know what I mean not you, you know what I mean now?

I_01 : I know I know what you mean yah.

AF_0020 : Because emergency what, right?

I_01 : Yah so you feel that one very helpful?

AF_0020 : If, if supposed since this connected then they also know right?

I_01 : Yup yah so this connection with…

AF_0020 : If that’s why I said this one cannot call right if they can like some see something like the reading not so good example suppose many time reading no good then they…

I_01 : Yah they called you check?

AF_0020 : No, no, no the tablet can, can, can called then we can talk you see then they said hello hoe boh, hoe [Means “are you good” in English] [Interviewer laughed] if no hoe [Means “good” in English] then they said ok I call ambulance.

I_01 : Ok, ok, ok I get it yah.

AF_0020 : Because call ambulance they call, we call very difficult you know the ambulance will ask you where, where, where you know if this can communication then better you know...

I_01 : Ok can.

AF_0020 : If this tablet got communication ring, ring hello, hello because we are pumping right?

I_01 : Yah, yah so that one you feel will be much more helpful will ok, ok, ok can we take note of it ok then about the this tablet…

AF_0020 : Because wait, wait because if no connection no communication purpose bo eng [Means “useless” in English] also what right you using you only know my data what…

I_01 : Only your data yah correct, correct yah.

AF_0020 : The purpose it’s there example like signal this one serious case they looked ok they send also must have some signal to them like this now the result very dangerous…

I_01 : Yah dangerous yah, yah.

AF_0020 : Then that fellow will call and say please checked again another one hour you know what I mean or not and if you are not feeling well then what happened you know must help people also right?

I_01 : So actually they, they we want to you know they want to hear from what you, you all think about this then we can improved yah.

AF_0020 : Yah, yah what I say is like this if you can, can better this one better.

I_01 : Yah make it better yah, yah…

AF_0020 : No just collect data for what?

I_01 : Correct, correct yah.

AF_0020 : I can collect data I can show my polyclinic what I just say sorry…

I_01 : I know I know it’s ok.

AF_0020 : I buy one brand “O” [Interviewer laughed] and collect, collect, collect. [Participant laughed]

I_01 : But they have your number also so, so maybe they can call you.

AF_0020 : I say…

I_01 : Yah, yah can, can…

AF_0020 : Ok, ok can, can, can.

I_01 : Ok, ok then like what about like you ok you find the tablet how’s the size?

AF_0020 : Ok can…

I_01 : Is it the thing…

AF_0020 : This is normal right?

I_01 : Is that too small?

AF_0020 : No just nice.

I_01 : Ok, ok just nice ok.

AF_0020 : Just nice just right.

I_01 : Then what about like the, the, the word there too…

AF_0020 : Ok, ok no problem no problem yah no problem.

I_01 : No problem with that ok then what about the brightness, brightness also ok.

AF_0020 : All ok.

I_01 : So let’s say if we have ok we have tablet we have phone we have computer right you will prefer to use which one we have tablets this one…

AF_0020 : Tablet better because tablet is there put on table together with thing like that, that like that I, I left it on the table like that one you know morning I left it on the table you want just opened up the, the…

I_01 : The strap yah.

AF_0020 : Then I just take the strap wrapped checked ok put it back I, I like that leave it one corner…

I_01 : Leave it one corner ok you prefer ok what about like if but if phone, phone smaller and…

AF_0020 : No phone don’t want.

I_01 : Don’t want don’t want ok…

AF_0020 : Phone don’t want.

I_01 : Ok, ok then like computer big one?

AF_0020 : No, no, no, no. [Participant and interviewer laughed]

I_01 : Don’t want ok, ok.

AF_0020 : Ding teh. [Means “occupied space” in English]

I_01 : Ding teh [Means “occupied space” in English] ok, ok then what about…

AF_0020 : Just tablet just nice.

I_01 : Ok, ok then also like one of the one of the question because some people like paper form if we give you paper form you like would you like it so that, that means…

AF_0020 : I know I know you record myself.

I_01 : You record, you sent to us after that you sent one…

AF_0020 : No need you just pumped clicked pumped already close already paper leceh, [Means “troublesome” in English] must have a pen there a book there or what I, I did that last time.

I_01 : Some people prefer paper.

AF_0020 : No, no I, I last time like that then sometime very lazy also [Participant laughed] already pumped difficult one thing pumped very then must record because must record must take note very fast after if no fast they just…

I_01 : Correct, correct.

AF_0020 : Just switched off already you know automatically like that.

I_01 : Yah, yah so you, you ok with the current one ok, ok.

AF_0020 : Yah, yah very happy.

I_01 : Ok, ok can then let’s say ok let’s say we have this device you, you when you found you when do you prefer we us like I said we introduced this device to you, you know you prefer like when you first got the AF…

AF_0020 : Of course no, no, no cannot have…

I_01 : Or like in the middle or on the at the end while like a…

AF_0020 : Until the person are stable you know…

I_01 : Ok stable.

AF_0020 : Psychologically you know after go hospital for after what you called you first got AF can also the person may get scared also you know every time pumped, pumped, pumped unstable like I already from one 4 I mean September one 4 one 5 one 6 one 7 now ok. 3, 2 or 3 years later you all tracked this person is stable already understand including medication understand already ok then you can start monitor like that.

I_01 : Ok so must be you feel must be stable first the condition you know may not be at the beginning…

AF_0020 : Bo a pa kia lang ma [Means “if not you scared people” in English] then the fellow keep on pumping you know then the, the blood pressure also go up… [Participant and interviewer laughed]

I_01 : Yah, yah it might caused it to be, be a bit worse right ok so like let’s say you, you think about other your, your friends your the people around you let’s say you, you we, we introduce…

AF_0020 : People also got AF I asked them to take this thing to do this thing, recommend?

I_01 : You ok yah so like do you think like they will pay for this or not you know…

AF_0020 : You see…

I_01 : If let’s say I will be introduced to them?

AF_0020 : That is why I think the patient initial one starting one they kia si [Means “afraid to die” in English] they will do everything even you say one, one hundred fifty or what they will still pay because they, they know you this one go to hospital one. They happy because hospital so call controlled the, understand there right…

I_01 : Right.

AF_0020 : They any amount they may pay right 200 also they pay right?

I_01 : For hospital side?

AF_0020 : No, no I mean I mean I’m a patient…

I_01 : Yah correct at, at the hospital…

AF_0020 : At the hospital if starting you said mister you need to monitor these you know you buy this my nurse will tell you the nurse said this one cost will be 250 you scared right you first time you scared you, you pay right. Then you said oh my house has that you don’t buy already right but then no monitor from here to there…

I_01 : Yah no monitor correct yah so let’s say if it’s not hospital one if it’s like let’s say pharmacy or…

AF_0020 : Outside that’s why if outside very difficult also…

I_01 : Difficult more difficult.

AF_0020 : Unless the person scared.

I_01 : Ok, ok so…

AF_0020 : But it’s better the hospital the health clinic I mean heart clinic and all that you see say you are now in this case after a person as I said stable already recommend this fellow like that.

I_01 : Ok so better at the hospital ok, ok then…

AF_0020 : Because you, you, you, you go brand ”O” company they don’t care one what you all so expensive 300 [Interviewer laughed] mai buay lah hor [Means “don’t but it” in English] really you know I buy this brand “O”…

I_01 : How much you got this?

AF_0020 : I nearly don’t want to buy I forgotten already 5 years already not 5 years...

I_01 : But very long you used very long.

AF_0020 : 10 years I think.

I_01 : But, but when you used this one you cannot track did you did you record down or not?

AF_0020 : I record!

I_01 : You record ok, ok that’s, that’s very good.

AF_0020 : I record!

I_01 : Very good yah very good.

AF_0020 : Very leceh [Means “troublesome” in English] [Interviewer laughed] no doubt can take the machine there…

I_01 : Yah correct, correct.

AF_0020 : The doctor will start pressing but no good we want to see because let the doctor see you see then the doctor circled very high how many high and all that.

I_01 : Yah ok so let’s say if we got you got any ideas likes we can improved you [Participant laughed] or kind of other things that you want to see or not because you know you used it for 6 weeks…

AF_0020 : Yah, yah I know what you mean yah.

I_01 : Yah so like you, you know any other features…

AF_0020 : No I don’t know I don’t know about…

I_01 : With the app to this one…

AF_0020 : About what…

I_01 : This device?

AF_0020 : But I think so far so good already understand if you want I’m I cannot recommend more unless the per- the patient is very, very chao kao [Means “in bad condition” in English] like that then you got a better complicated one you know but as I said I’m ok so I think this one got communication with hospital is more than enough.

I_01 : More than enough ok, ok but maybe have a bit of like mobile found the phone call the communications the introduction…

AF_0020 : Because l say at present I have not much complication so far so good that is all.

I_01 : So it’s ok.

AF_0020 : Ok? [Participant laughed]

I_01 : Ok alright ok so I think that’s about it we will stopped the…

AF_0020 : Yah good, good, good.

I_01 : Let me stopped the recording. [Participant laughed]

End of interview
