# Supplementary material for: Patient perspectives of the Self-management and Educational Technology tool for Atrial Fibrillation (SETAF): A mixed-methods study in Singapore
Source: PLoS One. 2022 Jan 21;17(1):e0262033. doi: 10.1371/journal.pone.0262033 (PMC8782297; doi:10.1371/journal.pone.0262033)
Supplement: S1 Data — (ZIP) [file pone.0262033.s006.zip › AFP2_IDI_0019.doc]

Participant ID number : AF_IDI_0019

Interviewer Number : I_01

Length of interview : 15.45mins

I_01 : Ok so thank you for taking part in this study so how long have you been diagnosed with atrial fibrillation and how, how have you how did you find out about it?

AF_0019 : I think 3 years already

I_01 : 3 years ok so how did you find out about it?

AF_0019 : Because 2 years 3 years ago I had 2 stroke so from there they went to do further testing then they found out.

I_01 : Ok so do you take any medication?

AF_0019 : Yah a lot.

I_01 : Ok a lot a lot of medication is it for the AF or for other the other thing you are talking about.

AF_0019 : Everything.

I_01 : Everything!

AF_0019 : I can show you this is the PM, AM.

I_01 : Ok so for everyday.

AF_0019 : Yah everyday and then I’m also a diabetic so I have my insulin jab these are the medicine I’m taking.

I_01 : Ok, ok so it’s all combination ok then other than these that you found what kind of other information the doctor gave, gave you or told you to do other than taking medication?

AF_0019 : They did mentioned because my vessel in my heart and in my brain is too thin too thin cannot do any operation too dangerous so they do not suggest to operate in fact they wanted to do a bypass but because my blood vessel was too thin so they don’t want to take the risk so anyway med- taking medicine is helping me to heal so that’s why they continue to ask me to take medicine, yah.

I_01 : What about like diet advice like exercise you know do you get any information from them?

AF_0019 : I can’t exercise because if I walked too far or too fast I get a lot of breathlessness so I only maybe I won’t say exercise just basically I’m walking that’s all yah.

I_01 : When you walk walking around here how long did you walk?

AF_0019 : Here I’m, I’m not I’m I mean basically is driving.

I_01 : Ok driving ok.

AF_0019 : Driving I have no problem yah.

I_01 : What about diet, diet advice?

AF_0019 : Diet wise I avoid all the greens because I’m taking warfarin so I am afraid because I if I take green then I have to calculate my vitamin K which is for me is very tedious so just to so called do not want to get involve in this tediousness [Participant laughed] I just avoid all the greens, yah. Spinach, broccoli which yah so I only take pak choi. [Means “Chinese cabbage” in English]

I_01 : Ok pak choi [Means “Chinese cabbage” in English] yah ok a lot of veg- green vegetables…

AF_0019 : I can’t take green vegetables spinach all these I cannot take.

I_01 : You cannot so you avoid green…

AF_0019 : Yah because that one is very high in vitamin K.

I_01 : Ok, ok so avoid green some of the green vegetables ok other than that right did your friends and family give you any advice did you did you just…

AF_0019 : Oh yah I’m taking a mixed of brown and white rice yah I don’t take pure white, white rice yah avoid I try to avoid pure white rice and I’m controlling my diet, yah.

I_01 : Ok, ok any other sources of information like say via the…

AF_0019 : Yah because I’m also limited on drinks I can only take one litre of water one and a half litre the most a day, yah.

I_01 : Ok, ok then do you get information from like internet, TV, newspapers, books…

AF_0019 : Seldom, seldom.

I_01 : No not at all not at all seldom means not at all or very rarely?

AF_0019 : Very rarely.

I_01 : Very rarely!

AF_0019 : Yah once a while I just look up, yah.

I_01 : Ok, ok then ok so during these 6 weeks of this study like what was the sequence that you how do you go about it to, to take your blood pressure and…

AF_0019 : Because I drive morning from morning 6 o’clock to about 10 o’clock in the morning so I come back and rest until about 4 o’clock so by lunch time then I do my blood pressure.

I_01 : Ok usually in the noon during noon time.

AF_0019 : Yah when I have enough rest I’ll do a blood pressure.

I_01 : Ok and what was the how do you feel when you when you when you take it like do you have any feelings?

AF_0019 : Ok so far so far the result has been very positive.

I_01 : Ok, ok actually when we were looking at I mean I we have also we, we are able too see also…

AF_0019 : Yah monitor…

I_01 : Yah so actually there was quite a bit of a gap is, is there a reasoning for that?

AF_0019 : Yah because I was admitted to hospital.

I_01 : Oh ok just now you ok that was how long was the, the…

AF_0019 : I was admitted to hospital for 2 weeks.

I_01 : Ok, ok admitted for 2 weeks at hos-…

AF_0019 : Because my leg got swollen so they have to admit me and used antibiotics for…

I_01 : For leg?

AF_0019 : To treat me.

I_01 : For swollen leg.

AF_0019 : Yah and also my testicles enlarged.

I_01 : Yah, yah ok, ok, ok so actually during these time period you didn’t bring the thing there you didn’t bring…

AF_0019 : No, no, no the hospital told me not to they will do my blood pressure testing one day like maybe 3 times.

I_01 : Ok so hospital told not to you were told not to bring, bring monitor ok, ok, ok so actually that’s the reason why there was such a big gap ok.

AF_0019 : Yes.

I_01 : But other than that right how, how do you feel about using this were there any what was the easiest parts of or aspect of this tablet app that you felt your…

AF_0019 : There’s nothing difficult about it…

I_01 : Is that can you…

AF_0019 : Very easy, yah.

I_01 : Ok, ok so like ok.

AF_0019 : Very straight forward.

I_01 : Overall very you find it very easy…

AF_0019 : Yah very easy.

I_01 : Straight forward ok then what about like did you watch the videos or not?

AF_0019 : Some of them.

I_01 : Ok how were you find the videos any favorites?

AF_0019 : They just explained to me what is what happens to me and basically what can I do about it that it.

I_01 : Any thing else you want to see in the videos like more…

AF_0019 : [Participant laughed] No, for me is a matter of information gathering that’s all, yah.

I_01 : Ok was it too long the videos?

AF_0019 : No ok, yah not too long not 3 hours 4 hours ok. [Participant laughed]

I_01 : [Interviewer laughed] Did you watch the there’s also the cooking one the diet…

AF_0019 : Cooking one I didn’t watched.

I_01 : Ok, ok the diet why, why you didn’t watched the cooking one?

AF_0019 : I think I missed it.

I_01 : Ok, or not really interested?

AF_0019 : No, no, no.

I_01 : Ok, not so interested ok then the diet one?

AF_0019 : Yah because the thing is, is very difficult because nobody is at home then if I go out and work usually I eat out and then very seldom eat at home.

I_01 : So not very relevant is it?

AF_0019 : So when I eat out…

I_01 : So…

AF_0019 : I try to choose to eat simply more soup base food.

I_01 : Healthy, ok then what about like the this one on the AF the information one do you how do you find that one the video?

AF_0019 : Ok informative.

I_01 : Informative, ok do you think that it will be useful for you in any way?

AF_0019 : Yah, yah at least I know what is happening to me yah.

I_01 : Ok do you feel that the video change your behavior in any way or not?

AF_0019 : I think no because I’ve been very I followed instruction of the doctor and the hospital should be so, so far so good.

I_01 : Ok, ok so you already quite disciplined already in terms of like following the doctor advice…

AF_0019 : Yah I won’t say disciplined [Interviewer and participant laughed] I tried to, to be as strict as possible.

I_01 : I maybe that one too strong too strong word yah.

AF_0019 : Yah I tried to follow as strict as possible.

I_01 : Ok, just try ok, ok then what about like the popped up messages you know some encouraging messages that came about you know how do you find it?

AF_0019 : Ok that one ok [Participant laughed] ok.

I_01 : That’s ok?

AF_0019 : Yah very good.

I_01 : Do you have any feelings that come about when you when you look at like look at them or not it’s like…

AF_0019 : It encourages you that’s all yah.

I_01 : So it does ok, ok do you find that it was coming out a bit too much in…

AF_0019 : Ok.

I_01 : Ok, then what about like the, the survey you did you do the surveys that were sent to you?

AF_0019 : Yah, yah I did all the survey.

I_01 : You did it ok, so how do you find the surveys?

AF_0019 : Simple very simple.

I_01 : Very simple ok, ok were it too long like these…

AF_0019 : No, no, no, no I’m ok with it.

I_01 : It’s ok, the language was that very diff-

AF_0019 : Ok very, very simple.

I_01 : Ok then ok so we talk a little bit about the interface of the tablet was the screen too small too big, average you know how do you find it?

AF_0019 : Ok nothing wrong.

I_01 : Nothing ok.

AF_0019 : Normal, normal, normal.

I_01 : It’s normal, ok let’s say we have a we have a phone we have a tablet we have a desk top computer or an other, other kinds of electronic device which one will you prefer?

AF_0019 : I think for home, tablet.

I_01 : Tablet ok…

AF_0019 : Yah tablet…

I_01 : Even, even in your home?

AF_0019 : Yah tablet is ok because phone unless you want to carry around which I don’t need to so tablet I just leave at home as and when I charged it and used it that’s it.

I_01 : Why would you not want to carry it around?

AF_0019 : No point [Interviewer laughed] after get lost get damaged worst. [Participant laughed]

I_01 : Ok so you’re…

AF_0019 : I only do it once awhile once a year, day you see yah I just do the measurement that’s it.

I_01 : Ok so you, you don’t…

AF_0019 : I don’t touched the tablet anymore yah.

I_01 : Ok just once a day enough ok, ok let’s say we have a paper form like paper meaning that you write down your you record down you own manually and then you sent it to us ok then we sent you some brochures for information would you like it in this form or electronic form better?

AF_0019 : Electronic form.

I_01 : Ok any reason why electronic?

AF_0019 : Paper, changes are I’ll missed it because it’s very leceh. [Means “troublesome” in English]

I_01 : Very leceh. [Means “troublesome” in English] ok and also may lose, misplaced…

AF_0019 : Yah then you don’t know where you put you go and look for it, don’t want [Interviewer laughed] electronic device you just take out do put it and then sent by itself I don’t have to border.

I_01 : Yah so you like you do like the convenience and all that.

AF_0019 : Yah, yah, yah it’s convenient.

I_01 : You preferred that.

AF_0019 : Yes of course.

I_01 : Ok then what about like let’s say ok how about do you find the, the font how do you find it, the font?

AF_0019 : Ok, yah it’s what you call this you don’t have special font for it so it’s ok yah it’s just a normal font it’s ok.

I_01 : Then the brightness?

AF_0019 : Ok, ok.

I_01 : It’s also ok, ok then would you want to see like any other features that we can be add on to this you know…

AF_0019 : I don’t think you need any fancy features this is a basically a medical survey in the way so I think just leave it as it is, it’s ok good enough, yah.

I_01 : And you mentioned also because you have other condition right you feel that if let’s say we have a tablet and an app that can also add more…

AF_0019 : Can add on yah that will be good that will be good…

I_01 : You, you would like to see that?

AF_0019 : Yah that will be good that will be good maybe you can add on diabetic all these can why not?

I_01 : So you, you would like see that?

AF_0019 : Yah, yah why not why not why not?

I_01 : Ok, ok, ok then what other things that you will like to I mean like you, you have any ideas that you feel you can you know gives us on in terms of like improving this experience of yours?

AF_0019 : I think no this is basically very convenient and it’s already very easy yah I mean I don’t have to border to even to send because it by itself it yah.

I_01 : Yah automatic one.

AF_0019 : Automatic one so I think compared to what I’m using for my heart my heart if I go for my heart appointment I have to send the information in the morning before I go for my appointment.

I_01 : So that one is you have to press the send button also right?

AF_0019 : Yah, yah, yah.

I_01 : How do you do it you keyed in manually is it?

AF_0019 : They have a procedure which I follow…

I_01 : Ok, ok and that one is…

AF_0019 : So the information for the last one month to be sent because it’s by the side of my bed and I have yah, yah…

I_01 : Yah like a…

AF_0019 : I have a what you called this…

I_01 : Pacer?

AF_0019 : It’s not a pacer it’s called a shocker so if I have a heart attack this will send a shock signal to my heart to wake it up.

I_01 : Automatic one.

AF_0019 : Yah so any, any every night they will I mean they connect and then they will monitor but the information is like if I have to then I if unless they download from here if they have to if not then I’ll sent every time I go for appointment.

I_01 : Ok, ok then you have you know when Miss “M” came to give you the device right how do you find the instruction that she, she explained to you?

AF_0019 : Ok very straight forward.

I_01 : Ok let’s say if she didn’t give you any instruction and advice or, or you know how to use it right will you think that you will be able to do it on your own?

AF_0019 : I’m I will figured it out but it’ll take a while.

I_01 : You, you will take a awhile?

AF_0019 : Yah maybe I will call you guys and asked ok, step one step 2 step 3 yah.

I_01 : Ok so you will…

AF_0019 : Ok she gave me very clear instruction what to do ok yah good.

I_01 : Then what about do you know that there was a, a, number right you know a number on the, the tablet on the side the side on the below do you or you aware of it?

AF_0019 : No.

I_01 : Ok Miss “M” didn’t tell you about it?

AF_0019 : No, no, no.

I_01 : Ok because there was a num- this the number is here this num-…

AF_0019 : Maybe she tell me I, I forget…

I_01 : Number that you can call out of this…

AF_0019 : Phone number?

I_01 : Yah the phone number phone number.

AF_0019 : Yah, yah if I have any problem I can call the phone number yes.

I_01 : Did you try calling it or not?

AF_0019 : No need no need because it’s no necessary [Participant laughed] no necessary yah.

I_01 : Ok, ok do you find that it was helpful to have the number there?

AF_0019 : Yah, yah at least on anything I can always call on the spot yah.

I_01 : Ok then ok let’s say we try to introduce this device like when you were when do you think is the best time to introduce it to, to you like ok when you first had the diagnosis of your like say AF and other conditions or in the middle or later on a bit?

AF_0019 : I think in the middle at least you get your condition then you treat your condition then this is like a extra help yah for me is in the middle.

I_01 : Yah why not at the beginning?

AF_0019 : Too many things in your hand then you get lost chances are you messed it up.

I_01 : Ok you so you, you, you think that there will be too many things to manage.

AF_0019 : Yah so when in the middle you already settle the initial part then you know what is going on what you need to do and then you just add on, ok.

I_01 : Ok so middle will be better, ok then a few more questions…

AF_0019 : Sure.

I_01 : Final questions would you I mean what other what do you feel about like some using a electronic device like you know because you have all these conditions you have to manage do you feel like it’s like a bit tedious troublesome you know…

AF_0019 : No, no…

I_01 : Electronic form…

AF_0019 : I don’t have a lot of electronic device I so it’s ok…

I_01 : Do you ok.

AF_0019 : Anyway today technology is you better used a electronic device because that is a tool to help you.

I_01 : Because you also have to man- you see you have to monitor all these other things that you have right…

AF_0019 : No it’s ok.

I_01 : Do you think that it’s like additional burden for you?

AF_0019 : It’s ok I don’t have a lot of things to monitor. [Participant and interviewer laughed]

I_01 : Ok, ok so it’s it you don’t mean you don’t mind something like that?

AF_0019 : Yah, yah it’s ok.

I_01 : Ok, ok, ok, ok so I think that’s all about it for the for the interview thanks very thank you very much…

AF_0019 : Thanks, thanks a lot.

I_01 : I’ll stopped the recording.

AF_0019 : Thank you for…

End of interview
