# Supplementary material for: Patient perspectives of the Self-management and Educational Technology tool for Atrial Fibrillation (SETAF): A mixed-methods study in Singapore
Source: PLoS One. 2022 Jan 21;17(1):e0262033. doi: 10.1371/journal.pone.0262033 (PMC8782297; doi:10.1371/journal.pone.0262033)
Supplement: S1 Data — (ZIP) [file pone.0262033.s006.zip › AFP2_IDI_0017.doc]

Participant ID number : AFP2_0017

Interviewer Number : I_01

Length of interview : 35.46 mins

I_01 : Ok so thank you for taking part in this study and helping us to do the blood pressure over a period of time so the for how long have you been diagnosed with atrial fibrillation the irregular heartbeat and how did you find out about it?

AFP2_0017 : I’m irregular heartbeat is about actually I don’t know how many exactly how many years but it’s quite long until I, I have my, my by-passed 2 at December 2015…

I_01 : 2015 had by-passed, ok.

AF_0017 : But after that still not cure not fully cure then I have done the balloon…

I_01 : Balloon, stands…

AF_0017 : Balloon last year, October 2017.

I_01 : Ok, ok so you have other heart like other conditions?

AF_0017 : Heart, heart problems.

I_01 : Heart, heart problems ok so like were there any medications that you take?

AF_0017 : Yah I have I take the diabet- dia- diabetes medicine blood pressure, cholesterol all these type of medicine and the, the heartbeat one I took to manage the heartbeat.

I_01 : Ok then what other sources of information right you know like how to manage all these conditions and also your, your other your irregular heartbeat other than the doctor who gave you some advice outside of the hospital were there any?

AF_0017 : Oh I I’m, I’m doing I’m doing some reading in internet how to manage cholesterol how to manage diabetes how to manage the, the blood pressure and what things to eat what’s supplement or herbs that can cure can man- can, can I mean not, not really cure but can, can, can make the, the body better, feel better.

I_01 : Ok, ok then including the, the atrial fibrillation the, the irregular heartbeat also?

AF_0017 : Yah, yah actually when we are healthier definitely that one because that things because of the cholesterol you see if everything is ok automatically the, the, the things is cure I think, it should be that, that way.

I_01 : So you think is I mean is it’s the cholesterol that, that, that levels that really affect it, you feel?

AF_0017 : Yah actually from my knowledge from my read on the in the internet actually we really we must have enough oxygen there is 5 things we need, oxygen the first thing we must make sure our body enough with oxygen. How to make to, to make sure the thing enough actually we must do some exercise to create the oxygen in the body, then the things second thing is the water we drink should be filter our normal water tap water is with the chlorine with a lot of things metal which is not good for our body actually. Number 3 is the, the, the food the food actually is we must eat the alkaline more alkaline food than the acidic food this is my understanding. The fourth one is the our mentality our mental should not be stressed should be always in the calm condition than our body will… will be better, number 5 all these things after we manage then our when we check our body in the alkaline condition is not in the acidic more acidic. That’s mean our food is very important…

I_01 : Food!

AF_0017 : Make sure we eat correct food.

I_01 : Ok correct mean food means what?

AF_0017 : Correct means must if you go internet you must you, you have the al- acidic food and alkaline food you smust you must eat more alkaline food alkaline food like the vegetarian the vege- vegetables…

I_01 : Yah vegetables…

AF_0017 : And like acidic food like meat all these even the smoke [Participant laughed] smoking and the alcohol all these acidic for example only.

I_01 : Ok, ok then are there any people whom you go to for information like your friends, family, colleagues or normally you…

AF_0017 : Normally no it’s normally…

I_01 : For advice?

AF_0017 : We receive no advice all I get from the internet a lot…

I_01 : What, what website you go to what kind of like website?

AF_0017 : Know you, you the, the, the health food that one the health, health website.

I_01 : Internet?

AF_0017 : You, you, you can just read about the, the doctor opinion whether true or not true it’s another thing [Participant laughed] but some is there are they said it’s proven like some herbal or whatever or vitamin because when we grow old we not produce our own things you see?

I_01 : Yup.

AFP2_0017 : Then these thing we need that’s why we need a supplement but without money we can’t we, we take we, we cannot buy all these things that’s the problem, if we have that type of problem the only thing we when we eat then we must control and we can do we can get the free oxygen by doing the exercise. And water we can’t have if we don’t have mon- money to buy the, the filter machine then the water filter machine we cannot buy then we just take the, the warmth water.

I_01 : Ok yah so do you do any exercise or not on your own?

AF_0017 : Actually at the moment my exercise only just walking only.

I_01 : Where?

AF_0017 : Am I normally the, the JE that one I just walk from the JE the I building because normally I with our friend all same age one we walk from the I building go to BB go one round few [Participant laughed] that one a few kilometres already everyday is like that.

I_01 : Everyday, everyday you walk?

AF_0017 : Everyday walk like that.

I_01 : Then ok then the cooking one right you, you normally you cook or…

AF_0017 : No, no we didn’t cook I didn’t cook because I stay in my, my, my, my sister house whatever she cook then I eat that only thing we must should not eat so much like the rice that we know if, if possible can eat just one time one day is good enough already. The, the because the quantity of food is play a part on my on my this, this thing know my chest because I take too much food then my, my chest not feel comfortable not very good.

I_01 : Ok, ok whenever you eat too much?

AF_0017 : Too much that’s why I must the quantity must reduce can eat more but spread the, the, the type the, the, the, the period like every time you eat just maybe a little bit a little bit a little bit you can eat maybe 3, 4 time but a little bit a little bit not so much.

I_01 : A little bit.

AF_0017 : That type the and the sweet one also must control the sweet things if too much sweet also my chest not feel comfortable that’s why.

I_01 : So you try not to eat too much.

AF_0017 : The, the quantity must reduce.

I_01 : Ok, ok then you know this 6 weeks of the study what was that how do you use the device on a average day what do you do first you know what, what time do you take the you know the average…

AF_0017 : You told you talk about this device is it?

I_01 : Yah the device, yah.

AF_0017 : This device I take this one then I on then I enter the code after that this the blood pressure I takes beside this one then I put on the hand the, the, the , the what you call it I don’t know. You put on your hand then, then I press the button then I just relax wait until the thing measure until finish get the number here then I wait a while then after that I open this one then I press the on the tab- on the tablet and then that thing come out.

I_01 : Ok so you take the you said that you take in the morning…

AF_0017 : When before, before fasting I take in the morning…

I_01 : Before you…

AF_0017 : Around I take my breakfast around 7, 7 thirty then around 2 hours after the, the, the breakfast I, I take the, the, the tablet measure blood pressure but now fasting after fasting breakfast then I take the medicine around 7 thirty or 8 o’clock then around 10 then I take the measure measurement.

I_01 : The measurement…

AF_0017 : Ah measurement ah.

I_01 : So at night, now?

AF_0017 : Now, now at night.

I_01 : Ok, ok then like when you take the blood pressure do you feel anything or not… any feelings that come up?

AF_0017 : So far no.

I_01 : How do you feel?

AF_0017 : Oh ok normal, because I relax only because I know when you taking the blood pressure you cannot feel uncomfortable or feel un- feel funny, funny thing cannot!

I_01 : You mean like anxiety?

AF_0017 : Anxiety yah something like that, you must feel relax and make sure you in the calm position not, not in the rush or whatever no! [Participant laughed]

I_01 : Yah were there any times when you felt a bit like an- anxious things like these?

AF_0017 : If felt anxious just a bit then thing will go up. [Participant laughed]

I_01 : So, so when you take do you feel that or not?

AF_0017 : I think got once only.

I_01 : Ok so most…

AF_0017 : Because…

I_01 : Of the time…

AF_0017 : I, I missed one time I forget nearly 10, 11, 12 o’clock midnight I kalam kabut. [Means “panicky” in English]

I_01 : So when late you feel…

AF_0017 : I late I put kalam kabut [Means “panicky” in English] then I do this one all fast one then around 12 o’clock that one because the date change after 12 you see, midnight.

I_01 : Ok, ok so you always take at the same time?

AF_0017 : I take I take 11, 12… 10, 10 thirty sometime like this if I forget 11 something like that.

I_01 : Ok, ok so you try to take at least one time everyday.

AF_0017 : Because everyday they, they, they tell one time everyday. [Participant laughed]

I_01 : Do you take more than one time?

AF_0017 : No [Participant laughed]...

I_01 : Oh no?

AFP2_0017 : Not instructed that way that, that way I follow the instruction only.

I_01 : You follow ok, ok.

AF_0017 : If they said can then I will take no problem because I’m always at home you see?

I_01 : Ok so did you manage to watch any of the videos or not got videos inside, you know?

AF_0017 : This one I, I watch it yesterday but this one all I know already in the TV you see?

I_01 : So you did look at it you…

AF_0017 : But it’s same like the TV what then I but they that one the video videos about the exercise is it?

I_01 : Yah.

AF_0017 : I, I know what is the exercise the, the best the best exercise actually is the qigong [Means “a kind of Chinese exercise” in English] you know breathing, breathing and that one is the…

I_01 : Did you manage to watch the video until the end or not?

AF_0017 : No, because I know is in the Tv already then…

I_01 : In Tv in Tv what program is it?

AF_0017 : Advert- when, when they advertise the… if you see the Suria [Means “A Malay channel” in English] one last time a few months ago when I saw the, the siri gajah [Means “A Malay program ” in English] all the, the life of the old people you see? What they should do what they, they, they should what they should do with their friend and one of them exactly is in, in, in this video right? [Participant laughed]

I_01 : So you, you remember!

AF_0017 : I remember I know already this one regarding the exercise I don’t want I, I know exercise.

I_01 : Ok then the cooking one got watch or not?

AF_0017 : No I didn’t.

I_01 : Then the information one the information one did you watch?

AF_0017 : My information to you what I talking right actually is cover everything.

I_01 : Ok but you got watch the video?

AF_0017 : I cover all the I didn’t watch the video because I myself understand actually, actually what, what I should do I know the only thing whether I can afford to do or not [Interviewer laughed] that’s all only, whether I, I lazy or not if lazy then I didn’t do what.

I_01 : Ok so you said that you watch the videos I mean you watch it on Tv program before right you kind of know already so what can make you or encourage you to maybe watch the video. Next like the next when we have this for patients you think we can make it more like attractive to you or not?

AF_0017 : This one depend, depend on individual and depend on the program you normally human being like, like when we see something already we, we have seen we som- seen it before then we don’t want to see anymore, [Participant laughed] something like that.

I_01 : Ok, ok then…

AF_0017 : Unless you are changing this one different month different program different then here everything every time new, people human being like that every they like to see new thing better for them.

I_01 : You want to see new things new things like how is it like?

AF_0017 : Whatever program you have to it, it should be different from the earlier one that’s all I mean.

I_01 : So you find that the video is similar?

AF_0017 : Yah similar to what, what the Tv I don’t want to see.

I_01 : The Tv program ok, ok, ok how long did you watch the video for like when you watch…

AF_0017 : Just a while only I just a while I see only then I don’t want to see [Participant laughed] because I know this one I already saw that.

I_01 : Ok then ok then you did you do the survey there are some survey questions that come out right?

AF_0017 : No, I didn’t do, I didn’t open also.

I_01 : How come?

AF_0017 : I lazy to do survey until you come like this then I, I do I have no choice you see if that one I have choice I don’t want to do.

I_01 : Why don’t want to do, why?

AF_0017 : I don’t want…

I_01 : You got any…

AF_0017 : I don’t even open also I don’t know what you are talking about inside, inside there…

I_01 : Oh, oh…

AF_0017 : I, I only do survey is do survey if, if you are in front of me then I, I talk, if…

I_01 : Ok if it’s the…

AF_0017 : Like, like this one on, on the more, more understanding more easier than then I have to read…

I_01 : Ok if let’s say…

AF_0017 : I have to read, I have to write, I have to answer, to me troublesome.

I_01 : Oh troublesome!

AF_0017 : Can, can put there.

I_01 : Ok so you find it a bit like troublesome.

AF_0017 : Ah troublesome only. [Participant laughed]

I_01 : Ok, is it because it’s too long the survey is it because…

AF_0017 : I didn’t see at all. [Participant laughed]

I_01 : You didn’t even see at all ok, but did Miss M, she came right? Miss M you know the one who gave you the device the, the girl she passed you the device she did ins-

AF_0017 : She…

I_01 : Gave you some instructions right?

AF_0017 : Yah she tell me already this one got survey got that you can I you can open or whatever she tell me how to use this one all, she explained what she need to explain if not how I’m going to know how to use?

I_01 : Yah, did you find it a bit how is her explanation, is it ok?

AF_0017 : Ok, normal.

I_01 : Normal.

AF_0017 : We can understand.

I_01 : Can understand.

AF_0017 : And we can follow, no problem.

I_01 : Ok not too difficult right?

AF_0017 : Ah not too…

I_01 : Let’s say if there was nobody to explain to you like I mean Miss M she can’t she came here she passed you the device but she never explained to you right you think you can still use or not the device?

AF_0017 : Unless there is a instruction there then we just follow if we can use we, we can manage then we use if not can’t manage they all lazy because I’m put aside. [Interviewer and participant laughed] It’s better personal ex- explanation is better than writing then you, you show me how to do it’s a one only what this one because everyone knows how to use, this one I know already no need to teach me. But this one how you, you, you want to enter code, you what whatever just press I believe one only not even 5 minutes a bit only you tell me I know already what better than to read instruction follow this one follow so many instruction if we want to, to, to put…

I_01 : So you still prefer people explain to you?

AF_0017 : Ah… just like that.

I_01 : Ok, ok then what about like the popped up message you know there ‘s these popped up messages like you know those encouraging message…

AF_0017 : This one…

I_01 : You know diet advice did you…

AF_0017 : Yah we already we already…

I_01 : See the, you already… how do you find it?

AF_0017 : Every time is same, well done it’s your, your, your blood pressure is at acceptable condition whatever blah, blah, blah I already know that every time the same then… [Participant laughed]

I_01 : So you feel that it’s al-…

AF_0017 : No, I…

I_01 : Always the same?

AF_0017 : All that one is the same after we do the blood pressure is the same yah.

I_01 : Any, any feelings that come up or not did it did it affect make you bet- feel better or not?

AF_0017 : As long as this one actually with without telling us this one also we know from the number we know is whether ok not ok the only thing if not ok also like that day one shoot to 170 you don’t have a I don’t know whether you we’ll give a chance. Come to hospital now, immediately you never give the instruction that way, correct? [Participant laughed]

I_01 : So ok, ok.

AF_0017 : Unless every time you see or not instruction go to hospital maybe oh your blood, blood pressure is too high already please make sure you take care of your, your food or whatever some advice.

I_01 : Did you see the advice when it when it went up?

AF_0017 : I, I don’t see because my, my…

I_01 : Really?

AFP2_0017 : My blood pressure all very good.

I_01 : But that one you said there was one time it went up very high right?

AF_0017 : Went up not very high…

I_01 : Like…

AFP2_0017 : It’s just 138 only.

I_01 : Ok, a bit higher…

AF_0017 : A bit only.

I_01 : Was there a message that came up, after that?

AF_0017 : Still good what [Participant laughed] the message still same, good!

I_01 : Ok, ok, ok then what about like the…

AF_0017 : Because as long as one below 140 should be ok what.

I_01 : Ok then did you like any of any feature that you like or not any anything that you like in, in this program anything that was your favorite?

AF_0017 : If you give like some knowledge what we go through what, what will happen to when your blood pressure too high what is the cause and what will happen after it’s keep on you cannot control your blood pressure then the, the, the you got stroke whatever you got heart, heart problem whatever. This one they must maybe tell if not in detail roughly what is happening and why we should why we should control and what things you must do with the medicine must give the solution roughly if, if you tell this, this sort of things is good, good for us good for the patient. What happen then how to manage after the thing happened?

I_01 : Ok, ok so you but there was this information the video you know the video on AF…

AF_0017 : Oh I…

I_01 : The information…

AF_0017 : I didn’t know I didn’t they don’t, days is it?

I_01 : Yah it’s a state index it’s inside there yah some of it is inside there.

AF_0017 : I, I didn’t this one I didn’t go through.

I_01 : Ok, ok and, and you didn’t go through because you said you, you seen it before already.

AF_0017 : Ah the first one I see I see this one before the Tv then [Participant laughed] no point.

I_01 : Ok, ok, ok what about like do you encounter any problems any thing or not any, any issues with the app any difficulties with app?

AF_0017 : No, no, no.

I_01 : Don’t have?

AF_0017 : Don’t have, this one simple.

I_01 : Simple?

AF_0017 : Simple, unless old already like my mother 80 years old maybe it’s not simple for them to use for us 60 years old this one, ok.

I_01 : Were you aware there was a number or not, a number you can call there’s this number…

AF_0017 : Yes, there is.

I_01 : Did you manage to call up the number?

AF_0017 : There is no problem I didn’t call.

I_01 : You didn’t call ok, ok, ok but you think it’s helpful or not to have the number?

AF_0017 : Yes, good.

I_01 : It is!

AF_0017 : It benefit really we need some help that we there is a number to call there is a phone.

I_01 : So you, you think it’s helpful, ok.

AF_0017 : Helpful.

I_01 : Ok then what about like the let’s say you know there this chart you know the, the, the graph the blood pressure that graph like did you go and see a doctor in this time period then you bring the graph, show your doctor?

AF_0017 : For sure this one is go to the hospital already what…

I_01 : Hospital correct, but let’s say you go and see another, another doctor right some other doctor then you got this chart do you did you did you in this time period go and see a doctor?

AF_0017 : If you have the I mean we can have this information why not but this type of things all like now I not intend to change to other hospital then Hospital N know…

I_01 : Only Hospital N ok, ok.

AF_0017 : So far Hospital N next time may maybe I become millionaire [Interviewer laughed] then I go to my private hospital already. [Interviewer and participant laughed]

I_01 : Hopefully one day [Interviewer and participant laughed] ok, so do you find the study this 6 weeks is it too is it just right, too long, too short how do you find it?

AF_0017 : To me no, no problem because I’m not working you see to me no problem at all ok, but this one more than 6 weeks what.

I_01 : I mean we come we come a bit later because we have to arrange with you right, yah and also we have things…

AF_0017 : This one 2 months already. [Participant laughed]

I_01 : Yah, 2 months but let’s say right if we do this study for 2 for more people to try you think 6 weeks is ok or how long do you think is the it’s a good time period?

AF_0017 : I think one month should be ok.

I_01 : One month?

AF_0017 : One month should be ok not too, too long already 6 weeks because if any if normally per month you, you count is better than the 6 weeks unless you have your own reason I don’t know but 4 weeks it should be ok.

I_01 : It’s ok, ok if we do it longer?

AF_0017 : Like to my opinion if you do one do it longer for patient like me no problem…

I_01 : No problem?

AF_0017 : Because it’s good for us what because we, we can manage to see every, every day yah for us because we are not working then no problem to the people who is working is troublesome.

I_01 : Ok, ok so for only for workers who are working ok.

AF_0017 : Working, or this one actually is movable can we can carry also or reason troublesome to carry. [Participant laughed]

I_01 : Is it you find it troublesome?

AF_0017 : No, no if we want because you will you go to work then you your bag is got your, your own things already there plus this one, if you…

I_01 : But let’s say if you go and see doctor then you need to bring this you find it o- is it troublesome?

AF_0017 : That one see doctor once a while only what not everyday we see what [Interviewer and participant laughed] but work everyday you must work…

I_01 : Everyday, yah.

AF_0017 : You must you have to carry this one everyday.

I_01 : Ok, ok so if it’s everyday then, then, then…

AF_0017 : Unless this one you leave it at, at the work place at same time you just measure the thing that one is no problem can be arranged if that one is depend on if you are working at the same place, no problem. If like if security they change they, they difficult sometime there’s, there’s no permanent place diff difficult because if I can work I work as security only but normally if I do if I work as security also when I not working then I measure this one same because normally permanent shift you see, yah. They all no problem at all actually, actually there’s no problem but it’s a it’s advantage to the patient actually if they have this one every time can manage their blood pressure whether is correct or, or whether in, in a good ter- good co- condition or not, then the doctor also know.

I_01 : So the, the where do you feel that like with this connection to hospital right and to the clinic you think that it’s it will…

AF_0017 : It’s a good…

I_01 : It will reassured you it will make you feel better?

AF_0017 : Yes, because anything happen they know also what or this one every time 170, 160 this one maybe they will call you to come also, correct or not? Or we have this number then my few days already 170, 170 how is it, you want me to come to hospital or not you can also ask what because we also worried if this one everyday keep on go up. But provide you allow to this, this one when we on the things we can measure every even every hour also ok we can measure or every 2 hours also we can measure, then it’s a good things if we have and collection then you know what is actually happen, at home.

I_01 : So you don’t mind if you can measure more yourself yah ok, actually you can you know the I don’t know whether Miss M did tell you or not but actually you can do more than one time it’s ok one.

AF_0017 : Never tell me that’s why I just…

I_01 : Miss M didn’t tell you…

AF_0017 : Leave it I leave it to one time only every day.

I_01 : Ok, ok Miss M so Miss M didn’t tell you?

AF_0017 : But sometime I didn’t hear or I miss, miss [Interviewer laughed] misunderstand sometime…

I_01 : Yah, yah no, it’s correct maybe…

AF_0017 : If, if you just tell ok Miss M say you can measure 3, 4 time per day also no doesn’t matter then sure I will do 3, 4 time.

I_01 : Ok so you don’t mind?

AF_0017 : I don’t mind.

I_01 : Ok, ok then if there ok let’s say we have a few different device ok we have tablet, we have phone, we have computer and then we have like other electronic device which one will you prefer which form will you prefer?

AF_0017 : For what purpose for me?

I_01 : Like let’s say we have this app but we have phone one we have a we have a tablet and we have a desk top computer and then we have other kind of electronic device which one will you which kind of electronic device will you prefer?

AF_0017 : This one is ok already what.

I_01 : It’s ok already, ok so you don’t you’re, you’re, you’re comfortable with a tablet. Ok, then what about like if we have a paper a paper form where you record on paper then you sent to us and then we sent you information via the brochure would you prefer a paper form or you prefer electronic form?

AF_0017 : This one better [Interviewer laughed] definitely.

I_01 : Ok so you prefer electronic form, ok so you…

AF_0017 : Automatic what this one go immediate, not like paper you must still submit or you must what’s app or whatever.

I_01 : [Interviewer laughed] So did you find any difficulty like going through the app or not like you know you know you have to scroll in to the your result then scrolled out then if…

AF_0017 : Yah that one no problem.

I_01 : No problem?

AFP2_0017 : No problem.

I_01 : Ok, ok then how’s the, the, the brightness of the screen is it ok?

AF_0017 : That one no problem my, my...

I_01 : No problem ok then the, the font the font is it too small too big?

AF_0017 : Ok, ok.

I_01 : Ok, the screen size?

AF_0017 : Ok no, no…

I_01 : Also ok, ok, ok and so like any other suggestions, ideas that you can you know you want to see in this kind of program maybe we can introduce to next, next time to more people including yourself right, what would you like to see that we can add on to this?

AF_0017 : If you like you, you know our concession normally diabet- when, when you have the blood pressure we have this diabetes also normally cholesterol and whatever the, the program you just give us some idea what to eat, what time or what ex- what exercise all, all these things. You put whatever the diet dietician when we they explain you can put that, that here better at least we can refer what food there I we should eat, what time whatever, whatever information you, you regarding the dietician.

I_01 : So but would you find that it’s too much if we add we add in more and more things?

AF_0017 : But because this one option, you can read or what but the things is the, the, the blood pressure you take everyday enough already and when you have time this one you can read if you have nothing to do, then you can read.

I_01 : What read, read what?

AF_0017 : Read the, the dietician information regarding the dietician for, for the blood pressure, for the diabetes, for the cholesterol for the heart, heart people heart problem.

I_01 : So you prefer reading because we do not you see…

AF_0017 : That’s why I read in internet all [Participant laughed] internet…

I_01 : You don’t so you don’t like watching video I mean not, not don’t like but like because we also have video right you prefer video or reading?

AF_0017 : Video, if I see already video once I don’t want see second time already.

I_01 : You don’t see second time!

AF_0017 : Unless exercise if exercise like you, you have the instructor there different but this because that exercise I saw in on the TV already I, I, I don’t want to see already.

I_01 : Ok so you want to see new things is it, new things?

AF_0017 : If possible not every time this one fix because it’s connected is it and you have your main computer there you can always…

I_01 : Change?

AF_0017 : Change the thing what.

I_01 : So if we can change the videos are all the time you…

AF_0017 : Yah, better!

I_01 : You are o- you prefer that.

AF_0017 : Yah of you all, all this one what, what you do is you programmed over there this thing we change every week or every month you no need to do anything everything you ready and press the button all automatic what, [Interviewer laughed] now all digi- digital already.

I_01 : Digital correct, correct.

AF_0017 : Digitalized already.

I_01 : Yah digitalized, so let’s say we introduce this app ok you said that you were diagnosed with AF in 20… a few years ago right 2015 is it?

AF_0017 : No I can’t remember what is the name but that one actually is the, the blood…

I_01 : The bypass…

AF_0017 : I do the bypass because the what you call it the vein stuck with the cholesterol that one…

I_01 : Yah, yah ok, ok so we you prefer us to introduce it when you have first was diagnosed with the AF or a bit later or in the middle like you know if we let’s say we have this program…

AF_0017 : No we…

I_01 : We, you looked back at your, your condition you, you prefer us when would be the best, best time for us to introduce this program to you when you first had it when you first dis-

AF_0017 : When, when first had it is better then we can we when why people don’t want to make decision because they don’t have the knowledge you don’t have the you don’t have anything you scare to do the, the decision or not you not confident. If you have the condition you have the knowledge some knowledge regarding after you have let’s say I first kena [Means “got it” in English] is mild heart attack mild heart attack. [Participant laughed] Very difficult to, to, to breathing to breathe but I don’t have the knowledge what to do after this they want me to do a bypass why suddenly I have to do bypass they say very difficult one. Then we do the angiogram we know what is our condition already then but this when you talk when you sick that time you cannot absorb everything you know went to the doctor talk any everything you can’t absorb. You know you, you, you have to do bypass how is it the condition then oth- outside people, don’t go bypass I think can do herbal or whatever all they give all alternative then that’s why we don’t know what to hear, I think they said one of them give me a suggestion do chelation therapy or maybe I try. Ok then, don’t want to the, the, the bypass it’s like that but if you have this one this one you, you we introduce after that after these people the patient first time kena [Means “got it” in English] then you introduce after maybe few weeks ok. This like this program you, you, you your blood pressure measure this one definitely got blood pressure got blood pressure that’s why got kena [Means “got it” in English] heart attack. Then while doing this program then you give all the information what will happen and for this particular individual one, your condition is now we already because only for him only this condition is you your, your concern like this if you want to do a, a bypass whatever this is the risk, all whatever doctor advice, put there.

I_01 : So the doctor’s advice put it inside the tablet…

AF_0017 : I this one I…

I_01 : Base on…

AF_0017 : Suggest only.

I_01 : Your results ok, ok.

AF_0017 : Because all these not difficult not easy [Interviewer laughed] to, to, to do.

I_01 : Yah a bit difficult, yah.

AF_0017 : Because this one private and confidential you see information you but I just suggest only you keep to the, the, the indi- individual per- person that, that in that type information when their condition is tell you but for general is just tell the general only.

I_01 : Ok, ok, ok so I think that’s all about it for the interview…

AF_0017 : Ok, never mind.

I_01 : Thank you very much I stopped the recording.

End of interview
