# Supplementary material for: Patient perspectives of the Self-management and Educational Technology tool for Atrial Fibrillation (SETAF): A mixed-methods study in Singapore
Source: PLoS One. 2022 Jan 21;17(1):e0262033. doi: 10.1371/journal.pone.0262033 (PMC8782297; doi:10.1371/journal.pone.0262033)
Supplement: S1 Data — (ZIP) [file pone.0262033.s006.zip › AFP2_IDI_0015.doc]

Participant ID number : AFP2_0015

Interviewer Number : I_01

Length of interview: 15.01 mins

I_01 : Ok thank you for taking part in this study the question the first question is like how long have you been diagnosed with AF and how did you find out about it?

AFP2_0015 : Many years already.

I_01 : When was that?

AFP2_0015 : How to remember, actually…

I_01 : Like estimated?

AFP2_0015 : Estimated may be 10 years with this doctor about 10 years.

I_01 : Ok, ok so like what are the ways that you, you tried to manage your AF outside of the of the hospital setting other than the advise that was given you by the doctor the dietician?

AFP2_0015 : How to answer that?

I_01 : Like on your own how did you find out what, what kind of information do you did you go through to, to find out to manage?

AFP2_001 : I read a lot I read up a lot so I will know like certain things to cut down like salt you know not to over pressure myself I will do exercise but moderately then I’m active I’m not always sitting around I do go around doing things. Just make sure that I do not like exert myself so the heart will not and then taking medication regularly taking medication.

I_01 : What kind of medication?

AFP2_0015 : The heart medicine.

I_01 : Warfarin is it?

AFP2_0015 : Warfarin also then another thing is that the doctor want me to take the blood pressure everyday so I actually have a machine of my own…

I_01 : Yah, so you have been doing it already?

AFP2_0015 : It’s a regular usually I don’t…

AFP2_0015_01 : Because before we go and see the Prof L we will send the, the whole…

AFP2_0015 : Yah my list my whole list.

AFP2_0015_01 : The, the blood…

I_01 : So you all already do the recording and sent the doc-…

AFP2_0015_01 : Sent to him.

I_01 : The whole records…

AFP2_001 : But of course mine is manually not done with the tablet, you know?

I_01 : Do you think that it’ll be maybe an improvement or better if we do it electronically you know to, to let’s say record it automatically and then like sent back to the doc-…

AFP2_0015 : Of course if it works…

AFP2_0015_01 : I suggest if the, the hospital want to improve certain things I think should help those retirees and you know to get a lower price and then we don’t mind to use it but and on top of that the gadget must be workable. I mean don’t give me something that work one day 2 day then down again then aft- very frustrated you know [Participant laughed] then we also worried whether these things really sent there or not you see these are our worries, we won’t mind.

AFP2_0015 : So to double, double make sure I record my own I do a manual recording everyday then I put the machine down you see so anyway it is useful if it is together the tablet plus the thing. I find it quite useful only the frustration is that the data doesn’t get through and I cannot get in to see the thing.

I_01 : Ok, ok so it ok so you mentioned that it was not working so what in what way it was not working, couldn’t on it?

AFP2_0015 : Cannot on to rec- record the data you know that means…

AFP2_0015_01 : Which means she cannot log into the thing even they given the password log in to the tablet it doesn’t work it showed…

AFP2_0015 : You saw right you saw…

AFP2_0015_01 : That oh the, the server is down and disconnected you know.

I_01 : That, that the server the error message there so you cannot even go inside cannot see anything…

AFP2_0015 : Cannot, so you all already set the thing I cannot do anything any changes with the settings so they were saying internet problem blue tooth all sort of thing…

I_01 : But you also pressed there’s the it’s on?

AFP2_001 : I never.

I_01 : I mean the…

AFP2_0015 : It’s, it’s on all the time.

I_01 : On, ok yah!

AFP2_0015 : I cannot adjust anything.

I_01 : Ok, ok so you mentioned that you called the Miss M a few times and she didn’t response, but then…

AFP2_0015 : She said she’s on leave.

I_01 : On leave, ok but then like I think…

AFP2_0015 : She…

I_01 : How many times did you call?

AFP2_001_01 : Yah she then…

AFP2_0015 : I called her…

AFP2_0015_01 : What’s app to her…

I_01 : Because of you know I’ll feedback to them, yah.

AFP2_0015_01 : Nearly a week and then she will reply then asked all kind of question of course she got a, a, a routine like thinking whether your phone I said silly what you give me a machine I didn’t do anything you all already do the setting I can’t I don’t even can access into the internet. So I say why she is asking the question yesterday I find it upset in the bus I was hearing her what’s app I said silly this woman you give me the, the, the gadget and then you asked me all kind of silly question…

I_01 : Ok, ok 26 April I messaged her ok the machine not working monitor and so on 26 the 2^nd^ of May then she answer me she said she just returned from work blah, blah, blah…

AFP2_0015 : Ok, ok so actually we… the technical side actually was supposed to be dealt with also company P so actually if they did not response also it’s quite something that we want to know and find out also because…

AFP2_0015_01 : She’s from company P one this lady?

I_01 : Correct yah she’s from company P yah…

AFP2_0015 : She’s from company P…

I_01 : Yah she’s not from hospital N, yah.

AFP2_001 : Then on the 26 I told the machine is down again so…

I_01 : 26 of what?

AFP2_0015 : May, May she didn’t answer me until the 30^th^.

I_01 : Ok 30^th^ ok I see.

AFP2_0015_01 : 26 of May I think.

AFP2_0015 : Then again she said that she was on leave.

I_01 : 26 of May yah ok.

AFP2_0015 : May it’s May…

I_01 : Sorry yup.

AFP2_0015 : I without re-…

I_01 : Sent…

AFP2_0015 : I did not delete the messages so yah 30^th^ May then she answer me.

I_01 : Ok so, so you what did you message earlier she re- we want to find out whether you also you mentioned about the device…

AFP2_001 : Maybe the, the device to be taken back also 26 May…

AFP2_0015_01 : Let me know the date and time but she never work… [Inaudible segment]

I_01 : How come you never mentioned there was a problem and the…

AFP2_0015 : Got.

I_01 : That was earlier is it ok, 26 of…

AFP2_0015 : 26 April.

I_01 : Ok, 26 April sent about problems…

AFP2_0015 : Then I say…

AFP2_0015 : About technical problems…

AFP2_0015 : I called the support line they do not know anything about this…

I_01 : Technical problems…

AFP2_001 : They say is there such a sur- a survey is there such a thing they don’t know anything so they couldn’t help me.

I_01 : Ok because the, the, the staffs at the…

AFP2_0015 : Hospital N…

I_01 : When you called right it goes to hospital N and the staffs they are not aware of the like say the details of the app…

AFP2_0015 : Then you all shouldn’t put that in…

I_01 : Actually that the app right is a it’s, it’s only a very small little trial that we do so it all not…

AFP2_0015 : Not many patient?

I_01 : Many people know only like the doctor TM like will know and maybe his assistant but I think other than that the other we haven’t trial to everyone else yet.

AFP2_0015 : So I give up so in the end [Interviewer and participant laughed] I told her then she answer me 2nd May then I continue again a lot of problem cannot get through so…

I_01 : Ok so you gave up completely!

AFP2_001 : I gave up completely calling her I still…

AFP2_0015_01 : But we still do, we still do.

AFP2_0015 : We still continue to use this machine to do then…

I_01 : Ok, ok that’s, that’s ok, yah.

AFP2_0015 : Yah then it’s ok so in the end on the 26 May I counted about 6 weeks so I called her so I say can you please take back the machine or not, very leceh. [Means “troublesome” in English] [Participant laughed]

I_01 : Ok, ok so anyway we just want to ask so let’s given that you didn’t really managed to use the new you know the quite frustrating experience right, let’s say we have a kind of a electronic kind of a device what would you like to see in it. I mean I understand that you already take your blood pressure quite regularly already and you sent the records to the doctor right so like if let’s say we have a device that we, we implement it like on a wider scale for patients. Like what would you see what would, would you like to see in addition to like say recording your blood pressure do you think videos will help, videos about like cooking, exercise, AF…

AFP2_0015 : This type of things?

I_01 : Some informational stuffs that we can add in do you think that will, will you like to see that?

AFP2_0015 : Like introducing the diets and helping your exercise and all that, well all those are in informative it is good but how, how many times we are afraid to actually go through. [Interviewer and participant laughed] Everyday we go through our own tablet I mean our own hand phone and so we, we relied on other things and I have health books and so on...

I_01 : Ok so if we incorporate it let’s say in your phone or something do you think that, that will be something that you would like to see?

AFP2_001 : No.

I_01 : Because you said that you, you used devices already right so maybe like if we incorporate in…

AFP2_0015 : That means in add this into…

I_01 : Yah correct…

AFP2_0015 : The phone?

I_01 : So the app will be like say in the mobile phone own form other than a tablet you know would you would that be better you know?

AFP2_0015 : Of course it’ll be better you know but you still have to try [Interviewer laughed] out to know…

I_01 : To know ok, ok…

AFP2_001 : Whether it can work or not yah like this one you try out work then it’s so frustrating then I tell myself or maybe I should just continued using this because I was thinking of going back to my manual one.

I_01 : Ok, ok so you’ll, you’ll still go back eventually I mean to this to that and all that…

AFP2_0015 : Maybe I…

AFP2_0015_01 : To be play safe because the doctor sometime will ask her did you do your, your blood pressure reading and all that…

AFP2_0015 : Because he want to know…

AFP2_0015_01 : So she will…

AFP2_0015 : The warfarin and the heart have…

AFP2_0015_01 : So she will everyday do and then we do what you called that put into a record and then we sent over to him then he will received the reading…

AFP2_0015 : Like which month I see him to the 6 months usually I will just sent 3 months I’ll sent…

I_01 : 3 months oh it’s, it’s quite long.

AFP2_0015 : Because I have to take ironol I have to take ironol in between.

I_01 : Ok, ok what is your experience like, like you know doing it manually right…

AFP2_0015 : No.

I_01 : Are there any challenges you know or difficulties?

AFP2_0015_01 : Actually is not much…

AFP2_001 : Very straight forward.

I_01 : Straight forward, ok but do you…

AFP2_0015 : No complications like…

I_01 : Forget or something like that since like use…

AFP2_0015 : Forget, sometimes if I go out too late or got some busy schedule…

AFP2_0015_01 : Maybe not one or 2 days missed like that.

AFP2_0015 : But I usually don’t missed.

AFP2_0015_01 : In fact I think she go overseas also she brought along.

I_01 : So you bring along ok, ok.

AFP2_0015_01 : Yah we do her reading everything.

AFP2_0015 : [Participant laughed] Go Hong Kong or whatever.

I_01 : You bring the blood pressure monitor?

AFP2_001 : I bring the blood pressure monitor yah.

I_01 : So something portable will be helpful to, to take around, ok.

AFP2_0015 : Of course if this is workable is good but it’s leceh [Means “troublesome” in English] I after using it…

AFP2_0015_01 : But this type of things went overseas will they still can receive those data?

I_01 : Yah it should be shouldn’t be a problem because it is all like mobile and connects to the server you know it’s like wifi like that you know it connects to internet and then like the internet will record it automatically, yah so that’s the idea behind it.

AFP2_0015_01 : But do can you recommendation to your hospital don’t use this brand, [All laughed] some faults…

AFP2_0015 : Faults…

AFP2_0015_01 : Still ok but it…

I_01 : Ok any rea- any reason why you why you say that?

AFP2_0015_01 : I very reserved when I look at company P things only I used company P things one things that I used…

AFP2_0015 : Spare…

AFP2_0015_01 : She bought for me the shaver that’s it.

I_01 : Yah no it’s ok it’s ok we want to hear we want to hear.

AFP2_0015 : Spare…

AFP2_0015_01 : To be frank I tell you because I looked at they even introduced this company P, A product and all that the colour is still…

I_01 : Different…

AFP2_0015 : Not very what you called that up to standard then I always go back to brand S and then after that now I looking at what you called that S1 brand, S1 brand is very clear because they got the machine there to generate the colour so S brand even I say sometimes it cannot beat S1 brand.

AFP2_0015 : This machine is also from company P?

I_01 : No this one is not from company P…

AFP2_001 : No this one is not…

I_01 : It’s, it’s a different brand actually…

AFP2_0015 : This one is different brand.

I_01 : Yah it’s only the device and the app is, is created by them and designed by them.

AFP2_0015 : I see.

I_01 : Yah, yah so… [All laughed]

AFP2_0015_01 : You know very reserved very reserved.

AFP2_0015 : But I doubt the government will you know…

I_01 : Actually their they, they, they are looking…

AFP2_0015 : Like they…

I_01 : To doing more using more electronic devices to help you know patients and…

AFP2_0015_01 : Originally they are from country H right the company P but I surprised man how come their device like that.

AFP2_001 : But if the tablet works or if it can be connected to the hand phone will be more useful I mean as it’s…

I_01 : Would that make it more as- reassuring to know that like say your recordings are it automatically goes back to the hospital?

AFP2_0015 : Yah of course of course you, you…

AFP2_0015_01 : Then she can save up the trouble to type all these things.

AFP2_0015 : Yah to type because every now and then I free I’ll just type in…

I_01 : How long does it take to type in everything?

AFP2_0015 : So fast, typing!

AFP2_0015_01 : She typing very fast! [All laughed]

AFP2_0015 : No, no problem one.

I_01 : Typing is the results the of the BP, ok.

AFP2_0015 : Yah, yah, yah I already have a format so I just type in…

AFP2_0015_01 : Feed in.

AFP2_0015 : Enter… then which date and which time he also wants to know the time like whether I take it in the morning or in the afternoon so usually I will put the time…

I_01 : The exact time right, like 10 a.m…

AFP2_0015 : Yah, yah when I take…

I_01 : That kind of thing, ok.

AFP2_0015_01 : We are not very smart and intelligent… [All laughed]

I_01 : No, no, no…

AFP2_0015_01 : But we feel…

AFP2_001 : Like that is…

AFP2_0015_01 : Try to cope, cope with everything.

AFP2_0015 : He also the doctor also feel more comfortable because he can look at the record and see you know whether it’s it affect me or not so if this is workable it’s actually good but this machine tak boleh [Means “cannot” in English] sorry, failure. [Participant laughed]

I_01 : No, no, no it’s not it’s not your fault at all actually in fact we want to hear…

AFP2_0015 : Maybe other…

AFP2_0015_01 : But this type of reading…

AFP2_0015 : Maybe is just this one…

AFP2_0015_01 : Prof L able to see or not?

AFP2_0015 : Can, can see.

I_01 : Yah they are able to see because there is a app they can logged in right then they when they logged in they can see also.

AFP2_0015_01 : That means if you have an account in the server take for them…

I_01 : Yah correct it’s already registered there already and so like you know when like say I logged in or the doctor logged in right you can see the…

AFP2_0015 : Even the graph?

I_01 : Can see the graph also and then like there will be also like surveys and encouraging messages to like you know help you to continue using it.

AFP2_0015 : Actually the survey and all those things inside I never even see. [Participant laughed]

I_01 : Yah you didn’t managed to even encounter it right?

AFP2_0015 : Yah because…

AFP2_0015_01 : Yah because sometimes this doctor she will write to him get some advice he very prone one he will reply one prof L is very good…

AFP2_0015 : Prof L is very good prof L…

AFP2_0015_01 : Even Sunday he will call and talk to her then I asked him why, why you’re still in hospi- [Both participants laughed] no I’m doing something.

I_01 : Ok so you find that kind of hearing from the doctor or maybe like a medical professional like quite useful and encouraging.

AFP2_001 : Yah when I when I was in country H I think because of the salty food and a lot of walking and you know country H country H don’t the Mrt not like ours you know…

I_01 : Yah different yah.

AFP2_0015 : So you have to walk and my leg start to swell…

I_01 : Oh ok so…

AFP2_0015 : So I emailed him you see doctor L what to do then he asked me to take what tablet and what to do so he’s a very good doctor.

I_01 : But let’s said he is not a doctor it’s like a nurse or like a…

AFP2_0015 : If they can give me the proper guidience, ok.

I_01 : Yah or like some other you know could be like, like pharmacist and some, some, some other person you know…

AFP2_001 : Oh no…

I_01 : A non a non…

AFP2_0015 : I would rather trust my own doctor.

I_01 : Because the doctor like the idea is that the doctor will be able to monitor but not all the time also because you know the…

AFP2_0015 : Correct but we also not all the time trouble him it’s just a situation that need…

AFP2_0015_01 : Like some situation like that he is very prone.

AFP2_0015 : Because if there is a, a swelling of the leg that means I’m taking too salty things already which is not in my diet one so I know then I asked him what to do to clear it so… yah this thing…

I_01 : Like if it’s a doctor’s assistant is it ok?

AFP2_0015 : Yah it should be.

I_01 : It’s ok, ok so I think any other comments that you feel that you know like you want to comment about this experience that you had? [All laughed]

AFP2_001 : No, no. no it’s ok.

I_01 : Ok, ok so thank…

AFP2_0015 : I think mine is one in a million. [Interviewer and participant AFP2_0015 laughed]

I_01 : No it’s not actually it’s not your fault anyway it’s just a by…

AFP2_0015 : Maybe it’s just this device that…

I_01 : The it’s the device it’s not you actually so I hope you don’t worry about anything on…

AFP2_0015 : Or they will…

AFP2_0015_01 : May maybe they…

I_01 : From on your side…

AFP2_001_01 : They are phasing out this type of model so they recommend to hospital quickly go all you know [Interviewer and participant AFP2_0015_01 laughed] maybe…

I_01 : You know…

AFP2_0015_01 : Possible, [All laughed] that’s why I said sometimes computer show you go down there normally they will throw out all those things implement some new thing but on top of that they said ok sales this one all are you know get rid of it.

I_01 : Ok, ok so anyway thank you for your feedback and your and your comments I’ll stopped the recording.

End of interview
