# Supplementary material for: Patient perspectives of the Self-management and Educational Technology tool for Atrial Fibrillation (SETAF): A mixed-methods study in Singapore
Source: PLoS One. 2022 Jan 21;17(1):e0262033. doi: 10.1371/journal.pone.0262033 (PMC8782297; doi:10.1371/journal.pone.0262033)
Supplement: S1 Data — (ZIP) [file pone.0262033.s006.zip › AFP2_IDI_0013.doc]

Participant ID number : AF_IDI_0013

Interviewer Number : I_01

Length of interview: 23.03mins

I_01 : Ok so thank you for take taking part in this study so the really the first question we want to ask is how long have you been diagnosed with AF and how did you find out about it?

AF_0013 : January.

I_01 : January when?

AF_0013 : January I think maybe 10 on the 10.

I_01 : Oh January 10, 2010 is it?

AF_0013 : 2018.

I_01 : 2018!

AF_0013 : Yah 2018 yah that’s right.

I_01 : Oh so it’s this year?

AF_0013 : Yah so found through I bought a machine so the machine has this irregular heartbeat picture so I discover from there.

I_01 : The, the what kind of machine is that?

AF_0013 : The I, I think is a those, those blood pressure machine.

I_01 : Blood pres- some so it’s a blood pressure monitor?

AF_0013 : Monitor yah it’s a monitor.

I_01 : Is it the same as this one?

AF_0013 : Oh no, no, no.

I_01 : The, the machine?

AF_0013 : The one is what you call the Japanese I think is Japanese brand or what…

I_01 : It’s a different brand…

AF_0013 : Different brand different…

I_01 : Is a brand “O”?

AF_0013 : Brand “O” yah brand “O”.

I_01 : Brand “O” but it’s a blood pressure monitor…

AF_0013 : That’s right.

I_01 : Monitor?

AF_0013 : That’s right.

I_01 : But does it comes with an app and all that the tablet and app?

AF_0013 : No tablet no tablet one.

I_01 : Ok, ok then you found out is when you when you take then like what how what was the like how do you know because usually when you take the it will just give you the result right?

AF_0013 : Ok because there is a feature the this irregular heartbeat feature so this one has previous monitors no such features.

I_01 : So, so that was how you get you found out?

AF_0013 : Correct.

I_01 : And then you told the doctor about it?

AF_0013 : So I went to went to the clinic and then after that the hospital ”N” that’s right.

I_01 : And that’s how they diagnosed you with the AF?

AF_0013 : Yah correct because it repeatedly shown on the monitor.

I_01 : Did they give you any advise about what to do you know…

AF_0013 : Who?

I_01 : Like the when you visit, visit the clinic right, right any advise about you know what to what to do…

AF_0013 : The advise?

I_01 : What did he tell you?

AF_0013 : Oh went through all these all the tests and went through the I think there’s one E.. E.. ECG…

I_01 : ECG!

AF_0013 : And got one ultrasound then after that follow by one is what, what FF what, what is that thing the one to check the blood blockage one…

I_01 : The check it, ablation?

AF_0013 : The, the what don’t know what term is that, that means check, check whether there is any blockage…

I_01 : Ok just to…

AF_0013 : AGI don’t know what you know?

I_01 : AGI!

AF_0013 : What is that what is that?

I_01 : E.. E.. EGC…

AF_0013 : Intigram?

I_01 : EGC is it?

AF_0013 : Egigram I think.

I_01 : I think Electro cardiogram is it?

AF_0013 : No, no, no agel... agel agelgram agelgram…

I_01 : Angio- angiograph graphy something like…

AF_0013 : Went through angiography so no, no blockage so...

I_01 : Ok, ok so you went to do all these other checks?

AF_0013 : Yah so I’ll be doing the, the ablation…

I_01 : Ok, ok, ok when, when is the ablation?

AF_0013 : Next week 15^th^.

I_01 : Next week ok, ok so like did you get any advice about like what the kind the other things like the diet you know exercise you know did the doctor tell you anything or not?

AF_0013 : No I do not have much symptoms so everything as per normal take the medicine…

I_01 : Ok then, then the…

AF_0013 : 5 mm, mg.

I_01 : Is it warfarin?

AF_0013 : No, you know can’t remember the term…

I_01 : Some, some medication…

AF_0013 : One some, some medication yah, yah.

I_01 : Ok so they so they prescribed that to you ok so then other then you ok this the advise that you get from the doctors and the, the hospital right the clinic right did you like you know find other information outside of it you know your…

AF_0013 : Yah, yah from the nets…

I_01 : Internet.

AF_0013 : To confirm what it was I’ve been told right.

I_01 : Is it internet search?

AF_0013 : Yah that’s right, yah.

I_01 : Ok so like what did you find from this search like generally you know?

AF_0013 : Ok, ok I’ve have it’s, it’s almost in line with what the doctor said ok so, so at least has more comfort…

I_01 : Ok, ok so it helps you…

AF_0013 : I mean talk to friends also.

I_01 : Talk to friends ok.

AF_0013 : Talked to friends some of them have similar maybe other types of symptoms not exactly relating to this but then it at least gives some relieve yah.

I_01 : Ok, ok so any other ways of looking for information and, and…

AF_0013 : The nets from friends…

I_01 : Any others?

AF_0013 : That’s all.

I_01 : That’s all I see ok, and do you find that the it’s helpful or not like you know the like your friends and talking to them and all that?

AF_0013 : Yes, yes, yes firstly you, you realized that you are not alone from the net actually you can confirmed what the you had been told so give you at least more comfort.

I_01 : Ok, ok so like you know during this period of 6 weeks of the study like what was the, the sequence that you, you, you take you I mean like you took your blood pressure…

AF_0013 : Follow diligently every you know every morning…

I_01 : Every morning.

AF_0013 : About say 7 then just read through those surveys and messages and the videos and just follow accordingly.

I_01 : Ok then do you think that do you take your blood pressure at any other time of the day?

AF_0013 : No, no same time.

I_01 : Ok is there a reason why you take in the morning?

AF_0013 : Morning convenience.

I_01 : Convenient.

AF_0013 : Convenience!

I_01 : Is it before you go to work?

AF_0013 : That’s right yah that’s right.

I_01 : Ok, ok then like ok so there are many features in this, this tablet ok we wanna just ask about every individual one so ok the survey for example right did, did you do any of the surveys or not that were given to you?

AF_0013 : Yes all surveys.

I_01 : How do you find it… like personal…

AF_0013 : I think survey is important I think they asked you how, how you doing for the day you know so at least that one that’s that is important because if that day you are not feeling well so it may affect your blood pressure so I think that part is important the survey is important.

I_01 : Do you feel that it’s too long or not?

AF_0013 : No quite short sometimes, sometimes a little long but most of the times are very short.

I_01 : Ok then what about like is it too frequent you know?

AF_0013 : No I think it’s ok frequency is ok.

I_01 : It’s ok, ok what about like do you think it’s too difficult or…

AF_0013 : No.

I_01 : Too easy or, or I mean how do you think about it?

AF_0013 : I think it’s reasonably simple yah quite simple only sometimes you, you certain sentence they structured in such a way you can’t understand them exactly.

I_01 : Ok in what way?

AF_0013 : So maybe, maybe sometimes they asked you no I can’t remember off hand but I mean there are only once or twice most of the time they are ok.

I_01 : Ok is it helpful or not for you overall?

AF_0013 : It is helpful it’s helpful.

I_01 : Helpful ok, then what about like the videos you watched them?

AF_0013 : Yes, yes, yes.

I_01 : Any favorites?

AF_0013 : Favorites, I think the one the part where they, they informed you that this actually AF can actually caused a, a stroke because all the while people thought AF has nothing to do with the, the accumulation of the what you called that the, the, the those…

I_01 : The…

AF_0013 : Particles…

I_01 : Yah correct the, the part-…

AF_0013 : So, so after looking at these things you realized that actually it, it actually can, can caused this, this is really useful.

I_01 : The that’s the information that one?

AF_0013 : That one with all the graphics over there’s…

I_01 : The, the overall one the AF overall…

AF_0013 : Overall the, the AF that one how to get, get the particles out and then how it go all the way to the brain and that is very useful.

I_01 : What about the others like the, the like the exercise one the cooking one…

AF_0013 : Cooking?

I_01 : Yah do you find that useful?

AF_0013 : Less useful because what we want is actually how, how, how these things will caused the stroke?

I_01 : Ok did it did you feel that it has change the way you like you know do your routine every day you know go like say when you go out you watched you know you are more careful about what you do all that…

AF_0013 : No, no because I, I think it confirmed what I have been doing that’s all because I already sort of know about it.

I_01 : You know about it.

AF_0013 : It confirms it confirms.

I_01 : Confirms.

AF_0013 : Confirms yah.

I_01 : But does it does it change anything?

AF_0013 : No, no, no because it, it just confirmed.

I_01 : Ok so it’s confirmed.

AF_0013 : No, no changes because all the while I’m doing what they actually recommend.

I_01 : Oh ok so you just follow.

AF_0013 : Correct.

I_01 : Ok, ok then like what about like the popped up messages you know there are some messages that give you some encouragements right?

AF_0013 : Yah, yah it’s good that is good…

I_01 : How do you find it?

AF_0013 : That is important because sometimes when a guy do it in TV everyday then quite boring…

I_01 : Quite boring.

AF_0013 : So some these, these messages do established rapport.

I_01 : Ok in do you feel that I mean what are some of the emotions that you feel when you see all these like messages you know do you feel like…

AF_0013 : Good feeling…

I_01 : Good feeling ok.

AF_0013 : Because of encouragement rapport.

I_01 : Ok so it does help?

AF_0013 : Helps a lot this is important.

I_01 : A lot ok.

AF_0013 : Because you, you are not doing alone you know that somebody, somebody encouraging you helps.

I_01 : Does it comes up too much?

AF_0013 : No, no.

I_01 : Ok, ok or too little or just right?

AF_0013 : Just nice just nice.

I_01 : Just nice ok it’s just nice ok so like there’s also the, the blood pressure monitor do you when you like when you take your blood pressure do you feel anything or not like when, when you try to take it every morning you know…

AF_0013 : Oh…

I_01 : What are the things you feel like you know…

AF_0013 : When most of the time we take blood pressure we also worry about the readings…

I_01 : Oh ok so you do.

AF_0013 : So these are very normal [Interviewer laughed] yah, yah the feeling normal feeling there’s normally you take you worry about how’s the reading going to be out so it is as per normal that type of feeling.

I_01 : As per normal…

AF_0013 : As per normal.

I_01 : So a little bit of worry?

AF_0013 : Anxiety…

I_01 : Anxiety yah ok.

AF_0013 : A little bit this is a the routine anxiety not, not because of this type this case.

I_01 : You don’t think it’s because of this I mean like it’s, it’s, it’s something that you, you feel like it’s just a normal anxiety that you get.

AF_0013 : Anybody who supposed to take a reading…

I_01 : Anyone…

AF_0013 : And supposed to know the result they have these normal anxiety.

I_01 : Ok, ok then like do you like you, you find it easy, difficult to, to take to do all these the whole process like or just right?

AF_0013 : Just right just right just right just right.

I_01 : Ok, ok so you want ok so we also want to ask you know when the, the Miss “M” passed you the device right when she came did she give like any instructions or anything…

AF_0013 : Yah, yah, yah she gave quite detailed instructions yah and then…

I_01 : Ok how do you find…

AF_0013 : Most of the time I think it’s quite simple it’s quite simple she gave instructions.

I_01 : Ok did you feel that it is like too ok so you said it’s simple for the, the instructions…

AF_0013 : For those who…

I_01 : Wise or the doing the device?

AF_0013 : Who had already bought such device is simple so to me is simple because I used this device before.

I_01 : Let’s say if she came then she didn’t give you the instruction will you be able to do it yourself?

AF_0013 : Got to struggle a bit like for awhile.

I_01 : For awhile. [Interviewer laughed]

AF_0013 : Roughly know what is happening but instructions given so ok.

I_01 : Ok, ok, ok then also like did were you aware there’s a number on the on the tablet a number there?

AF_0013 : Yah, yah, yah my name was there.

I_01 : Correct so did you try to call that number?

AF_0013 : No, no, no…

I_01 : Never?

AF_0013 : Not necessary.

I_01 : Why?

AF_0013 : Because no abnormal readings no, no issue because I can I can figure the how do it.

I_01 : Ok, ok, ok so, so you only call let’s say if, if as not normal?

AF_0013 : If some it’s a breakdown then yes.

I_01 : But like do you know you could also call it for like, like other things like advise and…

AF_0013 : No, no I’m not aware.

I_01 : There are other things you know?

AF_0013 : No, no called for advise because…

I_01 : Not aware?

AF_0013 : Not aware and it, it doesn’t crossed my mind.

I_01 : Ok, ok you didn’t of it that way the number…

AF_0013 : Yah because it’s, it’s AF after all, am I right? So I think it’s not those like what you called that the cardiac arrest thing and then you need advice this one yah because my, my the doctor I think she, she told me that this is not life threatening so ok.

I_01 : Ok, ok so you ok…

AF_0013 : There’s no necessity to border the, the whoever there I mean is bordering him he’s working right?

I_01 : Yah so like ok when you when you, you know this device right allow you to track your blood pressure like you know that you or do you think that it helps or not the tracking part you know the tracking of like…

AF_0013 : It helps I was a bit surprise because really looked at the top part the bottom they don’t looked at it they say the, the top is, is stylis- stylistic you know the, the other bottom one they’re only looking at the top part…

I_01 : So…

AF_0013 : So they, they view the top part only so normally they viewed top bottom…

I_01 : But…

AF_0013 : This case they only looked at the top part.

I_01 : Ok but in terms of tracking the this condition I mean like the, the different readings everyday and that does it help you?

AF_0013 : Helps, helps, helps it helps, helps.

I_01 : How do you feel when you look at it like you know like the graph and, and, and…

AF_0013 : The feedback from the message that says it’s normal so that gives some comfort except for one day there was one day the pressure extremely low so they come with an advice says it quite very low I have to take one more reading. If, if not then you if it’s consistently very low then you got to see a, a specialist so I…

I_01 : So you…

AF_0013 : Took one more reading after maybe about 10 minutes 15 minutes time then the, the reading back to normal, I don’t know what happened that day the reading I got to take 2 reading for one day…

I_01 : Ok, ok why?

AF_0013 : It, it should be inside the readings.

I_01 : Ok but usually is one, one reading only you take one time only.

AF_0013 : Correct.

I_01 : Ok, ok then like ok so like how do you feel about the overall duration of the study is it too long for you like you how do you feel about it too short…

AF_0013 : One hour…

I_01 : Too long too average?

AF_0013 : About one hour one hour you are talking about the period…

I_01 : As in like 6 weeks right?

AF_0013 : 6 weeks?

I_01 : Yah so…

AF_0013 : I think 4 weeks will be just nice.

I_01 : 4 weeks ok, ok.

AF_0013 : 4 weeks!

I_01 : Ok then like let’s say if this device could be introduced to your let you know when you first found out right that you had this condition right? Like this throughout this time period like if this device would do you think that it will be the best time to introduce it to, to, to you know in this time period. Throughout this time period right would there be a best time to introduce this device into helping you, you know manage and monitor your care is that at the beginning the middle or you know now?

AF_0013 : You know because most of the time you go and see a specialist so specialist will give you special instructions you know and…

I_01 : Let’s say yah…

AF_0013 : Oh you, you talking about monitor your pressure…

I_01 : Correct let’s say they try to introduce it to you right let’s say when you first found out like would it be better if you have gotten it at the beginning rather then you know now or like maybe in the middle yah.

AF_0013 : Ok I feel that since mine is AF this is only measuring the top and bottom and, and got one pulse rate this top and bottom so if you are talking about top and bottom not much relation to the to the to the AF, AF more on the more on the pulse more on the pulse…

I_01 : So you feel there’s…

AF_0013 : More on the pulse and this is more like blood pressure…

I_01 : Yah this is blood pressure correct, correct.

AF_0013 : Blood pressure so, so I think the I think they do have the, the blood pressure AF that, that feature there so that feature is useful so have I not to use them not use that and introduce this possibly this one will highlight the AF that, that feature only for my case.

I_01 : You mean the this for this tablet…

AF_0013 : For, for this…

I_01 : You are referring to…

AF_0013 : Monitor this monitor this monitor give blood pressure and pulse rate and then maybe there is one I don’t know whether I can’t remember whether they have a is it they have a AF there?

I_01 : Yah the AF is there.

AF_0013 : They have right they have right black colour I think so if regarding the tablet right the tablet shows nothing.

I_01 : Oh it doesn’t show anything to you?

AF_0013 : It doesn’t show anything about the AF so a bit irrelevant.

I_01 : You mean this tablet?

AF_0013 : The, the reading got the upper part lower part that’s all then no pulse rate.

I_01 : Ok so yah we, we don’t take that yah. [Interviewer laughed]

AF_0013 : Then, then what is it for the AF is for pulse rate isn’t it?

I_01 : Yah but also because you know the, the, the you know when you take warfarin it’s on…

AF_0013 : I didn’t do warfarin.

I_01 : Yah so, so for it’s for actually meant for, for that…

AF_0013 : For, for…

I_01 : For the potentially but also is relevant to you because you also you have AF so, so…

AF_0013 : Not so relevant to me because you are talking about the tablet not relevant. [Interviewer laughed]

I_01 : Ok so you…

AF_0013 : I feel so.

I_01 : You, you ok, ok you don’t feel it’s relevant?

AF_0013 : Oh, oh but I let me can see my blood pressure up, down that’s all.

I_01 : Yah only, only blood pressure.

AF_0013 : Then no pulse something there’s no pulse reading there.

I_01 : Actually it’s more like for the medication part and, and also knowledge, knowledge you know the overall knowledge that means the knowledge of, of, of AF you know the other things that they are in the videos the blood…

AF_0013 : Oh more for that not so much on…

I_01 : The blood pressure is more towards the medication side then the, the video is more for knowledge you know yah but also the idea is also to see if it’s something like that right can like help, help you to consistently you know maintain the your, your condition you know. To watch it monitor yourself yah, yah so that the idea is not so much you know trying to like find some kind of you know remedies certain things certain things symptoms more of to maintain certain conditions over time.

AF_0013 : To highlight to me the blood pressure that’s all.

I_01 : But also like you know for you to see if you can monitor consistently and if that helps you or not because, because actually it can be expanded to more things. Like we just now we also want to talk to you and asked you if it’s helpful so if you say if you think that you know it’s not really directly relevant so that’s a possibility.

AF_0013 : Correct, correct yah not, not directly…

I_01 : Not directly relevant.

AF_0013 : Relevant mine is AF and then that is a blood pressure and then not don’t even a pulse rate have you got a pulse rate at least more relevant.

I_01 : It’s poss- it’s possible to add in features so you feel that if we add something like pulse rate it will be…

AF_0013 : Yes of course of course of course.

I_01 : Much better?

AF_0013 : Of course of course.

I_01 : Ok, ok ,ok then like a few more questions would you…

AF_0013 : I give you a drink I‘m forgot give you a drink. [Interviewer laughed]

I_01 : It’s alright it’s ok…

AF_0013 : I, I give you.

I_01 : Doesn’t matter it won’t be very long.

AF_0013 : Give him a drink pour something pour something.

I_01 : It’s ok doesn’t matter don’t border.

AF_0013 : What, what you want?

I_01 : Anything, water will do.

AF_0013 : Anything?

AF_0013_01 : We have we have hot drink.

AF_0013 : Cold drink I think if you…

I_01 : Just water will do [Interviewer laughed] thank you.

AF_0013_01 : I think plain water if you don’t mind…

I_01 : Yah I don’t mind don’t mind don’t mind.

AF_0013_01 : Because we, we drink coffee because... [AF_0013_01 laughed]

I_01 : Yah please, please.

AF_0013 : Yah I got you.

I_01 : So like if would you prefer to use some something like a tablet form or phone form that or desk top or any of these device if you…

AF_0013 : To, to, to…

I_01 : Let’s say if we have alternative forms or, or device you can used…

AF_0013 : Phone.

I_01 : You prefer to use phone?

AF_0013 : Tablet, tablet.

I_01 : Tablet?

AF_0013 : Visible, phone are very small.

I_01 : So phone is too small, desktop like something like laptop [Inaudible segment]?

AF_0013 : Desktop you got to you got you got to take a long time to on it no it take quite a bit of time to I think use phone is better I think phone is better phone is faster even this tablet I got to on I off it every time I on it…

I_01 : It takes a bit of time right?

AF_0013 : Yah, yah, yah I think phone, phone will be better.

I_01 : Phone will be better right ok, ok then like let’s say if we try to also do like a paper form I know this is sounds a bit…[Interviewer laughed]

AF_0013 : Paper form very archic no…

I_01 : Yah, yah but because…

AF_0013 : Paper form.

I_01 : You know like for example if we give you some brochure then like you know to moni- to, to track your blood pressure you write down your blood pressure then you mail it to us like that.

AF_0013 : That one is no definitely no! [Interviewer and participant laughed]

I_01 : Ok, ok, ok then a little bit on the interface is it too do you find that the screen too small too big average or ok?

AF_0013 : Average, average just right.

I_01 : Ok, ok what about like the, the font how do you find the font?

AF_0013 : Font, font ok.

I_01 : Then the brightness everything…

AF_0013 : Brightness ok.

I_01 : Ok the how about navigating the app do you find it…

AF_0013 : Navigating no issue.

I_01 : Oh you find it ok.

AF_0013 : No issue no issue because if, if I think for the seniors they have a bit of problem for me I, I ok quite, quite…

I_01 : You’re ok.

AF_0013 : Quite familiar quite familiar. [Interviewer and participant laughed]

I_01 : Ok, ok right no problems. [Interviewer laughed]

AF_0013 : You came across people with problem?

I_01 : Some will have problem yah, yah it’s quite common actually among older people.

AF_0013 : Yah, yah agreed.

I_01 : Ok so like would, would there be any feature or any other than the one you mentioned the pulse one right you know that useful you find that it will be very useful to add to this kind of device…

AF_0013 : I, I…

I_01 : Add on to it?

AF_0013 : The pulse I think if you have a graph on the pulse and then the, the, the, the A, AF I think development of the, the, the situation of the AF is, is it is it ok this time on I mean this type maybe, maybe very, very irregular maybe this type less, less irregular I think we have some idea of these is better.

I_01 : So like…

AF_0013 : AF related.

I_01 : AF related you just feel that this blood pressure is just too not directly at all relevant?

AF_0013 : Not so not so.

I_01 : Ok, ok so other things that are more AF…

AF_0013 : Centred.

I_01 : Centred, and in some information that comes that related to AF directly?

AF_0013 : Correct, correct, correct.

I_01 : Ok, ok then what about like any other like kind of you know things you want to see and into in a device like that you know some ideas, comments that you can that can help us improve on, on something like that. You know that potentially we could expand it to more Singaporeans yah what would be useful you think.

AF_0013 : That’s about all that’s about all blood pressure, pulse…

I_01 : Any other things like more videos more…

AF_0013 : You are talking about the tablet?

I_01 : The tablet so there’s the, the things combined together.

AF_0013 : I think some the tablet of those stories a bit too long…

I_01 : Too long?

AF_0013 : Can cut it short.

I_01 : The video is it?

AF_0013 : The video too long.

I_01 : Too long?

AF_0013 : Can be shorter people not much patience [Interviewer and participant laughed] can cut it short?

I_01 : Is it?

AF_0013 : At least some of them can be halved, compact it.

I_01 : Halved, halved that means it’s a lot cut very big cut you know?

AF_0013 : You know it is quite long, halved it.

I_01 : Cut it’s a half it’s a big cut yah.

AF_0013 : Yah, yah, yah be you can have more, more other stories but the video itself cannot be too long especially those seniors talking and there’s one on this don’t know stroke victim a little bit too long.

I_01 : Was it too slow also?

AF_0013 : Slow and ss- [Interviewer laughed] and slow long and after a while people lost interest.

I_01 : Ok, ok, ok so you…

AF_0013 : Compact it.

I_01 : When you watched did you finished the video or not?

AF_0013 : I didn’t I didn’t I didn’t I didn’t [Interviewer laughed] I didn’t because you see one or 2 you see all almost same stories.

I_01 : All very it seems very similar to you the all the videos?

AF_0013 : No the, the stories look the same.

I_01 : Looks the same ok, ok.

AF_0013 : It looks the same and then yah, yah, yah I, I think maybe get one or 2, 2 stories and plucked in all the features that those others then they these 2 explained already and human being very short attention span.

I_01 : Yup I, I agreed [Interviewer and participant laughed] so I, I mean definitely this is very useful for us to know.

AF_0013 : That a bit long the, the one and the other one the, the cooking that one the…

I_01 : How, how do you find it?

AF_0013 : The, the food that one, food a bit long…

I_01 : Too long ok.

AF_0013 : Too that one is too long and you, you, you can get only of course you get to have the 4 races so cut it a bit short.

I_01 : Ok then the exercise one did, did you watch?

AF_0013 : Exercise one, I watched all exercise one…

I_01 : How did you find it?

AF_0013 : Can’t remember maybe it’s earlier on.

I_01 : Ok earlier on.

AF_0013 : No comment means ok.

I_01 : [Interviewer laughed] really ok.

AF_0013 : The best one is the one the, the, the thing they some the stroke thing…

I_01 : The AF one?

AF_0013 : The AF is the best those very visual…

I_01 : Ok so vi- vi…

AF_0013 : Without that we don’t know with, with that we actually we know luckily that one is in the beginning part so beginning part we have interest to see other videos you know yah if you show all those others first then we have lost interest in the…

I_01 : Ok interesting yah, yah so the sequence also matters the, the way we put the videos also matters.

AF_0013 : Correct, correct.

I_01 : Ok, ok, ok so I think that’s about it thank you for really sharing your, your…

AF_0013 : I, I give you an apple it’s better.

I_01 : Ok thank you very much so I just stopped the recording.

End of interview
