# Supplementary material for: Patient perspectives of the Self-management and Educational Technology tool for Atrial Fibrillation (SETAF): A mixed-methods study in Singapore
Source: PLoS One. 2022 Jan 21;17(1):e0262033. doi: 10.1371/journal.pone.0262033 (PMC8782297; doi:10.1371/journal.pone.0262033)
Supplement: S1 Data — (ZIP) [file pone.0262033.s006.zip › AFP2_IDI_0012.doc]

Participant ID number : AF_IDI_0012

Interviewer Number : I_01

Length of interview: 34.43mins

I_01 : Ok so thank you for taking part in this study…

AF_0012 : Yes.

I_01 : So the first question we want to ask is for how long have you been diagnosed with atrial fibrillation and how did you find out about it?

AF_0012 : Oh, oh ok because one day I got a allergy bad allergy so I go to the emergency then my blood pressure was over 200 so the doctor was shocked that he say machine must no good. So after that asked me to lie down and they think then they say they say you got the diabetes not diabetes [Participant laughed] high blood…

I_01 : Ok high blood ok.

AF_0012 : So you must take the medicine so I said ok I take the medicine.

I_01 : Ok how long ago was that?

AF_0012 : About 14 or 15 years ago quite long time quite long time…

I_01 : Quite long time ago so in the meantime you take any medications…

AF_0012 : Yes, yes all the…

I_01 : Ok this…

AF_0012 : Whatever the doctor gave me so beginning is we try and you know sometime not suitable sometime ok so just like that then is ok now I’m com- quite comfortable, yes.

I_01 : Ok so in the meantime right were there any kind of information where do you get information about say your condition that help you to manage it?

AF_0012 : Yes, television or book or something like that yah.

I_01 : Ok so what, what did you watch like what kind of things like what kind of is it a pro-…

AF_0012 : Program ok because I watched Japanese program yah so the knowledge from yah Japanese TV yes or Japanese book.

I_01 : And ok Japanese book ok what about your say friends and family you get any information from them…

AF_0012 : Yah because my son is a doctor yah so time to time come in check yes, yes.

I_01 : Ok, ok so that will be very directly helpful for you ok, ok then any other ways of getting like information or you know other, other than all these?

AF_0012_helper : Excuse me this is for you.

I_01 : Thank you.

AF_0012 : Because my son is professor know yah so best is ask him and the best is ask hospital “N” because I like hospital “N” yes so always when the come to check up then I will ask or doctor will say that ok this time your blood pressure is the too high so change the medicine or something like that. Yes I will listen to, to all the doctors, yes.

I_01 : Ok just a bit just curious what do you like about hospital “N” like what features of hospital “N” that you like about like?

AF_0012 : Because I think maybe nurse because my husband always go to private hospital, hospital “M” or, or all this thing I don’t really like their nurse yes but hospital “N” nurse is excellent it’s really, really very good yes.

I_01 : In the particularly in the heart clinic?

AF_0012 : Yah heart clinic yah heart clinic…

I_01 : Ok, ok then…

AF_0012 : Because I go to heart clinic he, he go to that’s right I pulled him to the hospital ”N” then he go to his how to say prostate the sexual one the fantastic and he had a bypassed yes so all done by Hospital “N” and it’s really very good. Of course doctor is very good but I, I want to emphasize is nurse is very, very good, yes, yes.

I_01 : Ok, ok in terms of like the you know interacting with you…

AF_0012 : Yes the service and these thing yes it’s really they are fantastic yes.

I_01 : I see ok.

AF_0012 : Compared to the private doc- private nurse yah.

I_01 : Really, even compared to the private I see that’s very interesting ok, ok then the next part will be your overall experience with your this, this study so during this time period how was it like that I mean how’s your routine or the sequence of used like everyday?

AF_0012 : The medicine?

I_01 : The, the, the this tablet…

AF_0012 : The measure…

I_01 : How do you do it everyday?

AF_0012 : Oh you mean this one?

I_01 : Correct the, the blood pressure machine.

AF_0012 : I, I find that [Participant laughed] your blood pressure medicine better than mine because the why because it’s lower I, I everyday monitor my blood pressure you see then I record it yes and the when come to the same day about 5 supposedly if I got 135 then used this one yours is 130. Of course I am happy with yours so I want to buy this one. [Participant and interviewer laughed]

I_01 : Ok so you find that it’s lower and you are happier with it the result particularly…

AF_0012 : Yah which one is true one I don’t know but is always this is lower yes.

I_01 : Ok just wondering because you know the you also used your own blood pressure monitor is the strap the same or different type of strap the strap?

AF_0012 : Mine is brown is it?

I_01 : Ok is it the same type I mean like if you have to do it the same way?

AF_0012 : Yes, yes, yes, yes.

I_01 : The same way ok you know what brand is it just to?

AF_0012 : Brand “B”, “B”….

I_01 : B….

AF_0012 : Yah I think so.

I_01 : That’s the brand name ok, ok, ok we actually we want to take note also because you know it’s very interesting for us why is it different, you know?

AF_0012 : It’s different and so I, I put 2 so I, I used mine first ok so it’s 130 ok then immediately I put this one and is 125 about 5 different.

I_01 : Ok on average.

AF_0012 : Yah higher part but lower part this one is more, more high…

I_01 : A bit more ok.

AF_0012 : Because my lower part is very low so higher or lower doesn’t matter…

I_01 : Doesn’t really matter?

AF_0012 : I only concern about the top part.

I_01 : And that is like for the throughout this time period when you have been taking it it’s always been like that?

AF_0012 : Yes always yah, yah.

I_01 : ok, ok that’s very inter-…

AF_0012 : Because this one, one month or so something like this yah so I told him I want to buy this medicine [Participant laughed] I mean the machine.

I_01 : Ok we’ll take note of it then, then we I maybe let me ask my the technician whose in charge of this and so that I can feedback to you I’ll take note already ok.

AF_0012 : Yes, yes then one thing sorry l want to say that…

I_01 : Yah sure…

AF_0012 : It’s I take blood pressure about said 11 or 11.30 or 12 so the other day the la- lady comes she was afraid I take medicine in the morning so the 11, 12 is my blood pressure to 135 the medicine is not working…

I_01 : Not working?

AF_0012 : This what she think but it’s not I get up at 11 so this you have to consider is I have normal medicine I take the blood pressure medicine blood pressure yet I take the medicine so people do at 7 o’clock but I do at 11, 12 o’clock yah.

I_01 : Oh she did she call you like…

AF_0012 : No first I, I said then he said are you sure that the medicine is working so I said yes because this is the just get up usually get up pressure is higher…

I_01 : Higher yah.

AF_0012 : Yah so that is ok but my time when the doctor want to study 11 o’clock means consider as you take your medicine already yet your blood pressure is this means is quite high right yah so this I, I want to tell you yah so ok that is consider as other people 7 o’clock, yah.

I_01 : Ok, ok so anyway you mentioned about when she, she came here to give the instructions Miss “M” right how did you find the instructions was it ok for you?

AF_0012 : Yah, yah, yah it’s ok yes, yes.

I_01 : Ok, how was it too long too short ok…

AF_0012 : No, no it’s ok.

I_01 : Just right ok was do you think that let’s say if ok somebody came to your house to but did not give you the instruction would you be able to use the device on your own and the app together with the app?

AF_0012 : Yes, yes because they, they will say something, something right, yah yes.

I_01 : Ok so, so it won’t be a problem ok, ok, ok then we want to ask a little bit about the, the features of the, the app did you watch the videos ok, how do you find the videos or which one is your favorite?

AF_0012 : I, I think it’s ok I think it’s ok yup.

I_01 : Ok any favorites?

AF_0012 : Yah ok the exercise one yah, yah.

I_01 : Ok, ok anything that you didn’t like?

AF_0012 : Not really, yah ok.

I_01 : Ok, ok then do you find the videos helpful for you?

AF_0012 : Oh yah I think it’s very, very helpful actually should everyone should have…

I_01 : Ok very, very helpful.

AF_0012 : Yes very, very helpful.

I_01 : In what way?

AF_0012 : Ok let’s say because [Participant laughed] this is you are monitoring right so when your blood pressure is a high in that day ok this, this record go to the Hospital “N”…

I_01 : Yah it does.

AF_0012 : So of course after that, that you are care about your good but if you measure ok a little bit high never mind tomorrow I try all the you know yah so I think it’s very good.

I_01 : Oh ok but ok so like the videos how about the videos?

AF_0012 : Yah videos is good yah ok yes, yes.

I_01 : Ok then what about like you know there are some popped up messages you know encouraging messages how do you find that?

AF_0012 : Yes, yes yah too many same sometimes because it said actually you what did it said you are normal you are normal or normal something like critics yah…

I_01 : How do you find that?

AF_0012 : Very boring. [Participant and interviewer laughed]

I_01 : Ok very boring ok too and too many it came out too much too many times.

AF_0012 : Yah so supposing 120 also say norm- normal ok you are you are ok 135 also say it’s ok so range which how much is the ok?

I_01 : So you…

AF_0012 : Yah so usually doctor said 120 yah but because we are old already so little bit plus, plus minus is 130, 135 usually doctor will say ok so because I had been doing this for a very long time so I can adjust but for new people I think it’s quite boring. [Participant laughed]

I_01 : It’s quite boring and, and also it doesn’t tell you whether is high or low is it like it doesn’t it just say ok is it?

AF_0012 : Yah, yah some something like that.

I_01 : Too, too general ok, ok, ok then what about do you find that what are some of the emotions that, that come up when you when, when you see the messages like…

AF_0012 : Nothing because…

I_01 : Nothing.

AF_0012 : Say you better eat more vegetables or you do some exercise or something like that or some are well done and some something like this right then the one thing very funny is you better go and catch pokemon [Participant and interviewer laughed] so I said I need to go and catch, catch pokemon. Yah so he said why, I said because the doctor said I need to go and catch so I went to catch pokemon yes so…

I_01 : Oh you mean ok so it helps…

AF_0012 : If the sentence is a little bit more and then like yah…

I_01 : Ok more creative ok and more interactive ok, ok sentence more creative more creative would help would help ok then what about like the, the survey does it help you feel?

AF_0012 : Yah, yah I hope everyday.

I_01 : That’s you know the asking some questions to you how do you find?

AF_0012 : Yes, yes, yes because very good the way they because doctor with me everyday so you must be a good girl right [Participant and interviewer laughed] yah so I want to suppose say I want to drink coffee l said this coffee whether this morning I had one I think I think better don’t or something like this. You always have someone here so is actually indirectly helping you…

I_01 : Ok, ok so you…

AF_0012 : So I find that is very good yah I hope everyday I have this one.

I_01 : Really you have awareness of, of, of, of somebody whose like monitoring you ok.

AF_0012 : Yes yah but because you do yourself that means always you have some excuse you know?

I_01 : Yah yup ok, ok what about ok the blood pressure monitor when you take it do you have any feelings that come about when you take your blood pressure monitor because I, I remember when I first I met you at the clinic right you told me that you have a bit of anxiety…

AF_0012 : Yes because even in front of my son yah my blood pressure will go up so someone say the you measure the blood pressure my blood pressure go up so National Heart Centre is worst every time I go there I don’t like the room my blood pressure go up to the 200.

I_01 : So it will affect the environment.

AF_0012 : Affect, affect a lot that’s why I don’t want to do any survey but because you say you want this for the medical used something so I said it’s ok because my son is a doctor so indirectly I think we, we have to understand each other so it’s ok but that’s what I say because this one is lower.

I_01 : Yah ok.

AF_0012 : So I’m happy to do that. [Interviewer laughed]

I_01 : Ok but maybe you are doing it also the different environment in your own home…

AF_0012 : Same here.

I_01 : If, if whole day right?

AF_0012 : Every day I’m here…

I_01 : Yah that’s right.

AF_0012 : I’m this, this place every morning I get up about 11, 11.30 I get the this machine and the other yah…

I_01 : Yah, yah so when you take it right you, you, you feel o- ok I mean like…

AF_0012 : Because the first day was low so I feel not afraid…

I_01 : Oh what if it was not so low?

AF_0012 : Then I think it’s affect.

I_01 : So, so you mean the first time it will affect…

AF_0012 : Yes, yes.

I_01 : Ok first, first reading will affect…

AF_0012 : Yes then but because just come out is I think it’s not that high I can’t remember yah so I said oh ok so not much diff I no need to be afraid yah after that I’m quite happy because this is always lower.

I_01 : What if let’s say you take a first time was ok maybe it was not so it was high then you take again then it was ok do you feel better let’s say like…

AF_0012 : Yah, yah, yah of course of course that means because I may talk to him or I may talk to my maid I think less so some day I think 14 something so I wait sit down then become one…

I_01 : Relax.

AF_0012 : Thirty something and then later on maybe say 132 it’s coming down that so about 2 or 3 times I sent because I’m yah yes.

I_01 : So you take a few times to, to verify it.

AF_0012 : To find that whether I’m ok or not yah then after I sent to you 3 times [Participant laughed] so I said too many already so I used mine and about the same then it’s ok so that is my blood pressure right yah just…

I_01 : Ok so you, you don’t want to use more I mean you won’t take more times because you don’t want to send too many times is it like to us.

AF_0012 : I don’t know whether I irritate you or not?

I_01 : Not real- not really because it’s like in a record right so yah but anyway I just asked we just want to know and asked yah.

AF_0012 : Yes, yes but you supposing if I have to take more I will take more yah.

I_01 : You don’t mind?

AF_0012 : I don’t mind yah yes.

I_01 : Ok, ok that’s great ok then the we want to ask do you know that there’s a number on the on the on this it’s over here right there’s a number…

AF_0012 : Yup, yup number…

I_01 : Do you aware of it throughout these time period like that you can call?

AF_0012 : I, I yes they said if anything you, you call, yes.

I_01 : Did you try calling did you?

AF_0012 : No, no.

I_01 : Didn’t ok!

AF_0012 : Because not, not particular thing if said supposing suddenly very high and I can’t find the reason then I will ask yah but it’s a I think it’s quite ok.

I_01 : If do you find that it’s having a number there is helpful or you prefer not to have any number?

AF_0012 : For me, helpful.

I_01 : Helpful ok, ok is there any reason why is you think it’s helpful the number? [Participant and interviewer laughed] I know ok I know what you mean ok anyway the which subsequently do you see any doctor in the meantime to like say when you see a doctor you bring the results to, to, to see the doctor with…

AF_0012 : No, no.

I_01 : Not, not yet ok, ok like let’s say if you have the chance will you do that like…

AF_0012 : Yah because usually doctor asked me to bring the, the things but because if it’s too high I’m afraid I go to see my son.

I_01 : Ok so you, you will go to your son I see…

AF_0012 : Yah so hospital “N” the medicine is not original, generic.

I_01 : It’s generic yes yah.

AF_0012 : Yah so I will change it with his medicine his original so whether got any different or not I’m not very sure but I will change I will all go to him and get all original yah then I take I was one or 2 weeks yah, yah.

I_01 : Ok, ok then I am going to ask about the interface of the of the tablet was it too how do you find the size of the tablet the screen?

AF_0012 : It’s ok yah.

I_01 : It’s ok, ok is that too small or big you know?

AF_0012 : For me I’m ok because I take 5, 6 tablet together so, so it’s size is ok.

I_01 : Sorry I don’t mean the tablet I mean the, the this that this…

AF_0012 : This one sorry, sorry yah…

I_01 : The monitor yah, yah.

AF_0012 : Monitor I think it’s ok…

I_01 : It’s ok.

AF_0012 : This is like a apple’s small one I, I have one this one yah…

I_01 : Is it?

AF_0012 : Yah so same size yah.

I_01 : It’s ok, ok is it too small the…

AF_0012 : For me it’s ok for me it’s ok.

I_01 : It’s ok, ok then what about like the font is it the wording is it too…

AF_0012 : Wording is very big so…

I_01 : Very big ok so ok.

AF_0012 : Yah so it’s ok yah, yah.

I_01 : Ok, ok and then what about like the brightness?

AF_0012 : It’s ok.

I_01 : It’s also ok, ok, ok so let’s say if we have a, a few options for you we have the phone we have the tablet which is the monitor you have a desk top computer and you or some other device what will you prefer to use of all these all that…

AF_0012 : I used this because I don’t know how to use computer.

I_01 : Ok, ok but what about like phone or something…

AF_0012 : Telephone ok yah, yah.

I_01 : Ok like hand phone it’s ok?

AF_0012 : Yah, yah telephone it’s ok.

I_01 : It’s ok with you?

AF_0012 : Yah so some of these program especially exercise one I will I video taped it.

I_01 : Video taped how do you do it?

AF_0012 : I just because they are moving so I just video, video it yah, yah…

I_01 : Ok, ok and then…

AF_0012 : Then I no need to do one whole session because the, the boy said you must put the leg up here or, or down so I just ok then go to another one, yah.

I_01 : Ok do you find the video like maybe a bit too, too long?

AF_0012 : Yah I just think so yah because the same one if you have to 10 times they do 10 times I think is not necessary yah maybe 5 times is ok.

I_01 : You mean the exercise duration you find it too long?

AF_0012 : Too long because you are doing the same thing this one please do 10 times so but one or 2 times when you see it maybe you forget so maybe said 3 or 4 time or 5 times ok but after 5 times I’m not watching because I already watched. Yah so I think is this one must do 10 times I think this is a bit for me a bit too long.

I_01 : Too long ok so you prefer some, some shorter kind of routine exercise.

AF_0012 : Yah so emphasize on exercise and times how many that’s ok but not necessary to do 10 times you do the same thing again and again and again [Participant and interviewer laughed] then I have to wait until these 10 times finished.

I_01 : Ok, ok that’s very it’s quite interest- so 10 times too long 10 times too long ok then if let’s say we have a paper form right because we also are con- like maybe for really you know some people they prefer like paper form would you want to use something like, like paper form? In, in terms of like for example we, we, we give you some brochures and then for information and then we also give you a piece of paper to record your blood pressure and then you…

AF_0012 : Then the this one give it to you.

I_01 : Sent it back to us yah would you prefer this or you prefer electronic form?

AF_0012 : For me this is easier yah…

I_01 : Ok, ok I see.

AF_0012 : But because sorry you recording you can stop.

I_01 : No it’s, it’s ok.

AF_0012 : This is just a [Inaudible segment] actually I…

I_01 : Oh you ok…

AF_0012 : Yah so I put so the time is always 11 something yah because I get up so the my weight and I write what I eat so this one is a so supposing this is ok this is not alright right yah so then I will check say what they eat and then maybe night sometime eat something, something [Participant and interviewer laughed] rubbish…

I_01 : And your and your…[Interviewer laughed]

AF_0012 : Is like oh yah so I have been I have been doing this for and 10 over year yah so…

I_01 : Wow it’s very, very good it’s really, really good.

AF_0012 : It’s, it’s yah everyday almost everyday yah, yah.

I_01 : Very, very I think this is really good happy and, and if let’s say we have some, some feature right we, we can put it inside this app we think it’ll be helpful to people or not do you think it will help other people also?

AF_0012 : I think so I think so because you see when you are using this one is run through it again ok now I’m I usually sometime got very bad one but I checked yes I drink coffee I drink supposing I eat a cake or something, something but still next day the blood pressure is ok. So today I cut the coffee don’t drink and the food also careful next day blood pressure high…

I_01 : Very high.

AF_0012 : So sometime I just don’t know.

I_01 : [Interviewer laughed] So it’s very like varies yah up and down and it’s not necessarily depending on the diet could be other thing also.

AF_0012 : I say I think basically is diet.

I_01 : Diet ok, ok.

AF_0012 : Yah basically I just think it’s diet yes, yes.

I_01 : Ok, ok sure that’s why you have this record, ok.

AF_0012 : Yes but someday because is at night I cannot sleep yah because not tired enough so then maybe lack of sleep also affect the blood pressure so I, I just lie down everything what is that…

I_01 : This is very, very good yah.

AF_0012 : I cannot sleep or where I go so then I know where I go what I eat I know it’s that so oh maybe this food is this sort of thing or something like this yah, yah.

I_01 : Ok, ok and it helps you a lot.

AF_0012 : To see then it said…[Participant and interviewer laughed]

I_01 : Then, then, then you then you know what to do yah that is very good that’s very, very good yah ok, ok then a few more questions let’s say we introduced this device to you when do you think will be the best time you know throughout this time period when you have atrial fibrillation right. When will be the best time to introduce it into I mean to you like at the beginning when you first know knew about it or in the middle somewhere in the middle…

AF_0012 : Ok sorry I don’t get it?

I_01 : In terms of like because we are thinking of like…

AF_0012 : Like what beginning means so when?

I_01 : Beginning of when you were diagnosed because you know we want…

AF_0012 : Yes, yes, yes because beginning we don’t know…

I_01 : Yah correct yah.

AF_0012 : Right some people but I mean I don’t know so suddenly say this take medicine yes I, I take medicine I, I do exactly what the doctor said but as I told you about 6 months I have to change and throw away all the medicine and this thing that thing yah so if you have this one from beginning then we learn. I think is I think is very…

I_01 : It’s very good.

AF_0012 : Yah it’s good.

I_01 : Would you think that it will create a bit like anxiety for people I mean like because they when they first knew about it right then they keep track of it sometimes it may be high for them and do you think that it will affect them affect people like at the beginning you now?

AF_0012 : Yah so that I think is if they have the place that put the weight how many kg yah then you have to have a weighing machine so I don’t know but it’s if you put that that means you are too fat because usually say that thing become blood pressure lower right so then you may be advise that you are too fat. Yah so eat less or something like that yah I think that is the one so but beginning I think is very helpful because…

I_01 : It’s very helpful.

AF_0012 : The first, first thing we don’t know I go and read book but actually don’t really understand so it’s a cut salt so ok I cut salt but it’s the not, not much yah so what kind of things…

I_01 : What to do right?

AF_0012 : Yes and sometime I think this is alright because when you eat this is not salty so it’s ok but how, how to say hidden salt which we don’t know yah then how come I eat this one and my tomorrow my blood pressure go up and this one look very salty but ok I try then, then it’s ok. Yah so I think is the beginning is the better then we can…

I_01 : Beginning can learn.

AF_0012 : We can study can learn yes, yes.

I_01 : Ok, ok, ok it’s very interest it’s very useful for us ok then like finally do you have any like suggestions you know on the kind of features that we can add on to the tablet?

AF_0012 : Yes I suggest.

I_01 : Yah great that’s great.

AF_0012 : Because, because I have this I get the finger infection yah because I do the gardening yah so the doctor asked me to take this medicine but they said is no side effect but I have side effect with this medicine then little bit the leg swollen. Yah and the my toilet yah a little bit hard ok that is the side effect but bring down the blood pressure.

I_01 : Ok so you…

AF_0012 : So usuaI- I have to take 4 tablet so I take 4 tablet morning 2 at night 2 so I think you I mean that you or your doctor or this lab people I think can study what is inside some of the medicine I think is the very good for lower down the blood, blood pressure about 20 different…

I_01 : Ok so you, you mean what do you, you mean that this, this medicine they affect your…

AF_0012 : Blood pressure…

I_01 : Your blood pressure…

AF_0012 : Pressure yah.

I_01 : And, and actually what, what I what I meant by suggestion was that you know suggestions that you can give me about the, the, the your tablet that we can improved on, on the functions you know?

AF_0012 : No, no that’s, that’s…

I_01 : How…

AF_0012 : What I’m, I’m answering…

I_01 : If…

AF_0012 : Because I’m taking this blood pressure medicine is good is good but when I take this one another average 20 down…

I_01 : Did you tell your doctor about this like what you are taking the other…

AF_0012 : No because I know you are coming yah so I want to talk to you then you, you can because my English is no good…

I_01 : Sure, sure, sure I can I can feedback yah…

AF_0012 : So you can talk to, to the doctor…

I_01 : I feedback for you.

AF_0012 : Of course I don’t know what is inside…

I_01 : Let me write down the name of this requests for from for doctor advise sporos- s.p.o.r.a.n.o.x this is for the infection is it?

AF_0012 : Infect is yah [Participant laughed] this finger for the finger.

I_01 : Ok for infection for infection…

AF_0012 : Fungus is it this is I think bater- what is it?

I_01 : Ok, ok so you 2, 2 times…

AF_0012 : Twice…

I_01 : Twice daily for one week twice daily so have it’s, it’s already one week already.

AF_0012 : Because I can’t take because my leg swollen so I talk to the doctor…

I_01 : Caused swollen caused swollen leg swollen leg…

AF_0012 : Yes but other people don’t, don’t report I’m, I’m the one.

I_01 : Times ok 2 times per week, no 2 times per day…

AF_0012 : 2 times per day yes, yes so now I’m taking only one.

I_01 : You taking one?

AF_0012 : One yah then it’s ok my leg no swollen no, no anything.

I_01 : Ok taking one now your doctor do you have his the name of the doc the doctor?

AF_0012 : Doctor is this “M” who is the clinic…

I_01 : I mean the one at the you so you want me feedback to your is it heart clinic the doctor?

AF_0012 : Yah so see whether anything can find ok supposing [Participant laughed] if say this is contained a, b, c, d, yah then because my blood pressure medicine doesn’t have “a” supposed so if put a little bit these are simple idea just a little bit of “a” put in so it maybe help the medicine of course I don’t how they do. But it’s a maybe the blood pressure can come down.

I_01 : Ok I, I understand what you mean ok, ok.

AF_0012 : Yes, yes so this is…

I_01 : Like caused the…

AF_0012 : Lab, lab study already not, not, not doctor I said yes, yes.

I_01 : I see I see ok, ok I understand ok, ok so we can possible reduced blood pressure reduced possible reduced blood pressure, possible ingredient, ingredient ok can I I’ll feedback to the doctor.

AF_0012 : So you can see some of them that day my blood pressure is 106 or one zero something it’s not because I’m good so proba- no because the side effect of this one.

I_01 : So actually you, you if you, you feel very strongly that this has this really affected your blood pressure lowered down.

AF_0012 : Blood pressure lower, bring down about 20 yes but 20 I got side effects so now I, I get one then maybe about 5 or 5 to 10 bring down yah so must be something content inside [Interviewer laughed] must be good for blood pressure I just think. Yah but, but simple…

I_01 : Ok yah I know I know what you mean.

AF_0012 : Yah, yah thinking so maybe not to do at all I don’t know.

I_01 : Because I mean the thing is that this might af- might be also they have other ingredients right yah, yah, yah so yah…

AF_0012 : Yah, yah that’s right that’s right so I don’t know what they said that yes, yes.

I_01 : So that one maybe a pharmacist will be more, more better to look at that and anyway about regarding the also like go back to a little bit to the app any other suggestions any other comments you know thoughts that you feel can be we can do to help you improved the experience or not?

AF_0012 : I think because exercise part is quite good and, and what else you have, [Participant laughed] yah.

I_01 : Ok exercise, cooking…

AF_0012 : Cooking…

AF_0012 : We have the, the, the…

AF_0012 : Cooking I didn’t see, cooking?

I_01 : You, you didn’t yah there also cooking one a cooking video I think I think I can show you and…

AF_0012 : Oh that means I, I missed.

I_01 : Ok I think this we have a cooking video…

AF_0012 : Oh ok then, then it’s good…

I_01 : It’s good…

AF_0012 : Then how to cook how to eat is it, this is very good but I didn’t I didn’t see I never know there is.

I_01 : Should be inside yah anyway do you find that this study is this 6 weeks is it too long you find it too long?

AF_0012 : No I think it’s ok.

I_01 : Ok it’s ok, ok do you find that the, the, the navigating the app right was it difficult to navigate through the different stages?

AF_0012 : It’s ok because the she asked me not to switch off so one whole day “Druu… druu… druu…”.

I_01 : You so that, was that irritating?

AF_0012 : Irritating. [Participant and interviewer laughed]

I_01 : Ok, that’s very good.

AF_0012 : So I switched off yah then when I want I switched on but she is afraid that I don’t know how to switch on so I said one whole day “ druu… druu… druu…” yes.

I_01 : Because this is not the first time we hear this also we have other participant, participants that tell us that the vibrating was very irritating and very…

AF_0012 : Yah, yah, yah because very often and then quite, quite noisy.

I_01 : Very noisy, noisy and, and it’s not it’s also vibrating when you are not doing anything?

AF_0012 : Yah just, just put there so until I’m so afraid that I have to put at the kitchen so that because the kitchen then is more people walk here and there yah because I’m afraid too hot or too cold or what you see yah.

I_01 : Ok so you put it in the kitchen…

AF_0012 : Yah because the kitchen is not using the yah…

I_01 : Ok this is very interesting because it will affect you’re the way you where put the things also the, the…

AF_0012 : Kitchen is cooler…

I_01 : The tablet.

AF_0012 : In my house kitc- the this my, my kitchen is cooler but I got the one big kitchen for the cooking yah so no fire no anything so it’s cool yah.

I_01 : Ok, ok, ok you know it’s not…

AF_0012 : So where is the cooking…

I_01 : The video is I think it’s yah it should I think it cook right and eat smart for healthy I think this the one yah…

AF_0012 : Oh ok I saw I saw yah.

I_01 : How you oh you, you watched this oh ok, ok…

AF_0012 : I saw I watched it this one yah, yah…

I_01 : So you also watched it ok.

AF_0012 : I thought that really cooking yes, yes.

I_01 : Ok, ok so you don’t find that this was like this was not really like cooking, ok I just pause for awhile…

AF_0012 : Yah I think could be a little bit more how to say detailed not, not…

I_01 : More, more detailed…

AF_0012 : Yah like not go to supermarket [Interviewer laughed] and then this is the one this is healthy ok you choose this I think everyone know already don’t you think?

I_01 : Maybe a mixed not every I, I supposed like you know there’s a wide range of people some will be more well informed some maybe not so aware…

AF_0012 : Yah so…

I_01 : But detailed in what sense like what kind of details you mean?

AF_0012 : Ok suppose say milk ok, full cream milk and the low fat milk just so usually said the low fat milk is better yah so instead of you choose the, the full milk full cream you choose but actually there is a skim milk yah so it’s there is one more.

I_01 : Ok so a little bit more detailed.

AF_0012 : Little bit more information, yah.

I_01 : Ok, ok, ok in, in terms of the kind of like the range the range ok what about like cooking do you think that there should be more details about how to cook you know like the step one step 2 step 3…

AF_0012 : This is because the Singapore lady they don’t cook…

I_01 : Yah not much yah.

AF_0012 : Yes so may not be necessary but for us Japanese yes we, we cooked so yes…

I_01 : It’ll be very useful.

AF_0012 : Yes I, I did I buy a lot of books for cooking so the way cook also yes not only the cooking yah.

I_01 : On selection choices food selection choices ok, ok can right so any other, other comments that you want to add on to this experience that you had you know how you feel?

AF_0012 : Yah I think is everything is ok but as you say it’s good because this one a bit too don’t know how to said like that kinder gardens [Interviewer laughed] under yah you have to teach little more to the people means more details. You, you, you have a choice of like example is the skim milk or suppose say the corn flake ok it corn flake is ok but the corn flake contained sugar so those because this related to the high blood and diabetes all these so in instead of this corn flake maybe either cut half or you know mixed with brown flake or some whereby it’s tastier so more, more detailed.

I_01 : More detail right and also make it more interesting maybe?

AF_0012 : I think those people who, who want to watch yes, yes because I study yes so I see this cooking this part is quite boring yah, yah.

I_01 : Ok, ok we take note of it definitely.

AF_0012 : Yes, yes, yes but for other people I don’t know because those who don’t cook many people doesn’t cook so they have no choice yah.

I_01 : But we want to really still hear from you because also like you know what you think about the video that’s lacking for yourself I think it’s very important.

AF_0012 : Yes, yes, yes the, the even the cooking oil also yah which, which oil…

I_01 : Many types.

AF_0012 : Many types yah then yah just little bit more try to be the, the not, not kinder garden [Participant and interviewer laughed] yah.

I_01 : Ok, ok it’s too simple ok.

AF_0012 : Yah, yah too simple I, I feel but maybe other people don’t feel yah because I do a lot of cooking yah…

I_01 : Ok right ok.

AF_0012 : Then they compared to the Singapore food and the Japanese food Singapore food too much oil too much meat.

I_01 : Yah agree I agreed. [Interviewer laughed]

AF_0012 : And too much meat so actually all, all these thing I said all just yah.

I_01 : Ok, ok so anyway thank you so much for sharing.

AF_0012 : You are welcome you are most welcome.

I_01 : It’s very useful for us and...

AF_0012 : Most welcome

I_01 : Thank you let me stopped the recording.

End of interview
