# Supplementary material for: Patient perspectives of the Self-management and Educational Technology tool for Atrial Fibrillation (SETAF): A mixed-methods study in Singapore
Source: PLoS One. 2022 Jan 21;17(1):e0262033. doi: 10.1371/journal.pone.0262033 (PMC8782297; doi:10.1371/journal.pone.0262033)
Supplement: S1 Data — (ZIP) [file pone.0262033.s006.zip › AFP2_IDI_0011.doc]

Participant ID number : AFP2_0011_180515_1931

Interviewer Number : I_01

Length of interview : 29.47 mins

I_01 : Ok so thank you for taking part in this study so for how long have you been diagnosed with Atrial Fibrillation irregular heartbeat and how did you find out about it?

AFP2_0011 : I think August last year.

I_01 : Ok, ok how did…

AFP2_0011 : How did diagnosed it…

I_01 : How did you find out find out, yah?

AFP2_0011 : Because I went to my company, company doctor to do a annual ECG they found irregular heartbeat then they asked me to go to poly so I scheduled for C poly with they too found the irregular heartbeat with then they refer to hospital N cardio heart centre. So they do again in August the doc- the cardiologist do a ECG again and they found now that I have irregular heartbeat under AF is there then they scheduled me in March I think March or February for do the echo so from there.

I_01 : Ok, ok did they give you any like medication to take and…

AFP2_0011 : No because I still on the normal hyper tension tablets so they does nothing.

I_01 : Oh ok the hyper tension tablet will is it the, the one will affect the blood pressure one?

AFP2_0011 : Yah to lower down the…

I_01 : To control it?

AFP2_0011 : Yah controlled yes correct.

I_01 : Ok, ok so you didn’t you are not taking like warfarin are you?

AFP2_0011 : Not then…

I_01 : Not yet because you it will clashed with the…

AFP2_0011 : Yes correct…

I_01 : The, the blood pressure one that you take.

AFP2_0011 : No, no, no because they have not seen the do the echo once they saw the result out in March then they found the from the result they introduced the I, I never take warfarin I take this asobat…

I_01 : Ok, another different type one?

AFP2_0011 : Yah blood thinner so that’s it.

I_01 : So, so wait for the results first.

AFP2_0011 : Then they adjust my medication.

I_01 : Ok so in the meantime right do you like get any sources of information to manage your AF or not how, how do you find out more about it?

AFP2_0011 : Through the internet, you tube, goggle…

I_01 : Ok did you do that?

AFP2_0011 : Yes then after that you give me the tablet the tablet will have a lot of survey…

I_01 : Correct, correct yah.

AFP2_0011 : Then video right? So it’s a similar thing.

I_01 : Ok so you, you, you have been doing this before the study?

AFP2_0011 : Yes correct.

I_01 : Ok, ok do you find it helpful and you know useful or not?

AFP2_0011 : Yes, correct.

I_01 : Ok what kind of websites by the way just to just to…

AFP2_0011 : Just normal google kind of thing then I just typed atrial AF then will come out then what are the ways everything to visit.

I_01 : Do you pay attention like the creditability of these websites you know because you know there are so many different websites they all like claim different things you know…

AFP2_0011 : Yah most of it is like I think not locally I think is more in US, UK those web yah those medical website yah so but basically when I compared to local it’s still the going to the same direction so, so…

I_01 : Ok, ok, ok so you also looked at the local ones, ok, ok.

AFP2_0011 : Yes, correct like Singapore Heart center I mean Hospital S heart center hospital N heart center so basically is telling all the same to the same direction so…

I_01 : So you compared lah, ok, ok.

AFP2_0011 : Yah so it’s the same so I’m not worry unless people say yah, yah so that’s it.

I_01 : So that’s how you find out in the consistency of it?

AFP2_0011 : Correct.

I_01 : Ok then like you know does your friends and family like help you and do you get any like you know advice from them or in any way…

AFP2_0011 : On AF?

I_01 : Yah and also your…

AFP2_0011 : My wife my wife and my sister my sister is a nurse in hospital N so they somehow helped me along the way to get more info and what precaution to take then that’s it.

I_01 : Ok, ok so in this period of the study how was it like on a typical day your sequence of used how do you normally use the device what do you do first you know when do you do it also?

AFP2_0011 : Normally is before I go to work is between normally I take the reading once a day right, so I will do it before ss- leaving work before 7.

I_01 : Is that how you do it like everyday say the same…

AFP2_0011 : Yes, yes most of it 90 percent will be consistent before yah at the first stage I think the first one month I do it after work 6 to maybe 7 pm. Then the latest part of the survey is about early in the morning so because I see the different is the reading is still there…

I_01 : It’s…

AFP2_0011 : The same yah.

I_01 : Ok, ok how do you find the like the frequency like you have to do this everyday do you feel is it ok is it…

AFP2_0011 : Whether comfortable doing or…

I_01 : Yah comfortable the, the…

AFP2_0011 : Ok.

I_01 : This period this during this period?

AFP2_0011 : Only the only problem I face is when I, I like I always go overseas so the incon convenience of bringing the thing I’m worried that put in the hotel lost it along the way so that’s the only thing, but for the doing the survey I only twice I think when I go going I mean overseas…

I_01 : Overseas ok, ok.

AFP2_0011 : Yah so not much this one.

I_01 : Ok then what was the fav- like what were, were the, the different features that were easiest for you to use you feel?

AFP2_0011 : The tablet is it?

I_01 : Yah the, the tablet the app anything?

AFP2_0011 : The video is ok because the video and survey is just incoming kind of the only face problem I face is the connection…

I_01 : Ok the connection what, what about the connection?

AFP2_0011 : Because it’s connected 4G right or bring data right…

I_01 : Yah data.

AFP2_0011 : So when I take the reading it will go in instant instantly some…

I_01 : You mean the it doesn’t go into the tablet?

AFP2_0011 : Yes I although I refreshed a few times it will not come in so I sometime because of time constraint I just have to leave it at home then at end of the day I on it back and then…

I_01 : You find that…

AFP2_0011 : Sometime sometimes is there sometimes is not there then…

I_01 : How, how often does this happen?

AFP2_0011 : Maybe I can say 50 percent of it.

I_01 : Really? That’s quite a lot actually.

AFP2_0011 : Yah so I did bring up to the, the one who give me but as time goes by it came but maybe one week after so the reading although it’s good is it doesn’t it the reading were still there only it’s not instantly…

I_01 : Not instant it’s ok also the one is lacked time.

AFP2_0011 : It could be connect connection problem or what that’s all.

I_01 : Actually it’s a blue tooth device so it should be connecting like even without the 4G and all that.

AFP2_0011 : That’s why so it’s near then I don’t know sometimes even one hour it won’t doesn’t refresh…

I_01 : Oh really?

AFP2_0011 : It doesn’t it could be an software…

I_01 : Usually the, the delay is you mentioned up to one day but the average was, was how about how long?

AFP2_0011 : Maybe in a day.

I_01 : So average was actually a day.

AFP2_0011 : If morning yah in morning if I take the, the reading right by the time I reached home it should be there normally it’s there.

I_01 : So you have to wait until you come back then you can see it.

AFP2_0011 : Yah because I leave it here I never bring along so…

I_01 : Ok, ok, ok, ok yah so ok then were there any features that were like difficult, difficult I mean this is a problematic…

AFP2_0011 : To ok…

I_01 : One right so like were there anything that were difficult to for you to use in the this app…

AFP2_0011 : No, yah nothing.

I_01 : Ok what about like ok you mentioned the video right were there any did you watch all the videos?

AFP2_0011 : Yes…

I_01 : Ok how do you find the videos?

AFP2_0011 : Most of it is educational just good info to me but I think for the how many weeks 6…

I_01 : 6 weeks.

AFP2_0011 : 6 weeks I don’t think there’s a lot maybe 6 or I think 6 or maybe 8 less than 10 videos right, so…

I_01 : Yah you but you watched everything?

AFP2_0011 : Yah, yah because there’s a popped up I just clicked and during my free time and just watched.

I_01 : Any favorite videos?

AFP2_0011 : Most favorite video, no but I more you know norm normally I focused more on the stroke wise they how AF will if I never control my AF whether blood clogged whatever so I now I know why the doctor is it introducing blood thinner in order to safeguard me from getting a stroke. Because the risk is there so from the video I constantly know why, why they do that why they introduced because as far as I’m concerned I don’t want to be on [Participant laughed] medication a lot right so I now I know why.

I_01 : Ok, ok and this one is the information video right then there’s also like other videos like cooking video…

AFP2_0011 : Yes correct yah correct.

I_01 : Exercise videos how do you find those?

AFP2_0011 : Very general.

I_01 : There are very general…

AFP2_0011 : Yah, yah correct.

I_01 : They do you think that it change your the way you like your habits and your behavior in a way?

AFP2_0011 : Not from the video not really because before that before seeing the video we already have the basic knowledge how to I mean and I already see the dietician so basically we know how to change my lifestyle, exercise and that’s all.

I_01 : Was it too long the video or too short?

AFP2_0011 : I think too long.

I_01 : Too long [Interviewer laughed] ok you think too long.

AFP2_0011 : Yah especially the exercise yah it, it could be doesn’t interest me that much because I already know maybe like maybe for the like more on AF then it will attract me more.

I_01 : So you, you’ve you found the video a bit too long and...

AFP2_0011 : Correct.

I_01 : Ok would you want to see like more like you’ve mentioned that you know you’ve already know all these things so like if there were like more videos would you want to watch it I mean watch…

AFP2_0011 : Yah correct.

I_01 : Other like on other aspect of, of you know that can help you?

AFP2_0011 : Yes, maybe yah.

I_01 : Ok what, what kind would you like to see like perhaps that will interest you?

AFP2_0011 : More on the diagnosis itself maybe.

I_01 : Ok, ok diagnosis means in terms of the…

AFP2_0011 : Let’s say if the survey the, the thing on AF right more on AF then people will more will be more keep I mean the knowledge is there yah.

I_01 : Ok, ok so that will be very useful with you?

AFP2_0011 : Yes correct.

I_01 : Ok, ok then, then you also mentioned there were you saw the popped up messages right like the encouraging messages the diet advise how do you what were the feelings that come out when you when you when you saw these?

AFP2_0011 : Doesn’t affect me [Participant and interviewer laughed] personally just a brief like what I know it’s like motivation kind of thing but it’s just a brief keep up the good, good work, yah.

I_01 : Yup, yup do you like maybe sat more satisfied or happier you know when you see it or maybe no not you know…

AFP2_0011 : Gen- like, like I said generally doesn’t affect me much, yah correct.

I_01 : Doesn’t really affect it has no it doesn’t really impact you in any way…

AFP2_0011 : Yes correct.

I_01 : Ok then was it coming up too much you feel or like too little or just right?

AFP2_0011 : I don’t know whether because every reading will come with one message, correct?

I_01 : Ok yah.

AFP2_0011 : Saying you whether your reading is at accepted range then after that it’ll give you a motivation kind of thing encouragement so I don’t know maybe the whole street is too [Participant and interviewer laughed] bordering maybe…

I_01 : Oh really?

AFP2_0011 : Like maybe for the first 4 weeks ok but it’s popping up the same thing it’s like a system generated like…

I_01 : So you, you also noticed that only.

AFP2_0011 : Correct it’s like the same word the same so maybe if you want to do that more encouragement maybe I don’t know maybe different…

I_01 : Different yah.

AFP2_0011 : Way first 4 week is different second 4 week is different maybe so people will not…

I_01 : Changed the wordings you know.

AFP2_0011 : Yah because normally I first 4 week ok I just do it the second [Participant and interviewer laughed] the next 4 week I just delete, delete, delete because I know it’s the same. [Participant and interviewer laughed]

I_01 : Ok, ok so it’s very it kind of become too predictable.

AFP2_0011 : Yah correct I already expect what to be written there [Participant and interviewer laughed] because when I see the reading I know because I know what is the range right the blood pressure is so I know the next reading will be at a accepted range.

I_01 : Ok so very predictable right?

AFP2_0011 : Yah correct.

I_01 : Like you kind of like after a while you don’t really border so much?

AFP2_0011 : Yes correct, yah sometime even I, I never see the message I instantly I wait 3, 4 day then I delete everything [Participant and interviewer laughed] flood inbox email inbox.

I_01 : Yah, yah like a bit like spam messages like that…

AFP2_0011 : Yah correct. [Participant and interviewer laughed]

I_01 : Ok, ok then ok what about the survey do you that how do you find it the survey?

AFP2_0011 : Survey…

I_01 : Yah was it just right…

AFP2_0011 : You’re talking about the survey that is not that not the one asking you how are you right the, the one with the detail…

I_01 : Yah correct the more details more questions about that one did you do them?

AFP2_0011 : Ok yah I do them.

I_01 : Ok how do you find them?

AFP2_0011 : Most of the question is correct I mean what they are asking is what I feel generally although a few 2,3 questions I don’t understand because…

I_01 : Why?

AFP2_0011 : Maybe I’m not that especially on AF when they go detailed into AF I will sometime I answer not sure…

I_01 : Ok not sure ok.

AFP2_0011 : So but as like healthy lifestyle, whether your mood because it really correct there’s a change I can feel that what they asked is that it’s in me…

I_01 : Ok, ok it’s relevant to you.

AFP2_0011 : Example they asked me whether it will affect your relationship with your family member I feel the other side because now with AF I think I feel like I more temperamental like easy to… yah…

I_01 : Ok, ok so, so that, that part the part about especially when asking about your relationships it really helped a lot.

AFP2_0011 : Yes correct because last time I was am like jovial person always laughed make jokes but when I was the when I was informed I got AF I tend to be quiet think about the sickness then so it will I think people around me sees that, yah.

I_01 : Ok, ok and you feel that these kind of questions are very helpful to you.

AFP2_0011 : Yah and is relay to relay to me [Participant laughed] so I think so I, I for it my take is whoever have AF if they got if they popped up this question so it’s a real feeling that a AF person goes through. So, so I just control the try to not to think of more of it, yah.

I_01 : So, so more of these kind of questions you it’ll be much more helpful.

AFP2_0011 : Yes correct, to guide along whether how to address it I think yah the question maybe the video back to the video I think you is best like you tell someone the patient how AF the risk everything right?

I_01 : Yup, yup.

AFP2_0011 : I think it’s best so the video how people to overcome it because like, like me example I feel there is a change in my mood or whatever then the vi- video will come in how we manage that video. Because we got people managed exercise we got video to manage food, dietician healthy lifestyle, eating or ever but there’s nothing to control your emotions…

I_01 : Oh ok so more on the psychological…

AFP2_0011 : Yes correct.

I_01 : Like parts of it, that’ll be very helpful to you?

AFP2_0011 : Yah I don’t see that any video come in from there angle you know.

I_01 : Yah not much from there, yah.

AFP2_0011 : Yes correct so at least the person can you know ok I f I tempted what just fit do something watched TV or what to just not to you know, yah.

I_01 : Ok so on, on coping your emotions like that ok, ok.

AFP2_0011 : Yes correct because people will change when someone know…

I_01 : That’s right.

AFP2_0011 : Yah you got sickness whatever being serious or not serious yah how to manage it yah not to affect others.

I_01 : Ok then about the interface how do you feel about the size of the screen is it just right?

AFP2_0011 : Ok fine yah just right.

I_01 : Ok and then let’s say we have a diff a few different kind of devices we have the tablet we have a phone and then we have a desktop computer and perhaps other kind of electronic devices which one would you like to use of all these?

AFP2_0011 : If you asked me, phone.

I_01 : Phone…

AFP2_0011 : Because it’s with you every time 24, 7 right and I if they can move forward without issuing a tablet to the patient because of I think inconvenience whatever right, I think just sent them a link user ID and password ask them to go internet…

I_01 : You…

AFP2_0011 : I mean to go a website…

I_01 : A desktop…

AFP2_0011 : A link then from there they just…

I_01 : Do it…

AFP2_0011 : Yes correct...

I_01 : From there, ok.

AFP2_0011 : Instead of like because it’s very…

I_01 : Instead of very…

AFP2_0011 : Now people nowadays people unless the apa [Means “what” in English] old generation I’m not sure but if old generation I don’t think they can use the tablet…

I_01 : That’s why yah [Participant and interviewer laughed] yah.

AFP2_0011 : Use the tablet if unless the kids or whatever assist them yah.

I_01 : Ok, ok so let’s say because we also like are considering it for like for all, all ages also we have a paper form right would you like to use a paper form like paper form in the sense that ok, you record your own blood pressure everyday and then you send to us. Or then we, we sent you brochures for like information you know about AF…

AFP2_0011 : On a daily basis?

I_01 : Regularly not, not maybe weekly.

AFP2_0011 : If with that I, I preferred tablet or phone for me. [Participant and interviewer laughed]

I_01 : Ok maybe you preferred the tablet.

AFP2_0011 : Yah correct those yah…

I_01 : Ok if…

AFP2_0011 : Because it’s very instant just add, add within 5 minutes or 5 to 10 minutes I just record yah.

I_01 : Ok so if, if it’s not daily it’s also you preferred it on the…

AFP2_0011 : Yah correct.

I_01 : Electronic, electronic.

AFP2_0011 : More convenient.

I_01 : Ok, ok more convenient ok so like Miss M she came and then she passed you the device like how would did she give you any instructions on how to use it?

AFP2_0011 : Yah he guide me along how to use the tablet and… yah.

I_01 : Ok how do you find the instructions was it you know understand do you understand?

AFP2_0011 : Yeah.

I_01 : And was it easy, difficult?

AFP2_0011 : No, because for me it’s ok because usually are young understand always in gadget everything so it’s no problem.

I_01 : Ok but let’s say if there’s nobody to give you instructions right you know like Miss M didn’t… came here but didn’t give you any instructions just passed you the device you think that you’ll be able to use it on your own?

AFP2_0011 : If there’s a manual with I think I can do it.

I_01 : Ok yah.

AFP2_0011 : If there’s a proper manual yah booklet or to give me what’s the pin number blah and the steps I should be no problem…

I_01 : Should be no problem.

AFP2_0011 : For myself.

I_01 : Ok just a manual ok then you are you aware there’s also there was also a number on the device the, the tablet?

AFP2_0011 : Serial number?

I_01 : There was a number a telephone number there?

AFP2_0011 : Yah, yah, yah…

I_01 : Did you…

AFP2_0011 : After the survey especially help desk or what you are talking about the help desk or…

I_01 : Something like in the one on the tablet like on top of it…

AFP2_0011 : Didn’t know this one.

I_01 : Like the one here… this one.

AFP2_0011 : Oh yah, yah, yah is the is the same as the one after the survey there’s…

I_01 : Correct a number is it, yah.

AFP2_0011 : Correct.

I_01 : Did you man- use this number or call it…

AFP2_0011 : No.

I_01 : At any anytime?

AFP2_0011 : I call once because of it’s near I’m I asking wondering when they are going to collect the thing.

I_01 : Ok, ok, ok so you called this number just to ask to for collection?

AFP2_0011 : But there’s no one but there’s no one pick up the phone.

I_01 : Ok but actually the nurses are tending to it but before it it’s more like medical side one so I so before that do you use it?

AFP2_0011 : No.

I_01 : Not, not really do you think that having a number is helpful or not to have it like you know there on the tablet?

AFP2_0011 : Probably, if there’s someone behind…

I_01 : Ok yah. [Participant and interviewer laughed]

AFP2_0011 : Because I tried to call twice…

I_01 : I know what you mean yah.

AFP2_0011 : But there’s no one, yah…

I_01 : I know what you mean yah.

AFP2_0011 : Yah so…

I_01 : So it must have somebody to be tending the number ok.

AFP2_0011 : Yes correct, yah because you put number if no one no one is attending might, might as well don’t, don’t put it there right? Yah.[Participant laughed]

I_01 : Yah that’s right ok so the number is useful only if there’s somebody behind it.

AFP2_0011 : Yah that’s why I can’t get hold of them I message Miss M again asking when, when are they going to collect…

I_01 : Ok, ok collect it.

AFP2_0011 : Is it still because I got a message at one time it’s saying that the service is ended…

I_01 : Yup, yup the end of it.

AFP2_0011 : Yah so yah so then only so I Miss M are you like coming over I think she never answer because she’s on leave or whatever then she reply back after a few…

I_01 : Ok, ok so let’s say right we did if you have these results that you can keep track of and let’s say you go and see a doc- did you bring it to the doctor or not like…

AFP2_0011 : Sorry?

I_01 : Did you go and see a doctor in, in this time period right and then bring it to…

AFP2_0011 : During the survey?

I_01 : Not the survey the, the, the blood pressure like you know you’re tracking it right did you manage to like in this time period see a doctor and then bring the, the tablet there to…

AFP2_0011 : No…

I_01 : Or the results to, to your doctor…

AFP2_0011 : No, no.

I_01 : Do you think that this kind feature will be useful and helpful or not, tracking it and then you know when you go and see medical doctor in the future right you can bring this like this, this tracking of all these, these results…

AFP2_0011 : Just to show them is it?

I_01 : Kind of like yah to show them to give more information about like you see a bit you know or your…

AFP2_0011 : Normally…

I_01 : This time period?

AFP2_0011 : Ok for my experience whenever I see a doctor be my company doctor or my cardio they will do a blood test they will do a BP by themselves ok, they seldom asked me did you rec- they did asked me did you monitor it? I say yes but for me to I did keyed in a f- maybe half way through but after that [Participant and interviewer laughed] like, like it’s like tedious to key in so I just… yah.

I_01 : Ok, ok so you…

AFP2_0011 : Unless, unless I don’t know how they can incorporate, incorporate inside the tablet but you must bring the tablet also right yah there’s a because sometime you have appointment along I mean if you leave it at home then half way through you from work you go also no point also.

I_01 : Ok so you may not necessary be like…

AFP2_0011 : Because nowadays if you go appointment the what you call that the, the trials is it will do a check then in front I, I think sometime at least 2 checks before you see a doctor and sometime the doctor is like do another check so I don’t think there is the need.

I_01 : Ok but the difference is that this one have the tracking over a long period of time the, the one at the…

AFP2_0011 : But I thought this one is going to push to them the doctor, no?

I_01 : Actually this one is ok this one we are asking you all these things in order we want to see what you think about it you know whether it if you can…

AFP2_0011 : But if it’s like by default it’s sent to the doctor who do the survey I think it’s ok…

I_01 : Ok, ok so…

AFP2_0011 : The graph everything then yah.

I_01 : Yah, yah then they will have the info and all that…

AFP2_0011 : Yah correct.

I_01 : So you think it’s ok in that way automatically.

AFP2_0011 : Yes correct.

I_01 : Ok, ok, ok then how do you feel about this duration of the study like this about 6 weeks right do you think that you want you know…

AFP2_0011 : Longer or shorter?

I_01 : Longer or shorter, yah. [Interviewer laughed]

AFP2_0011 : In the first place why they put 6 weeks? [Participant laughed]

I_01 : It ok I mean the it’s also quite arbitrary in that sense but I mean we want to see your preference also and all that say like we do it on more people you know because we you know might do this on larger scale it would do you think it’s ok or not, this duration?

AFP2_0011 : From my point of view maybe 6 weeks is about what…

I_01 : One and a half months, yah.

AFP2_0011 : One and a half months I think one month should be ok.

I_01 : One month ok.

AFP2_0011 : Unless they doctor have their own specific reason why they choose 6 weeks they must have a reason why they choose 6 weeks why they never introduce 4 weeks or 2 weeks they must have something that the cycle…

I_01 : Yah so actually the idea is that you know to start with a reasonable time frame first but we don’t know exactly when how much people really want so better want to ask you and to find out.

AFP2_0011 : I think in a month it’s ok.

I_01 : Ok one month ok, ok.

AFP2_0011 : Yah not where it’s sensible period.

I_01 : Ok then so let’s say if we introduce this kind of device into you like you know when you first you were diagnosed like I think last year only right?

AFP2_0011 : Correct.

I_01 : So you prefer it to be introduced earlier when you first when you first diagnosed or later a bit you know…

AFP2_0011 : I think it’s best during the initial intervention once the patient know because I got it in March right I mean somewhere there so it’s already what August 6 month? Yah I think by then people during that sickness people have by then already, like me already googled…

I_01 : Yah ok. [Interviewer laughed]

AFP2_0011 : By then, then the info will come to me yah correct if he is if for someone who don’t have the capacity of going google or whatever, with this tablet during, during the ini- initial inter when they diagnosed they can refer back as fast as for they 6 months then…

I_01 : And provide knowledge and, and, and…

AFP2_0011 : Yes correct, so be more prepared.

I_01 : Ok be more prepared.

AFP2_0011 : Or even though they can start with all the video exercise they start, that 6 months can make an impact, yah.

I_01 : Yah, yah ok so earlier as, as early as possible, better.

AFP2_0011 : Yes correct, yah.

I_01 : Ok, ok then a few more questions so would you like have do you have any like you know features that you would like to see you know from that in this in this program that can improve it and make it better. Like something that you feel that some features that you additional features that you like to see you know that you feel it can make it more, more accessible and more user friendly you know…

AFP2_0011 : No, I…

I_01 : Base on your experience you know something that, that you…

AFP2_0011 : No I think…

I_01 : Felt was lacking?

AFP2_0011 : It’s too, too many [Participant and interviewer laughed] too many thing inside also people will get confuse right, yah I think it’s just right.

I_01 : It’s just right, ok.

AFP2_0011 : Yah it’s very clear and what need to be done from the patient side.

I_01 : Ok, ok any other like you know comments on like what we can do to you know make like comments just general comments about like about your own experience how do you felt about it you know any...

AFP2_0011 : Yah like I say is know maybe video how to overcome emotions…

I_01 : Emotions ok, ok.

AFP2_0011 : Maybe help the patient yah how to because I don’t see and from coming from that vi- any of that video it more on what is introducing what is the that the diagnosis the medical term whatever and the health healthy cook diet and even exercise but nothing touched on the emotion side, yah.

I_01 : Ok, ok so more of that, ok.

AFP2_0011 : Yah, yah so that people can understand because when someone changed from behavior or whatever usually suspect is from that sickness but why, why it what triggered it why, then how to overcome it yah I think that is very useful.

I_01 : So actually I just want to ask a little bit if prior to this intro- you know this study where we have this video like you know like the survey questions that triggered the I mean like kind of probed asked you about your emotions. Before that did you realize it or not like do you on your own, yah?

AFP2_0011 : Before I was diagnosed?

I_01 : As in as in like before the study right before like when you had this survey where asking you about your emotion…

AFP2_0011 : The question everything?

I_01 : Yah so on your own do you like you know…

AFP2_0011 : Like googled it or what…

I_01 : Yah I mean like do you real-

AFP2_0011 : All…

I_01 : How do you find out about more about your say managing your coping your rela- your emotions you know?

AFP2_0011 : I think I self managed it.

I_01 : Ok in what I mean like do you get like in what in what sense that you…

AFP2_0011 : Because when I tried to find out this in the web there’s not much things also touching on how to… they never teach like how to some more we how to cope with stress how to cope with changes in your lifestyle so I don’t so I just like try to be the opposite. To be to be positive…

I_01 : More positive more positive…

AFP2_0011 : Yah correct, not think about it as time goes by because along the way for any patient diagnosed with anything along the way treatment phase there sure be a lot of appointments. So sometimes doctor doesn’t tell you if you never ask doctor will just treat it as you know something so let’s said if I why, why the reason what is got the connection of MRI heart scan that I did got to do with AF? What is the link to it?

I_01 : Did you manage to ask your doctor about all these…

AFP2_0011 : Yah, yah because…

I_01 : These, these stuff and all.

AFP2_0011 : Yes because I’m the person who asked but if for someone who never asked [Participant and interviewer laughed] he just go follow through doctor sent here I go sent correct or not, so if there is a lot of more educational on that particular sickness is good.

I_01 : Ok, ok did you manage to get a satisfying answer from your doctor or not when you ask him?

AFP2_0011 : Yes.

I_01 : So you managed to?

AFP2_0011 : Yah.

I_01 : Ok, ok.

AFP2_0011 : Maybe he’s good. [Participant laughed]

I_01 : Ok, ok it’s really it’s really good actually we I mean something that I think we, we…

AFP2_0011 : The knowledge are good knowledge for me.

I_01 : Good knowledge yah, yah ok, ok…

AFP2_0011 : Yah so I just finished my cardio yesterday was at hospital N you see?

I_01 : Ok anyway ok I think that’s about it so thank you very much for sharing with us.

AFP2_0011 : [Participant laughed] Yah no problem.

I_01 : Let me stopped the recording.

End of interview
