# Supplementary material for: Patient perspectives of the Self-management and Educational Technology tool for Atrial Fibrillation (SETAF): A mixed-methods study in Singapore
Source: PLoS One. 2022 Jan 21;17(1):e0262033. doi: 10.1371/journal.pone.0262033 (PMC8782297; doi:10.1371/journal.pone.0262033)
Supplement: S1 Data — (ZIP) [file pone.0262033.s006.zip › AFP2_IDI_0009.doc]

Participant ID number : AFP2_0009

Interviewer Number : I_01

Length of interview : 30.15 mins

I_01 : Ok so the first question is how long have you been diagnosed with AF and how did you find out about it?

AFP2_0009: 2 years ago I think yah correct so it was through a private visit right hospital N when I was admitted I think didn’t, didn’t, didn’t find this. So in the end I have no choice I have to go through private channels yah so they are the one who actually saw that I do have AF right, yah.

I_01 : But you are in the private you didn’t find out through hospital N?

AFP2_0009: No, because when then I have when first have problem I went to hospital N in 2015 or 16 I think it was quickly dismissed even the follow up visit I think was not done up properly that was my position to I think one of the AP before. Yah so it was through the private, private channel that they realized that actually I do have AF yah correct.

I_01 : Ok so in what do you think like when was missing you know what was not was it reading that was not taken properly or…

AFP2_0009: I, I, I would say basic care was not given that is my position yah on those case on that particular case of those few doctors MOs at the over seeing my case back then...

I_01 : You mean at the…

AFP2_0009: That’s why…

I_01 : The, the reading you mean like keep keeping track of your of your, your of your you know the read like…

AFP2_0009 : I, I think…

I_01 : I mean tried to take some readings…

AFP2_0009 : It was A and E it was A and E so I think A and E maybe for them they treat everybody like as it’s not a major case right so that time I remember when I went A and E they will treat 3 patient including me having heart condition issue. But I think the MO on duty I think it’s a lady I have all the information I still keep it yah, I think she just dismissed everybody yah even though I knew that I have irregular heart beat which is AF that day right? But I think they kind of like say something I think medically is not correct base on what I the contacts I’ve spoken to that I don’t think any doctor should easily dismiss irregular heartbeat as something as very normal. Because in the day you already beat thousand of heartbeats so if you had a number of heartbeats that gone missing it’s no big deal, that’s the position of the MO yah which is actually very scary.

I_01 : That was in A and E is it?

AFP2_0009: A and E hospital N yah, yah, yah so in the end I went back again follow up visit nobody even border to do a ECG for me right.

I_01 : You didn’t get the any…

AFP2_0009 : No, not at all…

I_01 : That was not recommended.

AFP2_0009 : My follow up visit yah it was not even done at all nothing, they just dismissed close my case and then after that one year later my, my condition went from bad to worst and that’s when I called my friend in the medical faculty hey I’m in trouble. I go again hospital N they’ll think that you know there’s nothing wrong with me right so I, I don’t trust hospital N anymore so I said can you guys recommend me someone or not so I went to see a very eminent cardiologist who used to be a professor in Hospital N. So I don’t quote the name here since recorded yah but when speak to them privately I quote the name of this eminent cardiologist who is also a professor who he…

I_01 : Who used to be there also.

AFP2_0009: Yah who teach hospital N as well yah so and he was the one that I think did all the proper care yah and I think he did the 24 hour holter test and that’s when he said that hey you have quite a serious AF here right now you know? So why did you come so late, that actually pissed me off yah, correct.

I_01 : Yah, yah then before the A and E actually were you did you see any doctor before that?

AFP2_0009: No, no, no, no, no for me I’m, I’m very clear I, I remember I told the hospital N back then one five I said I’m not that kind of patient who a little bit problem come and cry to you I, I know my health very well. In fact I don’t like to go to hospital unless there’s a need to so I nev- so the moment I had the problem I came in already straight away yah that’s why I went to A and E because I, I came back from travel I heart pain chest pain yah then I came back I breathless. I have no energy I’m supposed to fly again to another oversea country so in the end I couldn’t fly after I, I more observed sure whether am I having a stroke or heart attack or some serious symptoms, right.

I_01 : So when you after the see the private one currently at the moment right do you go back to the private or you go to the that hospital N to follow up for…

AFP2_0009: I, I go both because I don’t fully trust sorry hospital N…

I_01 : Ok fine yah, yah.

AFP2_0009 : Although I had a pack with the doctor in charge he knows I‘m he knows who I’m seeing he know he knows this fellow they all know this fellow this private doctor so I think government hospital [Interviewer laughed] normally don’t like when you are seeing a private doctors. For me no choice because I own to the private doctor than the that, that saved me and helped me alright so I would continue to see the private doctor more to follow up also more as gesture. And I think it doesn’t help me to balance up the view given by hospital N and the view given by the private yah just to make sure it...

I_01 : Yah, yah like kind of a second opinion.

AFP2_0009 : Until because I did tell the doctor in hospital N I think trust has to build between you and me as a patient you cannot just say that I see you I had to give up no, right? So I would say doctor L did a very good job I think he we had some consensus right otherwise I I’m definitely would have make it made this case very big yah I’ll will write officially and go through my contacts to help me yah.

I_01 : Ok, ok so actually you said you see the doctor L right the…

AFP2_0009 : Doctor L yah he’s good he’s good.

I_01 : So that was like aft- how long after the when you got the AF when you see the A and E?

AFP2_0009 : One year later when I become very bad yah or maybe no, no, no in 2016 December.

I_01 : So you saw 2 doctors concurrently the private one and the hospital N one at the at the time?

AFP2_0009 : No I was seeing the private one then later on when become very, very bad I actually admitted myself to…

I_01 : Hospital N, you admit yourself.

AFP2_0009 : Hospital N and that’s when he realized that it’s really, really bad yah so I got admitted for the first time in my life.

I_01 : And you found out only like at that point of time?

AFP2_0009: Yah then I think at that point in time was telling about all these issues yah so I think doctor L was the one that we reached some consensus so I told him at end of the day my stay there 5 day stay there I said I, I appreciate his openness. So I say then we’ll give each other a chance to work together as doctor and patient, yah.

I_01 : Yah, yah so did the doctor give you any advice about like what to do like the…

AFP2_0009 : Yah, yah, yah he did he did I think he did a very good job I think he was very accommodative he understand the, the situation I went through so I think within his capacity I think he authorized as, as many test as he can you know to help to work together. To find out what is a good cause right so I think he has done all his part that’s why I think for doctor L from my point of view I, I, I honestly I really appreciate him for what he has done right, I mean wished that there are more doctors like that in government hospitals yah.

I_01 : Ok yah you want to see more of this kind of doctors who care who care yah.

AFP2_0009: Yah of course I mean that you have good bedside manners [Interviewer laughed] you know, don’t dismiss patients easily like they don’t know anything like that.

I_01 : Yah then other then the, the hospital side you know and the your private doctor…

AFP2_0009 : Sure.

I_01 : Where do you how do you get information to like manage your AF like outside of, of these?

AFP2_0009 : No, actually my AF is all cleared already right so I also with this cardiologist I saw another what you called that AF specialist yah right so this, this lady used to be a president scholar not many every year in Singapore. So I think so I just had some oral medication so I think as far as it is right now it looks like there’s no AF anymore unless I do another 24 hour holter test right so I think that’s the situation now.

I_01 : Stable now.

AFP2_0009 : Stable, stable I, I, I don’t think I have hoped not yah I hoped not yah so it’s a it’s a it’s a it’s a green bill yah that I’m out of AF yah, yah.

I_01 : Ok, ok but in terms of maintaining of your own health you know like what do you do on your own the…

AFP2_0009: I, I, I think on the personal level I, I changed my diet yah a lot I watched my lifestyle so I do more exercise alright than what I used to be before learn to manage maybe stresses at work a lot more better. [Interviewer and participant laughed] So I think that’s all I think I’m done so I think they have been giving very good advice I think both from the private and both from the hospital N right now doctor L and doctor L so quite happy I think for now with the care that is given right, yah.

I_01 : Was there anything that encouraged you to change your behavior I mean like I mean to kind of was there like you watched a program or something you know and sources of information…

AFP2_0009 : No I, I think you did…

I_01 : How, how did you do it like…

AFP2_0009 : No I think it’s more a, a personal a awakening put it this way because when you, you know you all of sudden you realized that you are hit with such a you know chronic illnesses not just one [Participant laughed] but a few that’s a wake up call yah. So you also learnt to ok maybe you learnt need to learn to manage I think that is important so it’s more like a, a personal wake up called that’s how I see it. Yah not because of some video because you know if you don’t take care of yourself very soon some bad things is going to happen like it or not and not yet for me my time is not up yet. [Interviewer and participant laughed]

I_01 : Because we you know…

AFP2_0009 : But I must say one thing I like I see a big difference between the private and the government hospital I think private hospital or private doctors tend to be a bit more at least maybe for those at least for these ones I’m seeing more caring. They will find out about your background your children how old are they, they said ok you know you had to look picked up yourself you know there is a burp there is a bit more personal caring advice.

I_01 : So you think these kind of personal touch…

AFP2_0009 : Of course.

I_01 : Will, will make a difference?

AFP2_0009 : Of course of course unfortunately I think government hospital I think doctors are being slapped by the management [Interviewer laughed] maybe with a lot of KPIs they don’t have time to, to spend too much time talking to patients like us yah because there are too many patients to see. Yah, but I think sometime for us it’s good to know that doctors make the effort by asking in fact I at first initially I, I was wondering why do you need to know about my personal background or things like that right? Because then he said no, no I need to know so that I then he said oh yah your kids are still very young you know and you are only in your late 40s. It’s probably still a long way to go so I think he’s using that basis to motivate me to change my lifestyle and like that like that really work so these to answer your question not a video but I think the care of cardiologists from the private side they made me sit down and think. Yah you know it’s true what he said is very true because if I don’t heed his advice or changed my lifestyle or managed my stresses and learn to slow down a bit then something is bad is going to happen to me very soon. Right so if that’s not I want then I better changed, Chen Loong is here…

AFP2_0009_01 : Chen Loong?

I_01 : So you mentioned ok the public hospital I understand that you have taken they a lot of you know they have a lot of people to see the people so other then the canned base…

AFP2_0009 : Sorry…

I_01 : The, the public hospital the, the doctor right the care is not the same as the private so let’s say if we have a you know…

AFP2_0009 : Is this part of the interview I’m not so sure?

I_01 : It is yah it is yah so if we do the you had a electronic device right…

AFP2_0009 : Sure.

I_01 : That you, you, you used do you think that they will actually help a little bit more improved on that lev- on that on that level?

AFP2_0009 : Yes, in fact I, I, I really think this is a very good idea this tele medicine hold on for a while sorry I think [Interviewer laughed] yah so the ss- they are so is it’s a very good idea. I just think that if hospital N and in this case this research if you can cover more of about not just collecting the data but what the data can mean to me and how I can used that. I mean it’s very good to see all those videos stuff I, I learnt a few things to be honest with you I think it’s very good I think for people who are using for the first time to tell them to be aware of their illnesses how they can take control right? That part I’m not so sure here will be I think there is a case where you can see my data where my diastolic I always have problem with my diastolic when it hit up about 90 and above it’s still considered normal…

I_01 : Oh…

AFP2_0009 : Actually if you look at the American Heart Association standard I’m not so sure about Singapore standard anything that’s 90 and above is consider pre hyper tension 100 and above is I think it’s considered quite serious right, recommendation in AHA is to check in to hospital straight away. So a bit surprise ok what is this going to tell me right now oh your BP…

I_01 : Was there a message that came up?

AFP2_0009 : Yah it said it’s very good it’s still within the range so I a bit puzzled ok so I do not know what is Hospital N standard right because AHA standard which is the American Health Association is a different standard right. So I, I do not know alright so there is something about I do not know whether in the program whether did actually, actually modeled and triggered that by right if you hit 90 maybe a message should be hey you got to watch out you know? It’s on the high side you know it’s something like is it a lack of sleep is it you forget to take your medication…

I_01 : The 90s was how, how consistent was it on like was it one time one all or…

AFP2_0009 : It was there for fi- for that particular day…

I_01 : That one day ok, ok.

AFP2_0009 : Some never know when is it so normally when that happened and doctor L will know he looked at my A and E records so the last A and E that I went early this year I was told that if it ever happened more 90 or 100 I just need to take another half tablet more. So, so I just feel you know in that part maybe can improved a little bit more, yah.

I_01 : Ok, ok you mean the tracking part, yah?

AFP2_0009 : And, and yah you know the tracking has been maybe it’s…

I_01 : The feedback?

AFP2_0009 : This one is the feedback but my, my feedback may not be accurate may be wrong I’m just saying that 90 supposed to be high and above it should not be normal so by right if something like that happened maybe a message be triggered if this is high because 70 plus normal is ok 80 plus it…

I_01 : You are wondering why, why there wasn’t like a kind of a like popped up message…

AFP2_0009 : It should be alert message for me…

I_01 : Alert message?

AFP2_0009 : Rather than said good job motivate [Interviewer and participant laughed] otherwise it cannot be 90 plus is high already, yah.

I_01 : Yah because it could be also be like they, they don’t want to caused anxiety you know like…

AFP2_0009 : It could be it could be…

I_01 : With that kind of alert messages, yah.

AFP2_0009 : It could be it could be it could be…

I_01 : Yah, yah that type of warning messages.

AFP2_0009 : Correct, correct I’ll I’m just thinking whether is it that the program only measure the systolic and not the diastolic.

I_01 : Yah actually is both.

AFP2_0009 : By right it should be both you see.

I_01 : Yah that’s right, yah.

AFP2_0009 : So because some people has problem with systolic and some people has problem diastolic like for me is diastolic my systolic is always ok my diastolic is the one that giving me headache.

I_01 : Ok, ok, ok then what like what was your favor like you mentioned the videos was there any like favorite videos that you had?

AFP2_0009 : Ok I, I think for me I learnt a little bit more about the stroke yah so that is interesting to me I, I must comment that very good graphics there I, I really learnt something out of there. I also like that part including my children you know he said hey you know teaching the exercise. You know that 4 elderly folks that is actually very nice so I thought it was very good balance I, I must really comment that the video part the various videos are, are is really a very splendid job. I can see that from design from conception to you know to coming out the video there’s a lot of good thinking behind that, that is something that I, I in my opinion I think would really benefit patients who are suffering this for the first time or new patients if I may say that. They, they don’t know a lot of things for me I have the I have the I have the luxury to see some good doctors right whether private or the recent government you know like doctor you know L in particular right. So they educated me a lot right so the benefit I know quite a lot about this area already so for me I looked at that even that I still find that very beneficial to me yah so that part I think it’s very good video part, yah.

I_01 : Do you think that it make a difference to, to your behavior or not the video part?

AFP2_0009 : Yes, definitely it reinforced [Interviewer laughed] hey I have to be careful you are this category so it helped me to remind ok please continued watched your diet please continued make sure that watched your sleep hygiene you know enough sleep. Please continued to watch your exercise make sure you do it so these are very good timely reminders there is there is a subtle reinforcement or reminder.

I_01 : Reinforcement?

AFP2_0009 : Definitely, definitely for me yes, yah.

I_01 : Then what about you there were also the popped up messages right?

AFP2_0009 : Yes.

I_01 : Do you think that how , how do you feel when you saw those what was the feelings that you had?

AFP2_0009 : Ok so it’s good to it’s very refreshing to see that there’s a deliberate attempt by the application to give positive feedback in order to motivate you yah to keep our lifestyle those are very good that remind me of the application 365 from Healthcare Promotion Board. I only used it in the last season 3 and I find that it be very beneficial…

I_01 : Ok right.

AFP2_0009 : Because you know there is something that you aim to yah you know you want to where you stand among all the Singaporean yah so for me I want to be 10 thousand average every day alright over a month yah so that that’s my motivation right. So I, I , I I’m not so sure is it necessary yah if there’s a way that you can help patients like that to work towards a healthy lifestyle through application like this, that I think will benefit I think all those patients but I think the overall I think the healthcare ins- you know as a country. I think what we what the Ministry trying to do now is please adopt healthy lifestyle, Singaporean because we also don’t have so much in the capacity to handle so many thing best you take care of yourself so it doesn’t weight down on the whole nation healthcare system.

I_01 : Yah, yah but for your own self the, the popped up messages like when you saw it diet advice encouraging messages…

AFP2_0009 : Yah, yah it does help it does help…

I_01 : It helped a bit I mean…

AFP2_0009 : It does help it just that I think that you have to figure out long term if I used this for because I only used this for about 5, 6 weeks only…

I_01 : Yah that’s right.

AFP2_0009 : So it’s very refreshing for me but if used for one year if I still see the same thing you know it will lose it’s novelty…

I_01 : Ok, ok so you…

AFP2_0009 : So it’s something to work out I, I…

I_01 : So you feel that it will wear like…

AFP2_0009 : It will wear out eventually it will wear out…

I_01 : It will wear off a bit.

AFP2_0009 : Eventually I for me it will wear out.

I_01 : The interest.

AFP2_0009 : The novelty of the whole idea.

I_01 : Ok, ok that’s interesting then what about like the survey part did you do the survey part…

AFP2_0009 : Yes I do.

I_01 : That means the…

AFP2_0009 : I do I do.

I_01 : How do you find them?

AFP2_0009 : The survey is good there’s something I, I, I learnt in fact there are a lot of questions there some questions I, I are good I don’t know ok some questions I, I, I guessed I actually I wished that those that I keyed in wrongly you know they would actually have popped up answer for me. At least I learnt something so…

I_01 : The right answer you mean?

AFP2_0009 : Correct, correct, correct let’s say if I keyed in wrong so I so until now I also what is the right answer I don’t know…

I_01 : Oh ok so you are not very sure with that?

AFP2_0009 : I’m not very sure there, there are one or 2 questions I, I don’t know.

I_01 : There’s no response to, to, to, to that…

AFP2_0009 : No, no, no…

I_01 : The I don’t know part.

AFP2_0009 : I thought that will be one feedback I like to give you know I, I told myself that it’ll be good to because might as well right because you are trying to help us as a patient to understand what’s our condition what’s our illness. And if we do key in wrongly then maybe the application to prompt us the correct answer is this and maybe a little bit of explanation why is this at least we know.

I_01 : So you want ok more explanation also.

AFP2_0009 : Or, or give us the correct answer if we…

I_01 : Or the correct answer ok, ok.

AFP2_0009 : If we if we give the wrong answer yah.

I_01 : Ok so…

AFP2_0009 : I think it will help us, yah.

I_01 : Ok what about like the blood pressure monitor how do you feel when you when you take your blood pressure like and when, when do you when do you take it actually?

AFP2_0009 : I do it normally in the morning…

I_01 : Ok, ok.

AFP2_0009: Correct I think that is important right and I also deliberately did it on those days that maybe I’m feeling a bit off yah because I’m I, I because you all going to collect data right?

I_01 : Yup, yup that’s right that’s right.

AFP2_0009: Might as well I also let you all collect some data where I feel maybe off that’s why you see some I think there were cou- one or 2 days I can’t remember when my diastolic was very high was actually more than 90. That’s why I a bit surprised to see that how come still gave me the same message after all I also trying to test the system will it still give me a good, good, good within range it said within range I said cannot be 90 plus already cannot be within range already I considered high, yah [Inaudible segment].

I_01 : So you are very aware of all these things?

AFP2_0009 : Yah, yah, yah, yah we have to I mean especially if I don’t look after myself nobody is going to look after me.

I_01 : Ok, ok then was here any like I mean like did you do you subsequently when you saw the did you see the a doctor right and then you, you know there’s this tracking of the results right over time…

AFP2_0009 : Yes.

I_01 : Then do you like see a doctor and bring this bring this tablet to show the doctor the, the results you…

AFP2_0009 : No, I did not I did not I did not because during these period I did not see any it was only after the follow up visit the routine visits right then your team came in, yah so I didn’t have the chance to do this right yah.

I_01 : Do you think such a tracking way of tracking your blood pressure over a period of time will be useful when you go and see a doctor?

AFP2_0009 : I will think so right I’ll think so that because most doctor normally for I think especially for new patients right because it’s imperative to establish what is really wrong with the patient right and because at that point in time I think is just trying to narrow down the root caused. So if the doctor can have a full overview plus the patient co-operation like I remember when I see the private doctor he was very clear give me 2 sheets of paper everyday I want you to measure yourself. One in the morning one in the evening and I, I diligently do that know because I know I’m I, I have a problem so if I don’t do this for him he can’t help me so I diligently did that. My initial visit with this private cardiologist so with that he, he, he can helped me to figure what is really wrong then after some time he said don’t need already Mr T, stable already.

I_01 : Ok, ok so you were doing it for the private side?

AFP2_0009 : Yah because here didn’t ask me to do it what? [Participant laughed]

I_01 : Ok let’s, let’s say we, we if it’s a electronic form do you think it’s better than the paper form?

AFP2_0009 : Even better I bet I don’t have to write so, so that’s why I said I like tele medicine this tele what tele health monitor.

I_01 : Health tele health yah.

AFP2_0009 : I like it because I see the value of it right because I don’t have to find a paper to write down and then you plugged in popped then after that you sent the data to the back then, then you know I’m quite sure you can easily interfaced you know for the doctor to view.

I_01 : View, yah.

AFP2_0009 : So it, it works out well for everybody so I, I see a place for tele health and that maybe in my opinion I do not know how you talked about commercialization of the fee right? That one I think hospital N and P can always work out something and tell the patient that this is you know what we are going to charge you to help us. I think some patients will not mind right if, if you tell me that all these information is going to be viewed by the doctor I, I for me I’ll, I’ll pay for it, yah.

I_01 : Oh ok but you mentioned that you, you that let’s say like those…

AFP2_0009 : But I don’t want the tablet I want a smart phone this one let’s say…

I_01 : You want a you want a smart phone...

AFP2_0009 : Yah, yah correct.

I_01 : We’ve got all the forms like a smart phone…

AFP2_0009 : Why, why I want a smart phone because everybody most of us will carry a smart phone today right and some, some people like maybe like myself I’m not a gadget person I don’t like to carry smart phone i-pad in fact I don’t even have i-pad right. So I everything on my smart phone consider if everything can be if application can run on a smart phone why not if it can run on a pad it definitely can run on smart phone right and already I have my own data a already here.

I_01 : Do you think that it will look different because you know we have videos and…

AFP2_0009 : Yes.

I_01 : We have also like you know the like…

AFP2_0009 : Sure.

I_01 : The like the reading also it looked bigger you know?

AFP2_0009 : Sure, sure then maybe that…

I_01 : What do you think about that?

AFP2_0009 : Choice maybe that choice can be made available to the patient do you want a i-pad or do you want a smart phone and these days like it not if you go to MRT take MRT a lot of people are watching video out of their smart phone right?

I_01 : Yah, yah right.

AFP2_0009 : So I think that can be a personal choice I’m not saying that you have to do that but that’s my that’s my preference.

I_01 : Ok your own preference if you…

AFP2_0009 : I’m sure I’m sure if I’m given a choice between an i-pad and this some people will make that choice.

I_01 : Ok, ok so you, you, you don’t mind like even though the screen is smaller and also like…

AFP2_0009 : For me I don’t mind yah for me I don’t mind…

I_01 : Ok for…

AFP2_0009 : For me I don’t mind because I find the…

I_01 : For the tablet yah…

AFP2_0009 : For, for me the usability convenience.

I_01 : You like the convenience that matters a lot, mobility also ok, ok then like when Miss M she came…

AFP2_0009 : How long more because I didn’t my friend just popped by?

I_01 : Just a it’s a very short more, more so like I try to rush hurry up the last part the when Miss M they came did she explain to you a little bit about how this and that and then and it…

AFP2_0009 : Yes, yes, yes she did she did she did run through everything properly with me here in my home.

I_01 : Let’s say if she didn’t come if she came and passed you the device but did not give you instructions do you think that you still be able to do it yourself?

AFP2_0009 : I will be able to but I need instructions guide to be there, there is no instructions guide that’s why she had to explain everything if she if she doesn’t then at least minimum there should be an instruction guide. I think that can, can be something that we can leave it to the to the patient to decide right you can easily do things like ok if you do this on your own with the instruction guide you know then easy if you want someone to come down maybe you pay a bit more, yah.

I_01 : Ok then what about like the number do you manage to call the number or not like the, the…

AFP2_0009 : Didn’t, I don’t need to, yah. [Participant laughed]

I_01 : Do you think that it’s helpful to have a number there?

AFP2_0009 : It’s good of course in case there is a problem but I don’t have any issues you see, yah it’s basically the number to call when something happened, yah.

I_01 : Ok then what about like ok so a few more questions…

AFP2_0009 : Sure.

I_01 : If let’s say we introduce it this AF right to you do you prefer that it’s introduced at the beginning when you had diagnosed with AF or maybe a bit later…

AFP2_0009 : It should be in my opinion if this is used to monitor AF it should be early…

I_01 : Ok early.

AFP2_0009 : Yah why? Because doctor and patient could established quickly you know when I can put this under you know controlled that is important then I do not know I’m, I’m, I’m, I’m not a doctor trained right so it’s for the, the you know all these people to decide. Eventually maybe they have to figure out how long do we need to run this right maybe who knows it could be for the start initially to address, address, address the data record the data and after everything is stable maybe it could be who knows 6 months later. Do another one month just captured data make sure it’s really stable yah, or it could be something say or maybe every 6 months or every one year that could be something that can planned. Or if patient really want then it can be every day but then there must be some other value added stuff for patient to want to do this on a daily basis over a long term yah period.

I_01 : How do you find these 6 weeks is it it’s just right or too long too short how…

AFP2_0009 : No I think 6 weeks is ok yah but I…

I_01 : Let’s said we pilot into more sing like people more patients you think what, what when what do you feel is the right amount of time that they will be comfortable with?

AFP2_0009 : No, actually you should put in more apps inside…

I_01 : More apps oh, oh…

AFP2_0009: More features inside you know if…

I_01 : Oh ok more features!

AFP2_0009 : One, like for example if you want to target this maybe not just target AF maybe you know it’s really meant for people who have AF who have BP you know issue or maybe who have some other heart related condition. I, I don’t know I can be wrong here I’m just trying to think here and if can do that then you know like Apple have a new, not Apple it’s a application call cardiac or car- cardac health right there’s one that runs only on i-phone unfortunately don’t run on android. I’m using android phone I, I thought that was very good because it captured all the BP, the heartbeat and it do a lot of projection on graph it will tell the patient where is it right so that will also maybe give the patient the information to know how to care for themselves.

I_01 : Projection what do you mean by projection like…

AFP2_0009 : It means that they will like do what you are doing the last 7 days the last 28 days…

I_01 : Ok, ok but not the future you, you…

AFP2_0009 : No, no sorry that’s not what I mean…

I_01 : That’s what I thought what you mean…

AFP2_0009 : Projection means to have the data over graph this what I meant we can’t project that one…

I_01 : Yah, yah that’s why. [Interviewer and participant laughed]

AFP2_0009 : We can’t no way.

I_01 : We got it different yah, yah.

AFP2_0009 : [Participant laughed] Nobody can do that.

I_01 : Yah, yah we leave it yah, yah.

AFP2_0009 : So if we can do more then there is a reason for let’s say a patient like me to want to carry this I mean it’s a permanent feature like if today if S have I used it everyday why? Because it has so many things inside I can used it to measure my, my stress, my O2, measure my steps I’ve taken measure my BP right I measure how many steps I come a day so I used that you see…

I_01 : So that the steps…

AFP2_0009 : I used these everyday.

I_01 : It’s, it’s useful for you the steps that you...

AFP2_0009 : Yah, yah, yah because I have a S phone you see that’s how they make money right because today I’m using S already S health might as well I go and buy a…

I_01 : The wrist the watch.

AFP2_0009 : A, a S phone…

I_01 : And it’s connected already.

AFP2_0009 : Yah connected, yah so if you can do along that way then yah because at the end of the day you, you all have to create the whole system why a patient want to use this over a long term basis. The more the appli- apps you put in which is related to maybe the heart condition of a person the more I want to use this, not only when I have problem but I want to use it all the way. So data by right medical data should be collected continually not you know when I need it yah other wise it doesn’t help the patient it doesn’t help the doctor, yah...

I_01 : So any idea on like…

AFP2_0009 : That’s my view.

I_01 : Ok that definitely very useful because you, you know you something that you, you were using other things also right so you want to also find out what other things are, are useful for you. Like say you there was one the question about the you know let’s say we, we pilot this to more locations right do, do you think that 6 weeks is too short too long just right should we do it longer what do you think?

AFP2_0009 : No, I think 6 to 8 weeks is just about fine.

I_01 : That’s ok.

AFP2_0009 : Yah because we also don’t want to be too long and people not using it then no point so I think 6 to 8 weeks give you a very good guide because it’s about already a month to about 2 months. I think that should be good enough if you want to do a pilot anything more that people may not use it then your equipments are just there and you know no point yah because you just want to collect data right the user behavior yah.

I_01 : Ok so any other like comments about your experience and you have any other things to add on any things you want to say about your own you know about the whole experience of this tablet?

AFP2_0009 : No if I can if I can conclude because I said checked most of my feedback it’ll be good and what I think can be improved but overall I, I really think that this is a very good initiative that’s the reason why I volunteered.

I_01 : Oh ok, [Interviewer laughed] really?

AFP2_0009 : I said oh yah maybe I should try and you know and see how it helped is and how eventually this can help the nation you know people who have this problem like me right and, and I, I think it’s very good initiative. I really hope this idea won’t, won’t stops here I hope that you know hospital N and P can figure out how they can really work this things out. I really think by having more apps is really going to help and I really in my opinion I think giving the patient a choice of between the tablet and a, a smart phone will really helped us. Right so if you can work on those area yah maybe it will be more interesting data to come out I hope so yah and especially we are aging population and I think many of us are very well versed with smart phone and tablet yah, so I think you’re on the right track…

I_01 : Alright.

AFP2_0009 : This tele I didn’t know about tele, tele health…

I_01 : Health program?

AFP2_0009 : Yah but I know about tele medicine yah I don’t know about tele health. [Interviewer and participant laughed]

I_01 : Ok anyway thank you very much let me stopped the recording.

AFP2_0009 : Sure, sure, sure.

End of interview
