# Supplementary material for: Patient perspectives of the Self-management and Educational Technology tool for Atrial Fibrillation (SETAF): A mixed-methods study in Singapore
Source: PLoS One. 2022 Jan 21;17(1):e0262033. doi: 10.1371/journal.pone.0262033 (PMC8782297; doi:10.1371/journal.pone.0262033)
Supplement: S1 Data — (ZIP) [file pone.0262033.s006.zip › AFP2_IDI_0008.doc]

Participant ID number : AFP2_0008

Interviewer Number : I_01

Length of interview: 29.31 mins

I_01 : Ok so thank you very much for taking part in this study the first question we want to ask is for how have you been diagnosed with atrial fibrillation and how did you find out about it, from the beginning?

AFP2_0008: I think from beginnings I think is about 5, 6 years.

I_01 : Ok 5, 6 years.

AFP2_0008: Or, or more than that ok it’s started is when I don’t feel good and I decided to go to A and E to Hospital S heart ok then later was at the heart centre so do all sort of test and there was one day I really don’t feel good. I, I again so I went there A and E then stay for 2 days I think and then they say I had AF so they wanted to do a procedure but at that time I’m not ready and I refused and after that I, I wasn’t regular on my medication to be honest ok. So there was one day while I was attending a course I had very survey pain on my head very, very painful I couldn’t take it anymore I have to put down my thing packed and go left and I decided to get Hospital N which it is nearer. So when I was in hospital N and warded had more or less confirmed a diagnosed of course I get scolding for not being taking my medication regularly so from there I see doctor every time and then later I get to see doctor L. And after many type of medication many type, type it, it doesn’t really helps so he was suggesting that go for ablation so not too ready but in decide got to go means got to go. So decided to go during my first ablation wasn’t as successful in the sense that something happened in the midst of it even sort of thing I bleed internally so I was in ICU so thank god that I was I recovered fast. Ok and I after that continued my medication in fact he wanted want to go through again I said no, no way I can go through I won’t want to go through so upper after a year or 2 I think a year plus to I this plucked up the courage decided…

I_01 : To go for it.

AFP2_0008: Go there go for it and it was very success until now I don’t think I have any attack maybe one or 2 times ok but I don’t feel like last time like that I would say that this was quite success was success.

I_01 : Ok so the, the, the successful one the surgery was like last 2 years ago about that?

AFP2_0008: I think so must go and check my record. [Interviewer and participant laughed]

I_01 : Ok, ok because you mentioned that the first one was about 2 years after you’re the when you first diagnosed 5, 6 years, years ago right?

AFP2_0008: Yah, yah actually I was diagnosed AF is more than that but…

I_01 : Longer than that, ok.

AFP2_0008: It’s long it’s slightly more than 6 years…

I_01 : Ok longer than that.

AFP2_0008: Plus, plus hospital S and hospital N and then this kind of thing you see?

I_01 : Hospital S and N.

AFP2_0008 : This one my mom.

I_01 : Ok, ok then how do you manage in this time period generally your AF right I mean what kind of information did you go to you know to, to find out more about it you know out outside of hospital?

AFP2_0008: I mean I, I read books I know that the worst scenario case is I’ll get strokes but I don’t want to get strokes seeing my mother what my mom had gone through and other people I don’t want. So because of that even I even go through the sur-, sur- procedure and everything after the last admission at, at I mean the admission at hospital N my that’s the limit I start taking my pills regularly to avoid [Participant laughed] don’t play, play.

I_01 : Any, any issues later on, so your mom had, had stroke before the is it that’s how you, you know got familiar with you know the symptoms and all these?

AFP2_0008 : My mom got stroke but my mom doesn’t I don’t whether is related to AF or not because of old age and all the thing.

I_01 : Yah ok for other reasons could be other reasons.

AFP2_0008 : May maybe I don’t know.

I_01 : Maybe ok, so on like what about your family friends and you know other people whom you go to for advice did anyone, anyone else do you look for to…

AFP2_0008 : Advice they advice me or advice…

I_01 : Kind of like they you all talked to one another you know could be could be either way?

AFP2_0008 : They know I have problem but we didn’t talk about this issue ok we, we didn’t talk we talk is just like another…

I_01 : Ok, ok…

AFP2_0008 : Med medical team we, we don’t really…

I_01 : Ok l mean like share on this…

AFP2_0008 : No, no, no, no they...

I_01 : This particular issue?

AFP2_0008 : Didn’t get only to after my procedure what I have gone through and everybody is more or less like oh is there such thing oh you know what I missed out the awareness comes in the whole family and other people ok and my brother got it also my brother had AF it was after me. Everyday I go and see nurse and he is seeing Doctor L also.

I_01 : It’s so you are seeing Doctor L…

AFP2_0008 : Yah so happen I’m seeing Doctor L.

I_01 : Ok, ok, ok then yah what your family they’re closer to you so would they have you know helped you in any way and to, to you know to like say if you go exercise you know they, they…

AFP2_0008 : They accompany me?

I_01 : Yah accompany you or encourage you all these thing?

AFP2_0008 : No we, we just of course say you better go and exercise but whether only me and my husband that’s all.

I_01 : You and your husband so you all do…

AFP2_0008 : Encouraged one another.

I_01 : You all you all do go.

AFP2_0008 : But…

I_01 : Now and then.

AFP2_0008 : Yah now and then and the groups of my friends but not family members.

I_01 : Ok, ok then so like the these, these 6 weeks of the study how do you normally use the device on a average day?

AFP2_0008 : I usually because I was told to use once a day actually I want I’m tempted to do another time but I did not because I was told only do…

I_01 : Ok, ok oh why?

AFP2_0008 : One week one, one day one time enough ok because there are times that I don’t feel well because honestly during these 6 week there was once my blood pressure shoot up very high 200 over ok it’s very high…

I_01 : Yah it, it is.

AFP2_0008 : Because right now right now my you what my record is normal but because that day I don’t feel well ok and I went to doctors a GP and then take my medication to herself I think I was having cough or flu or anything and then when she checked my blood pressure wow, was very high. So actually I wanted come back I want to record it you know to show but then I was thinking well, said one day once what so ok so...

I_01 : Oh so who told you actually the one the only one…

AFP2_0008 : The lady who delivered.

I_01 : Oh Miss M is it, M.

AFP2_0008 : I don’t know who she said do…

I_01 : Her name is M, so oh ok anyway when she told you one time she’s did she say that you can do actually more than one time?

AFP2_0008 : I don’t think so I as far my, my understanding.

I_01 : Yah so there, there wasn’t any mentioned of it.

AFP2_0008 : She just mentioned that you’ve got any question you can call, call the help desk but I, I, I thought help desk means machine problem so I didn’t.

I_01 : Ok so actually ok we so we I want to ask a bit about the Miss M the instructions do you find it helpful for you or not?

AFP2_0008 : Ok very simple anybody can understand.

I_01 : Ok was it too long or…

AFP2_0008 : No, no, no, no it’s a demonstration and…

I_01 : Ok then, then the number you mentioned did you call or not actually?

AFP2_0008 : I didn’t call.

I_01 : Why, why didn’t you call you…

AFP2_0008 : Because I think not necessary I mean I see every time they message normal, good, keep up so ok no need to call. [Interviewer laughed]

I_01 : Do you find that the number is useful to be have, have it there?

AFP2_0008 : It’s good to be there it’s just that I didn’t used it.

I_01 : Ok, ok so but if let’s say you will have this for a longer term you…

AFP2_0008 : I might.

I_01 : You will prefer to have this number.

AFP2_0008 : Yah yes, yes.

I_01 : Ok, ok rather than no number.

AFP2_0008 : Yah, yah because sometime you, you like ok example take, take a scenario maybe suddenly you don’t feel well and then your pressure is up or whatever it is and it’s not serious enough to go A and E ok, but you need some advice no need actually no need to go doctor maybe through phone can advice.

I_01 : Ok so your you, you won’t mind a phone kind of…

AFP2_0008 : Yah I don’t mind because it it’s very important I felt that.

I_01 : Would you with ok let’s say if not as severe for the A and E right would you preferred to have a phone or you preferred to at least go and see somebody person?

AFP2_0008 : Can also ei- either way.

I_01 : Either way I mean…

AFP2_0008 : Either way you see.

I_01 : Ok, ok would you find that like any preferences?

AFP2_0008 : Because like honestly there’s once I was having an I mean serious of unfeel well that was before my ablation and this sort of thing I just want to ask for early appointment they don’t allow me they said go A and E. But because I’m a subsidy patient ok recorded it’s ok so I had a argue you mean your hospital part I go A and E is not serious enough I they you all medical or hospital is trying to encourage us not anyhow to use the A and E. Ok for me I know myself I know it’s not serious enough to be go A and E ok but just need some advice that’s all.

I_01 : So…

AFP2_0008 : Or, or early appointment to see doctors.

I_01 : And then they couldn’t they couldn’t…

AFP2_0008 : They could no, no, no you’re not…

I_01 : Not, not the appointment was not, not…

AFP2_0008 : Not, not there yet not a private patient private patient you can get straight this is Singapore so I was very mad I just throw down the phone [Interviewer and laughed] but I didn’t then realized so mad after 2 days they called me up and then asked me why I’m not there. So I’m honest I said of course I won’t but I was very mad there and there but that is out of this.

I_01 : Ok so more flexibility is better.

AFP2_0008 : Yes, yes.

I_01 : You and you would like that ok then also the you mentioned about the popped up messages how do you find it, was that the encouraging messages the diet advice all these things?

AFP2_0008 : Some are good some are very irritable…

I_01 : Oh ok.

AFP2_0008 : Because irritable in the sense that I did not off it, if let’s say I do, do already I just off it I didn’t off the whole tablet either in the middle [Interviewer and participant laughed] that’s one reason got fed up wake up I off. At first I thought what is this middle of night who messaged I, I didn’t realized it’s this I thought my husband phone.

I_01 : Oh so it’s was kind of like distracting in a very irritating…

AFP2_0008 : In a way because other than that ok, of course that time then later I learnt to silent mode it or just…

I_01 : Will there…

AFP2_0008 : But some messages are important, better read.

I_01 : Will there any feelings that you have when you read the messages like you know I mean I understand the, the vibrations that part was a bit irritating but then what about like if you when you read the messages how do you feel?

AFP2_0008 : Ok the, the messages are ok.

I_01 : Ok meaning like what how do you feel how do you feel?

AFP2_0008 : How I feel it, it I mean like I said ok…

I_01 : Like happy, happy you know you feel happy…

AFP2_0008 : Yah, yah happy ok!

I_01 : Sad? [Interviewer laughed]

AFP2_0008 : It’s ok can, can not, not say that kind of irritable or nothing but it’s encouraging words positive words…

I_01 : Positive lah.

AFP2_0008 : Even sometimes is I know is borderline case or a little bit high they will just encourage you the encouragements…

I_01 : Would…

AFP2_0008 : Tell you not to give up.

I_01 : Ok does that help you, you think?

AFP2_0008 : In a way, yes.

I_01 : Ok, ok, ok then what about like the videos did you watch the videos or not like the videos that when the…

AFP2_0008 : I, I watched actually not really watched but some after a few times a few day later then I decided to watch it’s very educational.

I_01 : Oh ok which was favorite, any favorites?

AFP2_0008 : No, no favorite but all are goods ok very encouraging and very knowledgeable I mean it, it tells you how the things really what happen to your body if you’re high blood pressure or if you’re AF what happen and, and this kind of thing you see.

I_01 : How about the duration how do you find the dur-…

AFP2_0008 : Ok just nice.

I_01 : It’s just nice, ok.

AFP2_0008 : Just nice, not too long not too, too long sometime you your mind can be…

I_01 : Yah then you know the exercise and the cooking one you know you know sort all these habit advice do you think that it will actually change your the way you, you know your behavior or not, do you think that really, really helps you to…

AFP2_0008 : Maybe I don’t know. [Participant laughed]

I_01 : You don’t know ok, ok [Interviewer and participant laughed] then what about like…

AFP2_0008 : Because I like, like 60 years old already ok, this sort of thing but at times I’m worried I’m worried I get stroke at times the only signal I worried I get stroke only you see.

I_01 : Ok so you…

AFP2_0008 : Who is going to look after me [Interviewer laughed] I mean of course I’ve 3 kids but I don’t be a burden to them.

I_01 : Yah, yah ok.

AFP2_0008 : Ok that that’s the problem I don’t want to get stroke so I take my medicine…

I_01 : Do you think that the videos address this worry of yours or not I mean like you know you mentioned about you know who is going to take care of you in future…

AFP2_0008 : I not really before that already… [Interviewer laughed]

I_01 : True…

AFP2_0008: I, I, I just sometime I do think one…

I_01 : So you have this in your mind throughout.

AFP2_0008 : Yes, yes not because of the video.

I_01 : Ok I mean like if let’s say if we have some videos can also like helped you to maybe assured reassured you and give you some comfort you know in terms of this, this worry that you have?

AFP2_0008 : Because I think this video not so much I mean we as patient we see but it’s good that caregiver see also you, you know because right now I not that kind like that I need a caregiver you know what I said but I do show my husband let them see you see. He might able who knows if he don’t take care himself he will get it also…

I_01 : Ok so…

AFP2_0008 : I can put there and show my family members they the boys and everything they have to see because they don’t take things for granted.

I_01 : Yah, yah so you don’t mind sharing it with your family and all that you think it’s good…

AFP2_0008 : Yes, yes.

I_01 : Ok, ok, ok then what about like you know the blood pressure monitor you said you take like quite often right, right…

AFP2_0008 : In a way in a way I think I missed a few times.

I_01 : Ok but generally speaking ok so how do you feel when you take the blood pressure monitor do you have any feelings or not like how do you like you feel stressed, anxiety or is it happy you know…

AFP2_0008 : I, I don’t feel stressed because before this thing start I do have a, a blood pressure that thing I take my own blood pressure once I meant regularly to check myself you see?

I_01 : You think there is a difference or not between the you know your own device…

AFP2_0008 : But of course this one like, like sure accurate psychologically [Interviewer and participant laughed] the same thing I mean I’ve a good one but psychology you felt that this one is good like, like, like because it can pass through the hospital passed through the you feel more…

I_01 : Assured?

AFP2_0008 : Assured!

I_01 : Ok so it make a difference.

AFP2_0008 : It’s regulate.

I_01 : I mean like compared to like one that doesn’t have the connections to the hospital so you, you think that it makes difference ok, ok that’s interesting.

AFP2_0008 : It but will make you all more [Interviewer and participant laughed] busy.

I_01 : Not no, no actually not necessary they, they are already you know thinking of making these on a larger scale so I think your feedback you’re actually you’re pioneer you’re like the first person to pilot this you know so, so I’m going to do that’s all.

AFP2_0008 : But I found that is good I mean I don’t because it give you assurance I have 2 blood pressure because actually I’m not only the one who feel that sometime you… accurate or not so my friend we, we do share I have a girlfriend we, we share our, our thoughts on these you see. Because her husband was like this girl buy 2, 2, 3 sets [Interviewer laughed] don’t know how many think young I said sometime I don’t feel don’t know whether the…

I_01 : Accurate?

AFP2_0008 : The reading is accurate this one also take and ok average out ok.

I_01 : So the result for this one you find is it about the same as or different from the one that you have?

AFP2_0008 : It’s about the same and but, but what I mean is that as I said the main thing is because it’s connect to hos- you feel like…

I_01 : Different?

AFP2_0008 : It’s difference you feel that it’s more accurate.

I_01 : Ok, ok, ok that’s very interesting actually [Interviewer laughed] then…

AFP2_0008 : Psychology all these are psychologies.

I_01 : Psychology it’s very important also it will make a difference ok then what about like the, the, the screen how do you find it was it just…

AFP2_0008 : This screen ok, ok.

I_01 : It’s ok, ok then the, the size of the words and everything, font?

AFP2_0008 : Ok, ok, ok.

I_01 : It’s ok the then what about the brightness and all that?

AFP2_0008 : Ok, for me no problem.

I_01 : Is it is ok then what about let’s say we have a we give you a choice between using a phone, a tablet, a desk top computer or any other electronic device could be a TV if any what will you prefer to use you know what will be your preferred form of device?

AFP2_0008 : If I don’t have a PC used this ok this ok if I have to used this also ok like solely on this no other nonsense thing…

I_01 : Ok you don’t mind?

AFP2_0008 : I don’t mind.

I_01 : Ok any preferred one?

AFP2_0008 : No, no, no, no.

I_01 : Ok so any, anyone will do?

AFP2_0008 : Any…

I_01 : Ok even if it’s like a say like a computer form you know where you got to log in all that…

AFP2_0008 : It’s ok it’s ok excuse me for a while we can stopped because of the rain.

I_01 : Ok then ok we were talking a little bit about like the interface of the tablet you know the different form ok then ok so let’s say we have the paper form you know paper where you write down and then you sent mailed to us back right and then we sent you brochures for information. Would you prefer a paper form or electronic form your own preference?

AFP2_0008 : Of course this is easier right 100 percent easier. [Interviewer and participant laughed]

I_01 : Yah I know I know that you find might find the question a little bit weird because we you know we want to…

AFP2_0008 : I know paper form paper is ok but it need discipline this one also need discipline but I think paper form will be people need more even more discipline so this one just take, take, this one you write you know so sometime you put on the table you forgot to walk out and post it.

I_01 : Yah correct so, so you so you preferred like…

AFP2_0008 : I, I don’t mind.

I_01 : Ok is, is it you feel, feel that it’s more convenient ok, ok def- ok then what about like the was there any favorite feature that you like or not anything that is your favorite like something that you feel like is the best feature?

AFP2_0008 : This one?

I_01 : Yah of…

AFP2_0008 : I think the graph.

I_01 : The graph ok so what about it?

AFP2_0008 : At the end of day the graph you see whether you improved or same and then when it goes up you will tell yourself what you, you tell yourself you need to do something about it that’s it.

I_01 : Ok, ok so let’s say if it goes a bit high would you get any feelings or not I mean I, I, I understand that you know you wanted to take a few times be you know because you there was a few times it was a bit high right? So like say if it’s I mean like if it’s too high for like maybe one point of time would you feel like will you feel like it will affect you or not you know in anyway?

AFP2_0008 : Emotion?

I_01 : Yah emotion, psychologically?

AFP2_0008 : Maybe if maybe it, it will I don’t know maybe it will and maybe if for me to tell myself I need to cool down take a rest or, or, or do some other exercise, why my I will asked myself why my pressure went up?

I_01 : Would you like to…

AFP2_0008 : Suddenly?

I_01 : To see like you know let’s say it’s a bit higher then they give advice about what to do and you know you would, would you would you like to see something like that?

AFP2_0008 : Yah why not cause I, I would before when I see if it’s high why I’m like that in the first the question I will asked what I suppose what causes me the main thing is what causes me you see?

I_01 : Ok, ok then what about like the let’s say right you, you said the there is this graph you can keep track of it over a long period of time so when you go and did you see a doctor subsequently to then you bring the graph to, to the doctor?

AFP2_0008 : So far this one I never bring.

I_01 : Not yet, would you think that it will be helpful feature or not?

AFP2_0008 : Yah especially when go seen doctor L, I mean if I go have you been taken where is your record?

I_01 : And also like other doctors also if let’s if…

AFP2_0008 : Yah not only him…

I_01 : If let’s say you go.

AFP2_0008 : Even if I have go GP or wherever…

I_01 : Yah GP.

AFP2_0008: I think it’s good, you see?

I_01 : Ok, ok so you think it’s good ok, ok then what about like do you know that you aware that there’s a number you said you didn’t called right ok, ok so I think we asked this already. The duration of the study 6 weeks would do you think that it’s just right how do you find it too long too short just right you know?

AFP2_0008 : I think a little bit short.

I_01 : A little bit short ok, ok.

AFP2_0008 : I think, I think because…

I_01 : Why, why do you say why do you say that?

AFP2_0008 : Because that’s the time where I start getting used to it the time where I get to like go on like exicited like, like I want stopped already.

I_01 : Oh ok so you feel ok so how long will you think will be the appropriate time because you know imagine we pilot this for like you know much I lot more people you know thousands of people so how do…

AFP2_0008 : 6 weeks…

I_01 : You think will be like?

AFP2_0008 : Maybe up, 6 weeks is how many months 6 weeks is…

I_01 : One and a half.

AFP2_0008 : One and a half, maybe 2 months.

I_01 : 2 months ok so a bit longer.

AFP2_0008 : A little bit longer.

I_01 : Ok but 2 weeks is not, not very long right you think?

AFP2_0008 : 2 weeks.

I_01 : I mean I I’m just, just wondering will you find it…

AFP2_0008 : 2 weeks is ok also.

I_01 : It’s ok, ok no I…

AFP2_0008 : 6, I mean as 6 weeks…

I_01 : Plus 2 weeks.

AFP2_0008 : Plus 2 weeks it’s ok.

I_01 : Ok so it’s like you preferred like 2 months.

AFP2_0008 : 2 months!

I_01 : Ok 2 months, ok then what about like you let’s say we introduce this device in a when you first diagnosed you preferred at the beginning when you first diagnosed or like later on a bit?

AFP2_0008 : I think beginning…

I_01 : The best time?

AFP2_0008 : The beginning.

I_01 : Ok why, why beginning?

AFP2_0008 : So that the person can aware what is happening and early intervention.

I_01 : Early intervention ok but let’s, let’s say like because you, you mentioned that you know you get bad anxiety if it’s no I mean not, not you I mean like it’s possible to have that right? So at the like the beginning because when you are new to it you think that, that will be an issue or not like for those people who first diagnosed, I’m just wondering you know you think that there will…

AFP2_0008 : It depends on that person…

I_01 : What you think?

AFP2_0008 : It depends on some people might worried so serious must do monitoring so it depends how the person take it as a whole thing.

I_01 : Ok, ok depends on individual ok so what about like ok just a few more questions will you have any like things that you want to see in this base on your experience right anything that you want to see in this app and the tablet you know something you can add on…

AFP2_0008 : This is a very good tablet and this, this, this study I mean who whoever it is called is good but I think you can add in more…

I_01 : Like what?

AFP2_0008 : I mean if you go to like recording your I think it’s difficult but don’t know how you going to do it like diabetes draw blood record that will be good.

I_01 : This ok, so like you like you know measurements of like the blood…

AFP2_0008 : Blood and how to record it in you see so that the hospital will know high blood like can be all coming to the same thing you see?

I_01 : So more conditions ok.

AFP2_0008 : I don’t know just said if it’s is good to open up to these like testing blood I mean of course the strip we can test our own recording in I know it take a lot discipline.

I_01 : Yah ok then what about like ot- like the feat- the, the, the, the electronic features of the app?

AFP2_0008 : It it’s, it’s ok, good.

I_01 : Ok anything you want to see you think you want to improve…

AFP2_0008 : I think it’s very good already.

I_01 : Ok you think is very good already.

AFP2_0008 : So far now.

I_01 : [Interviewer laughed] Ok, ok then let’s say right we do this on a larger scale do you how much do you think people will pay for it, like I mean…

AFP2_0008 : It, it depends on, on, on, on financial ground ok?

I_01 : Ok depends also.

AFP2_0008 : It yah it all depends on finance some even you asked them to do it they also won’t do it what like the amah [Means “elderly lady” in English] the maid rely on the maid sometime the maid might have forget or whatever and some family might can’t afford, I mean not said what some even really…

I_01 : Yah, yah that’s true ok, ok, ok so actually yah that’s I think that’s about it for the interview [Interviewer laughed] if you got any other comments on that, that you know you want to…

AFP2_0008 : No I think very well done you all, I just hope that you opened up to more…

I_01 : More people?

AFP2_0008 : More people.

I_01 : Ok any feedback you want to give to maybe doctor L? [Interviewer laughed]

AFP2_0008 : No, no, no he, he did… [Participant laughed]

I_01 : Because it’s all recorded we will he will hear it also will be like he will…

AFP2_0008 : Yah but anyway and then…

I_01 : He can get accessed to it.

AFP2_0008 : The, the team is good the team is doing well and, and this sort of things you see, but because it just that need I, I hope that it opened up to more people.

I_01 : Yah that’s the plan hopefully.

AFP2_0008 : Hopefully and, and these people who let’s says you all lend, lend on just like right now you do you, you all need to maybe monitor just for a few weeks or feel what their condition at home ok so make sure they really do it.

I_01 : Do you think there’s any down sides or not I just wondering you know of the back of your mind like if you think there’s any down sides to using something like that you know I know that is very good you know your experience have been you are quite satisfied. I mean it seems, but like say you think that, that could be a possible disadvantage?

AFP2_0008 : Disadvantage, people who used it if they are so anxie- excited anxiety type that kind of character gan cheong [Means “excited” in English] type little bit little bit call little bit little bit call you know you know that will be quite irritable to the per- the person who receive the call. Sometimes is very minor they, they, they called…

I_01 : But because you know…

AFP2_0008 : They must know and understand the sickness of the thi- this what is AF they must understand you see they must understand when you get panic no need to get panic now I I’m so used to it so like that. So like today that day I just received one call somebody says oh how I got palpitation and sort of thing I said calmed down just calmed down if you feel don’t really good just go A and E…

I_01 : Oh…

AFP2_0008 : That’s all then want go private get I said private going to cost you a lot just go to polyclinic ask polyclinic do a referral letter.

I_01 : Yah but ok let’s say if you if you s- if you used this device you know then you get a result that you know you get a bit maybe you feel that uncomfortable a bit would you go to AI A and E more or not or you would you call?

AFP2_0008 : It, it depend on the person if the person first get first to the disease or to this sickness they will straight away go straight away go like me beginning I a little bit feel very uncomfortable I go. But at the end of the day nowadays I checked if my pulse rate is not 100 over or very, very low I won’t enter A and E and also goes with my blood pressure ok. The other time of course I headache I don’t know my pressure and my now of thing but of course after discharged at times I got 134 or 145 or I missed a heart rate ok if it’s like that I would just take my handbag and just go that’s, that’s it. So either you stay one night or after a few hour they ask you to go home that’s it but after long term you know you don’t 80 over go already [Interviewer laughed] you don’t already because you are so used to it already you understand the sickness already.

I_01 : Yes, so understanding very important.

AFP2_0008 : Very important and take your medication very important especially I just like that day got A- the radio on about AF medication is very important like your but for me I think blood thinner is very, very important. [Interviewer laughed] I don’t want to get clogged and then [Interviewer laughed] go into my brain and, and I, I got this stroke you see.

I_01 : Yah you don’t you want to avoid that as much as possible ok, ok.

AFP2_0008 : You see.

I_01 : And, and you know that you can you can do something about it, ok.

AFP2_0008 : Yes, so it’s education we need to educate the group of people who has AF you see?

I_01 : So education will come in handy together with using this?

AFP2_0008 : Yes or, or maybe you all can set a, a what is what is that called a group that have AF supported I mean support you see some of them might be nervous don’t know what to do just encouragement all these need encouragement the support, support group I say…

I_01 : So the support group will…

AFP2_0008 : AF support group.

I_01 : So you, you, you won’t mind going if we organize something like that?

AFP2_0008 : Yes, I won’t mind just listened and then like amah [Means “elderly lady” in English] some amah who already real how that there was once I was waiting that the amahs will tell me how wo a sim [Means “my heart” in English] then you then you explained don’t worry what, what, what, what…

I_01 : Ok interesting.

AFP2_0008 : Just talk to them education, their family members also must understand don’t throw to the maid some of them [Interviewer laughed] just throw to the maid know?

I_01 : Really!

AFP2_0008 : It is true be fair.

I_01 : Ok, ok, ok anyway thank you so much for sharing with us.

AFP2_0008 : You, you need plain water I don’t have gassy…

End of interview
