# Supplementary material for: Patient perspectives of the Self-management and Educational Technology tool for Atrial Fibrillation (SETAF): A mixed-methods study in Singapore
Source: PLoS One. 2022 Jan 21;17(1):e0262033. doi: 10.1371/journal.pone.0262033 (PMC8782297; doi:10.1371/journal.pone.0262033)
Supplement: S1 Data — (ZIP) [file pone.0262033.s006.zip › AFP2_IDI_0006.doc]

Participant ID number : AF_IDI_0006

Interviewer Number : I_01

Length of interview: 36.53mins

I_01 : Ok so thank you very much for taking part in this study so the first thing is how long have you been diagnosed with AF when did you know about it?

AF_0006 : Oh no about maybe 2013, 13 about a few years back.

I_01 : Ok, ok so was there any like medication that was prescribed to you?

AF_0006 : Yah after that the one I think go for some op- operation then after that taking the warfarin…

I_01 : Oh warfarin…

AF_0006 : And some don’t know the blood heart one about heart the, the, the control the blood pressure something…

I_01 : So you found out at the where did you find it find out your…

AF_0006 : Actually I go to my this my company doctor then which is they detected got some AF then after he refer me to hospital “N” to see the doctor then from there then after that was, was refer to see the some cardio doctor.

I_01 : So that was in 2013?

AF_0006 : I think so I think now is 201…

I_01 : Or before 8, 1…8… 2018?

AF_0006 : I’m not so sure about should be at least at least got 5… 5… 4, 5 years already…

I_01 : 4, 5 years ok.

AF_0006 : 5 years already.

I_01 : About around that around that time.

AF_0006 : 20 about 2013 correct 4, 5 years about 2013.

I_01 : Ok, ok so other than the medication what other things do you do to help you managed your this condition?

AF_0006 : Well it means everyday whatever the doctor say it because you are taking warfarin if some internal bleeding may happen so you have to observe yourself you from the stools or something and any the bliss like how to say like some bleeding or something. If you say a cut or something say if it say a big cut or something must straight away go to hospital because the, the, the blood will keep on continue flow out you cannot stop by yourself unless a minor cut.

I_01 : So that’s what they told you?

AF_0006 : Then and then also the there some control on the some di- diet some food that you try not to take then if you say those food that you taken had to be almost like evenly not say too much or too, too little something you take mean daily every time you take the same something like that. Control the food that’s all don’t take too much don’t take too less also everyday maintain the same thing then ok.

I_01 : Consistent…

AF_0006 : Consistent!

I_01 : Ok then any other things that you do to help you to manage your condition or not any other things outside of these you know what your doctor told you right and the dietician told you right outside of, of all these things?

AF_0006 : Outside no I think not so much because after the thing I have been doing some, some meditation doing some meditation just from that to cool down or some something like that.

I_01 : How often do you do it?

AF_0006 : Ev- almost everyday…

I_01 : Everyday?

AF_0006 : I coming to office and do everyday do because this is daily you have to practice everyday to keep you like I do for the meditation for at least will be half an hour one hour at least.

I_01 : Around what time you I mean…

AF_0006 : Normally I come to office about 7 plus I start 7 thirty 7 twenty I start until 8 o’clock half an hour like that continue then sometime also I do outside do some how to say those helping others also because my teacher is teaching this some it’s also I just learn, learn from them and then ss-…

I_01 : Helping others means like volunteering?

AF_0006 : Volunteer job like that because, because some of the patient they, they come see my, my, my lao shi [Means “teacher” in English] actually is a zhong yi shi. [Means “Chinese physician” in English]

I_01 : Ok Chinese one.

AF_0006 : Chinese one…

I_01 : Chinese medicine…

AF_0006 : I never take the med- medicine I take the, the warfarin I never take Chinese it’s only I learnt from her is only the meditation you all is do for your this those mer- meridian or something jing luo. [Means “meridian” in English]

I_01 : Jing luo [Means “meridian” in English] ok, ok.

AF_0006 : All these thing so just to practice learn and then at the same time those people who learn how to want to want to learn we, we teach them how to how to learn it’s a make volunteer job then to make…

I_01 : Volunteer is to help them help to teach other people meditation.

AF_0006 : Not, not for this AF it’s only to keep yourself the, the maintain your this how to say the resistant or something immune system improve or something like that so I don’t whether that will cause to, to help my this also I not so sure.

I_01 : Yah so when did you find out about this like this, this tasking?

AF_0006 : I think after the op- after the op- I think after the op- for maybe a year or something then I, I found this it’s somebody introduce say that this thing whether you, you, you keen to learn or to, to I say ok don’t no harm because I also doing thing very fast wait I you know? So I cannot keep myself also busy so he say better calm you down something, something like that so I learn from there.

I_01 : Your friend recommend one?

AF_0006 : He say if you…

I_01 : Ok, ok then what about other things like any other things that you do to help yourself maybe…

AF_0006 : Other than that I think for…

I_01 : Could be like diet exercise could be diet…

AF_0006 : Exercise because I at place “J” because normally I, I like prefer walking something walking, walking so that’s all only but because if said too excited those exercise I, I will not…

I_01 : You won’t do that one.

AF_0006 : Because everyday working until maybe 8 o’clock then I go home so there is no time for me to exercise also I only on every like Wednesday I go for the, the class teaching class something like this meditation something like that just relax from there for one hour then Sunday also go to another place to teach something like that.

I_01 : Oh ok so it’s a few location.

AF_0006 : 2, two location, correct.

I_01 : Ok, ok so do you like do you try to find out anything about AF or not other than you know your doctor told you all these things right you go and find out…

AF_0006 : Yeah because sometime I…

I_01 : How?

AF_0006 : I yah sometime I go to the search for my the, the thing because sometime they AF they called Atrial fibrillation…

I_01 : Correct.

AF_0006 : It’s also they called air flut- fluctuate flut- flut- flutter, flutter, flutter something…

I_01 : Flutter yah.

AF_0006 : So actually I start confuse whether what [Interviewer laughed] is the this 2 term I keep on go internet and search a- fibrillation and flutter something…

I_01 : Yah correct.

AF_0006 : Yah because sometime I check myself I found like something my heart is like pumping maybe like not so not so consistent because this one is only check on pressure…

I_01 : Correct, correct yah.

AF_0006 : They never check from the heart rhythm or something right so I found myself sometime I find like rhythm look like the slow fast slow fast slow so whether is it due to that the AF or the, the flutter so I this one I confusing sometime the question asked also quite for me whether is AF. I know is I have AF but that flutter is during the operation come out then I have the flutter the doctor told me you have flutter so they do some ablation or something then…

I_01 : Yah ablation.

AF_0006 : Also, also come sometime have occasionally it come back occasionally come back again…

I_01 : Ok, ok so…

AF_0006 : So actually I not so…

I_01 : You, you not very you, you find that these 2 concept…

AF_0006 : I don’t know whether it belong to AF or belong to the flutter…

I_01 : Flutter so you are a bit confused between these 2 terms ok…

AF_0006 : So you must go for the, the doctor for the this I think ECG or something…

I_01 : ECG.

AF_0006 : To do go ECG then you are able to trace whether is AF or, or the flutter or something because my hands I, I feel sometime my, my, my this rhythm is actually sometime fast slow fast slow that, that is the, the thing that the rhythm out already so but that is belong to the flutter or belong to the AF?

I_01 : So you, you have this idea that, that they are they are different?

AF_0006 : There, there is different but I cannot tell what is the different. [Participant laughed]

I_01 : Ok, ok actually how do you find out that they are different like, like…

AF_0006 : Because the when I do for this I actually stated AF then I go for my op- go for the op- the op- is actually my got some heart hold on the valve issue…

I_01 : The ok, ok.

AF_0006 : So they repair the valve for me and then at the same time they also repair for me the AF so they said that this is already AF no more because I followed up then they said AF ok settled but after this operation yah come out they, they check something that is they got flutter. So because of something then become a flutter come out so I also confused that AF he said AF don’t have but your you have flutter then I also confused whether the flutter because of the rhythm he said the rhythm…

I_01 : Yah the rhythm…

AF_0006 : Is inconsistent or something so now until now I also confusing whether I still have the AF or not and I need to go back because every 3… 6 months or 3 month I need to go to hospital then the doctor check so I may ask the doctor again so whether is, is I belong to still AF or not or only the flutter.

I_01 : Did the doctor tell you or not the last time you go?

AF_0006 : Last time the doctor tell me is…

I_01 : Got asked him or not?

AF_0006 : He said AF no more after that never go for the, the, the ECG so I, I can’t tell whether they come back or not I’m not sure…

I_01 : Not sure you have to do more test.

AF_0006 : Yes every time they did if you say you require something then they may go ask you to go for ECG then they can tell if not they can’t tell from this heartbeat sometime I touch like that he said no, no this one is flutter not belong to AF your AF settled so I’m not sure…

I_01 : So you, you planned to ask the next time you go you go and the doctor?

AF_0006 : Yah go and see the doctor then I ask him how whether is it AF come back or because due to flutter because the rhythm also AF also because of the rhythm also.

I_01 : Correct yah, yah.

AF_0006 : Same the thing the flutter…

I_01 : Because they related yah.

AF_0006 : So how exactly I’m not so sure [Participant and interviewer laughed] internet see, see also confusing so not so sure.

I_01 : So the internet doesn’t really give you the information?

AF_0006 : Yes, yes, yes.

I_01 : Ok, ok so like during this what about like your friends and you know family members do you like asked talked to the them about your, your, your condition?

AF_0006 : Yah I talked to them I talk I sharing my, my, my this turn happen then also the, the operation side how also sometime I share with them…

I_01 : You shared with them?

AF_0006 : Shared with yah.

I_01 : Ok so do what kind of advice do they give you like you know?

AF_0006 : No, because they also never come across this [Interviewer laughed] so I don’t think but at least some information passed to them at least they, they next future they could feel anything which is this one then they know what or maybe I got this issue or something like that.

I_01 : Ok, ok then during this, this 6 weeks of this study when you were doing this device using the device you know what is your routine everyday how is your routine like? So that means like what will you do first, and what do you do second and what do you do third how is the, the sequence like?

AF_0006 : Oh no I think normally I maintain whatever I would normally when I wake up I, I would I would just check my blood pressure something like that…

I_01 : Oh ok when you up?

AF_0006 : Then after that I wake up I checked already I checked my like this thing then I go for breakfast something come to office already then do my meditation then working continue until night time then go back home then watched TV do my things. Sometimes until maybe sleep time about 11 sometime 12 something like that so before I sleep I take again something like that that’s all this is my daily thing.

I_01 : So morning you take…

AF_0006 : Morning wake up take and then before sleep take.

I_01 : Every day like that?

AF_0006 : Yah right.

I_01 : Got take any, any other day or not any other like…

AF_0006 : Have there’s something I feel the blood...

I_01 : Do it other time?

AF_0006 : The pressure sometimes, sometime too high or sometime one three something one four something is abnormal because normally I take one more time I will take one time take another time sometime you see my record got 2, 3 times because…

I_01 : Yah correct yah I saw that.

AF_0006 : I want to confirm again I want to confirm whether is it ok or not that’s all only what because…

I_01 : Oh just to…

AF_0006 : Sometimes because of maybe the, the, the flutter or the AF caused the thing adjust something a little bit because the heart sometime the sign something come up so maybe due to that so I reconfirmed that why I take 2 or 3 time more than…

I_01 : Ok so when you take your, your, your this blood pressure right do you have any emotions that come up or not I mean how do you feel?

AF_0006 : No normally I take because I since already before this I already buy one bought one those blood pressure monitor every day I also take so…

I_01 : So you already have it?

AF_0006 : To me to me is very use used to it because that’s why when the survey come to me said everyday I said can everyday because everyday I also taking what I got no harm taking then let the doctor review if any different or not like that.

I_01 : Do you feel any like anxieties stress…

AF_0006 : No.

I_01 : If you no, no don’t have ok, ok then how do you find easy is it easy to use or not the, the, the, the blood pressure monitor?

AF_0006 : It’s easy it’s easy right…

I_01 : Easy or difficult or ok?

AF_0006 : It’s, it’s easy ok.

I_01 : Easy yah!

AF_0006 : Because everyday just do this way it’s ok.

I_01 : Ok and you, you are also used to it already ok then what about like ok you know there are some surveys that are sent to you in the in the app, app right how do you find the surveys?

AF_0006 : The surveys I find I just answer because certain things I also not so sure what are, are certain things certain questions I’m not so sure I, I put don’t know those I know those that I think I understand one I will ticked whatever thing answer to them. If not so sure one because I also not so sure what they are asking and the thing whether will, will caused this, this or not I also not sure so I just put I don’t know.

I_01 : Which questions are, are those…

AF_0006 : Certain things…

I_01 : Those not sure…

AF_0006 : Certain things like…

I_01 : About what one?

AF_0006 : I forgotten already it’s something like… I can’t remember I certain thing that I, I also not sure whether like the you see the, the AF is it caused, caused by the something re-related to stroke or something that one I sure I know that the AF they said because of the my heart pressure heart pump pressure. Sometime pumped slow sometime pumped fast…

I_01 : Fast yah!

AF_0006 : So if you say the warf- the warfarin if I never take the warfarin dilute all the blood they then clogged up then if you said the thing suddenly the formed up then they go, go to your heart your brain something will become a stroke something so I, I know that one. The doctor got tell me this and that is because the issue of the, the pumping sometime slow sometime fast you see that’s why I need to thin the blood that’s all only. Now I, I very care on this whether from this AF will caused die or not so I said no, no because to me I, I, I, I not sure whether the AF is to the normally it to the blood pressure side not tell the doctor also tell me said the AF can caused you suddenly die or something so I said no or maybe I put don’t know I also not so sure.

I_01 : For the survey one?

AF_0006 : Ah the survey!

I_01 : The survey will you find that the, the wording too difficult or not?

AF_0006 : Ok because the thing that which I come across is the doctor already got some tell me something so I more or less know what they asking so it’s, it’s still ok.

I_01 : Ok so it’s still ok, ok then do you find it too long the survey?

AF_0006 : Ok.

I_01 : Too long?

AF_0006 : Not, not so not so long ok, because when they asked you just tick, tick whatever they want to ask I know one just answer to them .

I_01 : Ok, ok then like what about like you aware of the videos do you watch any of the videos the video yah…

AF_0006 : The video I watched I watched.

I_01 : Ok which one you, you like?

AF_0006 : All the video that mean they put sometime they put the thing how the thing work something but some of the thing I not so I not don’t not so understand just see the thing the diagram flow the blood from here go up then come out from here then go up. So I’m actually figuring my which are the heart burner side is belong to what the flippers or something like that.

I_01 : You, you mean don’t understand is, is that the, the, the, the language the language is it?

AF_0006 : Because not I don’t think they got put some they got some they put the, the diagram the, the blood flow is from here or something then they pumping then come up come down come up like that only.

I_01 : You don’t understand is which part which part of the video?

AF_0006 : Whether the this one is be belong to the, the, the, the…

I_01 : Shorter…

AF_0006 : Pressure got 2 got 2 right now one is on the high one, one is on the low one whether this one if go up is consider what the, the blood pressure don’t know systronic or something I don’t know belong to which one I’m not so sure.

I_01 : Ok, ok so that, that part of not, not very not very clear.

AF_0006 : So whether the, the high one is actually from, from heart pumped up or, or the some is the lower is the back return or something that one I’m not so clear.

I_01 : Ok, ok, ok then the which ok the videos you, you aware that, that there are other videos also right there…

AF_0006 : A few videos I think I think I can’t…

I_01 : Which one is your favorite that you like best?

AF_0006 : Forgot which one there’s, there’s one quite, quite ok one I forgot which one already you know never…

I_01 : You don’t really think about it?

AF_0006 : Never, just see or whatever just watched the thing but never really go and whether this one useful or not because whatever thing I said yah I know the thing that’s all I just see yah ok.

I_01 : Ok but like what about like the overall like how do you feel like whether it helps you or not the videos?

AF_0006 : Overall is overall still ok.

I_01 : Ok then you aware you’re aware there’s also the cooking one the exercise the video how do you find those videos?

AF_0006 : For me o- for me just an a knowledge like that you know just, just for some information some knowledge that’s all.

I_01 : Do you go and like after when you watched the video like seek it you know used that advice to you know your own life and like that…

AF_0006 : Some, some of them I think is I’m already something practice some, some of them I’m not so ss- or, or even got no time to, to…

I_01 : No time.

AF_0006 : So already working so late that mean want to do something so I never follow.

I_01 : So just listened and just watched.

AF_0006 : Listened, just watched give me some idea and some knowledge only.

I_01 : Ok, ok then what about the pop up messages there are some pop up messages right you know give you advice right you know those encouraged you encouraging messages do you how do you find them?

AF_0006 : But I don’t know what is that every time popped up saying messages are ok then something like that I don’t know what is that use for? [Participant and interviewer laughed]

I_01 : But how do you do you feel that it’s useful or not?

AF_0006 : That one that one is every time popped up they said you are doing well then suddenly you know the thing to me is nothing you know…

I_01 : Oh nothing!

AF_0006 : Don’t know whether how is doing well or not doing well I also not sure.

I_01 : Oh you also not very sure so you don’t know you don’t really you not so sure what is it for is it?

AF_0006 : Why, why they prompt me say your, your blood pressure is excellent or some something it’s only then but this one I, I also not sure why they popped up to sent maybe they from the record that my reading is actually all within that range so maybe they just tell me very good, then ok acknowledge ok.

I_01 : Just like that.

AF_0006 : Just like that only.

I_01 : You got feel anything when you when you see these kind of message also do you feel like you know in any like any emotions or not?

AF_0006 : No, no for me ok.

I_01 : Don’t have ok.

AF_0006 : For me already fixed already [Participant and interviewer laughed] already accepted you have to accept that’s all. [Participant laughed]

I_01 : Ok, ok, ok can then what about like the ok so like you, you all these features would you like to see any, any oth- any other things that we can add on to this features tablet or not?

AF_0006 : Actually to me if you say because, because every I think I don’t know whether this survey is actually for more on AF right?

I_01 : Yah correct.

AF_0006 : So in AF they will actually need to take, take the some medicine is it like for warfarin, aspirin or something right so whether that if you say people who take warfarin then maybe they have to come up. Because some people they may have forgotten they if they anything internal bleeding or some occasionally then they have go and must quickly go to hospital or something so this one I think put inside the video or something you know something precaution some let the people the patient know about it then I think better.

I_01 : You mean like medi- the kind of medicine?

AF_0006 : The medicine that if you say now if these people taking warfarin you have to monitor your, your, your, your, your blood whether got internal bleeding or not because this one is the internal bleeding is if those people don’t know those black, black also don’t care then maybe some have something happen. Then if you have got a cut a big cut all these thing if you say stopped you cannot stopped yourself you better go for hospital something to, to treat something put up some video at least people aware of my, my symptom of taking these, these medicine I need to aware about this. Taking aspirin maybe still not so serious because warfarin is every 3 month something you need to go for monitor your this index blood index something at, at or something, something like that so that put inside the video at least because I’m aware so I know. Some people those maybe older people or something like that they don’t care one also they don’t know then there is a concern…

I_01 : So those is like medicine related kind of videos that can…

AF_0006 : Something when they take this one be, be careful this you take one ok some aspirin I think it’s ok I think but effectiveness is this one may be better this one not so effective I mean also see individual people.

I_01 : Ok so more, more of that, that kind of videos so like ok then this ti- period of the study 6 weeks do you find that I mean is it how do you feel about it was it too long too short ok you know?

AF_0006 : Ok to me it’s, it’s still ok.

I_01 : Ok with that…

AF_0006 : I think if you say you monitor a pressure I think maybe 4 weeks 3 to 4 weeks should be enough because if you say your pressure blood pressure is consistent this way I think you can stopped already. You no point keep on continue because you know that the pressure is keep on maintaining then no point takes 6 weeks or one year or something.

I_01 : Ok so like yup I mean like you, you because we also want to ask about like let’s say if this was like if we keep on asked you if let’s say it’s off the shelf right or if, if it’s part of your medical care right. Would you want to like you know would you think that it’s something that you would buy like and then like you know it can it can last you like for a long time you it’s part it’s a long, long term medical care?

AF_0006 : You know of course if say you if you say you need to have this thing to monitor I think better everybody keep one at home that is better…

I_01 : Ok can.

AF_0006 : That’s why I, I buy it.

I_01 : Yah if, if you if you can yah. Ok then like actually I want to ask a little bit more about the, the, the tablet the just the fea- the, the interface ok so like the screen was it too small or too big or just ok?

AF_0006 : You mean the tablet?

I_01 : Yah the, the screen how do you find it?

AF_0006 : Ok the screen is ok.

I_01 : Ok then the font the size of the font…

AF_0006 : The font is ok the font ok, ok.

I_01 : Ok means like alright is it ok means al- alright…

AF_0006 : The thing alright it’s the, the, the yah because whatever the, the, the questions also they the whole screen only maybe they only occupied maybe the top, top portion something so you still got space so it’s still ok.

I_01 : Ok then what about like the, the brightness?

AF_0006 : Brightness you can adjust!

I_01 : Can adjust ok, ok then what about like the like navigating the app you know going to like inside out like go in and out of the app how, how do you find it is it intuitive for you or not?

AF_0006 : To me it’s, it’s ok it’s only that every time when you put up the thing sometime the, the thing just vibrate you know vibrate by itself and the and the light appeared so sometime like I found why is necessary the thing have to be appeared and vibrate?

I_01 : And vibrate…

AF_0006 : I don’t know whether it’s something that…

I_01 : Related…

AF_0006 : Prompt popped, popped in or something?

I_01 : Popped message?

AF_0006 : Something like that I don’t know…

I_01 : So you…

AF_0006 : It’s already midnight already sleep already then hear sometime got something like you know got something like brightness that is a the thing so I think not necessary because you already sleep you popped up any message also you can silent because you silent but he the thing is still vib-…

I_01 : Vibrating yah so…

AF_0006 : So it’s silent put to the, the lowest already it’s still vibrate.

I_01 :Do you find that a bit like, like this like…

AF_0006 : Irritate…

I_01 : Irritating yah is it irritating?

AF_0006 : Yah.

I_01 : Oh ok, ok, ok, ok so it’s a bit irritating the vibration…

AF_0006 : It’s silent vibrating…

I_01 : And you don’t why they are doing this?

AF_0006 : It mean silent means silent.

I_01 : But it’s not it’s vibrating.

AF_0006 : Because normal you know that I, I morning take night time take then in between in between no need.

I_01 : And you don’t know why is they…

AF_0006 : You already working they know no point you still vibrate for what still like for what you know.

I_01 : Ok, ok can ok then did you encounter any problems or not in the in this time peri- like peri- with any difficulties…

AF_0006 : No, no so far no.

I_01 : You know in, in like ok so it’s ok. Ok which one do you of this overall this features which one was it easy, easy for you or which one was it not so easy for you?

AF_0006 : To me is, is normal because everyday we using computer so we know what is what is what then we also using so for me it’s ok I found ok.

I_01 : It’s ok yah your ff- all of it?

AF_0006 : Yes, yes.

I_01 : Ok, ok then ok so when Miss “M” she was the one who gave you the device right?

AF_0006 : The lady?

I_01 : The lady right so did she give you any instructions about how to use or not?

AF_0006 : Yah have.

I_01 : How do find is it useful to have that?

AF_0006 : Yah of course because if those people who never touch on all these tablet meaning they don’t know how to operate but for, for me it’s ok because I never used tablet I never used tablet I know the hand phone the tablet is almost the same. Just button on off here like or something slide down whether you want to see the battery life or something then ok for me it’s still ok only for those people who maybe seldom touch on these type of thing maybe for them it’s, it’s a problem even now you tell them maybe later they forgotten.

I_01 : So let’s say if Miss “M” didn’t go down I mean she, she, she went down she gave you the device but she didn’t explained to you will you be able to use it?

AF_0006 : I may have to explore myself see how the thing.

I_01 : But you will be ok with it ok, ok then will you aware that there’s also like a number on the tablet or not like say you can call and…

AF_0006 : Yah I called the number…

I_01 : Did you try calling it using it?

AF_0006 : Yah I called up the thing that because during there’s one time 3 day I was away to Malaysia so I cannot bring this thing to Malaysia so then I think is ok so I just leave it at home only that one I called up one time.

I_01 : Did anybody answer or not?

AF_0006 : Yah, yah.

I_01 : So…

AF_0006 : Answer the person the person give to a the person who is who is not belong to this department or something then they asked me to maybe to call another number something then I call that number to look for…

I_01 : So it referred you to another num- number.

AF_0006 : Another number also because it’s not that number…

I_01 : Oh so…

AF_0006 : So actually this is the number it’s, it’s about the thing my these survey thing he said no [Interviewer laughed] so he say ok maybe I give you another number something I go for another number I forgotten something like that.

I_01 : Was it troublesome?

AF_0006 : Yah of course the number given is not the, the one then no point…

I_01 : [Interviewer laughed] Ok, ok so, so you…

AF_0006 : Because sometime you called the number is actually engaged one hospital ”N” something the, the, the phone is not easy to call in.

I_01 : Oh how many times did you call tried calling in?

AF_0006 : No I happen that, that one I called I think 2 time or 3 time then I get I happen to get something like that only.

I_01 : Ok so 2 or 3 times then you managed to get through.

AF_0006 : Get through then refer me to another side another person don’t know got too many…

I_01 : Ok, ok, ok , ok then did you man- talk to the other doctors right about the device when like you know like you know the, the it they can track your blood pressure right subsequently did you go and see any doctor then you bring this device…

AF_0006 : So far no…

I_01 : Oh not yet.

AF_0006 : So far no so far because I haven’t see any doctor only I think coming, coming May there’s one appointment…

I_01 : Ok so…

AF_0006 : For the doctor.

I_01 : Ok so do you think that like say in that appointment would you bring this let’s say you have this device would you bring it and, and, and show your doctor or not?

AF_0006 : May not because that one you don’t have a history something I found there’s no history.

I_01 : Oh there is a tracking list…

AF_0006 : Is it?

I_01 : You know yah also…

AF_0006 : Yah, yah there, there is a graph there is a graph…

I_01 : Correct, correct.

AF_0006 : Maybe I can I can maybe if forget I can still can show to the doctor there’s something that start from here then 7 day 28 day and I don’t know how many day only that one yah.

I_01 : But you but you actually before that you, you, you didn’t know that there was this thing…

AF_0006 : First I don’t know…

I_01 : Like history.

AF_0006 : Then I skipped on there is something there I see 7 day of the, the, the graph…

I_01 : A graph right?

AF_0006 : Something like that only the graph.

I_01 : Yah ok but be, be you were not very aware of it right just now I mean because when I you said when I asked you whether you, you know or not…

AF_0006 : Aware, aware because…

I_01 : You’re aware already!

AF_0006 : I forgot I thought…

I_01 : Oh you forgot about it ok.

AF_0006 : My, my device one without the tablet one is actually my this monitor you pressed they got a history something this day then the num- the, the, the blood pressure and your heartbeat then another day one another day one that one don’t have a graph but this one is different the tablet have. But your this monitor don’t have this don’t have this history I mean the monitor…

I_01 : Oh ok actually they have also.

AF_0006 : They have! [Participant laughed] I don’t know what.

I_01 : So you are not aware of it.

AF_0006 : I’m not aware about the monitor got history so because I see from the tablet the, the, the, the…

I_01 : You only know about the graph…

AF_0006 : The graph one.

I_01 : But not the history part.

AF_0006 : Not, not the…

I_01 : The tracking part…

AF_0006 : Not the not the monitor history part I thought don’t have I thought this one is only every daily something like that then linked to the tablet so I, I don’t know that the monitor itself also got the history.

I_01 : Oh ok, ok so you are not aware of it you mean the it…

AF_0006 : Monitor have? I don’t know I pressed, pressed I…

I_01 : No the blood pressure monitor you mean, the blood pressure monitor don’t have…

AF_0006 : But I don’t know…

I_01 : But the tablet have…

AF_0006 : Ah tablet have!

I_01 : So you are aware of the tablet has.

AF_0006 : I aware the tablet know that have is only the graph, graph only.

I_01 : Ok and also the, the date and all that.

AF_0006 : The date yes the mean the below they state from the, the know which date start or something then the, the one, one week and then 28 day one how, how the graph becomes smaller only that I know the tablet but the monitor itself never see the history.

I_01 : Ok the monitor don’t have…

AF_0006 : Don’t have.

I_01 : But the table the tablet have.

AF_0006 : Tablet have I know.

I_01 : That and also the history also.

AF_0006 : History that one what how they look like I not sure?

I_01 : It’s like I don’t whether I you know about it I just show you…

AF_0006 : Sure, I only see only I see the graph.

I_01 : Is it on?

AF_0006 : Maybe no battery already I keep for…

I_01 : No battery already?

AF_0006 : I keep for sometime… inside have?

I_01 : Wait any anyway just want to ask also like do you find that this tracking is useful for you or not this history like for your own purposes?

AF_0006 : Yah also because I see every time yah every time I see the morning time is actually on the higher when sleeping time is normally lower you can see the graph show the, the, the, the symptom like that normally night lower morning will be higher like the pattern is the same like that.

I_01 : I see I just ok I think never mind I just mentioned I guessed probably off. I mean this I, I, I don’t know how’s ok so the next one would be like how do you feel about like, like it do you think that this, this device can help you in term of your in terms of your life like a lifestyle or not like in terms of your…

AF_0006 : No.

I_01 : Not…

AF_0006 : This is only monitor your pressure how you how to track your lifestyle I don’t know. [Participant laughed]

I_01 : Like maybe you can the, the information that you get can help you to change your, your habits?

AF_0006 : Maybe the information that they prompt and then, then they say something that one is just to me is a knowledge how to whether…

I_01 : Whether you…

AF_0006 : Anything yah or maybe do this got something to be controlled or something ok that is like to me is a knowledge.

I_01 : Ok knowledge ok then let’s say if these kind of app is like in a in a ok which kind of device would you prefer like a tablet form a phone a computer all of it you know how with how would you what kind of device would you prefer these…

AF_0006 : I think the device wise I think because some of them they, they don’t have tablet phone everybody have so through phone is better and then also sent by email that mean the phone prompt you can see from your phone sometime it also go to a, a desktop or something need to see. From the email then you see that is easier…

I_01 : Oh it’s easier.

AF_0006 : That is faster you seethe thing quickly you checked, checked, checked ok I want to see detail more clearer or something I go to my computer to see.

I_01 : Ok, ok so you don’t mind if…

AF_0006 : It’s a prompt, prompt me something.

I_01 : A, a few device ok at any kind of device that you, you prefer more like than others you know what I mean there are all these device right which one will be the best for you?

AF_0006 : I think hand phone is faster…

I_01 : Hand phone!

AF_0006 : Hand phone is faster.

I_01 : Ok mobile, mobile phone that, that the form?

AF_0006 : Mobile phone is faster!

I_01 : Ok, ok so let’s say if it’s a paper form right would you like would it be ok for you or not like if this kind of app and the you know these kind of monitoring is via a paper that means like we…

AF_0006 : But, but…

I_01 : Paper meaning like, like ok we have like say a brochure some brochures for you for the information part right and then like for tracking part right you take the blood pressure then you write it down then you e- you mailed to us would you…

AF_0006 : I think I think…

I_01 : Would you like…

AF_0006 : Paper is not advisable because too, too many too many thing then you need to record or something in- instead of app faster everything go to there I don’t need to care what is the thing just linked to there I cannot do any cheating. [Interviewer laughed] Or cannot do any false report also directly go linked to there.

I_01 : Ok, ok so you…

AF_0006 : If say write one I can write whatever thing I preferred one. [Participant laughed]

I_01 : Ok, ok so you also feel it’s more accurate the, the this kind of direct linked.

AF_0006 : Yah direct linked no need…

I_01 : More accurate ok, ok then ok just a few more questions would you when would be a good time to let’s say if this one there’s this kind device when you first was diagnosed with AF right would be a bett- would be the best time to introduce it to, to you? Like when you when you first find out or mid, mid way through or maybe…

AF_0006 : Yah I, I think in you say you already have detected the AF I think from there you monitor better because at least you know that before your op your AF one is the reading of blood pressure something. Or, or some in, in regulate heartbeat or something then when after your op come back what is the different so you can track the different.

I_01 : So as when you that means like when you first found out like your AF you, you think that like when it’s introduced that time it’s ok it will be the best time?

AF_0006 : Yes, yes like monitor the thing that means they found every time my, my AF yah the rhythm is so much different and then you better is maybe some up sometime up like something like that then after your op then become stable something then there, there is a different.

I_01 : There is a different…

AF_0006 : If not you don’t know previously what is my blood pressure what is my rhythm I can’t tell.

I_01 : You can’t tell.

AF_0006 : After op you tell me this one is abnormal or normal I also cannot tell.

I_01 : You cannot tell ok, ok so the tracking part is quite useful for you?

AF_0006 : Yes.

I_01 : Ok so ok what how I mean what would you think would be like, like other people right when they when they let’s say if would you think that they would be willing to buy something like that or not or, or maybe like you know…

AF_0006 : They, they will buy…

I_01 : In their care plan…

AF_0006 : It’s only that depend on what kind of people if they can afford to buy of course they will buy it like those people can afford where they have certain con- constraint of the money you also for them even they want to they want to buy they also cannot afford to buy.

I_01 : Yah that’s right yah.

AF_0006 : They want to track they almost you know to them money already they cannot able to buy this tablet.

I_01 : Would you think ok so like finally right is it any other like features would you like to see in these kind of app and device or not because we want to improve it. And maybe also like you know used it for more, more patients in the future you know to, to improve the healthcare system in Singapore?

AF_0006 : Maybe, maybe to improve just now I, I mentioned about what is AF and what is flutter also actually flutter so I mean because put out the thing then let people aware ok all these thing then also tell people when AF after the AF you, you opted for the thing. They may also like lead to another, another sickness that like for me if after do the thing maybe they said that your blood pressure may cause higher that’s why I need to take the another for the blood pressure one take half tablet something to, to reduce my this blood pressure. That’s why you see my blood pressure is, is quite low 100… 110, 100 sometime one zero something is because I also taking some…

I_01 : Medicine.

AF_0006 : Medicine that the, the, the, the doctor give to me one because last time I don’t have any high blood my blood pressure only 120 for 80 after my op the blood pressure may go up to one 4 something…

I_01 : Oh really?

AF_0006 : One five something so that’s why they asked me to take the, the pressure…

I_01 : To lower it.

AF_0006 : Lower down because of the op just start starting time the, the, the early stage the blood pressure is actually higher so this is one of the thing that they cause the thing so if this information can put into the tablet then the people will know my pressure is actually normal. Now because I have op this one may caused this not, not at hardly everybody get the…

I_01 : Yah correct.

AF_0006 : In console some people may get it that’s all.

I_01 : Ok so you…

AF_0006 : Because these information to the patient is good.

I_01 : So medical information that is…

AF_0006 : Put up then, then also I found that this is only English they don’t have Chinese…

I_01 : You can you didn’t have the transcription the self transcription yet?

AF_0006 : Have, I don’t know I hardly [Inaudible segment] all English one.

I_01 : Don’t have yah it’s only English for now but they, they I mean of course it’s definitely also important yah so you feel that if there is other language will be more helpful…

AF_0006 : Yah, yah if...

I_01 : Like Chinese.

AF_0006 : Those like the old people they, they, they are Chine- Chinese they want to read Chinese also they are able to understand if not then they only give to those people who said if they must understand the English. The Chinese educated Chinese people only can read Chinese they don’t know you give to them you cannot survey these type of people.

I_01 : Were there anything that you didn’t understand like in the, the, the ba- because of the language?

AF_0006 : Some, some of the, the medical term those words I just googled checked this one. [Participant laughed]

I_01 : So you went google and checked it.

AF_0006 : Go to checked, checked what is the thing what they are referring to this meaning because medical term sometime we not sure…

I_01 : Ok so were there a lot of medical terms you feel?

AF_0006 : No, no not, not many.

I_01 : But you couldn’t understand some of it?

AF_0006 : No, some of it only.

I_01 : So, so you have to go and search after that?

AF_0006 : Yes, yes, yes.

I_01 : Ok, ok, ok any other like things that you feel that can be added to this to make it better you know?

AF_0006 : No more I don’t think so, I think quite ok.

I_01 : Quite ok, ok, ok , ok so actually I think that’s about it for, for thank you for sharing with us so I stopped the interview.

End of interview
