# Supplementary material for: Patient perspectives of the Self-management and Educational Technology tool for Atrial Fibrillation (SETAF): A mixed-methods study in Singapore
Source: PLoS One. 2022 Jan 21;17(1):e0262033. doi: 10.1371/journal.pone.0262033 (PMC8782297; doi:10.1371/journal.pone.0262033)
Supplement: S1 Data — (ZIP) [file pone.0262033.s006.zip › AFP2_IDI_0005.doc]

Participant ID number : AF_IDI_0005

Interviewer Number : I_01

Length of interview : 34.30mins

I_01 : Ok, ok, ok so thank you for taking part in this study so for how long have you been diagnosed with atrail fibrillation or irregular heartbeat?

AF_0005 : Very long very long 2008.

I_01 : How do you know about it?

AF_0005: I was admitted to the hospital.

I_01 : Oh for what?

AF_0005: For stroke transient stroke then only I know I have AF that time I have the AF the polyclinic they checked already irregular heartbeat they sent me to the “N” hospital then already monitored I have AF but they feedback to the polyclinic no, no the doctor never what?

I_01 : Never in informed?

AF_0005 : Yah never follow up never follow up…

I_01 : Never follow up ok never follow up.

AF_0005: Then after that I got the stroke transient stroke then only they find out then hospital “N” said last time you you’ve been here to check your heart why nobody follow up I said I don’t know doctor also never give me medicine for that, then until I got the AF got the stroke then only they monitor.

I_01 : So only when you got the stroke then they started to moni- started to actively monitor you ok, ok before that don’t have?

AF_0005 : No.

I_01 : That was in 2000 and 8 ok then after subsequently right do you for you, you take medication right?

AF_0005: Yah first I take warfarin, warfarin I take already got the blood…

I_01 : Ok you’re bleeding and bleeding right?

AF_0005: Yah blood bleeding, bleeding only all don’t, don’t know from where then they stopped they stopped then they changed to other tablet then but I got the take the warfarin my leg all got the spot, black spot cannot very dan-…

I_01 : It’s bleeding yah.

AF_0005 : Then the doctor changed to what is rivaroxaban.

I_01 : Ok a different one different type one.

AF_0005: This one is better, is better.

I_01 : Ok what, what other things do you take like…

AF_0005 : Cholesterol…

I_01 : Cholesterol medicine.

AF_0005: High blood…

I_01 : Ok, ok high blood…

AF_0005: Heart and heart…

I_01 : And heart one ok.

AF_0005 : That, that for the AF but AF they won’t control you even take the warfarin even, even take the rivaroxaban also like that.

I_01 : You feel you feel it don’t really ok…

AF_0005: Sometime you, you feel breathless you walked a few distant you have to sit down and, and…

I_01 : And rest.

AF_0005 : Rest first then we can walk…

I_01 : So…

AF_0005: Sometime I sometime do housework you see and then do all the then when people called how come you so breathless still have…

I_01 : So they asked you…

AF_0005: Still have still have…

I_01 : Still have it.

AF_0005 : Still have.

I_01 : So you feel that your I mean you feel different?

AF_0005: Yah tired.

I_01 : It’s you, you feel tired.

AF_0005 : Hen chuan. [ Means “very breathless” in English]

I_01 : Hen chuan [ Means “very breathless” in English] ok then like do you go like did they give you any advice for like diet exercise or not?

AF_0005 : Got they said go to exercise, exercise everyday I every time went to I walk from my house to “L” like that that’s the exercise fetch my grandson from school not school from downstairs like that exer- exercise.

I_01 : Any other things that you do?

AF_0005: Mopping the floor the household washing clothes like that that’s all.

I_01 : Then what about diet?

AF_0005: Diet, always take, take what brown rice vege must take more vege other there ok nothing, nothing much fruits…

I_01 : Fruits ok.

AF_0005 : Fruits, take fruits.

I_01 : Ok, ok then all these information is it you got it from the hospital one the doctor or like the dietician…

AF_0005: Dietician also…

I_01 : Dietician…

AF_0005 : Last time I was admitted dietician…

I_01 : Did, did…

AF_0005 : Also asked me not to take which what, what, what vege then I have the warfarin cannot take Chinese herbs but now with, with roxaban rivaroxaban I can.

I_01 : Ok so ok.

AF_0005: But I never take.

I_01 : Never take?

AF_0005: Never take.

I_01 : Chinese, Chinese herbs never take so any other like ways that you find out how to like managed your condition or not from out from other sources?

AF_0005 : Papers sometimes.

I_01 : Papers, newspapers oh ok.

AF_0005: Then from your this one we read from your tablet…

I_01 : The tablet, yah!

AF_0005: That also useful.

I_01 : But before that…

AF_0005: No.

I_01 : Like any other ways or not TV…

AF_0005 : Doctor, TV some you can hear news from friends they have…

I_01 : Friends ok.

AF_0005: Sometimes they, they the blood thinning they take too much the veins don’t know burst got blood…

I_01 : Yah got blood…

AF_0005 : Passed, passed away…

I_01 : Really?

AF_0005: Got some friends…

I_01 : So some of them?

AF_0005: I also worried too thin already the blood.

I_01 : What did he tell you?

AF_0005 : Then he, he said you have blood in your tools you know in, in your gums you feel uncomfortable something is bleeding inside the best is look at your tools…

I_01 : The stools the stools ok the stools.

AF_0005: Stools, stools, stools black my mother have it she, she, she has cancer in the intestine so I also worried then she also has heart problem high blood I think I inherit from my mother then for me I have irregular heartbeat, my eldest daughter have very fast heartbeat…

I_01 : So it’s diff a bit different.

AF_0005 : Very fast, but…

I_01 : Heart palpitation right?

AF_0005: Then she is doing doctor at the “S” hospital but no, no medicine for her I said must do surgery but she doesn’t want.

I_01 : So do you all you all help one another you, you and your daughter and…

AF_0005: But at least I listened to doctor she doesn’t everything also she don’t she doesn’t want to take tablet don’t want to do surgery I said what you what you want she said never mind die what to…

I_01 : Really?

AF_0005 : Cannot, cannot yah not but she’s stubborn she’s stubborn.

I_01 : Maybe it’s a bit of fear also you know?

AF_0005: Yah maybe she’s 40 only!

I_01 : Yah that’s why you know as fear of you know the surgery?

AF_0005 : Then I said maybe you go for second opinion maybe other doctor like that she said I don’t want to go all over again the procedure take, take test take this I said no, no need computer very easy. Once you pressed you know what you, you have already yah not I said why you like that don’t want to listen boh pian. [ Means “no choice” in English]

I_01 : Yah maybe…

AF_0005: Fear maybe.

I_01 : Fear and need some other encouragement also.

AF_0005: Heartbeat very fast how come maybe is it…

I_01 : You may want to go and ask the doctor.

AF_0005 : Always in the gene, gene she always told…

I_01 : That one actually is cannot can be we, we I cannot tell you also because like it’s better to ask the doctor they, they will know better and then also they will asked about other things…

AF_0005: She’s a policeman yah sometimes she got must exercise right or not cannot do vigorous exercise.

I_01 : Yup, yah it’s part of the work.

AF_0005 : Part of the work, stressed.

I_01 : Stressed yah.

AF_0005: Not even my this daughter also stressed always headache…

I_01 : How about you like do you have any something like that you know stress?

AF_0005: No, yah sometimes have some the children have problem they tell you, you also stressed.

I_01 : How do you manage like the…

AF_0005 : Cannot managed too many you, you cannot sleep the whole night I, I one thing I cannot sleep…

I_01 : You cannot sleep?

AF_0005: Cannot sleep thenI go in early 11 something my husband asked me sleep early sleep even I lie on the bed until morning…

I_01 : Really, until morning as in like…

AF_0005 : 5 something.

I_01 : So the whole night!

AF_0005: Whole night 5 something then I sleep already until 10 I wake up.

I_01 : Why you worry about something?

AF_0005: A lot of thing to worry.

I_01 : A lot of things.

AF_0005 : A lot of thing.

I_01 : What about day time can sleep or not?

AF_0005: Day time, sometime afternoon one hour like that.

I_01 : One hour, so not enough sleep.

AF_0005 : That’s why my dau- my husband said you always sleep in the afternoon no, one not one, two hours not, not, not, not enough to replace…

I_01 : Correct, correct.

AF_0005: Yah or not?

I_01 : And it’s daytime some more right, different.

AF_0005: At night cannot sleep more worst, never drink coffee also cannot sleep, drink tea and coffee sure the whole night cannot sleep…

I_01 : Really?

AF_0005 : Even without these also I cannot sleep.

I_01 : So do you drink or not?

AF_0005: Never drink.

I_01 : You don’t drink you don’t drink try to avoid.

AF_0005 : Avoid sometimes asked doctor to prescribe sleeping pill, cannot…

I_01 : Why?

AF_0005: Sleeping pill can he said…

I_01 : Can get yah…

AF_0005 : Don’t, don’t rely on the, the sleeping pill.

I_01 : Yup can, can get addicted to it, yah.

AF_0005: Last time is I was at work also I asked doctor to give me but not always now outside doctor won’t give me so cannot try to sleep I take calamine lo- calamine tea take the rose tea also cannot…

I_01 : Cannot?

AF_0005 : Cannot!

I_01 : Then what about so now you, you not working…

AF_0005 : [Incomprehensible segment]

I_01 : Are you? Ok, not working already ok.

AF_0005: Never work for 10 over years already.

I_01 : Ok, ok, ok, ok then you know throughout this period of the study this 6 week period so what’s the your sequence and how do you use the device everyday?

AF_0005 : Everyday I see test measure your blood pressure and see the information the news how to how to AF you have AF what exercise you must do like this, very good.

I_01 : So you watched the videos, how do find the videos?

AF_0005: It…

I_01 : Any favorites?

AF_0005: Any favourite, AF.

I_01 : The AF one ok.

AF_0005 : I like that one because it’s my, my, my category. [Participant laughed]

I_01 : Ok, ok then other videos the other videos, cooking…

AF_0005: Cooking, no cooking!

I_01 : Actually there is one.

AF_0005 : Don’t have cooking…

I_01 : Got one cooking one actual I don’t know whe- I don’t have the device now but…

AF_0005: Like the third one I don’t know what is that I cannot open something wrong…

I_01 : Cannot open?

AF_0005: Cannot open then I called the this here nobody can, can…

I_01 : So…

AF_0005 : Give me the information then I called this, this, this number then got, got one don’t know nurse or what she picked up…

I_01 : Yup nurse correct.

AF_0005 : She picked up…

I_01 : What did she tell you?

AF_0005: She said I don’t I said I’m doing the survey then the, the tablet cannot on got problem then I called you all hey you know where is this place I said I don’t know, it’s a heart clinic…

I_01 : Correct it’s a heart clinic.

AF_0005 : But don’t you have this survey.

I_01 : Ok so actually you called is because you want to there is a problem with the device you want to try to fix it you thought the number is for that one…

AF_0005: Nobody can help I called many times.

I_01 : Ok, ok so, so…

AF_0005: Until that I, I’ve never see the what, what, what is that.

I_01 : Number ok so the, the number you did tried how many times you tried calling?

AF_0005 : Try many times got one time got one girl very helpful I don’t know what’s her name I never she is try to help me but nobody pick up.

I_01 : Ok so you did tried to call many times the number to trouble shoot the, the…

AF_0005: Then the 4^th^ one the 4^th^ one it can open only 4 only what only 4 only…

I_01 : I think the yah ok…

AF_0005 : Still have, I don’t know.

I_01 : 6 I think.

AF_0005 : Don’t have.

I_01 : Ok anyway the which one is your you said you, you like the, the information one…

AF_0005: AF!

I_01 : Then the…

AF_0005: The stroke…

I_01 : The ss- ok the stroke one how do you find like is it too the time is the duration how is it how is it?

AF_0005 : Ok.

I_01 : Ok then…

AF_0005: Better.

I_01 : Do you think you get it got help you a bit or not to change your behavior?

AF_0005 : Got, got, got.

I_01 : Got. How, how did it help you like…

AF_0005: Information then you know how it sometime you, you we see facebook also you see how to prevent a stroke what to eat then you what sign you should watched out for stroke it’s if it’s good what this, this one.

I_01 : It’s good ok so you like that?

AF_0005: I like that.

I_01 : Ok then the…

AF_0005 : The best part is…

I_01 : Yah what?

AF_0005: Every morning we have to monitor our blood pressure without that we lazy sometime don’t know I mean you got headache you know you go and measure ok very high like you’re the tablet very useful very high not, not very good if normal range well done, keep it up.

I_01 : So you like that.

AF_0005 : Encouragement…

I_01 : The feedback is you, you like that?

AF_0005: I like that.

I_01 : Ok then also the you said that last time you when on your own you don’t really do the monitoring?

AF_0005: Never, never, never.

I_01 : Why, why now you why now you then do everyday?

AF_0005 : Because I find it info what if you, you know sometime your blood pressure high sometimes it’s too low you will monitor if you don’t, don’t do everyday you don’t know then you can tell the sometime I keep the paper I write the everyday our my blood pressure. Sometime I give let my doctor see good it’s good if too high they, they said ok like maybe I increased the dose like that sometime you never monitor when where you got very headache you don’t know when yah or not how high is it you forget already.

I_01 : So the popped up the popped up messages what when, when it came up how do you feel how do you feel like…

AF_0005: If it, it said well done keep it up you feel very re- relieved so feel so happy yah you done well you monitor well you eat well then maybe it said you get enough rest sometime, any pressure any stress sometime are you happy today? [Participant and interviewer laughed]

I_01 : How do you find that?

AF_0005 : Ok interesting, interesting.

I_01 : Ok, ok do you do you find that it’s coming up a lot I mean the, the messages is it…

AF_0005: Yah sometime…

I_01 : The frequency is it ok?

AF_0005: Ok.

I_01 : Ok, ok then what about the survey you did the survey also right?

AF_0005 : Yah.

I_01 : How do you find the survey?

AF_0005: Ok it, it means you, you, you, you see they, they give you one ques- message to read like AF then they asked you question about AF then you answer the question correctly, well done you are up to it keep it up you feel very encouraging.

I_01 : The survey do you find that it’s too short too long o- just right?

AF_0005 : Too short I think too short.

I_01 : Too short ok survey too short?

AF_0005: I think too short.

I_01 : Ok so you prefer longer survey, what about like the, the is it too difficult?

AF_0005: No, no, no for me no don’t know with others I think too short…

I_01 : Too short!

AF_0005 : A bit more…

I_01 : The questions, the questions ok.

AF_0005: Better.

I_01 : You enjoyed doing it?

AF_0005 : Yah don’t have cooking, have cooking…

I_01 : Cooking, there’s one video on it.

AF_0005: I don’t have maybe I don’t have they collected the 4^th^ survey, the 4^th^ one they collected it because I went for a tour they come and collect the that lady I actually I will go tour on the 4^th^ of April she, she, she came don’t know 2^nd^ or what.

I_01 : Ok then the do you know the exercise one did you watch that…

AF_0005: Yah, yah, yah.

I_01 : So did you do the exercise when you watch it?

AF_0005 : Yah.

I_01 : You did?

AF_0005: Even in the polyclinic also have these exercise the same…

I_01 : Yah correct.

AF_0005 : The same lady also what the Indian lady right also very good sometime in the clinic cannot there’s many people stared at you cannot at home we can do.

I_01 : So you, you, you every time you watched you do?

AF_0005 : Do, do.

I_01 : Oh how often do you do it?

AF_0005: Not where everyday, everyday…

I_01 : Everyday you do?

AF_0005: Everyday sometime I do not but I never follow that one I follow mine one…

I_01 : You, you…

AF_0005 : The, the, the where you wake up is after waking up you stretched yourself exercise your leg like that then do all these. [Participant and interviewer laughed]

I_01 : Yah, yah so that one is your, your own one.

AF_0005: My own one.

I_01 : Your own one, ok.

AF_0005 : My own one then brush, brush here the, the temperate the here all, all the all the pressure…

I_01 : Yah all pressure yah.

AF_0005: It’s good I do mine one…

I_01 : Your own one!

AF_0005: Like that I do all mine one.

I_01 : Ok, ok is there a reason why you didn’t you didn’t follow what the video want…

AF_0005 : Sometime you forget…

I_01 : Forget?

AF_0005 : The, the sequence…

I_01 : Seq- yah.

AF_0005: The chair one I remember the other step that one the leg up then that one have other than that sometime near the wall there, forget.

I_01 : Forget, then if you forget why don’t you watch the video like the you know because you forget right you can watched the video?

AF_0005 : Yah the tablet take already how to follow?

I_01 : So that one is after, afterwards but during the time period…

AF_0005: Have, have, have…

I_01 : You…

AF_0005: During the tablet there have I followed.

I_01 : Ok, ok so you did followed it during that time when you have the tablet it’s only after that then you maybe forget the…

AF_0005 : Forget!

I_01 : What to do also.

AF_0005: I follow my own self one…

I_01 : Your own one.

AF_0005 : My own up down up down move… [Participant laughed]

I_01 : Ok, ok yah, yah correct it’s still it’s still good it’s still good to do it.

AF_0005: Move the leg sometime you see cramped you do all these.

I_01 : Ok, ok then the blood pressure monitor when you take quite regularly right you take normally in the morning, afternoon or…

AF_0005: Morning after, after breakfast.

I_01 : After breakfast I thought “M” told you before breakfast…

AF_0005 : Never.

I_01 : She mentioned, never so you…

AF_0005: Never doctor, doctor say one…

I_01 : So you took…

AF_0005 : After breakfast.

I_01 : After breakfast, doctor said?

AF_0005: Ah doctor said.

I_01 : Then any way do you have any feelings when you see the result or not?

AF_0005: No feelings…

I_01 : Feelings like how do you feel?

AF_0005 : Sometimes if you have headache you will feel the pressure high if you never sometime I never take tablet my high blood then I measure I take the teperat- the blood pressure, high!

I_01 : High ok.

AF_0005: One four something if I take already it go down to one two something I know must take high blood if stopped cannot stopped.

I_01 : Cannot stopped ok but you, you I mean do you feel like any stress when you take it or not…

AF_0005 : No.

I_01 : Anxiety?

AF_0005 : No I...

I_01 : You know worried, worried about the result you cannot think…

AF_0005: No, no…

I_01 : Don’t have ok, ok then…

AF_0005: But I have anxiety if got stress like…

I_01 : About other things?

AF_0005 : Problems at home with the you know the they sometime they quarrel and he tell me my daughter problem and mostly my daughter’s problem.

I_01 : Ok, ok so all those things actually added adds on a little bit to stress but, but when you take the tablet you are ok you feel ok?

AF_0005 : Yah.

I_01 : You feel calm?

AF_0005: No, no not really.

I_01 : Not really not really so got, got worry?

AF_0005 : Ah worried, sometimes I…

I_01 : But you still take.

AF_0005: Yah all these all these all my this one heart problems all these maybe caused by a lot of problem my own self my own problem last time my daughter also very sad, depression commit suicide…

I_01 : Really?

AF_0005: 2 times 2 times twice all these become my heart problem that time I was admitted in the hospital…

I_01 : That was before.

AF_0005 : But I cannot have problem you tell me I cannot sleep the whole nearly one week also cannot sleep cannot I cannot s- sometimes somebody if I called somebody she not at home I worried I scared don’t know what happened to them I’ve these type of problem I have these type of problem maybe you called anxiety.

I_01 : Anxiety ok you have that.

AF_0005: Have that.

I_01 : A bit of that so ok anyway the, the we want to know also about the what was your favourite like feature of the entire tablet this tablet and the app what was the favourite?

AF_0005 : Favourite? The picture…

I_01 : The picture…

AF_0005: The picture…

I_01 : Picture is the…

AF_0005: Some type is the there the AF the blood clot…

I_01 : The video is it the video?

AF_0005 : Yah.

I_01 : You like the video?

AF_0005: Video!

I_01 : Ok, then anything that you don’t like?

AF_0005 : No, everything nice it’s good.

I_01 : Ok then also right the num- ok you said that you, you wrote down your result and then you went to doctor right is it the, the result you, you take it from the tablet the is it?

AF_0005: No, no, no I have a paper the…

I_01 : Yah the…

AF_0005: Nurse give it to me everyday measure blood pressure.

I_01 : Yah so you, you, you bring to your doctor?

AF_0005 : Ah doctor.

I_01 : So the do you feel that it’s helpful or not?

AF_0005: Yah helpful it’s helpful.

I_01 : It is, ok.

AF_0005 : Is it if the doctor say…

I_01 : Why, why is that helpful?

AF_0005: Very good still normal ok you know doctor said no stress say ok yah doctor will ask you got any breathless or not any pain or not they will ask you these.

I_01 : So let’s say if we have if let’s say you can also bring the results for the, the one that you take for the tab- in the tablet one to bring to doctor you feel that it’s helpful?

AF_0005: Yah, yah.

I_01 : It’s a it’s a something that you already do what ok, ok, ok then…

AF_0005 : But one thing it’s very inconvenient you have bring…

I_01 : Ok is it too big?

AF_0005: Not big…

I_01 : And heavy?

AF_0005 : If said like you say bring the tablet you bring but the blood pressure no need the tablet…

I_01 : The tablet…

AF_0005: You see even I come to my daughter’s house I also bring everything here they don’t have the blood pressure the, heavy I from “C” walked until here there the “TP” car park…

I_01 : Yah the, the…

AF_0005 : Walked until I walked also…

I_01 : Panting!

AF_0005: Pant- age may be old age some more blood pressure a lot of stress it...

I_01 : So let’s say we have a few different device ok we have a tablet form we have a phone we have a computer desk top computer and also other electronic device which one will you prefer which device will you prefer?

AF_0005 : I prefer tablet.

I_01 : Prefer tablet ok, ok why, why will you say that?

AF_0005: Computer yah computer no need no need the electric as long you can charge well you see but tablet I think convenient.

I_01 : Convenient ok, ok what about phone, phone also convenient?

AF_0005 : Phone, phone also can…

I_01 : Also can.

AF_0005: But I never experience. [Participant laughed]

I_01 : Never experience never haven’t tried it yet…

AF_0005: Never tried.

I_01 : So don’t know, ok.

AF_0005 : I find first time I find tablet, last time they come not this survey last time they come they, they see if can lift up or not I also do all these survey the came to my place.

I_01 : Ok, ok then I wanna ask ok a bit about the, the tablet do you find the screen how do you find it?

AF_0005: Ok the screen ok.

I_01 : It’s ok, ok then the font the font size?

AF_0005 : Ok just nice what not so big not so just nice I find it nice.

I_01 : Ok, ok the brightness?

AF_0005: Brightness ok.

I_01 : It’s also ok then what about the, the you know navigating the app that means going in and out of the page?

AF_0005: Very easy, easy.

I_01 : Ok very easy ok, ok.

AF_0005 : Very easy not, not so a lot of steps just pressed here pressed there can already yah, any problem the, the “M” said you, you call these people nobody…

I_01 : Nobody the, the…

AF_0005: Nobody…

I_01 : So the, the call the calling…

AF_0005 : Some of them also don’t know they said don’t have [Interviewer laughed] this is a heart clinic I called so many time the nurse said do you know what is this place where is this place I think you called the wrong number, then I, I told this number I told this the doctor know…

I_01 : Yah, yah then what did she say?

AF_0005: The doctor not here.

I_01 : So actually the…

AF_0005: The girl don’t know very rude.

I_01 : Very rude ok, ok.

AF_0005 : Not here!

I_01 : Ok, ok I think I’ll write this down.

AF_0005: My daughter also wrote email to them what.

I_01 : Oh ok this…

AF_0005 : This…

I_01 : Yah very rude don’t know.

AF_0005 : Maybe they don’t know the survey maybe also find it so irritating I asked her [Participant laughed] maybe not her day on, on that day I cannot say.

I_01 : Actually they are also very busy.

AF_0005: I cannot say she’s rude.

I_01 : Yah I mean yah I’ll just put you, you did it’s, it’s ok one yah.

AF_0005: It’s ok.

I_01 : It’ll be hon- I mean you’ll be, be frank about with us you know and then we can feedback also, yah.

AF_0005 : They don’t know the survey I say how come you don’t know the survey I have this form are you calling, calling the right number, asked me.

I_01 : Ok so you, you just now I you mentioned that you write it down on paper already if we do this study in paper form let’s say we give you a paper to write down track your blood pressure then send to us back…

AF_0005: Can, from…

I_01 : Also can you don’t mind right?

AF_0005 : Don’t mind don’t mind no problem.

I_01 : But let’s say we got a paper form and the electronic form which one will you prefer?

AF_0005: Electronic…

I_01 : Electronic, ok, ok.

AF_0005: Faster yah or not if I have to send to you I don’t know what’s the outcome if they cannot give me the answer right away the, the tablet form or the what form phone form I can know the result straight away I don’t have to wait yah or not, direct.

I_01 : Ok so you prefer that one.

AF_0005 : I’m like…

I_01 : You, you also like the, the connection also you how do you find it?

AF_0005: Ok, ok.

I_01 : Connection is ok, ok.

AF_0005 : Very fast very fast blood pressure what, what “tut, tut” you know already so... [Participant and interviewer laughed]

I_01 : Ok so a few more questions let’s say right we introduce this device to you, you know when, when at the very first time when you had AF you were diagnosed with AF it’s been you had you had stroke then you found out right? You prefer us to introduce this kind of device at the beginning in the or in the middle or maybe later a bit, which, which time period?

AF_0005: Later I think, later…

I_01 : Later a bit why, why later a bit?

AF_0005: Because you don’t know anything about AF maybe can, cannot say later after you find out that time after…

I_01 : Aft- right after you, you found out.

AF_0005 : Diagnosed.

I_01 : Diagnosed, so that will be the best time.

AF_0005: Best time cannot say after.

I_01 : Ok, ok not right not immediately after but sometime after ok, ok.

AF_0005 : Then after that doctor said you had AF what is AF I don’t know then after the let the doctor the nurse explain then you know that is that is the most important time.

I_01 : Ok, ok so you why do you prefer that time like why do you think that’s the…

AF_0005: At least you know at least you know.

I_01 : At least you know ok, ok.

AF_0005: I also don’t know I have AF then everybody that’s why everybody the, the all the doctor new one I think young one all o- everybody come and monster like that ah, ah AF [Interviewer laughed] I also don’t know what don’t do so many come so many of them know…

I_01 : So many…

AF_0005 : Hospital “N”.

I_01 : The, the NU the heart clinic?

AF_0005: Heart clinic that time I A- I have the AF thing they gave me the tablet warfarin I take I think I take don’t know how many time I, I got bleeding then it stopped I was in the cancer clinic in the cancer clinic I said…

I_01 : How come you were there?

AF_0005 : Don’t know then they put me there then they, they asked me to go then they checked everything I scared I asked the doctor I have cancer, he say no I said this is a cancer clinic don’t worry don’t worry we, we want to monitor they do all sort of thing. They do the even the chest they checked full blast you know…

I_01 : Really?

AF_0005: 800 plus.

I_01 : The that one was the scan, the scan…

AF_0005: Then do the there got one tube it sucked don’t know what, what all the phlegm inside they checked a lot of test I don’t have cancer I don’t have tolme they scared I got TB I said I don’t have I said I not coughing blood I was like wake up in the morning got, got saliva I just spit out got blood.

I_01 : Oh, oh…

AF_0005 : Then they were they want to find out where the blood come from maybe you, you cough you lung or what the checked everything.

I_01 : Maybe they just want to make sure make sure…

AF_0005: They checked the lung already, they cannot find out then they sent another time asked me to full blast, that one very, very expensive.

I_01 : Yup the tests are quite expensive yah, yah.

AF_0005 : Yah spent money then even hospitalization insurance cannot claim.

I_01 : Ok, cannot?

AF_0005: Because it said I got heart, heart problem cannot.

I_01 : Ok so let’s say we introduce this app and this device to more patients it do you think that like people let’s say would help would they be willing to pay for something like that?

AF_0005: Pay?

I_01 : Other people, like who have the same condition like you know like AF right and maybe potentially other more…

AF_0005 : Yah some…

I_01 : Conditions also.

AF_0005: Some maybe not all said some maybe pay half…

I_01 : Half?

AF_0005 : Or maybe subsidized, maybe.

I_01 : Ok so that will be that will encourage more people to use, ok.

AF_0005 : Yah but some people find it inconvenient everyday have to do it’s a source then…

I_01 : And, and long period let’s say if you, you do this you have to monitor everyday for like more than one year are you are you be willing to do it?

AF_0005 : So long, half a year.

I_01 : Half a year, ok.

AF_0005 : That one too long.

I_01 : Ok so, so how about…

AF_0005 : Like sometime you go holiday how?

I_01 : Yah so holiday you can take a break ok you can you can paused for a while then after that you come back then you take again so everyday same, same thing as what you did but longer so you’re, you’re ok with it?

AF_0005 : Can, can but I worry sometime we go for so long we come back then everything completely out how, the information if cannot on how then like, like supposed I need help I called nobody can help…

I_01 : So that, that part you are worried…

AF_0005 : That I worried.

I_01 : The, the trouble shooting part ok, ok, ok.

AF_0005 : Sometime got problem like you see you want to on that day the third week I want to on then cannot on then got problem can you call this number nobody pick up, that’s the problem.

I_01 : And when they picked up it’s they, they don’t know also ok, ok.

AF_0005 : They don’t know also they don’t know there’s a survey going on they said heart clinic, I cannot be the wrong number I said the number is correct what.

I_01 : Because there are a lot of them also.

AF_0005 : I want to talk to this doctor he said doctor not in but he pro he’s busy.

I_01 : Ok so let’s say if you got any other any other things you want to see in this app on the device that you have used for this time period or not there’s some something else you want to see that, that but can improved it can enhance it something that you think that you want to…

AF_0005 : The heart, heart...

I_01 : That you that can help you?

AF_0005 : The heart, high blood, diabetes…

I_01 : So the other conditions you don’t mind having a app like that also, oh ok.

AF_0005 : Yup diabetic I’m diabetic also…

I_01 : Like diabetes ok.

AF_0005 : Everything I have then sometimes we take too much tablet don’t know maybe is it diabetic we have the leg cramp got problem, leg cramp then you connect a UTI all these I know information it’s useful.

I_01 : Useful ok, ok you don’t mind the app that will have more information about other conditions?

AF_0005 : Like last time I don’t know I always have UTI then the my, my clin- the doctor outside one then said better go and check whether you have diabetic are you diabetic I said no that time I don’t know I’m not yah really go and checked, really diabetic no wonder I alway got UTI that information I don’t know.

I_01 : Ok, ok any other things that you can maybe any other comments that you can give us to help us improved you know opinions about what we can do in the future for more patients which mean based on your experience you know…

AF_0005 : I see.

I_01 : We want we really want to hear from you…

AF_0005 : Me also…

I_01 : Because you are the user you know?

AF_0005 : Yah no, [Participant and interviewer laughed] I also don’t know.

I_01 : Don’t know?

AF_0005 : Yah don’t know maybe doctor, doctor can help doctor can give some speech maybe like the what dietician can talk about what food you take for more people to know like diabetic people will take what heart one like that. If you have you have taking blood thinning you shouldn’t take what, what food not only the like we have a booklet…

I_01 : Yah, yah so something like the, the diet…

AF_0005 : Diet…

I_01 : The things that you can, can and cannot eat based on your medication.

AF_0005 : Yah, yah that also useful what.

I_01 : Ok you, you find that useful if we add it in?

AF_0005 : Maybe if, if they in introduce those food to eat ok how to cook that also good.

I_01 : How to cook ok, ok.

AF_0005 : How to cook?

I_01 : Yah we had we had the cooking one but maybe…

AF_0005 : I, I don’t have I never see.

I_01 : Yah you didn’t yah you didn’t managed to see ok, ok, ok.

AF_0005 : That also good.

I_01 : Ok so you’ll like that.

AF_0005 : I like that.

I_01 : Ok like what, what about recipe?

AF_0005 : Recipe also can people like those like I like those caregiver like those who are sick caregiver how to like you can become a caregiver if suppose your somebody is sick how they take of them also that information also good.

I_01 : So you will like that also.

AF_0005 : Like that I mean not like my husband old already sometime what happened to him we can take care how to take care to bring him to the toilet bathe for him like that also good.

I_01 : But with if we air it add too many things in will you find that it will be too much?

AF_0005 : No, topic by topic it’s ok what.

I_01 : It’s ok, yah.

AF_0005 : Not, not all together one this week this topic next week another topic won’t what, don’t like rojak [ Means “a kind of mixed vegetables desert” in English] like that no like, like you doing you doing introduce that time that one also nice what I like that it’s very informative and...

I_01 : The video?

AF_0005 : Very, very nice but a bit short.

I_01 : Oh a bit short in, in what way you mean…

AF_0005 : The, the answer the question thing…

I_01 : The, the survey…

AF_0005 : The survey…

I_01 : The survey a bit short.

AF_0005 : Short!

I_01 : Ok, ok so ok so only the survey that part the video is it too the length is it…

AF_0005 : Ok, ok, ok.

I_01 : It’s ok, ok.

AF_0005 : Yah no wait if too long also boring if see, watch ok.

I_01 : Ok [Participant laughed] thank you very much ok so that’s about it for the, the questions let me stopped the recording.

End of interview
