# Supplementary material for: Patient perspectives of the Self-management and Educational Technology tool for Atrial Fibrillation (SETAF): A mixed-methods study in Singapore
Source: PLoS One. 2022 Jan 21;17(1):e0262033. doi: 10.1371/journal.pone.0262033 (PMC8782297; doi:10.1371/journal.pone.0262033)
Supplement: S1 Data — (ZIP) [file pone.0262033.s006.zip › AFP2_IDI_0004.doc]

Participant ID number : AF_IDI_0004

Interviewer Number : I_01

Length of interview : 36.56 mins

I_01 : Ok, ok so for the first question is for how long ago have you been diagnosed with AF or irregular heart beat you know the first time when you found out about it?

AF_0004 : It’s was it was December 2013.

I_01 : Ok so how did you find out about it?

AF_0004 : Oh I actually I, I had some valve problems which I I’m fully aware.

I_01 : What problems again?

AF_0004 : Valve, defective valve so I kind of have a bit of a fair bit of AF because my husband is, is a AF patient himself so we have been monitoring the situation over the past 10 years yah, so I myself I think this defective valve were due to some inborn or I can’t I didn’t know how it happened. So I, I already had some problem with my heart then I exercise regularly and one mo- one evening in December 2013, I was walking…

I_01 : Outside?

AF_0004 : Opposite yah on my own and usually I walk between 5 to 6 kilometres at night and that particular night I suddenly felt that I, I my breathing was bad and I, I, I, I felt breathless and I, I didn’t feel good at all so I cut short the walk and I came back and…

I_01 : Did you go and see the doctor?

AF_0004 : And then I every time when I try to walk I, I couldn’t climb hill at all then I, I didn’t see the doctor until time because I was already attached to hospital “A” at that time so they did a, a echocardiogram and then they said oh you have to put you on medication because you, you have AF now. And then they also started me with warfarin at the same time and, and subsequently they put me to, to a surgeon and we, we had agreed on, on the operation to correct the defective valve, yah.

I_01 : So that was how you found out at the beginning, ok so since then right how have you like taken care of your this you know AF like since that time period right from the very beginning when you found out?

AF_0004 : I, I’m very careful with my medication I, I took it regularly as well as I also with very, very careful with my diet so long as I feel, feel that I’m tired or I or I didn’t feel well I would not exert myself but I continued with my exercise so and then eventually in February I went for the ops.

I_01 : February this year or?

AF_0004 : No, no 2014.

I_01 : Oh ok that was the year after this...

AF_0004 : Correct yah so and then they at that time when they did the they did the replacement of the valve they also did another procedure I don’t know what is it called I can’t remembered already but basically they, they try to correct the AF. And of course it didn’t it didn’t go off immediately it took about one, one to 2 years…

I_01 : I see ok.

AF_0004 : Then I during those period I continued to take my medication until such time when I, I regularly see my doctor and then they test you see they go for the holter test as well as well as the echocardiogram and they found that we I I’m well no more AF and then they also stopped my medication.

I_01 : Ok so it was maintained very well based on the results that you got from…

AF_0004 : Correct.

I_01 : The, the tests that they did so actually the during this time period you, you take medication right you, you went to go back to the clinic to, to take the medication is it?

AF_0004 : Correct yah hospital “A”.

I_01 : Ok so how often do you to go back like?

AF_0004 : Every 3 months I have to go back because I need to test the blood for the warfarin…

I_01 : Correct, to adjust right?

AF_0004 : Just to make sure just to make sure that it’s at the right gauge then at the sometime they will give me the beta blocker for, for slowing down the heartbeat you see?

I_01 : Ok, ok so where did you get the let’s say when you’re taking care of your condition you know where do you get the generally the information from? Like the you know what to do what kind of medication to take and, and you know and also like the diet, exercise all these, these information where you get it from?

AF_0004 : Well when, when I saw the both the cardiologist and the and the surgeon they, they will be giving me advise on what you should do and what you shouldn’t do. And then usually when, when have I have any questions that relating to my, my, my problems I will make sure that I write it down then when I see them I will ask them, so in addition to that we, we used we google and try to...

I_01 : Oh google ok.

AF_0004 : Try to get information from the media you know?

I_01 : Ok media in what sense?

AF_0004 : Let’s say if I want to let’s say warfarin I want to know is this particular is this part particular type of food will hamper the, the, the function of the warfarin and then they will they will give you some gauge some ideas you see?

I_01 : Ok, ok that, that will be like TV is it?

AF_0004 : No, no…

I_01 : Or like newspapers or what sus…

AF_0004 : You go online you know…

I_01 : Online.

AF_0004 : Those you, you type in the so…

I_01 : So is there any particular website name that you, you are familiar or you go also or just generally?

AF_0004 : No, no gen- general yah.

I_01 : Ok then you just scrolled through the different websites?

AF_0004 : That’s right, also as I mentioned earlier my husband discover his AF since 2, 2007 so you are talking about some 11 years now so I, I monitor his case and make sure that he eats right and exercise right also so, so since so far it had been under control.

I_01 : Oh ok, ok so also same for your husband and that’s…

AF_0004 : Yes, yes but he is under a private doctor you see?

I_01 : Ok, ok that’s how you really got to be familiar with what to do and everything?

AF_0004 : Correct, correct yah, yah.

I_01 : Ok, ok so it also helps that you are that also you all also like you helped him also…

AF_0004 : Correct we support each other… [Interviewer laughed]

I_01 : Support each other.

AF_0004 : So we discussed and also we together we and we just move on you know, yah.

I_01 : Do you think it, it helps better than like say than going online or something in terms of getting you know like…

AF_0004 : Well going online is kind of…

I_01 : Additional.

AF_0004 : Additional just to get some support and information but we also try to discuss with friends who happened to have similar cases you know so usually they, they will they can exchanged opinion and ideas you now, yah.

I_01 : Did you find that you find that useful for you, helpful?

AF_0004 : It certainly it’s a good information but whether I eventually adopt the, the, the, the, the information or it’s another thing you see yah.

I_01 : Another thing ok, ok, ok so we go a little bit to your overall experience about the tablet ok so during these 6 weeks of the study so how did you use the device on a average typical day on a average day. So the, the in, in what sense we’re talking about is the sequence of the use so what did you do first?

AF_0004 : I, I…

I_01 : Or at what time?

AF_0004 : I usually will measure my, my blood pressure and to sync with the tablet so that they, they get a record then the, the, the questionnaire to, to be frank…

I_01 : I see, yah.

AF_0004 : Most of them I actually I’m aware of so I can answer it quite well but I find the video quite helpful also the video is very good and especially they recommend the exercise and which for old people like us would be useful to you don’t need to go out you can do it at home. Simple and yet it gives you a good workout of the overall body then diet wise I’m quite I’m quite familiar with that because I, [Participant and interviewer laughed] I will someone who can’t ss- can’t don’t like to be too overweight you see so I’m very careful with eating well. And then of course the, the, the, the information about how to prevent stroke and ex- explain to you when you have a AF what, what are likely to happen all that are very, very useful I mean you…

I_01 : You referring to the video is it?

AF_0004 : Yah the video itself .

I_01 : Ok which was your favorite video?

AF_0004 : I like the exercise most of course I also like to the prevent stroke all that also very good.

I_01 : Ok so which one not so useful or not so interesting for you?

AF_0004 : There’re all interesting.

I_01 : They all ok, ok, ok so you find them all quite interesting and, and helpful?

AF_0004 : Correct, to a certain degree they are helpful and interesting, yah.

I_01 : Ok do you think that if like we have more videos will be helpful or not like more, more…

AF_0004 : Certainly so certainly so but sometime need to be a bit don’t make it too long because otherwise people get fed up of looking at…

I_01 : And bored.

AF_0004 : Bored you know so you, you have to like for instance the exercise it’s quite lengthy it’s more or less like a full exercise so I don’t know how [Interviewer laughed] unless they follow it to do is one thing but if you just for information people may find it too, too lengthy you see. But the first time I looked at it I after watching for a while I felt a bit bored then I realized that why don’t I follow and exercise at the same time so I quickly put on my sport’s shoe and do with it so it was a good it workout a good sweat.

I_01 : Ok, ok so did you feel that it was you felt that the exercise was a bit long at the beginning but when you did it with together it was you didn’t feel long…

AF_0004 : You, you know what you should do you, you, you have to try to tell people quickly put on your, your sport’s shoe we do it together instead of because people when they…

I_01 : Watch?

AF_0004 : When they turned it on they thought that it’s for your information and they are introducing this but they didn’t realized that people are showing doing the full exercise you see so if you make it clear to them that then they may if they willing to follow then they won’t find it bored you see. Because it only about I think 10, 15 minutes altogether but when you sit and watched you may find that it is a little bit, yah.

I_01 : Ok so if we add it in an additional thing which is to give a kind of instruction to, to recommend to also do it together with the video it’ll be much more in-…

AF_0004 : Correct, correct yah you can modified a bit to explain to them that this will be good and shall we do it together just put on your sport’s shoe wherever you are you can still do it not necessary outdoor you can do it at home you know that kind of thing so it may help you see, yah.

I_01 : Ok then what about like the ok you mentioned like the popped up messages right so the popped up messages there were some encouraging messages and so the diet advise do you how do you find it when you when you when you saw the messages how did you feel?

AF_0004 : [Participant and interviewer laughed] Frankly speaking all most I would say almost 100 percent of the popped up messages are very encouraging yah, I don’t know is it because I have been a good girl in the sense that I dutifully I don’t missed any except 2 days I was in Malaysia. But I mentioned to your colleague that I, I won’t be able to do it she said fine…

I_01 : Yah it’s ok.

AF_0004 : So well it’s a good it’s a give a bit of a encouragement for the for the folks hope…

I_01 : You like it?

AF_0004 : Yah, yah of course when, when people give you praise is always good you see it makes it makes you feel good, yah.

I_01 : Ok, ok so a another feature will be the survey right we also sent some surveys that were that, that, that you got occasionally so how did you feel about the survey, your opinion about it like it was it do you think it was helpful the survey?

AF_0004 : To me not so because I knew exactly what is AF what, what it will trigger and what are the various method to, to, to, to try to elevate the condition so of course I dutifully I still answer the survey accordingly, they also asked a little bit about if you have the condition how would it affect? Frankly speaking you know when, when sometime when the survey come it so happen that day I was feeling depressed but not necessary to do with the, the, the AF just…

I_01 : Did… yah I know…

AF_0004 : For an example recently I just lost my younger brother who…

I_01 : I see.

AF_0004 : Who passed on very, very suddenly as a young man so that depressed the whole family and it kind of stay in our so those question like not necessary duty they asked you how do you feel how do you feel do you feel good? I was really very depressed but then if I said I’m depressed they may think that the AF may have triggered this you know which wasn’t the case you see so I’ve a bit of problem how should I answer you see?

I_01 : I see ok, ok.

AF_0004 : Because there are many factors and can affect your, your well being in a day you know…

I_01 : That’s right that’s right that’s right!

AF_0004 : But of course if it’s solely physical then it’s very easy but it’s not I mean in life is not so simple you know yah…

I_01 : Correct, correct there are other things that come into play.

AF_0004 : Some sometime even if there is a serious issue happened in the world it can also depressed you a bit yah or, or family matter or even I think environment also could sometime like if it’s too hot you can also feel a bit unwell you see, but on the whole…

I_01 : See…

AF_0004 : I, I, I mean that is my opinion about…

I_01 : Yah sure.

AF_0004 : The survey.

I_01 : We definitely want to hear you know how actually how you feel in terms of like you know whether this, this feature is useful for you or not so you felt that it didn’t really captured the accurately the sense that you had the, the, the feeling that you had even though…

AF_0004 : Correct, correct.

I_01 : It was a question about your feeling right ok, ok what about like do you find the it very diff- difficult or not easy or difficult the survey?

AF_0004 : : It is easy for me.

I_01 : Ok, ok easy ok were it too long was too long?

AF_0004 : No, no not at all.

I_01 : Ok then you also mentioned ok because you know you had all these other events that happened that, that affected you also do you feel that it give it made it give you stress and anxiety like this kind of surveys that asked you about your feelings at that point of time?

AF_0004 : No, no I think it’s not fair to blame it on the on the survey you know it’s just that at that moment I’m have a dilemma because if they on top they do say that with the AF does it make you feel how would you feel you know not too bad and…

I_01 : Sickening…

AF_0004 : Very depressed you know that kind of thing.

I_01 : The question was too also too general you feel is it?

AF_0004 : I, I would think so yah I would think so.

I_01 : Ok, ok can.

AF_0004 : If you if you specify very clearly that I don’t know I’m sure the expert have a way to, to do it, yah.

I_01 : Like I said we because you know this is like a pilot test so you know what, what you have said told us which is very, very useful yah and definitely helps because we are going to improve on it also ultimately.

AF_0004 : Of course of course of course yah that is the reason why I participate because I mean ev- everybody put in an effort…

I_01 : Correct.

AF_0004 : They can come out with…

I_01 : Definitely yah.

AF_0004 : Something even more user friendly as well as I mean of course you can never feed [Interviewer laughed] everybody but if people put in an effort put in your opinion so you will just have to…

I_01 : That’s right.

AF_0004 : Come to a…

I_01 : Kind of have like…

AF_0004 : Yah have…

I_01 : A kind of standard or a kind of average yah, ok then the next one is that ok there was this blood pressure monitor that you, you also used right so like when you when you strapped on and then you take your blood pressure right how do you feel about how do you feel in general? Was there any emotions that came about when you use tried used of it?

AF_0004 : Not at all because I myself I, I, I have one at home I used it I won’t said everyday because I more or less know where I stand you see so maybe once a week I do it but my husband I try to do it more often because his is more serious you see yah. So no let’s just go on…

I_01 : No it’s ok just, just feel free to mention… [Interviewer laughed]

AF_0004 : No I in fact I was hope hoping that if the survey was extended to my husband it will be fantastic you see because he will be in a much better position to do it it’s, it’s so unfortunately that he’s not tie to hospital A so he wasn’t given this opportunity yah.

I_01 : That’s right yah.

AF_0004 : Because for me since I’m, I’m already off medication I have no AF so it I can only tell people based on my experience yah.

I_01 : But it’s still relevant it’s still relevant…

AF_0004 : Of course of course yah.

I_01 : Because again you had it before and also now you are trying you also managing it now right yah, yah?

AF_0004 : Of course even the doctor my g- my cardiologist told me that don’t rule out that it will recur again you know in fact from there I was experienced it happened to a fair bit a number of patient you see. So I, I don’t I’m not entirely free of it you see in that sense you see?

I_01 : You still have to continue to watch and maintain?

AF_0004 : Of course, of course.

I_01 : Yah ok then the next one is that ok so were there any aspects of the app and tablet right were that you felt that was easy overall you know or if you considered everything right you know the, the videos, apps, messages, the blood pressure monitor right? Was there which one you felt was easy to use of all these different features?

AF_0004 : I think overall the most all of them are very easy to…

I_01 : Oh all of them, ok…

AF_0004 : All of them are very easy to manage because when your colleague deliver of course she explained to me but still you have to go in you know to try on it so the I was a bit apprehend in the beginning I were but when I first started using it. Then I realized that it’s, it’s very, very straight forward and very easy to use.

I_01 : Ok, ok so we will ok you mentioned the colleague the “A” came is it so I just want to ask a little bit because we also want to find out more about how they conveyed the message to you right? So that one is how do you feel about like you know they con- when they conveyed the message to you was it very long, was it too long?

AF_0004 : No, no not at all she didn’t spend much time here, yah.

I_01 : Ok, ok was it very how did she take about I mean like estimate like estimate?

AF_0004 : 10 minutes.

I_01 : 10 minutes ok, ok that’s not very long ok so was it easy or difficult you felt you know?

AF_0004 : As I mentioned earlier [Interviewer laughed] when I first received I although I’m very familiar with this…

I_01 : Correct, yah.

AF_0004 : The tablet itself is, is I’m a little bit [Interviewer laughed] I won’t say nervous you know I, I was wondering whether it would be easy to handle or not but then once I started using it wasn’t at all difficult I find that I can managed it very, very easily.

I_01 : And that was when she was also when she conveyed the message to explained the instructions that was also helpful?

AF_0004 : Yah, yah she explained to me the various features what I need to do the all the all the pox I mean all the…

I_01 : The…

AF_0004 : On the menu what, what box is for what then that kind of thing so overall it’s, it’s, it’s ok it’s fine, yah.

I_01 : It’s ok, ok, ok then so that will be that do you are you aware that also like you know in the device there was also like a, a kind of number that you can call to contact a medical team?

AF_0004 : I in fact I tried to call once but it rang…

I_01 : Oh ok, ok did anybody answer?

AF_0004 : And rang nobody answer…

I_01 : Oh ok nobody answered!

AF_0004 : I called 2, 3 times that was…

I_01 : Oh you called 2, 3 two three times ok.

AF_0004 : On the same day because…

I_01 : Same day ok.

AF_0004 : I, I, I was going on holiday in Malaysia so I thought I need to bring this in to continue the, the, the measurement but then I realized that this works on wifi so how do I…

I_01 : Correct.

AF_0004 : I need to log in the I mean connect the wifi in, in the hotel so I was hoping to get through to somebody and nobody answer…

I_01 : Nobody answered ok, ok.

AF_0004 : So eventually I contacted “colleague A” then she told me to don’t border we just leave it in Singapore.

I_01 : Ok but you were aware that there was a number?

AF_0004 : Yes, yes yah the number is stated on the tablet.

I_01 : Ok, ok so you ok do you, you didn’t get through that’s the thing…

AF_0004 : It rang and then nobody answer.

I_01 : Ok I see ok so one thing is that subsequently when you were using the tablet throughout this time period from the beginning until now did you like used it to see the doctor or not like for example there’s they, they have the results there right that you can track right? So did you use to like you know let’s say you did you go back to the clinic or you go to another doctor to and then used the results to help you?

AF_0004 : Because since I last saw the doctor my next appointment is sometime in May and August I have 2 appointments but so it’s not within that visiting time you see, consultation so I, I didn’t in but I believe overall the graph is quite stabilized. So there’s no issue and I don’t see the need to bring it up to my cardiologist that means.

I_01 : Ok, ok but you, you didn’t see any other doctor ?

AF_0004 : No, no.

I_01 : Ok, ok, ok that’s all we want to find out if it helps you in you know subsequent visits…

AF_0004 : No.

I_01 : Ok, ok the other part is how do you feel about the interface because the part about the overall tablet right do you know the how do you find out the size the screen is it too small or too big how did you find it?

AF_0004 : I think it’s just nice.

I_01 : Just nice, ok!

AF_0004 : Yah just nice you, you don’t need to have a very big screen you see it’s quite easy to handle yah it doesn’t take up too much space as well yah.

I_01 : Ok then what about like the font is it too small too big the font you know sometime very hard to read right you know or not? [Interviewer and participant laughed]

AF_0004 : No the font is [Interviewer and participant laughed] is in good size even I with such bad eyesight I find it very easy…

I_01 : Really, no I think you are ok. [Interviewer laughed]

AF_0004 : Bad my eyesight is really very bad yah.

I_01 : Really yah ok what about like colour is it ok or not?

AF_0004 : No problem no problem colour no problem yah video is very clear.

I_01 : Ok clear! Ok then ok just we just move on to the overall acceptability so how do you find overall about the duration of the study like do you find it was ok it was too long too short you know how do you find it overall?

AF_0004 : It may be stretched a little bit too long I think a month should be good enough they need…

I_01 : Ok a month ok.

AF_0004 : Yah that’s my opinion but I may be wrong because, because people with AF the fluctuation may be worst so they may need a longer time to, to get correct data from these people you see to find out how they fair in…

I_01 : But for your own you felt it was a bit long?

AF_0004 : Yah, yah.

I_01 : Ok, ok, ok then the other one is that if you are giving this device to help with your life like you know in terms of your health and you know in just average on a normal day maintaining it and watching it right? How much time would you be willing to spend on it I mean if let’s say it’s not a study it’s like your own ok you do it you, you used it for your own main maintaining your own condition watching it so how long will you think you will willing to spend on this kind of device? Let’s say let’s say you have it?

AF_0004 : I think probably about less than half, half an hour will be good enough.

I_01 : Ok that will be like everyday?

AF_0004 : On a daily basis, yah.

I_01 : Ok but what about like in terms of like, like will you be willing how long will you, you be willing to hold on to something like that you know that? If let’s say a doctor you go and see a doctor right the doctor gave it gave it to you, you know just for maintenance of your and, and to watch your condition so how long would it like would you be willing to take? It’s not a study it’s just a average normal visit so like how long will you be willing to just hold on to it?

AF_0004 : That’s a diff- difficult question to answer [Participant laughed] because a lot depends on the condition you see if it’s very stabilized then…

I_01 : Yah so in your situation right you know when sta- it’s already very stabilize so like your doc- say one day you go and see your doctor you know just for get your medication for example. So like how long maybe will you be willing to hold on to a device like that to watch it you know over a period of time it could be like indefinite it could be like you know as long as possible also?

AF_0004 : I, I, I would think in my case probably a year?

I_01 : Ok a year ok.

AF_0004 : Yah hold on to it for a year and because I, I I’m quite concern with my own condition because whether in 6 months or in a year’s time it may it may happen again you see so to, to have the device it probably will help me to detect it and then I can discuss with the doctor about it you see?

I_01 : Ok, ok then next one will be like ok would you prefer to use it as in like ok this, this a tablet form right so are there any other forms that you feel will be more convenient for you? Say like it could be like a phone it could be like a watch it could be a computer you know which other ways do you think that would be you know useful for you in the form in the form of a device that you have?

AF_0004 : Well instead of a, a…

I_01 : Alternative forms for example?

AF_0004 : Yah I, I think if maybe I this is only for my personal view…

I_01 : No we, we definitely want to hear from you very important.

AF_0004 : Because I, I used a, a laptop so if the web can be I mean the app can be downloaded to my laptop so because I turned on my laptop on a daily basis you see for other purposes so at the same time I can also go into the app and do whatever I need to do you see. So it may be more convenient and also it will prompt the user to use it more readily instead of having a separate device you see so but of course…

I_01 : So…

AF_0004 : A lot of people don’t, don’t operate a, a, a desktop or a laptop you see so it may it, it, it all depends yah…

I_01 : But in your case…

AF_0004 : But in, in my case I think if it can be connected to a, a desktop a laptop…

I_01 : A laptop ok, ok.

AF_0004 : Is good enough yah I can also do it ev- everywhere I want you see.

I_01 : Ok, ok so, so you don’t mind that ok, ok then let’s say if it’s the, the do all these content you know like it’s delivered like the survey everything right it’s delivered like in paper form would you be ok with it compared to like electronic form right so would you prefer electronic or paper?

AF_0004 : Oh definitely electronic yah because…

I_01 : Any reason?

AF_0004 : It’s so easy to answer and then and then submit if you do it on paper it quite it’s, it’s more work you know got to print it out and waste, waste paper you see so why not if, if, if but then some of the some of the older people may not may not find it so convenient to use a computer. Then it may be a different scenario…

I_01 : But for yourself?

AF_0004 : I prefer it online.

I_01 : Electronic…

AF_0004 : Electronic yah.

I_01 : Ok, ok so you’re alright with that comfortable a bit.

AF_0004 : Very comfortable.

I_01 : Ok, ok, ok then the, the, the last few questions ok which is the let’s say the device was introduced to you when you were first diagnosed with the AF right and from the beginning do you think it will be helpful to you? Or do you think that like when will be the best time to in- to introduce it to you? Is it the beginning the middle when you are taking your medication or maybe at the current mo- at the current time like would there be a particular time which you feel will be suitable or anytime?

AF_0004 : Of course I think at, at the onset of it when you first get it because you are new to the problem this if this can be introduced it will help a lot more and, and the person will get better information and also to, to be able to follow it closer in the middle of it I am not too sure. But in my case I, I think at the beginning should be good.

I_01 : At the beginning!

AF_0004 : If you can catch the patient in, in good time, yah.

I_01 : That will be much better than, than maybe like at the current time…

AF_0004 : Right.

I_01 : It’s more stable?

AF_0004 : Yah correct.

I_01 : Ok, ok so like ok in do you have any like further like say suggestions about how we can improved this tablet app like, like any other features that you feel we can add on to it, it can be anything you know…

AF_0004 : I can’t think of anything right now.

I_01 : It can be the wildest you know can be the most [Participant laughed] can be anything that you can think of you know yah?

AF_0004 : Can I think about it and then tell you later?

I_01 : Yah sure I mean you can take, take your time to think about it about it.

AF_0004 : No not now I mean.

I_01 : Oh not now, ok.

AF_0004 : I can…

I_01 : You can write you can write give me like write to me maybe like…

AF_0004 : Yah, yah I give you I you…

I_01 : I have you have…

AF_0004 : You did you write it on called me on, on your…

I_01 : I called you yah…

AF_0004 : Mobile?

I_01 : I did I did yah.

AF_0004 : Ok let me think about it then I, I text you, you have whats app right?

I_01 : Ok, ok good I have what’s app so you maybe you think of like you know…

AF_0004 : Ok I can send it to you.

I_01 : Features that like say could be could be anything could be like say a tracker a like exercise tracker it could be like a you know like some other monitoring like your hea- your could be like your blood pressure all that. Oh this is blood pressure already but other, other ways of monitoring your blood sugar level even you know anything you can think of yah so…

AF_0004 : Ok there’s something that I noticed that the emphasis is only on blood pressure…

I_01 : That’s right.

AF_0004 : Why is it that they are not…

I_01 : Good question, yah.

AF_0004 : They are not into, in into the heart rate?

I_01 : Yah ok if the I think the, the purpose right for this set they want to start very small first so they, they start with one particular it could be actually the heart rate is a possibility also but given that blood pressure most people are more really like more familiar to use using it before right?

AF_0004 : Right.

I_01 : So we decided that something very simple and also something more familiar we start with one feature first we introduced in and see if people if it’s comfortable then, then we can let’s say if you know the feedback when we get we can build upon that, yah start with something very simple first.

AF_0004 : Because if I understand correctly AF will influenced the heart rate very much you know…

I_01 : That’s right that’s right.

AF_0004 : It can go very high it can go very low so that’s how you get blood pulled and clogged and…

I_01 : That’s right.

AF_0004 : And get this small little thing that travel round your body so I, I personally I’m very particular about my heart rate also…

I_01 : Oh ok.

AF_0004 : So I was thinking…

I_01 : Heart rate ok, ok!

AF_0004 : If, if it can be incorporated into the study at the same time it will be useful for the patient…

I_01 : And if it’s a…

AF_0004 : As well as the, the people who are developing this you see?

I_01 : Ok and if it’s a additional feature also to measure the heart rate it will be useful like a, a additional feature like blood pressure and heart rate will it be useful?

AF_0004 : Well ideally you [Interviewer laughed] you if you can meet if can captured it in the same device then you don’t have to do too many things you see you know old people usually they are not into don’t, don’t like too many…

I_01 : Devices?

AF_0004 : Devices you see.

I_01 : So you feel that less device is better?

AF_0004 : Of course of course the, the least the least is the captured the most information is the best, [Participant laughed] but often that it cannot it may not be possible yah, see I’m doing a track tracking…

I_01 : You also have a tracker yah, yah.

AF_0004 : But this is under the Health Promotion Board one yah.

I_01 : Ok do you find it useful, the tracker?

AF_0004 : Yes, yes, yes.

I_01 : You found it, ok…

AF_0004 : It’s very useful because it does prompt you to, to, to be more active you see so me and my husband we walked almost daily try to hit the 10 thousand step which is our minimum target you see.

I_01 : Oh ok, ok so you all do follow the guidelines also I mean the recommendations ok.

AF_0004 : Correct, correct yah.

I_01 : So is this did this tablet also like kind of encourage you to exercise more you feel in terms of…

AF_0004 : No…

I_01 : You don’t feel that?

AF_0004 : No, not at all.

I_01 : Except the video?

AF_0004 : The video are [Interviewer laughed] I mean the I, I, I you know why because as, as we get ag- aging we, we do get like a bit of aches here and there and you find that your, your overall fitness is not there. So the video will help to I mean I don’t have to go out I can indoor stayed at home and still able to move different part of the body in fact I think if they can develop more exercise to introduce to the older folk which, which is very good, yah.

I_01 : Ok so exercise videos but also like something that can encourage the exercise…

AF_0004 : Yah correct, yah.

I_01 : In the as a part of the additional feature?

AF_0004 : Yah correct, yah.

I_01 : Ok, ok that’s very helpful actually I think so finally right maybe we just want to ask like is there like anything any thoughts any comments any opinion about say your experience so far about this device this tablet?

AF_0004 : I ad- ad- admire the, the, the effort put in by, by the team of people who come out with this although I mean there are of course plenty of room for improvement it is a good start. And then I’ve I hope eventually you all will make it a success and more and more people will benefit it and use it that that’s all I can appreciate about it, yah.

I_01 : Ok, ok thank you very much for for…

AF_0004 : Right, right.

I_01 : For you know these feedback it’s very helpful, ok I stopped the recording ok.

AF_0004 : Yah thank you.

End of interview
