# Supplementary material for: Patient perspectives of the Self-management and Educational Technology tool for Atrial Fibrillation (SETAF): A mixed-methods study in Singapore
Source: PLoS One. 2022 Jan 21;17(1):e0262033. doi: 10.1371/journal.pone.0262033 (PMC8782297; doi:10.1371/journal.pone.0262033)
Supplement: S1 Data — (ZIP) [file pone.0262033.s006.zip › AFP2_IDI_0003.doc]

Participant ID number : AF_IDI_0003

Interviewer Number : I_01

Length of interview : 45.03 mins

I_01 : So thank you very much for taking part in this interview the so the really first question we want to ask is to that how long have you been diagnosed with AF and you know when did you first find out about it?

AF_0003 : [Participant laughed] I don’t know the exact date probably a few years ago ok maybe as much and 3, 4 years ago ok but I, I, I’m, I’m lost track of actual time probably around 3, 4 years ago ok?

I_01 : Ok around that time and then how do you find out during that, that time?

AF_0003 : One afternoon I have suddenly h- very high blood pressure 196 it shouldn’t be you know I don’t have that sort of high pressure around 1… 130, 140 so and the trouble is it’s not one off it persist for half a day. Despite me going to botanic garden walking to calm myself down and so on it still persist so I went into the emergency at hospital “A” that’s when they told me it should be having this the problem go and see the your Mis- doctor “A” is it right? That’s how I that’s what I was told when doctor “A” when I went to see him on the spot I have this irregular heart beat, on the spot while I was seeing him hey, hey, hey you know he checked hey yah you so confirmed that’s why I that’s why confirmed on the spot I have AF, that’s all.

I_01 : Ok so did you get I mean was there any medication was recommended to you, you know by the doctor and by the nurse?

AF_0003 : I first my first check up because although I was in emergency and then appointment come in later I do went to the place “B” the following day so they gave me these blood pressure pill and, and aspirin you see right? So when doctor “A” saw me he replaced it with something more effective yah so and it has worked alright and then he has fine tuned it because initially it may be that high dosage and he has fine tuned it and now it’s better. Because in between there’s some hiccup…

I_01 : I see ok.

AF_0003 : Right it’s too strong right.

I_01 : That one it’s for the blood pressure?

AF_0003 : Yes.

I_01 : Too strong it’s for the blood pressure mainly?

AF_0003 : Yah, yah ,yah while in, in essence I get giddy spells right and I collapsed twice so once, once I collapsed the next day I went see Place “B” say you better go back see heart doctor meanwhile take away your blood pressure pill. So I took away the real pressure pill and confirmed I don’t need blood pressure pill anymore right so and, and later on the dosage of the other things are also fine tuned you see the blocker all that is fine tuned right. Not the blood thinner the blocker was fine tuned to half and that is fine now.

I_01 : Ok, ok so on top of that right also like do you have like, like diet and exercise advise given to you by the you know…

AF_0003 : I did myself I, I the moment I get this irregular heart beat this is a wake up call for me in the past I’m very carefree because I’m…

I_01 : Yah, yah I know.

AF_0003 : I’m, I’m born…

I_01 : I know yah.

AF_0003 : Damn rugged therefore I’m healthy all the years so then, then I’ve been I’ve been eating well drinking well and so on then I realized hey I put on a lot of weight so then that, that with that wake up call I decided to put myself on a vigorous exercise regime and very careful on my diet. So over process in the space of a few a few years I’ve lost most of my fat about 30 over pounds of fat I can even said 40 pounds because losing that much fat I replaced…

I_01 : After all, yah.

AF_0003 : I replaced them with 10 pounds of muscles right I have now muscles you see right my legs and hand have muscles now because I do cross training so I have been doing cross training for the last 2 years and before that my sort of brisk walking for another 2 years so total I’ve done I think 4 years of exercise. 2 years of cross training must including weights, weights you know…

I_01 : Wow it’s very it’s very good yah.

AF_0003 : Everyday!

I_01 : Yah very nice.

AF_0003 : Everyday I spent one and a half to 2 hours everyday in down the gym so half an half an hour of treadmill and one and a half hour of weights you know so that my upper body is all strong with the set new sets of sort of weight yah. And then my legs are with the clock treadmill for half an hour so that keeps me goes up and down the whole body now is very fit that’s why. And then my diet I was reading professors of medicine in USA about dieting you see so I’m very carefully careful of changing my diet to suit the healthy pattern…

I_01 : I see.

AF_0003 : So day time I’m diet daytime I’m actually a vegetarian and night time I eat some steamed chicken or, or usually kampong [means “village” in English] chicken or fish, wild fish right…

I_01 : Healthily cooked.

AF_0003 : Yah, yah these so these are the only meat I take you know fish and kampong [means “village” in English] chicken only right alternate days but the, the, the rest of the day I’m vegetarian because I have nuts I’ve sweet potatoes I’ve milk I, I’ve maybe some oats crackers and big plate of green leafy vegetables. And night time maybe raw capsicum you know different colours and so I’ve maintain this very healthy diet, you know yah.

I_01 : I see then all these…

AF_0003 : All these diet, wine which I’ve been drinking for the last 25 years just red wine you know alright red wine ok.

I_01 : So other than you know getting the information from the you say you, you read up…

AF_0003 : Oh yah quite a lot.

I_01 : Is it from a book or like a website…

AF_0003 : No, no medical books.

I_01 : Oh medical books ok, ok.

AF_0003 : One is a University “A” by the, the medical school it recommend for people about 40 years old I’ve been reading it for the last 20 years so I’ve been I’ve but I haven’t vigorously followed it the last few years I followed it I work the book was there I’m aware but I didn’t practiced it you see. But last 4, 5 years I practice it you see and additional I go for the U.S. professors who actually aging you know how to manage your aging?

I_01 : Is that like a video or…

AF_0003 : No, no, no book, book!

I_01 : So all book so mostly from books?

AF_0003 : Yeah, yeah, yeah I read.

I_01 : Ok any other sources like you know you watched like video all these like…

AF_0003 : No I don’t.

I_01 : Online you know yah?

AF_0003 : But I picked up whatever medical news on the newspaper…

I_01 : Newspaper ok!

AF_0003 : You know occasionally they have they give you some advice from newspaper “A” you know on certain health issue and I normally cut it out and, and keep it so that you know…

I_01 : Remember…

AF_0003 : Yah so that occasion I, I read it you see then once it sink in I throw it away once I already internalized I throw it away so that’s helpful not necessary just a heart condition but other health you see…

I_01 : Yah in general.

AF_0003 : Usually it is yah other health issue cancer or what and so on so I picked up so that, that, that helps me supplement me from these 2 book which are quite comprehensive because these are medical books ok.

I_01 : So you take your time to just go through them you know…

AF_0003 : Oh I think no just go through [Interviewer laughed] I know them cold.

I_01 : Oh I see.

AF_0003 : Cold and thus if, if you give me a test I’ll get “A” [Participant laughed] you know on all those stuffs there alright.

I_01 : Ok, ok so you really learn the thing there?

AF_0003 : Yah, yah I, I, I as I said I learnt it cold you know so I passed the exam I’ll get “A” you know if you put questions there…

I_01 : So you…

AF_0003 : I can I can teach people there you know…

I_01 : Right.

AF_0003 : Its already all internalized.

I_01 : Inter- ok, ok so we want to ask a little bit about your experience in this study during these 6 weeks so how do you use the device on a average day a typical day?

AF_0003 : Oh I was advised, advised by that lady equipment picked it a time when you are calmed so in other words I’ve taken my medication one hour for that my food basically one hour one hour after those things when I’m calmed then go and register. So I do not do it straight after my gym exercise or after a bath a shower or immediately after food so normally is a period when I’m really relax and calmed. So normally I watched news on the TV watched I don’t watched other any except news you know so I watched the few news channel BBC the Chinese news channel and so on after watching the news actually I’m very calmed.

I_01 : Usually that is morning, afternoon, noon or, or evening?

AF_0003 : I, I taped it taped it so that I watched it at my leisure time.

I_01 : In your leisure…

AF_0003 : So normally it’s around maybe 11 to one around that time.

I_01 : About noon like that?

AF_0003 : Yah while I’m having my green vegetable that’s when I will watched.

I_01 : Take it.

AF_0003 : Yah so…

I_01 : And that’s when you also take the that’s the best time for you?

AF_0003 : Yah because I’ve, I’ve taken the medicine already early, early in the morning after my breakfast and I’ve taken my green vegetable and after watching the news one hour later I’m calmed I’m totally calmed you see I go in so the readings are always very good readings because I’m not stressed up ok. That’s when I always take my readings.

I_01 : Ok, ok so how were there any like overall features that you felt was like ease aspects were easy for you or more difficult which one will you feel was easy which one give you worst…

AF_0003 : Inside that one.

I_01 : Like overall yah…

AF_0003 : From the program there no, no it’s nothing it’s just no, no brain [Interviewer laughed] no brainer no, no big deal it’s very simple…

I_01 : Ok, ok so you find it all…

AF_0003 : It’s too routine you see.

I_01 : Oh too routine.

AF_0003 : Yah too routine.

I_01 What in what way is it routine?

AF_0003 : The survey is boring in fact.

I_01 : The survey!

AF_0003 : The survey is boring it’s the same question you see so it’s, it’s you know so after a while I don’t border any more you see.

I_01 : Ok, ok what other aspects like you found a bit like too routine?

AF_0003 : Except for that the rest helpful the reading is captured on a 20 what whole period right I can see the 20 day average and so on that’s helpful I having a quant background early in my years numbers I can read and understand numbers easily right. I just don’t read the number but from there I can sort of a…

I_01 : Gauge…

AF_0003 : Make instances of those numbers right so you know I can draw conclusion from numbers that’s helpful that chart is helpful because I can read charts and numbers easily that’s helpful right and your info provided for example what is what is this illness is…

I_01 : The AF.

AF_0003 : Showed the heart and should observed that is very helpful to me.

I_01 : Is it the video?

AF_0003 : Yah, yah video.

I_01 : So you…

AF_0003 : Video!

I_01 : So you found that…

AF_0003 : I found the video helpful.

I_01 : Ok, ok so which one do you like the best the for the videos like?

AF_0003 : A few of them like that one showing the heart inside there and so on what constitute a blood clogged all including the brain and so on and how they get the stroke what is actually this AF you know they showed the picture…

I_01 : That’s right.

AF_0003 : And I think…

I_01 : The, the cooking one the exercise one the…

AF_0003 : No that, that is not, not important because…

I_01 : Not, not really ok, ok.

AF_0003 : I, I know more than that you see like I say essentially medical information…

I_01 : Medical ok…

AF_0003 : Is helpful for me medical information…

I_01 : So mainly that?

AF_0003 : The cooking and exercise I, I know more than them so those are you know, yah.

I_01 : Ok so what about the other features like for example the ok the how do you find you said the survey was a bit ro- you know repet-…

AF_0003 : You see the same question that they asked.

I_01 : Same questions so it’s like repetitive.

AF_0003 : Yah in fact that’s why it’s quite routine I, I, I, I think they could have asked gather more information from me in the survey you know when asked went into a survey you want to get some extra information for the client you see. But if you asked the same quest- question everyday you are not getting any more you see, right?

I_01 : Ok, ok so you won’t mind something a bit more and like because…

AF_0003 : I think it not me mind I’ll be surprised the researcher having a, a person here do not want to clean more information to help your research you see. So I’m sure if I’m the researcher I’ll put in something which is something which I really want to know a extra I put it in whether I collect it well response is 50 percent, 100 percent is, is, is never mind. I see if I get 50 percent then I can still give me some understanding you know alright right or I, I would used you did not research end neck total evidence you know right even if I get 25 percent response you see you know for, for those difficult question that the respondent may not be answer you see right? So with IQ not high enough why not you see right but those who response I get info you see that help a researcher because you’re getting accessed and you don’t used it you see. Now why, why you want to… [Participant laughed]

I_01 : Because the idea is to also you know because we assumed that ok we start simple first and then we see…

AF_0003 : I understand I understand because…

I_01 : The experience like…

AF_0003 : That will be suitable for the whole range of IQ low IQ also…

I_01 : Yah, yah I know I know yah.

AF_0003 : Can do it you see but I would I would think if you would like to get more information on research that’s where you can get it you see right rather than more costly to come in to talk to me you know right you, you can’t come often you see right because there, there are so many clients out there you see right? But there’s a or you can even have tailored made you know for the more regarded phase for example are you a university graduate or talk to a person even not you see but he’s form 5 but he’s already matured in the what the university hard knocks you see. Then he’s intelligent enough you can asked more questions you see so that it need not be standardized those routine question so different clients you have set A, set B, set C you know then it help your researcher why not you know set C hey this guy can help me in my research then I get more information you see feedback to you, you see right? He happens to be a client AF client but also he’s highly intelligent then he can helped you in research but he he’s a AF client but he’s a low iQ then you give him routine question because you not, not getting much you know nothing much you see but you helped him more. But the other guy he can help you, you see why not you see so since you accessed him already and he’s willing right if he’s not unwilling that is different he’s willing what’s the big deal you see right, ok that’s all.

I_01 : Ok so like what about like the, the popped up there are some popped up messages like you know diet advice encouraging messages do you found that helpful on some of the…

AF_0003 : Not personally for me I keep telling you because I…

I_01 : Because it’s a bit different from the survey right?

AF_0003 : I know but say my, my diet my exercise is more vigorous than them [Participant and interviewer laughed] you know so those are I would say too elementary for me…

I_01 : Too ele- ok, ok.

AF_0003 : Right so yes it’s I think for the for those other guys maybe they don’t exercise…

I_01 : So you don’t really have…

AF_0003 : Mine is already like…

I_01 : [Interviewer laughed] I know.

AF_0003 : Training like athlete you see right what 2 hours one and a half to 2 hours every morning.

I_01 : Much more comprehensive and, and, and vigorous right? [Interviewer laughed]

AF_0003 : Oh yah, yah, yah so these like bring my blood pressure from 1, 140 and about maybe 90 to now one, one something you know one, one something to below about 60 something 70 you know so it brings my pres- pressure to down compared to and I can brisk walk and eat well sleep well you see. So it’s I’ve optimized basically right so I don’t think I need much help in these 2 areas on diet or on exercise but medical information is always welcome always welcome any new things on AF coming in which I should know even advanced information I’m able to understand ok I got science background right?

I_01 : Ok so you, you actually prefer more of it?

AF_0003 : Yes!

I_01 : A lot more.

AF_0003 : More medical information more even advanced information about AF so that with that I can help myself you see I don’t need to wait for the doctor to tell me I’m a person with science background who can used that information to start a program for myself without waiting you know, right? Like my diet you know my exercise and so on.

I_01 : And you are more than happy to, to do it like that way?

AF_0003 : Oh yah, yah really it is my health if I don’t take care of myself who is going to take you see right, yah.

I_01 : Yah that’s right ok so then how were there any other emotions that you have when you, you know approached to doing this you know. Other than feeling calmed right you know when you when you try to take your blood pressure and then, then maybe watched the videos were there any other emotions that come, come to you or not like…

AF_0003 : No I, I, I just I said it’s just a recording but to me I’ve my measurement anyway if I don’t it I’ll do it my own anyway so it’s just you know I don’t I stopped doing my machine and do your machine that’s all so it’s a replacement, right? I, I do it on daily basis the machine so now I do it on your machine I don’t do mine so there’s no difference right it just that…

I_01 : It’s like accepting…

AF_0003 : Just that I changed my pattern right.

I_01 : Ok, ok like continuity yah.

AF_0003 : Yah, yah it’s, it’s you think I doing everyday [Interviewer laughed] alright the only thing that I get advice is that do it at those time when you are calmed you see but in my case no I just in the past do it anytime I think I should do it you know then go and sit down and do it you see. So I may have timed it straight after my shower straight after my gym you know so I’ve very diverse type of reading because of the impact of something else you see like you, you just finished your gym heart rate will go up you see so I record good my heart rate as well you see. Hey heart is high but then because I finished my gym you see you know what I mean so I wake up in the morning then sometime your pressure high higher a bit because you just woke up and then maybe have a lousy dream you see so those things are captured there you see diverse. But this tell this good this tells me that because I now standardized the timing and one environment so it’s again really something which is therefore consistent you know the environment consistent. So the mean, it’s meaning the those figures over time will not be impacted by other ex-strenuous factors you see so I’m helpful I, I find this helpful ok so from now on I’ll take pres- blood pressure that way, ok.

I_01 : Just curious you know at the beginning when you started using this blood pressure what motivated you to start using the blood pressure monitor you already had right before last time before you had you were in this study what motivated you to, to, to get it yourself to use it on your own?

AF_0003 : After my heart irregular heartbeat before I don’t border.

I_01 : You didn’t take it before then?

AF_0003 : No, no I didn’t do I, I will always I was always been a rugged individual, born rugged you see so, so and I was in school athlete and so on you see so I didn’t border then once in a while and check up and so on and pressure’s still within range then as you age some borderline you see I still didn’t border you see. Until the wake up call then I just I buy the machine you see?

I_01 : I see ok.

AF_0003 : I monitor everyday.

I_01 : Ok, ok that was then when you first started and you do it like…

AF_0003 : Yes… it…

I_01 : Very consistently throughout…

AF_0003 : Everyday, everyday.

I_01 : Ok, ok and that really makes it much easier when you when you were doing the study?

AF_0003 : No, no, no big deal looked at it I’m monitoring everyday, yah.

I_01 : Ok, ok so you know when the Phillips right they came Miss “B” right she came to like to pass you the device and how do you find about her the, the explanation that she or the instructions that she, she gave was it good?

AF_0003 : Well… well…

I_01 : Was it ok?

AF_0003 : Well I mean I will say adequate right.

I_01 : Ok, ok was it too long?

AF_0003 : No it is adequate…

I_01 : Adequate ok.

AF_0003 : I won’t I won’t say it’s A plus [Interviewer laughed] or, or C why not you see it’s adequate I think maybe I will say B you know right?

I_01 : Ok, ok, ok can alright so like did were you aware also like that, that there was a number on the tablet that you could actually call?

AF_0003 : Yes.

I_01 : So how do you find about do you think it’s a useful thing to have like a contact number and or do you use it?

AF_0003 : Yes I mean I think for your general respondent having such a number is useful because if for example suddenly on certain day the blood pressure shot up alright? They should really let you all know, it’s now for some reason it gone up to 190 or 180 you see then it’s good to alert you then to wait for the next medical appointment you see then, then you all you, you already saw it in your indicator you know? Then, then you say this is the case which you may want to intervene you see right than wait for the time the doctor will when it’s too late to address this patient’s you know? So you know when there’s some exception readings I think you all should somehow be aware and somehow interact with patient to find a why you know because the patient some may not be “intelent” intelligent enough to know that there is something wrong you see right? So it it’s use to find if you said oh because I’m quarreling with my hus- wife or husband of course your blood pressure goes up you see. Then is no issue you see but at least you know then you said hey you shouldn’t be read recording straight after a quarrel you should wait for a calmed period you see. But, but it could be due to medical factor you see for example we all age and then there are time when may not be heart you know may not by the AF you know may be something else cropped up you know then it’s good for you to know. Because since you the patient is on your device since there’s something come back which is sort of a seeing something unsatisfactory then it’s good to alert you see alright since and patient here patient is not intelligent to himself or herself go for medical ad- advice or emergency and so on. You all should say you better go to emergency you see you know what I mean that will be I think for you all your purpose having a device you all can extend your service to help the client alright ok? So, so that the machine is there help the client don’t just focus on just you know hey the reading you know [Participant and interviewer laughed] I, I I’m for my research only but then…

I_01 : Correct, correct.

AF_0003 : Hey I see one nine 196 high blood pressure or below 100 you know this is danger point you know then why don’t you even tell the client you see you have the right cord you know right?

I_01 : That’s right that’s right yah, yah.

AF_0003 : Down there you see so maybe for that each time you reached that reading those exception be come out you know “bling” you know so somebody’s hey this patient needs help you know so from your side as a researcher you alert the nurses this patient is may need help you know? So that is I think additional…

I_01 : Service!

AF_0003 : I would say since the client have been having that and you have the sort of computer you know connection that is something which you all can usefully offer that service to serve the client right he’s a patient, right?

I_01 : But actually I was asking about the more of the number that you know on the was on the tablet so you, you know that there was a number there right yah did you use it in the end like in the meantime?

AF_0003 : Now I, I, I called the number on, on the tablet server’s tablet right then I asked for when are you collecting I was quite dis- sort of disappointed that the person said that I’m admin you know I’m only admin I’ll get the nurse to call you back you see now that was something a let down. Because you gave me a number I called I will call right then you should really straight away passed to the nurse or whatever it is you know if you said you can’t said I’m the admin then I’ll get the nurse to call you, the nurse never called that’s where the disappointment come about. Nobody call that was yesterday you know then I’m not yah, yah it’s, its yes- yesterday nobody call you see so I took the initiative later on and calling the you called I the Miss B I sm- Whatsapp her or whatever is you know right then she said her colleague will call me. That’s when you called me but I was driving a car each time I drive a car like when you once I think once you called me I said…

I_01 : I know.

AF_0003 : Inconvenient to talk you see when I drive a car I don’t want to talk because…

I_01 : Yah I know I know it’s not to talk.

AF_0003 : Very clumsy for old people you can crashed on each side you see.

I_01 : I know.

AF_0003 : So but then I called you back straight away after once I realized hey there’s a missing number.

I_01 : Ok, ok but it was disappointing that you couldn’t the I mean like when you called it wasn’t a medical staff imi- like immediately who answered.

AF_0003 : Yah I don’t mind the admin doing the first screening but once they know realized who it is they should get the nurse you see but…

I_01 : How, how you do that?

AF_0003 : But the number is given alright the first round screening admin it’s fine because some sometime people called for not important questions and chit chat and so on you see alright, you screened. But once you find that hey I when are you calling getting these nobody come and get it back you see then it’s something more, more relevant to this research you know then all she said is I’m admin I have to get the nurse to call you back you know and nobody called me back. So that’s where the disappoint come that means if I’m a in a real crisis I called your number it’s not dependable so why give me a number which not dependable you know the what 699 something…

I_01 : Yup correct, correct.

AF_0003 : You know that it’s printed on the…

I_01 : It’s on the…

AF_0003 : In the multivar you know?

I_01 : That’s right yah.

AF_0003 : Yup so why give a number when I just talked to admin and say hey not, not my problem [Participant laughed] none of my business you know.

I_01 : Yah so it’s much better if it’s like more like somebody whose can you know and, and somebody is really listening somebody really listen to…

AF_0003 : I, I don’t mind if it’s centralized but please go to go to some source to make sure the client gets contacted if you screened and find that it’s relevant you know if it’s not relevant chic chat then fine you said I’m admin is fine. But if it need some information right where these who is going to collect it back you know get, get, get the relevant party to notify the relevant party you see right not just admin you see. For example you said ok the nurse I’ll get the nurse then the nurse will know what to do the nurse will call you right then at least but she blur, blur you know [Participant laughed] that’s bad so that number shouldn’t have been there at all if, if that’s what is there you know.

I_01 : Sure definitely we are taking of these all because we really want to hear but also you mentioned that you, you kept you kept track of the use it’s very useful to keep track of the of your blood pressure over time that graph right? So did when you went did you see your doctor in, in this time period right and then did you bring this like tablet to or these information to see a doctor?

AF_0003 : No my, my medical appointment is every 4 months or 6 months so this, this period…

I_01 : It’s too short…

AF_0003 : Yah no there’s no overlapped you see…

I_01 : No overlapped ok.

AF_0003 : I haven’t seen Doctor “A” yet right?

I_01 : Ok, ok, ok then like how do you feel about the overall duration of the study is it too short too long just right?

AF_0003 : I think for this purpose it’s, it’s right.

I_01 : Ok just right.

AF_0003 : It’s yah just right I don’t know what are your main underlying purpose of your study so, so I won’t know depends on your intention of what you are trying to get research results findings you must have some initial inclination of what findings you want to do. Then you used these data to substantiate it or reject that hypothesis you see so then your duration is fixed by you, you see is that enough data what you called time series data or is it too short too long you know I won’t know. But to me 6 weeks is fine for me…

I_01 : It’s fine ok.

AF_0003 : Right but if it’s too long and you don’t need it then is burdensome you know it’s burdensome because you must make a point to go and because it’s obligation to record it you know I missed a day but on my own if I missed one or 2 days if I’ve gone to KL no big deal you see. I just forego it one week also no big deal you see but if my obligation I make sure I do it so even though I come back late in the afternoon I quickly go and measure you know so I give I give back the input and before I left in the morning at 7 o’clock I did my reading. So that, that day I had a recording and the following day I was in KL missing that day Monday morning yah when I came back on , on the next day I it late, late evening I give a recording. So at least early morning late, late evening and in between one day missing you see so I still give you a recording only one day missing, this is obligation but on my own I won’t I won’t do such readings you know I just I’m on holiday you know why, right?

I_01 : Ok, ok, ok so next one is that…

AF_0003 : So you, you got so the duration is up to you, you know but my, my feeling is if it’s too long it’s burdensome for the client right say if I do it for one year continuously then it’s burdensome because you know I’m obliged to make sure I give you a reading everyday.

I_01 : So what if it was not a study right so if it was kind of like something that you maybe you bought it right and, and so you can used like for a time period of and you have all these features that are already in this tablet and the device. So would you be ok if using it because you that will be with you for quite long right you know what I mean?

AF_0003 : Oh yah, yah, yah I wouldn’t have bought my machine if I, I have you all [Interviewer laughed] which linked up to your computer you know I would certainly buy yours you see even if it cost more that the other one because now I got linked up to your computer and get info you see. I will choose this option better than the other one you see right certainly right?

I_01 : So this, this option is, is really something that it’s like and you find it quite very, very useful?

AF_0003 : Yes, because now I’m hooked to the university alright so in other words I know that occasionally I’ll be getting some care and support you know right then I’m on my own you know then I’m my own nurse every 6 months only I get consultation or why not you see? But at least I’m hooked up there I know hey you all are at least getting info on me you know so how further will you sort of extend this service I won’t know or is it just maybe for collection researcher nobody give a damn. [Interviewer laughed] Then I you know that means but if is intention later on from this it progress to something like a, a client support you see right then for example let me give you exam- for example the client is monitored and he’s ok careful he doesn’t even need to see the specialist that often right that’s fine. So you can used this to monitor his condition you know hey, hey, hey he’s ok you know right he doesn’t need to come every 3, 3 months or 4 months you know so that’s fine you see know give him more medicine he comes once a year. Because looked he’s, he’s fine you know so that, that may be good you see but provided you have this support but this is for period for research then the client get, get nothing out of it you know. It’s only you guys getting something out of it getting something into your computer system but the client is supplying you free what you called data you know…

I_01 : Data yah, yah.

AF_0003 : That’s all you know then it’s a one way you see?

I_01 : Yah, yah so it just that I think they ultimately also want to you know see it, it works…

AF_0003 : I hope so I hope so because… [Interviewer laughed]

I_01 : And implement it more widely you know yah.

AF_0003 : I hope so if, if doctor “A” knows that you now have access to client and if this available why don’t you used that access since there’s info coming in you know alright then you, you looked you get your researcher give me the exception report you know? Because if things are coming fine I don’t need to know but if there are some irregular readings let me know then I can have a special look at the client I don’t have to wait for him to came and see me and check him on that spot which I had been missed the reading oh he has forgotten something happened in the past. He doesn’t know right then you then the then the read then the medical guy may missed some critical readings somewhere on the line you see alright before it’s too late you see. But now you have daily you know information coming in you know life, you know so why not exception report when you have readings above a certain band or below a certain band low lower side upper side alert the specialist you know. Passed info there it’s up to him whether he need to address it or not but at least he’s informed you see then he know that client has some problem should I call him in specially you know or not you see or should I send somebody to go and a nurse go and check him up you know you see what I mean? That will be a more advance level of service offering you see right which a specialist would like to offer if he wants Singapore to progress to go up the, the you know the rep- repute, reputation stake you see.

I_01 : And I and I think they’re really seriously considering it you know given that you know that they have a lot studies in this tele health programs thing…

AF_0003 : You should [Interviewer laughed] because that is our brand you know Singapore brand by doing that we have more overseas patients coming in to say we are the top medical centre in Asia. Then you know if you if you do not think of advancing ev- every now and then like Olympics you know scored the next record then you know after a while people overtake you, you are you’re one of those common you know bypassed by people you know?

I_01 : That’s right that’s right.

AF_0003 : I think Singapore with in, in certain fields we can we can sell medical is one of them you know certain we cannot be outstand in football or rugby and so on you see we are a small population but in medical brain subjects we should be ok to, to, to be among them you know ok.

I_01 : Ok so like how do find about the overall like ok the device do you find it was too small like you know the, the screen and…

AF_0003 : For me it’s fine.

I_01 : It’s ok.

AF_0003 : For me it’s fine.

I_01 : The, the font was it too small also?

AF_0003 : No, no, no it’s big enough…

I_01 : Or, or normal?

AF_0003 : Because if you, you need too big then space it occupied my space you know I, I, I would rather limit it to a certain size then it won’t take so much space you know alright.

I_01 : So if there were other options like for example you know a phone app oh no sorry a computer a desk top version or like you have a phone version you know like a mobile phone version which, which devices would you prefer or you prefer something that’s non electronic?

AF_0003 : Let me put it this way this ans- answer this question answer depends on the person’s age hierarchy right if an old foggy like me I, I’m not keen to be very advance in this what you called it…

I_01 : Like electronic devices…

AF_0003 : New, new gadgets and so on you see?

I_01 : Gadgets ok.

AF_0003 : So some people they may not even used a mobile phone you see so when you mentioned those things these are for tech savvy people you see but for people like my age group I think we prefer something more simple.

I_01 : Like paper?

AF_0003 : Not necessary your, your, your, your tablet is good you know…

I_01 : Ok like…

AF_0003 : Like even email is good for me right?

I_01 : Email ok.

AF_0003 : But, but for some people of my age email is no good you know they don’t even used the email ok so you must see which client you address you see the, the intellectual level as well as the age group age group…

I_01 : But for…

AF_0003 : But if it’s for 20 something 30 something is you can give him anything you know a computer or even put something on his wrist you know wrist watch and so on that’s fine you know.

I_01 : But for yourself actually we worry about you know your…

AF_0003 : I I’m ok with email, email.

I_01 : But email we cannot do the measurement so like if it’s but it’s a desk top app, app you are ok with it like a desk top kind of but you know it is the same app but it’s on a desk top?

AF_0003 : No problem.

I_01 : Computer…

AF_0003 : No problem.

I_01 : You’re ok with that what about non, non electronic like paper will you prefer like something like paper form that means like you record down your you know you record down and then you sent to us like the, the, the, the readings…

AF_0003 : But if I sent you by paper form…

I_01 : We can send you brochures all that.

AF_0003 : But if I sent you in paper form why don’t I put it in a on email it will be more convenient paperless you know right I can just filled it up you see but paper is, is equally good for me you know.

I_01 : It’s ok?

AF_0003 : Yah it’s ok right.

I_01 : Ok but for you what you prefer of all these options available let’s say you have all these options on the table right so which one would you, you know most inquisitively and most attractively take…

AF_0003 : How often do I send need to send paper to you?

I_01 : Paper will be like you know it can be like every week you sent us the, the your, your, your readings you know and then we also send you like brochures and educational materials to…

AF_0003 : Ok then I, I prefer the paper.

I_01 : So you, you prefer paper compared to tablet that you had just used?

AF_0003 : Like, like I just fill in numbers there right?

I_01 : Yah you just put the numbers and then you just sent to us mailed, mailed to us and then like the…

AF_0003 : Through the tablet is it?

I_01 : Like for the, the, the tab- no the paper and the tablet right so the…

AF_0003 : No, no the ta- ta- the tablet I entered the number it automatically go to you, you know it automatically go to you.

I_01 : If it’s the tablet you don’t need to enter anything you don’t need to enter your, your blood pressure because it’s automatic, yah.

AF_0003 : Yah, yah so, so in other words if I don’t need to do to do anything that’s of course the best option for me you see [Interviewer laughed] alright the readings it go alright to you right that’s great that’s great you know it’s only when I need to do something that’s different you see. Then I got to type out a special email why not and so on it’s more work you know.

I_01 : Yah and then paper form there won’t be any videos also it wasn’t possible so we can only at the most for example sent like educational materials and, and maybe like some brochure some booklet or something like that so you, you…

AF_0003 : That I prefer the video and the tablet.

I_01 : Ok, ok, ok got it, got it ok.

AF_0003 : Yah it’s, it’s, it’s, it’s not just video right it’s audio video audio you see so that…

I_01 : Audio video.

AF_0003 : You have a whole thing and you get to play it again right whereas paper I think there there’s a lot of limitations right?

I_01 : Ok more interactive more dynamic you prefer something like that?

AF_0003 : Yah it’s well I said you both see and hear alright that’s different from just seeing you know on, on cold print you see?

I_01 :Yah, yah ok so like last few questions we just want to ask you, you know would you want to see anything additional to the features that you have already seen you have already used in this tablet and device you know something that you feel will be helpful in making it better?

AF_0003 : Are you confining to just AF?

I_01 : It can be anything, anything you can think of you know anything that you want to see…

AF_0003 : Oh ok I, I think ok I, I must I can speak only on behalf of the people of my age group for elderly people then any information on age related diseases will be most helpful age related disease in other words there are certain standard disease which we should prevent. How do we prevent from it from getting hitting us or at least we are early, early notified we know how to minimized the impact you see so these are the age related diseases such as cancer you know heart diseases or, or, or chronic these chronic diseases affect old people you know. Dementia, Alzheimer you know what you called that hand shaking what you called that the, the what’s, what’s those things where do you shake hands you know can’t move you know do you know the medical thing condition?

I_01 : Not, not really but you mean the, the like the movement is it?

AF_0003 : Yah the hand they can’t, can’t move you know?

I_01 : Ok so you, you, you…

AF_0003 : You know those are age re- when people get old these are the normal diseases who knocked them you know so all these info coming in will be helpful because then we can sort of looked out for those things happening and what are the preventive measures? Then we would sort of live it that healthy lifestyle to push back these things from hitting us early you know one day we will go alright whether it’s 85, 90 or lucky 95 you see we will go but at least we will by being able to get these advice live healthily. Practice in a way where we pushed it back and when we go it will be less painful less suffering you know for both ourselves and our caregiver that’s what I like to see you know additional info given.

I_01 : Addition information…

AF_0003 : Yah all the age related diseases.

I_01 : In term of message like the way like ok we talking about the delivery of the…

AF_0003 : The, the, the yah the, the video is very helpful very helpful…

I_01 : The video so, so the videos ok, ok really works ok.

AF_0003 : Yah very helpful.

I_01 : Ok, ok then what about like other ways of like may could be like messaging you know like we, we sent more messages like that…

AF_0003 : Oh it’s fine for me.

I_01 : It’s also ok?

AF_0003 : Yah I’m, I’m ok in fact I, I think video helpful for a lot of people to less what is literate you know they can watch and hear but for us who are well sort of used to reading and the reading is very fast we just alright it sinks in so no, no problem. I think reading you can put more things in video you, you have only confined yourself for certain things you know but writing you can put all the things in and then well I, I welcome more info alright that’s why I read books.

I_01 : Oh ok what about if let’s say we have another way, way of measuring let’s say heart rate monitor right other in addition to the blood pressure monitor we add more of these measurements for you would you be comfortable using more of these or not? Kind of you know you might have to like maybe wear put a wrist watch here…

AF_0003 : Yah why not?

I_01 : Connect then you connect for example you are ok with that?

AF_0003 : Yah, yah, yah.

I_01 : Ok so you don’t mind?

AF_0003 : An- an- anything which helpful [Interviewer laughed] what I mean is that’s why if it really helped us in promoting our health yah I’ll welcome it you know yah.

I_01 : Ok, ok, ok then like…

AF_0003 : It let me put it this way my management is discreet data collection I measure once a day that’s all the info but I believed if you have a wrist watch on is, is continuous you know so that you can know what every moment you know? Then that’s more helpful because you have up to date I would used the word real time you know the real time full more on your health why not you see if you have all the blood pressure reading [Participant laughed] and, and every heart rate everything else there hey your blood sugar or not all those things are there in real time, excellent alright. Like I said people like me data is easy alright yah.

I_01 : Ok and you don’t mind the privacy aspect of it you know what I mean…

AF_0003 : No, no I, I I’m…

I_01 : You know what I mean?

AF_0003 : I was a regional researcher it, it it’s only then people steal my data to for some ulterior motive which is bad for me then I don’t like you see because it harms me but if I’m giving my data which helped in research and also helped myself always helpful really.

I_01 : So you don’t mind with…

AF_0003 : No, no, no.

I_01 : Especially if it’s your health care provider?

AF_0003 : Yah, yah no, no. [Interviewer laughed] Yah only when people who harm me collect data alright then of course I won’t like them to know you see alright but if I’m giving my care provider why not you see alright?

I_01 : Ok, ok so actually I think this is basically all about it you know if you have any extra thoughts about you know about this cam- this whole process or like anything you want to see in the healthcare you know in the future you know like…

AF_0003 : I want Singapore to be number one at medical service [Interviewer laughed] so you guys now already making these step so that you are interactive with the with the with the client with the patient I’m I, I think that’s the way to move forward. One day that you know we’ll sit in the house you sit there you know what [Interviewer laughed] going on here you see and then we can say hey we, we run a short lesson one, one email to you then you said go and collect your medicine it’s already ready in the pharmacy you know. Then all these are interactive then there’s a cut down in all those inefficiency, waiting you know time and so on…

I_01 : That’s right that’s right yah.

AF_0003 : And even the specialist will be spending less time on things which are minor, minor issues you know, right?

I_01 : Right yah.

AF_0003 : They can focused on the major issues then their time well spent you see…

I_01 : That’s right that’s right.

AF_0003 : So they also be happier that, that they are well utilized you see.

I_01 : More efficient also more like holistic the whole day ok.

AF_0003 : Yah, yah for me it’s, it’s an advancement because Singapore we, we in Asia is one of the top cities…

I_01 : That’s right.

AF_0003 : And let’s moved you know continued moved up you know don’t, don’t stay stagnant you know things are doing fine here ok, alright.

I_01 : Definitely we are taking the first steps I believed…

AF_0003 : It’s…

I_01 : I mean in terms of this, this part of it the electronic tele health thing.

AF_0003 : If for example I have very low opinion on UK medical service you see.

I_01 : Oh ok, [Interviewer and participant laughed] where do you hear about it?

AF_0003 : So let’s, let’s not sink let’s not sink to that level they were once very advanced when we were in the colonial days now they sink to that level where they…

I_01 : Heard a lot of stories about it [Interviewer laughed] yah.

AF_0003 : Ok don’t sink [Interviewer and participant laughed] we were there keep it there you see don’t be so arrogant I’m a what empire which empire hey now no more you see it’s sunset you know?

I_01 : Yah taken over those countries already yah.

AF_0003 : Sunset, sunset you see ok.

I_01 : Alright.

AF_0003 : Every, every finished is it, ok?

I_01 : I’ll just stopped the recording.

AF_0003 : Yah, yah.

End of interview
