# Supplementary material for: Patient perspectives of the Self-management and Educational Technology tool for Atrial Fibrillation (SETAF): A mixed-methods study in Singapore
Source: PLoS One. 2022 Jan 21;17(1):e0262033. doi: 10.1371/journal.pone.0262033 (PMC8782297; doi:10.1371/journal.pone.0262033)
Supplement: S1 Data — (ZIP) [file pone.0262033.s006.zip › AFP2_IDI_0002.doc]

Participant ID number : AF_IDI_0002

Interviewer Number : I_01

Length of interview : 44.34 mins

I_01 : Ok so I just have to ask one more time for the rec- to be recorded if you don’t mind if this interview is recorded it’s the, the interview will be used for research purposes only is it ok?

AF_IDI_0002: Yes ok.

I_01 : Ok so the first question is you know how long have you been diagnosed with AF irregular heartbeat?

AF_IDI_0002: Since 09 that is about 9 years.

I_01 : Ok and how did find out about it?

AF_IDI_0002: How do I find out about that?

I_01 : Yah how did you find out about it?

AF_IDI_0002: There were certain, certain whereby it’s irregular and I couldn’t find out until there was one day I, I catch it on ECG I just a walk in and catch it on ECG and yah that is how then further studies is being made and that’s how they, they find I mean they term it as AF.

I_01 : Ok and during that time period right did you were on did you take any medication were you prescribed any medication when you found out?

AF_IDI_0002: After during the investigation?

I_01 : Yah when you when you first like at the beginning when you first found out?

AF_IDI_0002: Well they trying to when I first sorry when I first find out…

I_01 : Found, found out yah correct so we start at the beginning first when you find out about this and then how it progressed and yah…

AF_IDI_0002: Ok the during the investigation medication wise they try to give me beta blocker and it doesn’t work yah so it doesn’t work on me because I went I couldn’t function after that yup.

I_01 : So you tried other things?

AF_IDI_0002: Tried other things, for example?

I_01 : Other medication or like other kinds of mediation for example?

AF_IDI_0002: No they I mean I wouldn’t try anything on my own, [Participant and interviewer laughed] yah.

I_01 : But the doctors they you, you saw the doctors and they gave you advice about what to do like?

AF_IDI_0002: Yes I mean they let me try the beta blocker and it doesn’t work and they try I can’t remember it’s like they probably try those pill in pocket then and stuff like that yah, yup.

I_01 : Ok, ok so like you know in this time period when you had after you first diagnosed with AF right how did you manage your condition how did you like how did you manage or…

AF_IDI_0002: How do I…

I_01 : Yah how did you manage it?

AF_IDI_0002: Manage…

I_01 : In general so like are there any like technological methods that you used or like how do you what do you do to monitor it constantly and regularly?

AF_IDI_0002: Oh I couldn’t because I don’t have any machines that is able to detect the irregular heartbeat so even the norm- normal blood pressure couldn’t, couldn’t like able to help me how do I manage? I could not [Participant laughed] ok it comes and go without knowing anything and I mean it doesn’t have warning or anything like that so yah if you say manage wise I couldn’t I try to eliminate cause like caffeine and things like that…

I_01 : I see ok.

AF_IDI_0002: But it doesn’t it doesn’t seem to be ap- applying or something like that yah, yup.

I_01 : So where do you get the information about the you know you mentioned caffeine right you, you, you ate less of it I mean like you through like maybe coffee or something else like how do you where do you get this information from in general in general?

AF_IDI_0002: In general well if it’s I think I go if I am not wrong I do read up a little bit so because everybody try to be a doctor of their own. [Interviewer laughed] So friends or me friends and relatives will say oh you should kind of like stopped eating these doing stopped doing that and things like that so it’s like I’m trying in the midst of trying to find out what exactly caused the AF, so I mean I do my own elimination yah.

I_01 : I see so your, your friends and family what do they tell you generally or I mean to help you…

AF_IDI_0002: Well I think in general they also just find that they probably feels that well caffeine makes the heart go a little bit faster or, or certain food or something like that so that’s why they have their own ideas as well yah.

I_01 : Ok what about like doctors their advise?

AF_IDI_0002: Doctors, doctors advise I can’t remember [Participant laughed] that is like 10 years ago yah so I can’t remember exactly what kind of advise but in yah I, I, I think they do try to ask me to eliminate certain stimulants like caffeine coffee, tea things like that. I think they do advice me on that but it’s, yah.

I_01 : Ok it could be also in between like you know not just the beginning when you first diagnosed but also like after that in, in the middle right like how do you like where do you get information from you know? Like say to remind you about what you need to do or not, not don’t need to do or you can’t should not do so was there any other sources like the doctors and like did they what did they tell you in, in the in between, yah…

AF_IDI_0002: Oh I think one of the doctor…

I_01 : As it progress?

AF_IDI_0002: In well because straight after that I have the my first abrasion so they realized it’s, it’s permissible and it’s really due to not thyroid right I think yes, yes I forgot they quickly want to check on my thyroid the sodium and things like that yah. So nothing due to that so went to abrasion so after that in between you were saying that how do I what, what’s the question again?

I_01 : Like so like how do you where do you get the information to help you like give you knowledge to help you manage…

AF_IDI_0002: Where do I get information is it?

I_01 : Information yah.

AF_IDI_0002: Internet and doctors [Participant laughed] yah so doctors mainly back to TTSH one of the doctors can only tell me that you must lose weight because I eat up but, but what other than that can I do right beside losing weight that is quite a know factor…

I_01 : I see.

AF_IDI_0002: But be between that it’s like what can I do right and I I’m having I’m also anemic as well at that time so that was quite a few years down the road already I’m anemic and the only advice that she gave me is you should lose weight that was not helpful. [Participant laughed]

I_01 : Ok, ok so were there any other ways that you feel that could be like they were more helpful because like you mentioned like the doctors and you know this advise this particular advise is not so helpful to you in managing and helping you to improve right? What about like other things were, were there any other things that you found more helpful?

AF_IDI_0002: Well, well until recently I think I think because during when my period with TTSH is that there there’s really not much advise beside, beside having the increased of medication which is like it doesn’t really help already the that medication. So, so yah this the other some one other doctor actually, actually advised me to take another blood test that’s where I got realized that it’s like I’m anemic so I mean it’s like whether is helpful or not I think is also the wisdom of the doctors. [Participant laughed] Yah to give me I, I mean go through to give me certain advice to take certain test yah if it’s, it is not just like it’s like the one of the doctor were said I’m, I’m fat or things like that which is not helpful which I really want to exercise…

I_01 : I understand.

AF_IDI_0002: But I cannot exercise yah so actually a lot depend on the wisdom of the doctors [Participant and interviewer laughed] or the knowledge of the doctors as well yah, because on our side we can only read that much right, and there’s a lot of terms that we also don’t know. Yah so I’m not sure whether I answered…[Participant laughed]

I_01 : Definite definitely it’s use it’s relevant what about like you mentioned about reading so where do get the some of the information from like reading sources?

AF_IDI_0002: Reading sources there will be from different I would say medical whether be it like, like, like I think from internet…

I_01 : Internet.

AF_IDI_0002: Mainly from internet so you don’t realized that’s why and these information sometimes you don’t know whether it is like it’s applicable [Participant laughed] or not that kind of thing so you just try read according to the dia- doctor diagnosed with AF so you try to read what is AF. And things like how to manage it and things like that which didn’t talk much how to manage it so yah so it just tell you the symptom and, and that’s it.

I_01 : Ok, any particular name of the website that you, you go through?

AF_IDI_0002: No can’t remember because there are various, yah.

I_01 : Various ok, ok in general that’s how you feel about it ok, ok so we just go a little bit to the tablet during this 6 weeks of the study so how did you use the device on, on a typical day?

AF_IDI_0002: Typical day that will be in the morning in the morning just after I wake up or, or, or when I can remember [Participant and interviewer laughed] yah so I’ll just take the once of the blood read- blood pressure reading and that’s it. And usually there’s it, it comes to the survey as and when, when I’m, I’m more I, I have more time yah, ok.

I_01 : Ok is that like ok, so you do this like everyday in the morning was there any other days where you do it at, at in another part of the day?

AF_IDI_0002: There’s only once whereby I was away so, so I can only I was away for one day so I can only do one at night or, yah.

I_01 : So on generally you, you, you take one time per day is there like more than one time you tried using it?

AF_IDI_0002: More than one time also during the, the day it’s when the reading looks a bit odd [Participant and interviewer laughed] probably I moved or something like that so I just take another time just to, to make sure that’s it yah usually once in a day, yah.

I_01 : Day you mean by morning or afternoon or noon…

AF_IDI_0002: Morning.

I_01 : Morning ok.

AF_IDI_0002: Usually I’ll, I’ll take in the morning yah, noon I don’t bring the machine out, [Participant and interviewer laughed] yah.

I_01 : Ok is there any reason why you take in the morning like…

AF_IDI_0002: In the morning...

I_01 : Rather than afternoon?

AF_IDI_0002: The reason is like I think I think more the time the time is, is more consistent yah because I of course it’s afternoon you don’t have the machine with you when you go to work or something like that and at night you probably forget because sometimes it’s too tired. Or sometimes you have other appointment or something like that so you can’t keep it at one log in at one particular hour or something like that yah.

I_01 : So in terms of remembering also, also ok.

AF_IDI_0002: Correct.

I_01 : Ok then the next part is that like overall right how comfortable do you feel about the tablet and app how comfortable like in terms of like using it?

AF_IDI_0002: I’m ok I’m like pretty comfortable with it it’s quite easy to use that’s all, yah.

I_01 : Ok, ok so were there any like aspects that were easy for you like which part which feature for example would you say it’s easy?

AF_IDI_0002: It’s quite easy [Participant and interviewer laughed] yah I mean in general sense it’s quite easy of the overall yah particular part I, I don’t know. [Participant laughed]

I_01 : Ok because like for example like taking the blood like measuring the blood pressure…

AF_IDI_0002: It’s easy.

I_01 : Or could be like using the app all that?

AF_IDI_0002: It’s easy. [Participant laughed]

I_01 : Ok, ok, ok were there any aspects that were difficult for you or not you feel personally?

AF_IDI_0002: Difficult no but a little bit time consuming that will be the survey.

I_01 : Ok, ok so we asked a little bit about that later on ok, so what about the interfaced of the tablet like the size of the screen you know the font size the different scrolling…

AF_IDI_0002: Oh that’s, that’s fine with me.

I_01 : Through the different the parts of the app?

AF_IDI_0002: Different parts of the app…

I_01 : Like in…

AF_IDI_0002: But there’s only one app. [Participant laughed]

I_01 : Correct there’s only one app but the app you have to go into different pages right you know you…

AF_IDI_0002: The home…

I_01 : Have to watch the video for example…

AF_IDI_0002: Like it’s, it’s…

I_01 : How do you find that?

AF_IDI_0002: Easy it’s easy yah that’s but yah it’s, it’s…

I_01 : Do you find it do you like it personally?

AF_IDI_0002: Like it [Interviewer laughed] I’m actually neutral, [Participant and interviewer laughed] no particular liking. [Participant and interviewer laughed]

I_01 : Ok, ok so what about like the content you know like do you find it too difficult for example the videos or…

AF_IDI_0002: No but I guessed it’s like the video is a bit towards more elderly [Participant and interviewer laughed] yah, so but I mean it’s like that help helpful information, yah.

I_01 : Ok, ok so we ask a little bit about the video also a bit later what about like the quantity is that too many things to…

AF_IDI_0002: Too many things to…

I_01 : To do like the everything in all overall right was, was there too many things that you need you felt you need to do?

AF_IDI_0002: No I’m fine with that.

I_01 : It’s ok.

AF_IDI_0002: It’s ok.

I_01 : Ok the what about the quality the quality meaning, meaning like you know you how do you find the survey and what it was measuring do you find that it was useful?

AF_IDI_0002: Is it useful I think you guys have to answer that [Participant and interviewer laughed] I’m sure not me I just follow that sort of thing.

I_01 : Because we wanna get your feedback about how you feel…

AF_IDI_0002: The…

I_01 : About how you feel about the like everything you know I mean this is more like the overall view of things how you felt how you felt it?

AF_IDI_0002: I’m, I’m actually ok how I yah the, the survey everything it’s ok except for one video that it’s like not playing so…

I_01 : Ok so we’ll go back to that later ok so this is more to the overall experience that you have…

AF_IDI_0002: Overall experience it’s ok definitely that’s ok.

I_01 : Ok, ok meaning what like…

AF_IDI_0002: Ok that means it’s like it’s easy doesn’t cost a hand a leg to try to figure out what it’s going to do and things like that like it doesn’t take a lot of time yah, yah.

I_01 : But what about like attractiveness or like you know like in terms of like lika- like in terms of liking particular aspect aspects of it?

AF_IDI_0002: Liking, actually neutral seriously it’s quite neutral there’s no yah…[Participant laughed]

I_01 : Sure, sure we want your honest opinion so…

AF_IDI_0002: Yah of course it’s like the interface can be it’s easy but it’s like can be more the only, only pictorial that I see is the sunflowers that’s all.

I_01 : Ok, ok that that’s which is the…

AF_IDI_0002: That other than the video yah so yah.

I_01 : Ok so we’ll talk about the different aspect of the app ok the, the first one is the blood pressure monitor right so how is your experience using it what’s when you when you used it? Were there any particular feelings that you had you know like say it gave you like any stress anxiety or were you like happy to used it [Participant and interviewer laughed] different emotions whatever was there any emotions that came about?

AF_IDI_0002: No there’s no emotions just like…

I_01 : That you can think of?

AF_IDI_0002: Everyday that’s your duty to do [Participant laughed] yah.

I_01 : Ok well do you find that it was easy or difficult?

AF_IDI_0002: It’s quite easy yah…

I_01 : I see, yah.

AF_IDI_0002: To blood pressure moni- monitor…

I_01 : Yah about blood pressure monitor yah…

AF_IDI_0002: It’s really good as compared to what we are. [Participant laughed]

I_01 : Ok, ok so the next one will be about the survey how do you find the survey that were sent to you the device I mean like the length of it?

AF_IDI_0002: The length of it?

I_01 : The survey, yah.

AF_IDI_0002: The length of it ok I need to sit down to, to really go through the survey so yah the length of it…

I_01 : Was it too long or too short or o- just right?

AF_IDI_0002: The, the, the regarding the publication regarding the, the emotions and things like that one probably can yah probably can shorten a little bit because it’s like I guessed if it’s if it’s I, I don’t know is it your, your participants is all around my age or if it’s…

I_01 : We have a range…

AF_IDI_0002: Much older yah probably they probably need a, a shorter one I will…

I_01 : But your own…

AF_IDI_0002: Find it because it takes awhile to for me to read it right and go through it so I’m still o- I’m still fine with it but anything longer I’ll like such a long…[Participant laughed]

I_01 : Ok but you mentioned that it was a bit too…

AF_IDI_0002: Was a bit…

I_01 : A bit too long is it?

AF_IDI_0002: No if it’s a bit longer one a bit longer I’ll find it a bit too long.

I_01 : Ok but at, at this I mean what you did was ok?

AF_IDI_0002: Ok!

I_01 : Ok, ok then what about like the, the, the like the quality of it?

AF_IDI_0002: The quality, quality of is in the question or video?

I_01 : I think maybe the quantity not the quality sorry the qual- the quantity yah.

AF_IDI_0002: Quantity of the survey?

I_01 : Yah so like was it too many or too little?

AF_IDI_0002: I think you repeated quite a fair bit like I this, this I think I’m I about to take a third time third round of the same survey…

I_01 : I see.

AF_IDI_0002: I think so but I think I, I managed to do twice or I can’t remember already yah so I will say during this period of time before and after will be enough. Rather than the, the like so many will be the same yah unless you want a certain reading that is in between yah I I’m not sure, yah. [Participant laughed]

I_01 : Ok because the, the I think the point of like having the same survey also is to kind of like to be able to see…

AF_IDI_0002: Regulate…

I_01 : Yah to see the like measure the same condition at, at 3 different points of time over the, the period but I mean personally you felt that it was repetitive?

AF_IDI_0002: Yah probably that is necessary I’m not sure but I find repetitive that one [Participant and interviewer laughed] ok the same survey again.

I_01 : And that was very obvious.

AF_IDI_0002: Yah it is obvious! [Participant and interviewer laughed]

I_01 : Ok, ok, ok then what about like the videos like were there any videos that you like particularly?

AF_IDI_0002: I like the of course it’s like I, I like the informative one and that is about AF and it’s about what is that the, the stroke one yah then well the other one I like is the, the, the so call the what is that the, the exercise one but it’s a bit a little bit slow. [Participant laughed]

I_01 : Oh ok, ok it’s, it’s too long.

AF_IDI_0002: Yah it’s a bit…

I_01 : Or the pace is too slow the pace?

AF_IDI_0002: No, not the not I mean it’s good, good information on how to exercise how to eat well but it sometimes but some information vary like you know so you say particularly what I like about the, the, the videos that you all sent is the animated videos the more informative one. Yah those that help you when it comes to, to, to I mean to know what is the information that is necessary for me to understand yah, yah.

I_01 : Ok and as a form of encouragement like do you think that it helps or not like in terms of like say you know cooking better or exercising you know going out to do something?

AF_IDI_0002: The encouragement one right it’s a bit funny I find a few that’s why because, because it’s like oh do you know that it’s like what stopped smoking also, I don’t smoke. [Participant and interviewer laughed] You know it was like this, this you know it’ll be you know it’s automated encouragement motivation and things like that…

I_01 : Oh ok so that part is very it’s quite like you, you, you noticed that quite a bit the automated part?

AF_IDI_0002: Yah. [Participant laughed]

I_01 : Ok, ok so it, it didn’t really have a like…

AF_IDI_0002: No it doesn’t have a personalized kind of not say personalized but you know it is it is just automated [Participant and interviewer laughed] like you sh- you know it’s like you should, should exercise is like or you know you should have you quit smoking or something like that.

I_01 : I see ok.

AF_IDI_0002: That was like then you can picked it up very quickly oh that is automated. [Participant and interviewer laughed]

I_01 : Ok so if would you think that if there is something more like naturalized you know or like something that have a more human feel to the app right you think that it will be better?

AF_IDI_0002: I think…

I_01 : About or it won’t make a difference?

AF_IDI_0002: It will I think it will be I, I find because when it becomes you realized it’s automated then you don’t want to read you don’t want to read that motivation kind of thing…

I_01 : Did you have a feel of that when you when you read all that?

AF_IDI_0002: Yes I do that’s why we because it’s you realized it’s automated then it’s like oh just another well ok [Participant and interviewer laughed] you’ll find it’s not so motivating anymore…

I_01 : Actually…

AF_IDI_0002: You still you just know that it is ok it is part of part of the job of the [Participant laughed] ta- or, or the tablet or the or the, the, the survey or something like that yah.

I_01 : So something more personalized would be more helpful?

AF_IDI_0002: Probably will be helpful, yah.

I_01 : But will it encourage you more you feel will it make a difference?

AF_IDI_0002: Not, you mean when it come to personalized?

I_01 : Something more personalized something that more tailored to your needs.

AF_IDI_0002: It probably, probably encour- probably encouraged a little bit more yah then l mean it’s like [Participant laughed] yup correct.

I_01 : Ok so what about like the there’s some like pop up messages right ok we talk a little a bit we talk a little bit about it the encourage the encouraging messages…

AF_IDI_0002: Motivation messages and things?

I_01 : Will there any emotions that came to you when you felt when you when you saw it or not other than you know some of it was a bit funny…

AF_IDI_0002: Funny! [Participant and interviewer laughed] Other than that it was like oh ok like…

I_01 : Other emotions and…

AF_IDI_0002: No, after a while you know the pattern you just skipped, skipped, skipped yah correct.

I_01 : Oh ok, ok and then it, it kind of like you, you had a expectation of it of what what’s to come?

AF_IDI_0002: Yes so yah becomes like you just need to like clicked off or something like that. [Participant and interviewer laughed]

I_01 : Were there too many of it you felt?

AF_IDI_0002: It’s fine it’s, it’s fine but you…

I_01 : Or too little?

AF_IDI_0002: Not say too little I mean that mo- motivational it becomes as I say it become mo- become automated right then you feel that it is just you just chucked it aside. And you just like you don’t feel too many or too little or you just find it a like not necessary probably, [Participant and interviewer laughed] yah.

I_01 : Ok, ok so like con- like we consider the, the features right blood pressure monitor, survey, videos and messages right do you think that for each of these like, like all these features six weeks is enough for the app and device to be helpful for you. Like this time period like the 6 weeks period of study right for all of these features right do you think that they are like sufficient for like for it to be helpful or not sufficient ?

AF_IDI_0002: I think…

I_01 : The time the length of time for all these?

AF_IDI_0002: I think it’s fine, fine yah I, I’ve no I’ve I’m quite neutral about it, yah.

I_01 : Oh ok, ok do you felt that it was like a bit too…

AF_IDI_0002: Too long?

I_01 : Yah.

AF_IDI_0002: Not really.

I_01 : Yah ok or too short maybe?

AF_IDI_0002: Not really as well [Participant and interviewer laughed] yah because particularly it’s after, after my abrasion so I’m, I’m kind of like used it for me to monitor my blood pressure as well so, so yah the, the, the duration is quite ok for me it is, yah.

I_01 : I see ok so would that be a better like say you because you, you just after you had your abrasion right so that would this device would come in handy right when like say after the procedure let’s say you didn’t have this procedure would you still considered using it?

AF_IDI_0002: Would I still considered using it?

I_01 : Yah, yah or would it still you think would it still be helpful for you or not?

AF_IDI_0002: Not ss-

I_01 : Like…

AF_IDI_0002: Not so much because the blood pressure wise is like for me I, I know that my blood pressure is not is quite, quite ok it’s not those like high or too low or something so if it’s not after abrasion this one wouldn’t, wouldn’t help me that much yah wouldn’t help me that much. I think after abrasion it somehow you find that you are being monitored then, then that’s that does bring comfort yah there’s it’s like you realized you are still I mean being monitored so ok, [Participant and interviewer laughed] yah.

I_01 : So that will be it I mean after the procedure you think that it’s more like it’s helpful because of that not so much because of I mean like in order more ordinary circumstances like you would…

AF_IDI_0002: Yah because…

I_01 : You would not go all out to, to, to get, get something like that or would you?

AF_IDI_0002: No I would…

I_01 : Or neutral?

AF_IDI_0002: I wouldn’t go out to get a device or a blood pressure measurement because I don’t really need it, yah unless it’s like it’s being shared by another member who has blood pressure issue. [Participant laughed]

I_01 : Oh in what, what you mean by shared by another member?

AF_IDI_0002: Shared by that means it’s like one machine I won’t go out particularly to used them buy a blood pressure machine unless it’s like it’s a family unit that, that needs it right so yah so tablet wise it’s I would I wouldn’t well it comes together so unless it’s an app I don’t know. I probably will think about it. [Participant laughed]

I_01 : Ok app and the with a I mean it should, should be…

AF_IDI_0002: Connection to like connection to blood pressure machine or something like that yah probably I’ll get I’ll, I’ll think about it, yah.

I_01 : Do you yah…

AF_IDI_0002: Sorry just a app, just a tablet I won’t yah ok.

I_01 : Ok, ok on your own do you have a blood like do you have a device already that you used…

AF_IDI_0002: I have.

I_01 : Like to help you monitor…

AF_IDI_0002: I don’t usu- it’s not for it’s not particularly for me I just used it because my, my mom has it.

I_01 : It’s a blood pressure monitor is it?

AF_IDI_0002: Yes that is not very good for one as well [Participant and interviewer laughed] it’s just a general pressure yah so she has it so I just used it yah so once awhile I, I will just measure it.

I_01 : I see ok once awhile which is like how, how often generally?

AF_IDI_0002: Once a month or when I feel that is like if I have a heavy head headache or my head is heavy or light or something like that I suspect it’s due to blood the pressure then I’ll just measure a little bit see whether to confirm whether it is or not yah. So yah that’s general how I used the BP machine.

I_01 : Is there a particular like device that would like be preferable for you like you know in terms of like…

AF_IDI_0002: AF?

I_01 : Could be yah you, AF.

AF_IDI_0002: For my AF?

I_01 : For your AF, yah?

AF_IDI_0002: I would usually used the oximeter because that kind of like reads a lot of my heartbeat and the amount of oxygen that I have [Participant and interviewer laughed] blood pressure measure I, I don’t usually used that more often is oximeter, yah.

I_01 : And they are different devices right?

AF_IDI_0002: Yes.

I_01 : So would like the number of devices also affect like I mean like will you would you prefer less or more devices like overall?

AF_IDI_0002: Of course lesser will be good but it’s like I can’t bring a I mean I can use a oximeter I can bring a oximeter all around but I can’t bring a BP meter there so [Participant laughed] you know.

I_01 : So the portability also matters like how mobile it is?

AF_IDI_0002: Yes yah I do used a watch I do had a, a watch type of BP reading previously but it’s not so accurate.

I_01 : Is it something like that?

AF_IDI_0002: No, it’s a BP reading that was many years ago but it’s not accurate so it’s like I just discarded. [Participant laughed]

I_01 : So you stopped using for but you felt that the portability actually did help you a bit help you to bring it around ok, ok so we just go a little bit to the part where when you know Phillips right they approached you at the beginning right? So did they teach you how to use the device or not like you know…

AF_IDI_0002: When they sent the machine?

I_01 : Correct, your interaction with them?

AF_IDI_0002: Yes, yah.

I_01 : Ok were did you tap did you feel that it was use useful or not when you interacted with them and they, they taught you?

AF_IDI_0002: Yah it is their, their help clear I would say yah clear…

I_01 : Was it Michelle who went down?

AF_IDI_0002: Yes Michelle same person yah.

I_01 : Ok and was it very long?

AF_IDI_0002: No, was like 15 minutes 15 minutes and…

I_01 : 15 minutes ok.

AF_IDI_0002: And then that’s it, yah.

I_01 : Ok, ok do you find it particularly like easy or difficult or how do you feel about the you know the way the message was conveyed to you?

AF_IDI_0002: It’s easy, yup.

I_01 : It’s easy ok, let’s say the, the Michelle didn’t I mean she went down to give you the device but she didn’t teach, teach you how to use it, will you feel that you still be able to use the device on your own?

AF_IDI_0002: Yes probably there’s some written instruction or before the yah before the whole I mean inter interface for the how to teach you how to use the device yah I think that will be will be fine.

I_01 : So you need an instruction to, to, to look at it first before you can I mean like let’s say there was nobody to teach you, you will need the instruction sheet or something?

AF_IDI_0002: Correct, probably because it’s like it it’s designed to what you guys want right so the instruction wise will be good from to, to if she’s not there, yah.

I_01 : Ok, ok so like you had a interaction I mean are you aware that there’s a contact number inside the form the app that you can you can used.

AF_IDI_0002: The NUS and then the phone, yah correct.

I_01 : Yah so did you man- manage to use any…

AF_IDI_0002: No.

I_01 : Any [Participant and interviewer laughed] time during, during these period?

AF_IDI_0002: No.

I_01 : Ok, so like the contact number at the beginning was it relate to you or told you that you know like Michele when she went down was, was it told you that there was a contact number?

AF_IDI_0002: There was a contact number if but I can’t remember is it pertaining to the machine or is it my condition [Participant laughed] I forgot yah so I couldn’t remember that, yah.

I_01 : Ok do you find that the contact a contact number will be useful for this app and tablet in addition to everything else like a kind of like, like you know like whenever you need any help like you can you can call somebody…

AF_IDI_0002: For the tablet is it?

I_01 : For the tablet and app, yah.

AF_IDI_0002: Probably because there’ll be time whereby it just die off almost the, the tablet almost die off on me I forgot to charge it. [Participant laughed]

I_01 : Oh, oh is the charge it’s the charging part ok.

AF_IDI_0002: So yah, so if let’s say there is a, a number like whether to get it back to the interface that you guys intended to probably yah a, a number will be helpful just in case yah the, the, the tablet went hay haywire. [Participant laughed]

I_01 : Ok so subsequently when you got the tablet right do you see a doctor and did, did you bring like say did you see a doctor then you mentioned to them that, that you use a tab- you were using a tablet and a app?

AF_IDI_0002: No…

I_01 : Ok so you…

AF_IDI_0002: Like today I don’t I didn’t mentioned…

I_01 : Didn’t mentioned it ok.

AF_IDI_0002: I didn’t mentioned.

I_01 : Ok, ok then the next one will be the your overall acceptability of this how do you feel about the duration of the study?

AF_IDI_0002: Duration the 6 weeks?

I_01 : Yah.

AF_IDI_0002: It’s fine it’s ok, yah.

I_01 : Ok would it…

AF_IDI_0002: The duration is, is workable for me yah not too not too long or not too short.

I_01 : Ok and let’s say it were if it was not a study and you using it on your own you know like say to manage monitor your condition will you feel that like you it’s too long too short you know what will you feel is a good period of time?

AF_IDI_0002: My own usage?

I_01 : Your own usage, yah.

AF_IDI_0002: My own usage I wouldn’t…

I_01 : Ok you wouldn’t.

AF_IDI_0002: Yah I, I, I don’t think I would go for the tablet, yah.

I_01 : Ok, ok would you go for any other de- technological device like to help you or not?

AF_IDI_0002: Techno- well I’m using a, a, a watch like those tracker but…

I_01 : Ok so a tracker something like that?

AF_IDI_0002: Yah a tracker would what I mean is for me it’s, it’s helpful to just to track my heart rate and things like that but I understand it’s like it’s never 100 percent accurate just a gauge only, yah.

I_01 : So the that you mentioned accuracy right the gauge so you, you said that it’s not very accurate right would it would that will give you any like you know like thoughts about you know second thoughts about what you what you’re reading may not be actually what you have you know?

AF_IDI_0002: Yes but it’s but it’s just a gauge for me like I don’t need a I didn’t I don’t need a full accurate reading so that I can know what to do [Participant and interviewer laughed] yah because it’s like you mainly you don’t really know what to do. You just oh it is for my tracker it’s like I realized it’s I have my palpitation has been above 120 for constantly for like roughly about a few hours so that kind of information when I see a doctor probably I will just mentioned it to doctor. This is this is what a, a tracker will be helpful to give me a rough gauge it’s like but I won’t tell the doctor it’s like to 121 I’m at or [Participant and interviewer laughed] or 130 that I’m at yah so…

I_01 : So you…

AF_IDI_0002: Yah correct so it’s just a gauge for, for us if it’s very accurate reading is like I don’t think as a general user I, I, I need I need that, yah. [Participant laughed]

I_01 : Ok so you all you really need is a, a gauge that will be that’s good enough where you feel that you know that’ll be you, you feel you feel more com- confident and comfortable…

AF_IDI_0002: Yah correct like even for BP machine a gauge of whether I’m having a hi- my press- blood pressure is a bit high or blood pressure a bit low for me but it it’s also just a gauge yah so…

I_01 : Ok, ok that’s sufficient.

AF_IDI_0002: That’s sufficient, yah.

I_01 : Ok but also like were there other things like maybe the portability and mo- the like in terms of it being rea- more easier to bring around all that also?

AF_IDI_0002: Yes definitely.

I_01 : That matters?

AF_IDI_0002: Yah like the oximeter that I can bring around so of course it’s like the oximeter is more accurate than my tracker so that one is I, I, I will bring around when I’m really having like severe palpitation by then, yah.

I_01 : Ok, ok can we already mentioned it a bit earlier right ok so a few more questions ok would you prefer the contents to be delivered to you electronic or non electronic? Like let’s say there were another option of like say you know the, the contents of the tablet right when, when you are doing this study right the this electronic right so would you prefer like other forms of like the information given to you?

AF_IDI_0002: I, I preferred the electronic one rather than…

I_01 : Than paper and…

AF_IDI_0002: You record your own whereby yah you probably will misplaced all those information I like the idea that the information just sent directly rather then, then and we have to bring it to then after that you study I don’t know. [Participant laughed]

I_01 : Yah, yah, yah so that, that one you’re fine with, with that?

AF_IDI_0002: Yah correct.

I_01 : Ok, ok then ok so the let’s say this device right was introduced to you at the when you were first like diagnosed with AF right because you were looking at the timing also the timing of it being introduced will you think that it will be helpful for you? Like when you first diagnosed at the beginning right you think about your whole progression of this like what when you first found out and all that like when we introduced it during that time right do you think that it will be helpful for you? Or like or like maybe if we introduced it in the like when you are taking your medication already seeing the doctor in the middle so was would there be a timing particular timing that you feel it’s it will be like more appropriate for like say introducing this device? Other than you because you mentioned that you had abrasion you went through procedure that, that that’s that had helpful…

AF_IDI_0002: If so I think…

I_01 : But other times?

AF_IDI_0002: I think it will be helpful in terms for the reading in terms like different medication because some medication actually do interact with your BP reading so, so probably that would be helpful to track yah, yah correct.

I_01 : Ok, ok so like finally right is there any particular feature that you feel that you know could be added in or taken out to improve this experience that you had with this app?

AF_IDI_0002: Added in added in like?

I_01 : Like maybe a particular something that you would like to see that is more maybe could be more interactive more dynamic you know that you know given what you had used right in the past in the past 6 weeks right could make it more you know more…

AF_IDI_0002: Interactive?

I_01 : More, more useful for you and helpful for you to improve it because we are looking for really the features that can be improved?

AF_IDI_0002: Oh probably I don’t know just…

I_01 : Just say it…[Interviewer laughed] everything you have. [Interviewer laughed]

AF_IDI_0002: Ok probably in terms of like a game thing I, I, I don’t know like for example…

I_01 : Ok, ok every yah…

AF_IDI_0002: Rather than like giving information on what to eat and things like that probably they can come in the form of game [Participant laughed] like what you choose tonight, [Participant laughed] yah.

I_01 : Ok what any other ways that we can maybe make it more, more, more interactive?

AF_IDI_0002: Interactive?

I_01 : Yah or, or more encouraging?

AF_IDI_0002: I know, I have no idea [Participant and interviewer laughed] probably audio…

I_01 : Ok audio, audio…

AF_IDI_0002: But, but it can be creepy when it’s… [Participant and interviewer laughed]

I_01 : So the audio at audio at the right moment the right time not, not any not just au- any other audio…

AF_IDI_0002: Yah it, it probably audio or anything yah.

I_01 : Ok what about the videos?

AF_IDI_0002: Videos, videos is, is it’s good yah but as I said you for me I realized that it’s like more for the aging population the, the elderly of course I’ll begin what I am talking about… [Participant laughed]

I_01 : Of course but you’re still young.

AF_IDI_0002: The elderly in the sense in that sense right so sometimes you for me I can be a little bit impatient with the video right.

I_01 : So you don’t maybe for like a younger generation you feel that it will won’t be really that appealing?

AF_IDI_0002: Yah it, it, it’s just fun to watch [Participant and interviewer laughed] you have time you watch then if not then it’s like ok I won’t follow the exercise one, two, three… [Participant and interviewer laughed] you know that’s why I just get a rough gauge it’s all what is what is, is the exercise that is needed to be done and, and get it. [Participant and interviewer laughed] Yah.

I_01 : Ok sure ok any other comments any other thoughts about…

AF_IDI_0002: No more I think yah but it’s like if it is just for particular one as I said if it’s for one app for one particular reason then one tablet then is a little bit too much gadget [Participant laughed] that it yah but other than that it’s fine, yah.

I_01 : Ok, ok alright so anyway we want to thank you for like taking part…

AF_IDI_0002: Welcome!

I_01 : In this ok so I just stopped the recording.

AF_IDI_0002: Yah.

End of interview
