# Supplementary material for: Patient perspectives of the Self-management and Educational Technology tool for Atrial Fibrillation (SETAF): A mixed-methods study in Singapore
Source: PLoS One. 2022 Jan 21;17(1):e0262033. doi: 10.1371/journal.pone.0262033 (PMC8782297; doi:10.1371/journal.pone.0262033)
Supplement: S1 Data — (ZIP) [file pone.0262033.s006.zip › AFP2_IDI_0001.doc]

Participant ID number : AF_IDI_0001

Interviewer Number : I_01

Length of interview : 35.11 mins

I_01 : Ok so the first question is that how long have you been diagnosed with AF or irregular heartbeat and how did you find out about it from the beginning?

AF_0001 : From young when I was primary 4, I went for sport practice then suddenly I got like stomach pain and then I got fever high fever and then I got ulcers on my mouth all these. Then I go to polyclinic and the doctor said I have to go hospital “A” for the how to said like you know the result one so I went there then they do for me x-ray then they find out that I, I have irregular heartbeat. So from there my condition is not that good always sick always sick like sometime one week I went to school that, that time I was in when the thing serious is I already went to secondary school. So even in primary also I always in and out of hospital then after that when I go to sec. one, sec. 2 is become more serious even sometime I get easily to get sick like dengue fever all these then after that the doctor keep on asking whether I want to do for the operation. But my mom don’t allow me you know right last time…

I_01 : Scared right?

AF_0001 : I scared, they said don’t want so I’m the one suffer so I said at first I thought yah, maybe I also don’t want I also scared right then my mom said don’t want don’t want if anything happen to you then what will happen to me my mom said I said. Then I said ok then just, just follow what she say every, every month go for check up all these and then after that slowly when I was in sec. 2 I quit my school because sometime I go to school one week one day only. And then sometime 2 days even my principal wrote a letter told my mom said that I played truant run from school this and that but my mom go to the school and then explained to the principal and said no actually my daughter at home she’s sick this and that. So the doctor I mean the principal know then I got excused even my P.E. also they, they won’t let me to do then slowly when I was in sec. 2 I quit my, my school then I take the operation. That was in hospital “A” ey Hospital “B”...

I_01 : When was that?

AF_0001 : I think it was 19 if I’m not mistake is 1972 I think.

I_01 : So it was very long ago?

AF_0001 : And then the doctor asked me again said whether I want or not then I just tell my mom I said yah I want to go for it then my mom said no, no I said no I am the one who suffer I said never mind I said die, die [Interviewer and participant laughed] I said then…

I_01 : But she, she must be very scared right, right?

AF_0001 : Then my mom said ok if in that case then if you want because you are the one who suffer but the doctor also advice said at young age is good go for the operation instead of getting old right so I said never mind pray hard then I go for the operation.

I_01 : So ok already?

AF_0001 : Yah I, I went I stay about one month plus then after that ok then have to go follow up check up…

I_01 : Follow up, yah.

AF_0001 : 3 month and later 6 month all these then have to go under medication have to take medicine until my age 21 but [Participant laughed] in between before I reach 21 already quit I didn’t go to the hospital [Participant laughed] then I just stopped. Then after that I got married is it ok to tell you…

I_01 : Yah, yah it’s fine.

AF_0001 : After I got married then after that my first child just now my son my first child that was I think 8 month plus I got suspect hypertension just a suspect because maybe headache or what I don’t know. Then after that admitted inside the hospital “A” then from hospital “A” they sent me to hospital “B” then the doctor said everything ok so normal birth nothing because one of my friend is the same case but she already got one kid then when she had this same sickness like me right the doctor said she cannot give birth another anymore that means. So I thought maybe my life will be the same… [Interviewer and participant laughed]

I_01 : But no, no ok, ok.

AF_0001 : No, then the doctor said the doctor told me everything is ok but my first child caesarean then after that when I gave birth to my second child its still ok then my third child is ok. But when my age come to fifty plus…

I_01 : Yah is it current around this time you know?

AF_0001 : Yah when I was fifty plus then I start like got chest pain like very hard to breathe maybe overwork tired or what I don’t know and then…

I_01 : And also age?

AF_0001 : Due to age wear and tear [Interviewer laughed] then after that I went to hospital “C” and then admitted, admitted and then they find out my heart still come back to the same like before then after that they so I just go follow up follow up…

I_01 : Medication…

AF_0001 : Medicate and beside that I also had what gallbladder I got gallbladder because at first is the heart then slowly go for screening everything scan this and that find out gallbladder…

I_01 : Gallbladder.

AF_0001 : [Interviewer and participant laughed] and then after that they said I have to take out. But I’m using the bag for 6 month and then after that…

I_01 : Take out already?

AF_0001 : Yah take out, they throw everything then after that because when I had this gallbladder my heart also got problem at that time right so it’s very hard actually I have to go for the operation for the ballooning at hospital “D”…

I_01 : Ablation right?

AF_0001 : I have to go there but then because of this bag I cannot do this operation so dragging 6…

I_01 : Have to wait until finished first?

AF_0001 : Yah 6 month and then until the bag came, came out itself also you know yah 6 month…

I_01 : I see I see.

AF_0001 : Just carry here carry there so troublesome then after that doctor “A” said ok last year July fifth I think do the ballooning…

I_01 : Ok did it already?

AF_0001 : Then after do the ballooning then I go for the gallbladder after the gallbladder now they suspect I got liver so pek cek. [ Means “frustrated” in English]

I_01 : But one by one right you know right so it will improved like you know treat one by one like that you’ll be get better right?

AF_0001 : That, that is what happened to me.

I_01 : Yah so after that when you when you were trying to maintain after you did the ablation right your heart one right then you how do you maintain like you go back to doctor to get medication…

AF_0001 : Yes I have to continue my medication like water retention and then potassium all these for the heart and then blood…

I_01 : Blood pressure…

AF_0001 : Blood pressure and then I also take warfarin.

I_01 : So the ok then where in this time period right where do you get the information to like say you know helped you to manage your condition other that the doctor like the doctor will give you some advise about what to do right and also the medication what to take right? So other than that do you where do you get any other information like say diet…

AF_0001 : Information…

I_01 : Exercise other things…

AF_0001 : Yah dietician I went also and then even the pharmacist also I went they also give me advise not to take this not to take that right so I just, just follow then before my weight is 81 but now already 67 yah because…

I_01 : Ok big, big changed?

AF_0001 : Yah because of the maybe sick too sick then lose weight but good I prefer then I got knee also…

I_01 : Now?

AF_0001 : Not now I, I had it for…

I_01 : During, during that time?

AF_0001 : So long already.

I_01 : A long time ok, ok.

AF_0001 : Before my they find out about my heart already had this and then because of the knee problem also I have to lose weight right but cannot and then because of this sickness I really lose weight. [Interviewer and participant laughed]

I_01 : So in the end also you, you also have to lose because of all these things then you, you ate less and all these going through all these treatment all that right, yah.

AF_0001 : Then even the liver doctor told me this they sent me to dietician then asked me to cut down oily food and then try to bring down my weight again and then do exercise all these but only I’m only like if I walked too far I got my leg problem and then furthermore sometimes I like breathless too, too far…

I_01 : Yah too far.

AF_0001 : Yah that’s why I have to walk slowly and then exercise I don’t know what exercise I should do… [Interviewer and participant laughed]

I_01 : So do you ok so other than doctor, dietician right they do you have any other ways of getting information…

AF_0001 : No.

I_01 : So like if say TV, newspaper, online, your friends, family do they give you any like you asked them from them or not?

AF_0001 : Yah my, my family my family…

I_01 : Ok so, so how do you all help one another?

AF_0001 : They always advise me tell me not to do this not to do that then tell me don’t always stressed yourself this and that try to like overcome anything like relax don’t like don’t kan cheong [Means “excited” in English] all these [Participant laughed] tell, tell me. So don’t be stressed if anything you think you have something to, to say or what just share with us then lose all your tension this and that then asked me to eat good food like healthy food…

I_01 : Healthy.

AF_0001 : And said yah...

I_01 : Your family meaning is your just…

AF_0001 : My sis- my sibling…

I_01 : Sibling ok.

AF_0001 : We are very close.

I_01 : Very close ok so they also they come, come here often?

AF_0001 : Yah when, when I was sick they come and help me to the housework cooking all.

I_01 : Very good very good very nice.

AF_0001 : One thing my not say I want to say good about my family [Interviewer laughed] but my sibling is very good we are I mean we are very close our bonding that’s why ok I’m, I’m very happy also to have all my sibling with me…

I_01 : Everyday helps you a lot also feel better also right, ok so…

AF_0001 : Especially my youngest sister everyday called me everyday every morning without fail called me sometime after work also called me asked me how is my condition then asked me any if I got anything or what let me share. And then just tell her this and that at least I got someone because sometime family you know when we stay family like sometime we stressed right sometime kids problem this and that right but ok so far better.

I_01 : Better right ok so actually we go a little bit to the tablet you know you using this tablet the past 6 weeks so actually like how do you use it on a av- on a typical day and a average day how do you use it what’s the time that you normally use?

AF_0001 : Normally morning…

I_01 : Morning?

AF_0001 : I will used morning because morning I will take break- I have to take this medicine before food half an hour before food so I take around 8 o’clock sometimes sometime earlier but then sometime I busy right prepare things maybe not say half an hour more than half an hour then I take breakfast. Normally my breakfast will be around 9 I after my breakfast only I will take my medicine after I take my medicine then I will do this, this tablet I used this tablet…

I_01 : Ok so this is…

AF_0001 : After I relax after makan [Means “meal” in English] normally one hour after my, my breakfast one or one hour plus like that.

I_01 : So you rest first then you feel more like relax…

AF_0001 : More time…

I_01 : And comfortable then you take ok, ok so how often do you use it on a every like everyday?

AF_0001 : Normally everyday every morning after, after breakfast one, 2 hours like that and then only sometime if I didn’t used is because I walked early like let’s say I go market then I come back so late sometime forgot already then I, I missed I missed if not I will do everyday.

I_01 : Everyday so quite consistent ok, ok.

AF_0001 : Yah and then that time I went Malaysia also about 3 days I never do also [Participant laughed] I did asked I did asked Miss X I said can I bring this along with me when I go oversea she said no need don’t trouble yourself just leave it she said when you come back then you do, ok! Then that day I went to Korea so Miss X came and took the thing on 2^nd^ I went on the 3^rd^ so that means the 6 weeks already complete.

I_01 : Complete yes yah ok.

AF_0001 : Then I got missed a few days because I, I go I go market sometime that’s why only that.

I_01 : And then you won’t take already after that just leave it for the day?

AF_0001 : Yah because I don’t know don’t know because Miss X told me best to do after breakfast one or 2 hours you rest already then after that you take I said ok, I just follow what she said and then when I missed out let’s say I go out early 8 o’clock or what then I missed out to do the thing only that.

I_01 : I see so Miss X when she gave you this advise and instructions is there anything else that she mentioned to you other than you know after breakfast yah but were there any other things that she said?

AF_0001 : No she only just said if let’s say if I can do it everyday it’s the best so down there they can know right and then if let’s say if I had something on then I missed out then it’s ok sometimes ok. [Participant laughed]

I_01 : Ok how long how long did she takes to tell you the mes- to give you the message the instructions to use the device at the beginning?

AF_0001 : At the beginning when she sent the things she already she told me I did asked her…

I_01 : How long the, the time when she talked to you about all these things?

AF_0001 : A few minutes I think…

I_01 : A few minutes ok, ok.

AF_0001 : About 20 minutes or half an hour like that she sits and talk and tell me how to use the device this and that I said ok.

I_01 : Do you think it’s helpful for you when she…

AF_0001 : To me it’s ok it’s ok at least I know right, at least yah you know no doubt I have my own but I don’t do it as a habit then the thing also won’t work anything right so if like this it’s a must for me [Participant laughed] it’s a job for me to do.

I_01 : I see.

AF_0001 : So that’s why I just follow what they instruct me to do then I just follow only.

I_01 : So let’s say if Miss X right didn’t really ok she came here and then let’s say she didn’t give you the instruction right will you still be able to use it on your own let’s say…

AF_0001 : Once if let’s say she, she tell me then I just follow I will I will follow I will just do.

I_01 : But if she came here right and then let’s say imagine she didn’t give you instruction you know she let she let you used it on your own straight away…

AF_0001 : I will called I will called and asked…

I_01 : You called and asked!

AF_0001 : Will asked and then give me the thing [Interviewer laughed] and then I don’t know then just keep for what right I have to like they give me the tablet so I must do my duty is to do like what they give me right so I do…

I_01 : So you…

AF_0001 : If not then they won’t know right they won’t know actually because they said is linked to the hospital right so it’s best for me that they know what happen to me like for my blood whether up and down this and that right and then at least got feedback right, that’s all.

I_01 : So you, you prefer I mean if you, you know that it’s linked to the…

AF_0001 : Hospital.

I_01 : The hospital right does that give you how do you feel about this how do you feel about this, this link you know?

AF_0001 : About the link yah I, I feel like how to say it’s good for me it’s good I mean that at least they know what happen and then at least I, I also do my part and they also they if anything they will call me or maybe I don’t know may- whether they will call me or not. But I think in that way maybe if they see something is not right maybe they can call me or, or maybe they just message me give an advise or what then I’ll just follow.

I_01 : I see and there were also, also like popped up messages like in word those encouraging messages right how do feel about this do you do you feel like it’s useful for you…

AF_0001 : Yah useful…

I_01 : Helpful for you or not so?

AF_0001 : Useful…

I_01 : Useful or not!

AF_0001 : Useful and helpful also at least I know right because sometimes we don’t know much also right at least from this we, we know right I mean ok at least be more careful right?

I_01 : So how do you feel when you see all these messages do you have any feelings or not like, like any emotions that, that came out when you fee- see all these messages popped up all the time?

AF_0001 : No.

I_01 : As a it’s ok?

AF_0001 : For me it’s ok because it’s a lesson, lesson for me to know like knowledge like they…

I_01 : Yah knowledge, yah.

AF_0001 : Yah after all yah I only that I got sickness but I don’t know much about the knowledge like what happen this and that like they give me videos and then they give me message at least I know, know I mean I mean I know more. Instead of like oh I only know that I sick normally people are like that right especs- especially like old people like me… [Interviewer and participant laughed]

I_01 : No you are not. [Interviewer laughed]

AF_0001 : Sick…

I_01 : You are not!

AF_0001 : Sick [Interviewer and participant laughed] but we don’t know why the consequence all these right and then when I have all these at least I know and sometime I share with my, my kids oh you see right they give me this one this one. Sometime they asked me, Ma what they write Ma, I didn’t know you see and sometime my daughter read, oh good Ma! I said yah, yah good at least I know you see because yah sometime we want to ask so much question to like we go hospital this and that sometime we never come out you know. Like never think you know but when we go back, forget to ask doctor this and that at least like this one they give me knowledge at least I know.

I_01 : You know so it’s kind of give you like even if you forget it’ll still help you…

AF_0001 : Yah, yah.

I_01 : Ok, ok then what about like the videos how do you feel about the vi- anything that you like any videos that you like or don’t like which one is your favorite?

AF_0001 : I forgot [Interviewer laughed] forgot.

I_01 : I mean there’s the diet one there’s the, the cooking one there’s the exercise one which or the AF one…

AF_0001 : The AF one…

I_01 : The, the have a few right?

AF_0001 : The AF one.

I_01 : So you, you watched that one you was that too long for you the video?

AF_0001 : Not really.

I_01 : Not really it’s ok?

AF_0001 : Yah it’s ok.

I_01 : Ok then the blood pressure monitor right like when you, you said you used everyday right?

AF_0001 : Yah I used.

I_01 : So everyday when you used it how do you like were there any how do you feel when you ss- when you start using it like it does you find it easy, difficult?

AF_0001 : Easy.

I_01 : Easy ok then what about like do you feel any stress or anxiety when using it or not like get, get a bit anxious?

AF_0001 : No, no, no.

I_01 : Oh so it’s ok.

AF_0001 : Only sometimes like today go up go down only like that right because down there they, they write down right and then so we know and then we can checked back refer again you see?

I_01 : Correct.

AF_0001 : Today very high this one very low sometime then from there maybe I take my food maybe too oily or what so just from there I just balanced my, my food try to balance my food.

I_01 : So tracking the, the result will also kind of also affect how you eat you know when you look at your results right if like too high then you try to adjust a bit…

AF_0001 : Yah, yah adjust yah!

I_01 : Ok, ok so, so you like adjust your food?

AF_0001 : Yah adjust because or maybe even my blood also sometime I check because my sister she had that thing right so sometime when I go there I, I checked sometime if too high then cut down whatever can cut down the food just even like for the blood also if too high maybe less salt. And then like nonsense, nonsense things [Interviewer laughed] like titbits all these don’t take.

I_01 : What about like your sur- will also be that there was some surveys right the, the survey they asked you some questions you know did you do the survey?

AF_0001 : Because Auntie not clever [Interviewer laughed] it is that’s why I want to do things that I can I just put a tick then those I cannot…

I_01 : Understand…

AF_0001 : Understand I just stopped there.

I_01 : Ok so some of it was maybe like you don’t really understand the question and everything ok, ok the survey you when you was it too long or not the survey you feel the…

AF_0001 : No, no…

I_01 : It’s ok?

AF_0001 : Ok the survey ok.

I_01 : Ok then…

AF_0001 : Because Auntie nothing to do what [Interviewer laughed] morning Auntie after finished everything sit then I do the tablet go and checked, checked everything sometime free time afternoon also go and checked got message or what only like that.

I_01 : So when you have time then when you don’t know sometime sometimes you asked your daughter or what also?

AF_0001 : Yah I did sometimes I did asked then she helped me then she, she explained Ma this one and this one ok, ok…

I_01 : But only sometimes only not every time ok, ok [Interviewer and participant laughed] ok, then the next one is that you how do you find about the tablet the screen size is it too small?

AF_0001 : No, no it’s ok.

I_01 : Too small ok just right?

AF_0001 : Just right!

I_01 : Ok then what about like the font the font size meaning that the words too big or too small?

AF_0001 : Ok for me ok I just use this one [Interviewer and participant laughed] without this yah cannot like that…

I_01 : So it helps a bit then what about the color, color was it too bright or too dark or…

AF_0001 : No, no, no it’s ok.

I_01 : Ok also ok then what was your which one do you feel was like the easiest to do the easiest to use for the entire you know all these the tablet the app the blood pressure monitor right all of these which one was the easiest you feel was to use?

AF_0001 : To me the tablet ok easy then even the blood pressure that one also is easy also…

I_01 : It’s ok.

AF_0001 : It’s ok!

I_01 : So you…

AF_0001 : Simple only what.

I_01 : So you don’t find it like too difficult or what?

AF_0001 : No, no, no only that one, answer…

I_01 : Only the survey that one ok, ok [Participant laughed] get it I get it it’s a bit too difficult that one ok, ok, ok so this is going to be further ok the do you know that there’s a number for you to call right the, the did you ever use it the number?

AF_0001 : No the tablet there right got number right because I find, find, find out that there’s nothing to for me to call and ask because everything I just follow ok should be no problem [Participant laughed] but I don’t know whether there’s a problem or not I don’t know. Only sometimes when, when I pressed the thing home this and that only they said sorry because doesn’t linked or what and then later I checked again I got time I go and checked, ok already.

I_01 : So it’s like the connection I think…

AF_0001 : The connection because sometime after we check our blood pressure wait, wait, wait don’t have…

I_01 : Oh is it?

AF_0001 : So for…

I_01 : I think you have to try again is it?

AF_0001 : Yah I try again but then sometime also…

I_01 : Also don’t have, oh?

AF_0001 : Then have to wait again then I see oh ok already.

I_01 : Oh so it’s it will take some time you feel it’s too is it very long the waiting time?

AF_0001 : No, no, no not really.

I_01 : How long around how long?

AF_0001 : Few minutes only.

I_01 : Few minutes but still have to wait I think.

AF_0001 : Yah still have to wait because sometime already do sit checked, checked, checked how come don’t have [Interviewer and participant laughed] haven’t then pressed home this and that then ok, then later come out ok when come out already right Auntie happy Auntie oh this one ok already. [Interviewer and participant laughed]

I_01 : This one is the, the recording of the of the results the tablet right, ok it take sometime ok, ok so the next one is that do you find that the with a number there right is it helpful for you the number is helpful for you or not?

AF_0001 : Which number?

I_01 : The number on the tablet for you to call one?

AF_0001 : Yah, yah is helpful also.

I_01 : Do you think it’s a useful thing or not?

AF_0001 : Yah it, it…

I_01 : I know you never used but…

AF_0001 : Yah I think…

I_01 : Let’s say it is just there.

AF_0001 : It is yah it is because for that time when Auntie [Interviewer laughed] used the tablet is that no problem so I don’t have to call and then if let’s say anything happen to the tablet then I must call so the number is help, help if I have the number. If don’t have then the thing just leave like that because Auntie don’t know who to call right wait until they come to collect there’s no story.

I_01 : Yah, yah so you just leave it there you just leave it there ok.

AF_0001 : Then if got number if anything happened we can just called said the tablet not working or whatever right so we can let them know right then if there’s no number then how to call them then so the thing just leave it there, for me that is what I think.

I_01 : Yah ok, ok then the how do you feel about the overall duration of the study was it too long too short how do you feel 6 weeks right was it…

AF_0001 : No, no, no for me ok, ok nothing like just like housework homework like that nothing.

I_01 : Ok means too it’s just right or…

AF_0001 : Just right.

I_01 : Just right ok!

AF_0001 : Ok because only when I go out I feel bad when I, I never take my blood pressure I feel bad because already my duty to do right [Interviewer laughed] then I missed out one day don’t know whether they that’s why I don’t know Auntie also know that time mistake never called or asked this Miss X. Said that if let’s say morning I cannot do maybe later or maybe late later like quite late or what still can take the blood pressure or not I don’t know?

I_01 : Actually can you know…

AF_0001 : Can… wasted! [Participant laughed]

I_01 : Can it’s any anytime no but it’s ok doesn’t matter one because it’s just the average right yah so but actually can also but Miss X didn’t tell you that?

AF_0001 : I didn’t asked her.

I_01 : But she didn’t say at the beginning?

AF_0001 : She, she just say she, she tell me say that if, if the best time is one, 2 hours so I take that words and then said but I don’t remember whether she said after that later can do or not I don’t remember that.

I_01 : She only mentioned the best time.

AF_0001 : The best time!

I_01 : Ok, ok, ok can then the next is the ok so you prefer to use it in a like tablet because you know we have many different kind of devices nowadays to track your you know your heart, your blood pressure right? You prefer it in tablet form in phone form or in a computer form or you prefer like paper form non, non electronic form is there any kind of device you prefer?

AF_0001 : I think tablet and phone also ok.

I_01 : Phone also ok phone, handy yah?

AF_0001 : Handy so everywhere I can bring along right so I don’t have to rush home let’s say if I go out or what only that.

I_01 : So if, if mo- if it’s mobile is better you feel oh ok.

AF_0001 : Yah but tablet also like that day the that size it’s still ok for me to carry no problem but only thing the blood pressure that one…

I_01 : It’s quite it’s big!

AF_0001 : Bulky right?

I_01 : Yah bulky yah but will you be able to do you mind like carrying it around in a you know to other places if you go out you know like visit your relative all that?

AF_0001 : Can, can no problem.

I_01 : You’re ok, ok with it.

AF_0001 : But that, that time Miss X said no need if want to go out then just leave like that ok. [Participant laughed]

I_01 : Ok, ok, ok then if let’s say if it’s a non pap- non electronic form right you know paper form like the survey we give you in paper form you know the, the educational messages we give you in paper form would you prefer that?

AF_0001 : I’m still not clever… [Participant laughed]

I_01 : Paper mean meaning like it’s the same but it’s just we give you paper non, non electronic…

AF_0001 : Yah I am yah I know but then sometimes the question I don’t understand like asked me to write down ok my blood pressure this and that ok and like asked me like the what survey all this…

I_01 : Survey yah… this…

AF_0001 : Jia lat [Means “bad” in English] Auntie cannot do. [Participant laughed]

I_01 : Only the survey but let’s say we just give you paper to write down the your blood pressure keep track of it it’s ok?

AF_0001 : It’s ok.

I_01 : Ok it’s ok, ok, ok then a few last few questions so when will you think it will be the best time to introduce right this device to you, you know because you had this AF quite long already and then you had operation also which is quite recently. And then do you think that when will when would it be right the best time to have this device say introduce to you throughout this time period you know to help you to maintain to, to, to monitor your condition?

AF_0001 : I think earlier.

I_01 : Earlier, earlier better?

AF_0001 : Yah earlier better.

I_01 : Like when?

AF_0001 : Before I have my operation. [Participant laughed]

I_01 : Ok, ok before that?

AF_0001 : So maybe it can help me so maybe I don’t have to go for the operation only thing that, that time don’t have right?

I_01 : That time yah, yah.

AF_0001 : So for now also it’s ok still ok.

I_01 : Still ok.

AF_0001 : At least got something right rather than just like that nothing right so I also don’t learn anything I also don’t know only, only Auntie know Auntie got, got AF only this, this, this sickness only.

I_01 : But after your operation you won’t mind using it you know and like at this time period you’re ok with it?

AF_0001 : Ok, if asked me to do like what they sent me to do what that day right the tablets and the blood pressure Auntie don’t mind ok if let’s say…

I_01 : The tablet sent blood pressure what do you mean?

AF_0001 : I mean the, the tablets…

I_01 : You…

AF_0001 : And the blood pressure test that one if they asked me to use I don’t mind I if go anywhere also I can carry.

I_01 : Ok and you don’t mind using it for as long as possible like would there be like a time period like you feel like it’s good it’s a good time to use you know?

AF_0001 : Sorry…

I_01 : Yah don’t worry?

AF_0001 : Hello bahru ada orang datang [Means “someone came here” in English] interview nanti bahru, bahru [Means “Later, just, just” in English] ok bye ok bye, my sister.

I_01 : Ok you are very close right you all [Interviewer and participant laughed] very nice she asked you a lot of things yah so like…

AF_0001 : Yah she asked me whether you come already or not I said yah just only I said. [Interviewer and participant laughed]

I_01 : Yah finishing already anyway so the time period meaning like you know if let’s say the device was given to you and will there be a like a time period or it could be like forever, I mean like you know as long as you can used, used it that you prefer to use?

AF_0001 : I think how to say should be no problem for me should be no problem…

I_01 : Ok no problem ok.

AF_0001 : No problem, for me just simple things like that should be no problem, how long also it doesn’t matter.

I_01 : Doesn’t matter ok, ok.

AF_0001 : Its for my own good also it helped me right so I don’t mind.

I_01 : Ok then the finally right any you know other thoughts or opinion to help us improve this tablet right and you feel that any features can be improved or anything you can add on to it right to make it better [Interviewer laughed] your own opinion, can be anything can be anything.

AF_0001 : Yah like, like Auntie, Auntie worried about my I worried about my you know cholesterol but I don’t know [Interviewer laughed] how cholesterol have to go for blood test right?

I_01 : That’s right.

AF_0001 : That’s why that’s the main point sometime even if I go check up also they seldom checked for my cholesterol then it make me worry also because sometime we eat we also don’t know we eat this one more or less then whether the cholesterol high or low right? Then as what I know but I don’t know it’s true or not the cholesterol medicine is not that good for kidney I, I heard they all said.

I_01 : Yup it will affect in fact.

AF_0001 : That’s why I try to cut down but I don’t know whether it’s ok or not but that’s why I’m worried about that.

I_01 : You worried about this so like if there’s a feature can kind of helped you in this also it’s ok it’s good?

AF_0001 : Yah it’s good.

I_01 : Ok so, so cholesterol any, any other things that possible can helped you also like you can add into the as a feature to the tablet?

AF_0001 : Like what?

I_01 : Like other than cholesterol is there any other things that you want to look you want to see that hopefully the future tablet will have or not? [Interviewer and participant laughed]

AF_0001 : Hope you all improved, improved more but I don’t know how to say also [Participant laughed] because for me this one is helped is already helped me a lot also, I’m so happy also and thank you to you all also.

I_01 : No thank, thank you actually because we, we really want to hear your, your, your opinion and thoughts yah you are the one who really, really used it right you know on the ground [Participant laughed] so we really appreciate it. [Interviewer laughed] Ok so that’s the end I, I have here so I just stopped the recording.

End of interview
