# Supplementary material for: Patient perspectives of the Self-management and Educational Technology tool for Atrial Fibrillation (SETAF): A mixed-methods study in Singapore
Source: PLoS One. 2022 Jan 21;17(1):e0262033. doi: 10.1371/journal.pone.0262033 (PMC8782297; doi:10.1371/journal.pone.0262033)
Supplement: Attachment — Submitted filename: Response to reviewers.docx [file pone.0262033.s007.docx]

September 10, 2021

Re: Resubmission of manuscript PONE-D-21-19770 (*Patient perspectives of the Self-management and Educational Technology tool for Atrial Fibrillation (SETAF): a mixed-methods study in Singapore*)

Dr Muhammad Junaid Farrukh

Academic Editor

PLOS ONE

Dear Dr Muhammad Junaid Farrukh,

Thank you for the opportunity to revise our manuscript entitled, “*Patient perspectives of the Self-management and Educational Technology tool for Atrial Fibrillation (SETAF): a mixed-methods study in Singapore*”. We appreciate the careful review and constructive suggestions. It is our belief that the manuscript is improved after making the suggested edits.

We have dutifully responded to every comment. We have addressed all the points brought out by the reviewer to the best of our ability. The changes and our rebuttals are presented after each comment. Changes made in the manuscript are tracked. The page and line numbers mentioned in our response are according to the revised manuscript.

This revision has been developed in consultation with all co-authors, and each author has given approval to the final form of this revision. Thank you for your consideration and we look forward to your favourable response

Sincerely,

Dr. Brigitte Woo

Research Fellow,

Alice Lee Centre for Nursing Studies,

Yong Loo Lin School of Medicine,

National University of Singapore.

**Editor’s comments to author**

1. Please ensure that your manuscript meets PLOS ONE's style requirements, including those for file naming. The PLOS ONE style templates can be found at 
<https://imsva91-ctp.trendmicro.com:443/wis/clicktime/v1/query?url=https%3a%2f%2fjournals.plos.org%2fplosone%2fs%2ffile%3fid%3dwjVg%2fPLOSOne%5fformatting%5fsample%5fmain%5fbody.pdf&umid=04E8D6E5-CB5D-1405-9D93-56F7C9BF425C&auth=6e3fe59570831a389716849e93b5d483c90c3fe4-555acbf8fc5d83da67da269c4cb2d79d882a2059> and 
<https://imsva91-ctp.trendmicro.com:443/wis/clicktime/v1/query?url=https%3a%2f%2fjournals.plos.org%2fplosone%2fs%2ffile%3fid%3dba62%2fPLOSOne%5fformatting%5fsample%5ftitle%5fauthors%5faffiliations.pdf&umid=04E8D6E5-CB5D-1405-9D93-56F7C9BF425C&auth=6e3fe59570831a389716849e93b5d483c90c3fe4-8c47293dd5dd6502c7a8c7993d1ba4a88f1d6711>

Response: The manuscript has been revised to meet PLOS ONE’s style requirements

2. Please include additional information regarding the survey or questionnaire used in the study and ensure that you have provided sufficient details that others could replicate the analyses. For instance, if you developed a questionnaire as part of this study and it is not under a copyright more restrictive than CC-BY, please include a copy, in both the original language and English, as Supporting Information

Response: The survey used in this questionnaire has been included as Supporting Information (S1. File)

3. When reporting the results of qualitative research, we suggest consulting the COREQ guidelines  or other relevant checklists listed by the Equator Network, such as the SRQR, to ensure complete reporting (<https://imsva91-ctp.trendmicro.com:443/wis/clicktime/v1/query?url=http%3a%2f%2fjournals.plos.org%2fplosone%2fs%2fsubmission%2dguidelines%23loc%2dqualitative%2dresearch&umid=04E8D6E5-CB5D-1405-9D93-56F7C9BF425C&auth=6e3fe59570831a389716849e93b5d483c90c3fe4-860c02cb43b02a39d38719c1b99d0fb90a836b38>). Moreover, please provide the interview guide used as a Supplementary File.

Response: SRQR was used in the reporting of this study as mentioned in line 115 and the completed checklist is submitted as Supporting information (S2 File). The interview guide is also included as Supporting information (S2 File).

4. Please provide additional details regarding participant consent. In the ethics statement in the Methods and online submission information, please ensure that you have specified (1) whether consent was informed and (2) what type you obtained (for instance, written or verbal, and if verbal, how it was documented and witnessed). If your study included minors, state whether you obtained consent from parents or guardians. If the need for consent was waived by the ethics committee, please include this information.
If you are reporting a retrospective study of medical records or archived samples, please ensure that you have discussed whether all data were fully anonymized before you accessed them and/or whether the IRB or ethics committee waived the requirement for informed consent. If patients provided informed written consent to have data from their medical records used in research, please include this information.
Once you have amended this/these statement(s) in the Methods section of the manuscript, please add the same text to the “Ethics Statement” field of the submission form (via “Edit Submission”).
For additional information about PLOS ONE ethical requirements for human subjects research, please refer to <https://imsva91-ctp.trendmicro.com:443/wis/clicktime/v1/query?url=http%3a%2f%2fjournals.plos.org%2fplosone%2fs%2fsubmission%2dguidelines%23loc%2dhuman%2dsubjects%2dresearch.%22%29%28&umid=04E8D6E5-CB5D-1405-9D93-56F7C9BF425C&auth=6e3fe59570831a389716849e93b5d483c90c3fe4-7648a745adfe6670965b5c7a2d02c4758e4d64b6> you can modify it if you want, just leaving the part about retrospective studies)

Response: We have further clarified that written informed consent was obtained for all study participants (line 163-164).

5. Please ensure that you include a title page within your main document. We do appreciate that you have a title page document uploaded as a separate file, however, as per our author guidelines (<https://imsva91-ctp.trendmicro.com:443/wis/clicktime/v1/query?url=http%3a%2f%2fjournals.plos.org%2fplosone%2fs%2fsubmission%2dguidelines%23loc%2dtitle%2dpage&umid=04E8D6E5-CB5D-1405-9D93-56F7C9BF425C&auth=6e3fe59570831a389716849e93b5d483c90c3fe4-82079791b1d8a8f6742f24408a79b447b3df4601>) we do require this to be part of the manuscript file itself and not uploaded separately.
Could you therefore please include the title page into the beginning of your manuscript file itself, listing all authors and affiliations

Response: A title page listing all authors and affiliations has been included in the beginning of our manuscript file.

6. Thank you for stating the following in the Acknowledgments Section of your manuscript:

"This study was funded by a Health Services Research New Investigator Grant (HSRNIG13nov002) from the National Medical Research Council, Singapore. We would like to thank Koninklijke Philips N.V. for loaning the tablets. Neither the funders nor Philips have any role in the study design, data collection, analysis and preparation of manuscript. We also thank Dr. Joanne Yoong and Dr. Luo Nan for their input in the study methodology. Lastly, we would like to thank the patients for their enthusiastic participation in the study."

We note that you have provided funding information that is not currently declared in your Funding Statement. However, funding information should not appear in the Acknowledgments section or other areas of your manuscript. We will only publish funding information present in the Funding Statement section of the online submission form. 
Please remove any funding-related text from the manuscript and let us know how you would like to update your Funding Statement. Currently, your Funding Statement reads as follows:

"This study was funded by a Health Services Research New Investigator Grant (HSRNIG13nov002) from the National Medical Research Council, Singapore."

Please include your amended statements within your cover letter; we will change the online submission form on your behalf

Response: The statement ‘This study was funded by a Health Services Research New Investigator Grant (HSRNIG13nov002) from the National Medical Research Council, Singapore’ has been removed from our Acknowledgments section.

7.We note that you have stated that you will provide repository information for your data at acceptance. Should your manuscript be accepted for publication, we will hold it until you provide the relevant accession numbers or DOIs necessary to access your data. If you wish to make changes to your Data Availability statement, please describe these changes in your cover letter and we will update your Data Availability statement to reflect the information you provide.

Please review your reference list to ensure that it is complete and correct. If you have cited papers that have been retracted, please include the rationale for doing so in the manuscript text, or remove these references and replace them with relevant current references. Any changes to the reference list should be mentioned in the rebuttal letter that accompanies your revised manuscript. If you need to cite a retracted article, indicate the article’s retracted status in the References list and also include a citation and full reference for the retraction notice.

Response: No papers in the reference list have been retracted. No addition or removal was made to the reference list. References 1, 3 and 4 have been updated to reflect the latest available statistics.

**Reviewer’s comments to authors**

Reviewer #1: Dear Authors,

Please kindly consider the following suggestions for further improvement of the manuscript:

1. Title: To change the term 'mobile health' to the actual tool used i.e. SETAF

Response: Thank you for your suggestion we have updated our title accordingly.

2. Methods: To include any specific info or features of SETAF that patients are required to explore during the 6-week duration. My concern is some patients may not explore at all or focus only 1-2 features that they like the most, given that there is a huge gap in the age of patients participated in this study. This is of course, unless, the researcher could remotely track all patients' use of SETAF throughout the duration.

Response: Information regarding the features of SETAF has been described in our Methods section under ‘Setting and sample’ (line 120-129) and summarised in Table 1. The patients were encouraged to explore all the features, however the research team did not dictate how they should do so in order to capture their actual usage behaviour. The patients’ actual usage behaviour was described in our Results section (line 382-405)

3. Methods: To include a more detailed inclusion and exclusion criteria for the patients. For example, background educational level, familiarity with using smart phones and apps etc. This could potentially affect the results of this study.

Response: The inclusion and exclusion criteria for the patients were intentionally broad to be able to capture the viewpoints of patients from diverse backgrounds and with varying experience using technology.

4. Data analysis: To correct the completion rate of the self-management triage assessment. I noticed there is an overlap between the values i.e., 25-50% and 50-75%. The info in the table 3 is correct.

Response: Thank you for your correction, we have amended line 189-191 to reflect this change.

5. Data collection: To include the detail on whether or not a consent is obtained prior to the audio-recording.

Response: We have added that audio-recording during the interviews were done with participant’s consent (line 156).

6. Results: There were different N used in the results section. In Line 365, it was mentioned that 33 participated in the survey, and not 38 as mentioned in the earlier section. Please clarify.

Response: In total, 37 participants were recruited to participate in this study. 4 participants declined to complete the survey, hence only data from 33 participants were available. The demographics and application usage data from these 4 participants were still included in the data analysis. We have updated our Results section to clarify this (line 215-217).

Reviewer #2: Participants ages were stated by the author begins from 25 years old , while the
mean mean age was nearly 65 years old. This age issue represent an acceptable conflict as this high variation in participants ages implies different motivations to use mobile health . The validity and reliability of the used mobile health wasn't mentioned in the study. The author didn't mention how intra co- researcher variability was overcomed. Regarding to sample size calculation was not mentioned , however sample size was small. statistical method was unclear and not obviously mentioned.

Response:

Although the initial inclusion criteria for the study was AF patients aged 21 years and above, the final age range of recruited participants was 41-78 years old as AF is more prevalent in the older population.

The validity and reliability of the SETAF were not reported in this study. However, these been explored and reported in an earlier study which contributed to the development of SETAF (Cher BP, Kembhavi G, Toh KY, Audimulam J, Chia W-YA, Vrijhoef HJ, et al. Understanding the Attitudes of Clinicians and Patients Toward a Self-Management eHealth Tool for Atrial Fibrillation: Qualitative Study. JMIR Human Factors [Internet]. 2020 Sep 17 [cited 2020 Oct 21];7(3):e15492.)

The interviews and surveys were done by the same co-author, thus minimizing variability during data collection. In addition, we explained that the co-authors discussed frequently to compare interpretations until consensus was reached for the themes and subthemes. (line 177-179, 201-202).

The primary objective of this study was to collect qualitative data on AF patients’ perspectives of SETAF, hence the sample was purposively selected and continued until data saturation was achieved. Participants who did not want to be interviewed were still recruited and data from the survey and application usage data were collected and included in the analysis.

Due to the small sample size, we did not perform any statistical test and reported our findings descriptively (line 182-192).
